# Potential and Limitations of ChatGPT 3.5 and 4.0 as a Source of COVID-19 Information: Comprehensive Comparative Analysis of Generative and Authoritative Information

**DOI:** 10.2196/49771

**Published:** 2023-12-14

**Authors:** Guoyong Wang, Kai Gao, Qianyang Liu, Yuxin Wu, Kaijun Zhang, Wei Zhou, Chunbao Guo

**Affiliations:** 1 Children's Hospital Chongqing Medical University Chongqing China; 2 Women and Children's Hospital Chongqing Medical University Chongqing China; 3 Guangzhou Women and Children's Medical Center Guangzhou Medical University Guangzhou China

**Keywords:** ChatGPT 3.5, ChatGPT 4.0, artificial intelligence, AI, COVID-19, pandemic, public health, information retrieval

## Abstract

**Background:**

The COVID-19 pandemic, caused by the SARS-CoV-2 virus, has necessitated reliable and authoritative information for public guidance. The World Health Organization (WHO) has been a primary source of such information, disseminating it through a question and answer format on its official website. Concurrently, ChatGPT 3.5 and 4.0, a deep learning-based natural language generation system, has shown potential in generating diverse text types based on user input.

**Objective:**

This study evaluates the accuracy of COVID-19 information generated by ChatGPT 3.5 and 4.0, assessing its potential as a supplementary public information source during the pandemic.

**Methods:**

We extracted 487 COVID-19–related questions from the WHO’s official website and used ChatGPT 3.5 and 4.0 to generate corresponding answers. These generated answers were then compared against the official WHO responses for evaluation. Two clinical experts scored the generated answers on a scale of 0-5 across 4 dimensions—accuracy, comprehensiveness, relevance, and clarity—with higher scores indicating better performance in each dimension. The WHO responses served as the reference for this assessment. Additionally, we used the BERT (Bidirectional Encoder Representations from Transformers) model to generate similarity scores (0-1) between the generated and official answers, providing a dual validation mechanism.

**Results:**

The mean (SD) scores for ChatGPT 3.5–generated answers were 3.47 (0.725) for accuracy, 3.89 (0.719) for comprehensiveness, 4.09 (0.787) for relevance, and 3.49 (0.809) for clarity. For ChatGPT 4.0, the mean (SD) scores were 4.15 (0.780), 4.47 (0.641), 4.56 (0.600), and 4.09 (0.698), respectively. All differences were statistically significant (*P*<.001), with ChatGPT 4.0 outperforming ChatGPT 3.5. The BERT model verification showed mean (SD) similarity scores of 0.83 (0.07) for ChatGPT 3.5 and 0.85 (0.07) for ChatGPT 4.0 compared with the official WHO answers.

**Conclusions:**

ChatGPT 3.5 and 4.0 can generate accurate and relevant COVID-19 information to a certain extent. However, compared with official WHO responses, gaps and deficiencies exist. Thus, users of ChatGPT 3.5 and 4.0 should also reference other reliable information sources to mitigate potential misinformation risks. Notably, ChatGPT 4.0 outperformed ChatGPT 3.5 across all evaluated dimensions, a finding corroborated by BERT model validation.

## Introduction

The COVID-19 pandemic, caused by the SARS-CoV-2, has had a profound global impact [1]. As of June 1, 2023, the pandemic has resulted in over 767 million reported cases and over 6.938 million fatalities worldwide, marking it as one of the most significant pandemics in human history [2]. The complex transmission modes, extended incubation period, atypical symptoms, and emergence of multiple variants pose substantial challenges for pandemic prevention, control, and treatment [3].

Efforts to prevent and treat COVID-19 continue unabated, and there is a high public demand for related information [4,5]. World Health Organization (WHO) [2], a leading authority in public health, has published a series of frequently asked questions about COVID-19 on its official website [6]. These frequently asked questions provide comprehensive coverage on various aspects of COVID-19, including basic knowledge, transmission modes, prevention methods, treatments, and its impact on different populations and environments [7-9]. However, the sheer volume of information, frequent updates, and potential language barriers may hinder access and comprehension, leading to misinformation [5,10,11].

The advent of artificial intelligence (AI) technology has seen the rise of dialog models that are gradually replacing traditional search engines. These models, based on large language models, use deep learning to generate natural language text in various formats, such as questions and answers (Q&As), summaries, and stories, based on user input [12]. ChatGPT, an advanced dialog model, leverages a large corpus and powerful neural networks to generate fluent, coherent, and logical text. It has found applications in numerous fields, including medical information provision, education, and scientific research, offering users convenient and efficient information services [13-16].

This study aims to assess ChatGPT’s capability as a COVID-19 information service platform, providing the public with accurate and relevant information about the virus [17,18]. This research not only evaluates the performance of ChatGPT in disseminating COVID-19 information but also offers insights into other informational services related to epidemics.

## Methods

### Ethical Considerations

This study was conducted in alignment with the Declaration of Helsinki and did not necessitate ethics committee approval.

### Study Design

The research was executed in 2 stages. In the initial stage, we extracted 487 questions related to COVID-19 from the WHO official website and used ChatGPT 3.5 and 4.0 to generate corresponding answers (Multimedia Appendix 1). Two clinicians were invited to score these generated answers, referencing the authoritative WHO responses. The scoring evaluated the quality of the answers across 4 dimensions: accuracy, comprehensiveness, relevance, and clarity. Each answer was assigned a score from 0 to 5 based on a predefined scoring standard (Table 1). Concurrently, we used the BERT (Bidirectional Encoder Representations from Transformers) model to compute the similarity score between the generated answers and the official WHO responses, with scores ranging from 0 (completely dissimilar) to 1 (identical).

**Table 1:** The scoring system (0-5) used for evaluating COVID-19 information from ChatGPT 3.5 and 4.0.

Criteria	0 points	1 points	2 points	3 points	4 points	5 points
Accuracy^a^	Completely wrong or irrelevant	Mostly wrong or irrelevant	Partially wrong or irrelevant	Few wrong or irrelevant	Mostly correct or relevant	Completely correct or relevant
Completeness^b^	Completely missing or redundant	Mostly missing or redundant	Partially missing or redundant	Few missing or redundant	Mostly covered or concise	Completely covered or concise
Relevance^c^	Completely deviated or unrelated	Mostly deviated or unrelated	Partially deviated or unrelated	Few deviated or unrelated	Mostly close or related	Completely close or related
Clarity^d^	Completely vague or ambiguous	Mostly vague or ambiguous	Partially vague or ambiguous	Few vague or ambiguous	Mostly clear or explicit	Completely clear or explicit

^a^Accuracy: measures the factual correctness.

^b^Comprehensiveness: evaluates the breadth or depth of information.

^c^Relevance: assesses how directly the information relates to COVID-19.

^d^Clarity: scores readability and understandability.

In the second stage, we conducted a quantitative and qualitative analysis of the first-stage data, comparing it with the official WHO information to assess the quality of the COVID-19 information generated by ChatGPT 3.5 and 4.0. This analysis facilitated a discussion on the strengths and limitations of the answers generated by ChatGPT 3.5 and 4.0 and allowed us to propose suggestions for improvement.

### Data Source

All questions and answers used in this study were sourced from the Q&A section about COVID-19 on the official WHO website. This website is a primary source of authoritative and reliable COVID-19 information, with its content undergoing professional and scientific review and updates. We extracted 487 questions covering various aspects of COVID-19, such as basic knowledge, transmission routes, preventive measures, vaccination, and travel advice, as samples for this study. These questions were input into ChatGPT 3.5 and 4.0 to generate corresponding answers, which were then compared with the official WHO responses to form the data set for this study. To mitigate the influence and bias of context association in information generation, we used 2 separate accounts, with each question being asked in a newly created dialog box. The complete list of prompts used for this purpose with ChatGPT 3.5 and 4.0 can be found in Multimedia Appendix 2.

### Data Processing and Analysis Methods

#### Expert Scoring

Data processing and statistical analysis of clinicians’ evaluations were executed using RStudio software (version 1.1.35; PBC). Two clinicians, hailing from tier-3 class-A hospitals in China and with substantial contributions to China’s COVID-19 response, independently scored the answers generated by ChatGPT 3.5 and ChatGPT 4.0. Scoring was carried out across 4 predetermined dimensions—accuracy, comprehensiveness, relevance, and clarity—and was benchmarked against the official answers provided by the WHO. Both clinicians were blinded to the source of the answers, ensuring a double-blind evaluation process. Additionally, the sequence of answers for each question was randomized to further minimize bias. Prior to the evaluation, the clinicians consulted an authoritative compendium of COVID-19 questions and answers from the WHO to ensure a comprehensive and accurate understanding of the subject matter. The individual clinical evaluation scores by KG are detailed in Multimedia Appendix 3, and the scores by QL can be found in Multimedia Appendix 4.

We examined the consistency of the scores from the 2 clinicians, calculating the Cronbach α coefficient of the scores for both versions. Furthermore, we performed a descriptive statistical analysis of the average scores of the generated answers across the 4 dimensions and compared them with the official WHO answers. Before conducting hypothesis testing, the distribution of the data across the 4 dimensions, accuracy, comprehensiveness, relevance, and clarity, was considered for both versions of ChatGPT. Given that the Mann-Whitney *U* test does not assume normality of the data distribution, this nonparametric test was directly applied to evaluate the statistically significant differences between the responses generated by ChatGPT 3.5 and 4.0, which is especially appropriate for our data as it does not require the assumption of normality.

#### BERT Scoring

In this study, the BERT model, a pretrained deep learning model renowned for its efficacy in natural language processing tasks, was used to appraise the quality of responses generated by ChatGPT 3.5 and ChatGPT 4.0. The BERT model is adept at identifying intricate semantic patterns in text, thereby generating high-quality text representations [19]. We calculated the cosine similarity between the vector representations of the authoritative responses from the WHO and the responses generated by ChatGPT 3.5 and ChatGPT 4.0. The closer the calculated value is to 1, the higher the semantic congruence between the generated response and the authoritative answer. This method provides a quantitative measure of the quality of the information provided by the AI models in relation to the authoritative source. A detailed comparison of the BERT scores and the responses is presented in Multimedia Appendix 5.

## Results

### Expert Scoring

Using the Mann-Whitney *U* test, we discerned statistically significant disparities across all assessed dimensions, namely, accuracy, comprehensiveness, relevance, and clarity (each with *P*<.001). Notably, ChatGPT 4.0 outperformed ChatGPT 3.5 in every evaluated dimension, corroborating the hypothesis that ChatGPT 4.0 is superior in generating responses that are not only accurate but also comprehensive, relevant, and clear (Table 2).

**Table 2:** Statistical comparison of ChatGPT 3.5 and ChatGPT 4.0 across evaluation dimensions.

Evaluation dimension	Score for ChatGPT 3.5, mean (SD)	Score for ChatGPT 4.0, mean (SD)	Mann-Whitney *U* value	*P* value
Accuracy	3.47 (0.725)	4.15 (0.780)	263,250	<.001
Comprehensiveness	3.89 (0.719)	4.47 (0.641)	283,632	<.001
Relevance	4.09 (0.787)	4.56 (0.600)	328,018	<.001
Clarity	3.49 (0.809)	4.09 (0.698)	294,482	<.001

The consistency of the scores assigned by the 2 experts to the responses generated by both versions of ChatGPT was rigorously evaluated. This evaluation was grounded on the detailed scoring provided in Multimedia Appendices 3 and 4. The Cronbach α coefficients for the scores from ChatGPT 3.5 and 4.0 were .94 and .92, respectively, indicating a high degree of consistency in the evaluations made by the 2 experts. These coefficients, significantly exceeding .9, denote a robust agreement between the experts in their assessment methods. This level of interrater reliability not only confirms the consistency of the expert evaluations but also enhances the validity of our study's conclusions. The values provided above are directly derived and calculated from the detailed scores found in Multimedia Appendices 3 and 4. The high α values, approaching 1, signify a strong consensus in the expert evaluations, reinforcing the reliability and credibility of their assessments of the answers generated by the 2 different versions of ChatGPT.

### BERT Scoring

The average similarity scores between the responses generated by ChatGPT versions 3.5 and 4.0 and the official WHO responses are discussed here. Both versions achieved similarity scores above 0.8. Specifically, ChatGPT 4.0 scored slightly higher with an average BERT score of 0.85 (SD 0.07) compared to ChatGPT 3.5, which scored an average of 0.83 (SD 0.07). This suggests that ChatGPT 4.0 has made improvements in terms of semantic similarity. For a detailed view of the responses generated by both ChatGPT 3.5 and ChatGPT 4.0 for the COVID-19 Q&A, refer to Multimedia Appendix 6.

### Descriptive Analysis

**Figure 1:**
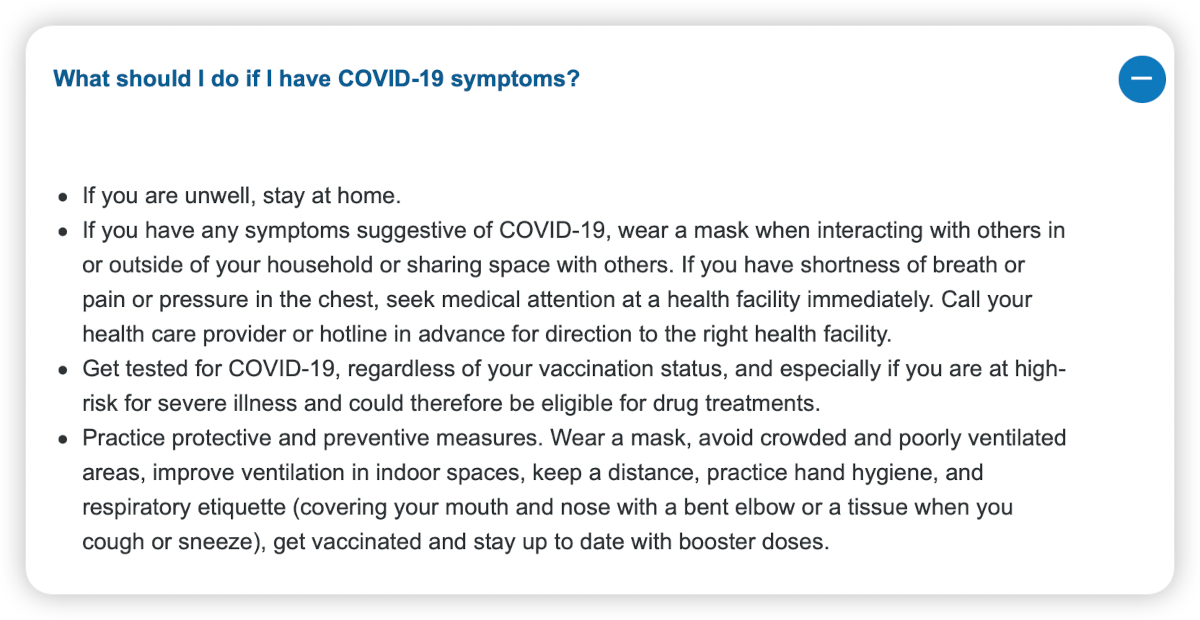
World Health Organization answer—“What should I do if I have COVID-19 symptoms?”.

**Figure 2:**
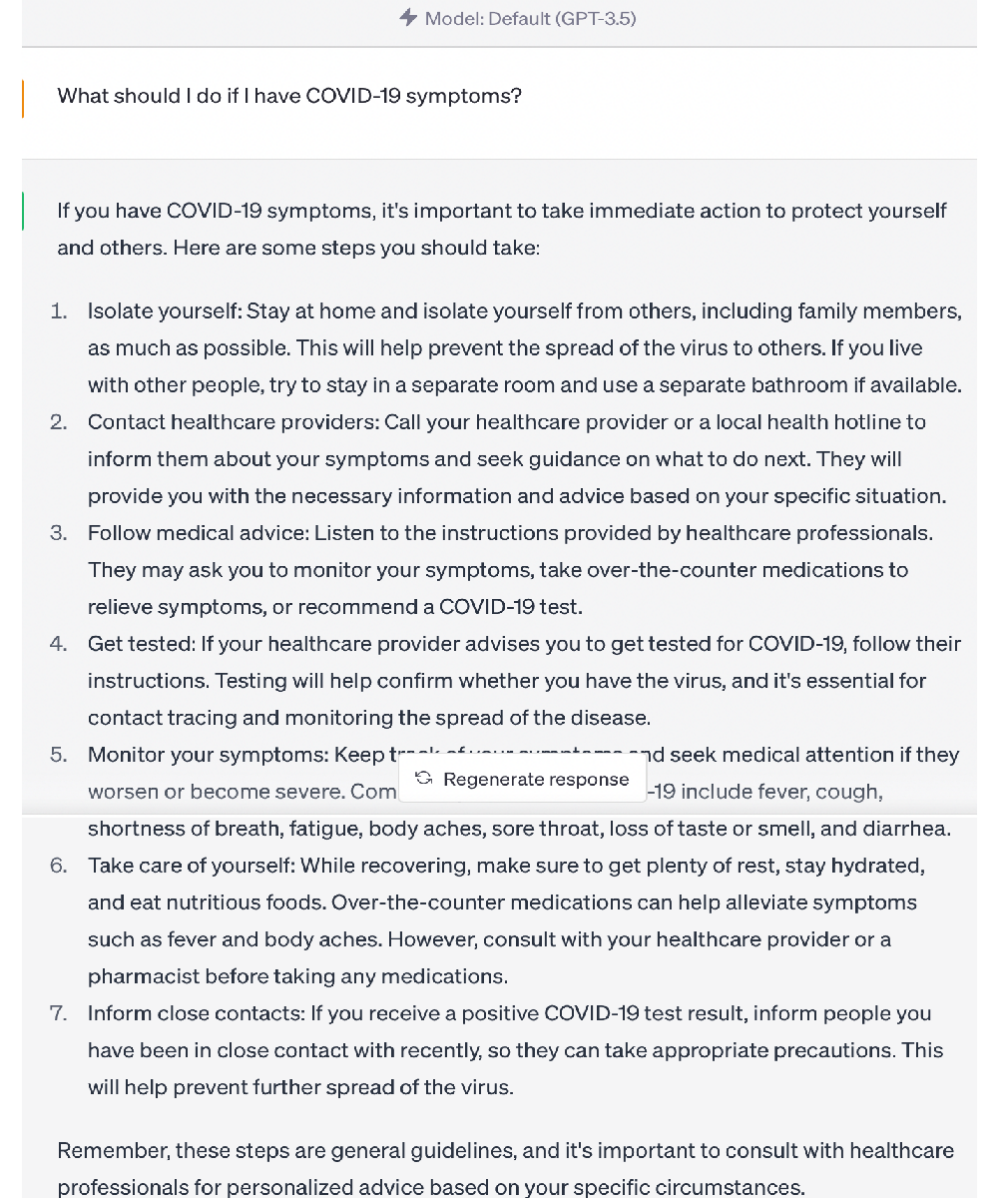
ChatGPT 3.5 answer—“What should I do if I have COVID-19 symptoms?”.

**Figure 3:**
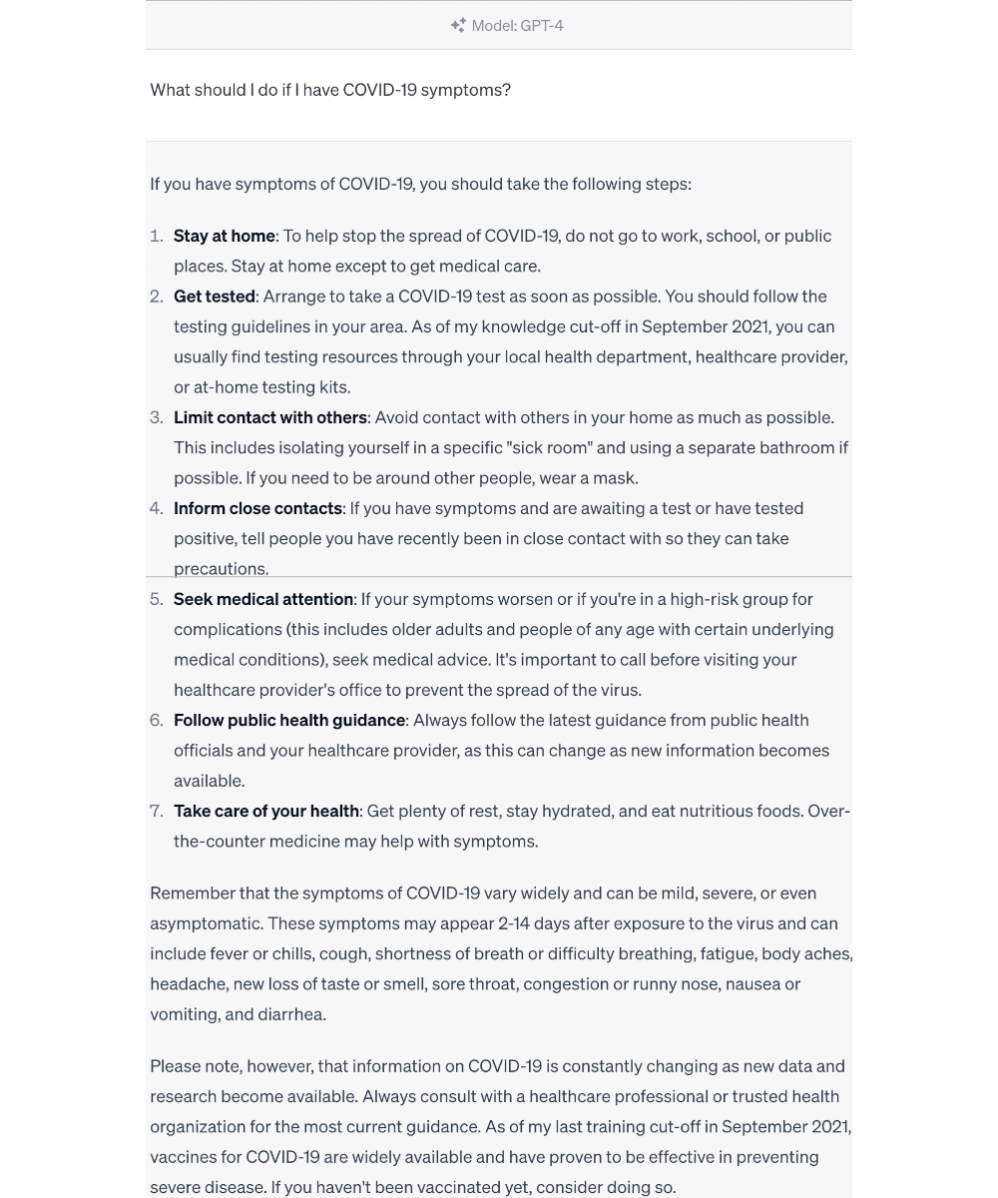
ChatGPT 4.0 answer—“What should I do if I have COVID-19 symptoms?”.

Our analysis revealed that the responses generated by ChatGPT 3.5 and 4.0 to certain questions were on par with the authoritative responses from the WHO, as demonstrated by high clinical expert ratings and BERT scores (Figures 1-3).

**Figure 4:**
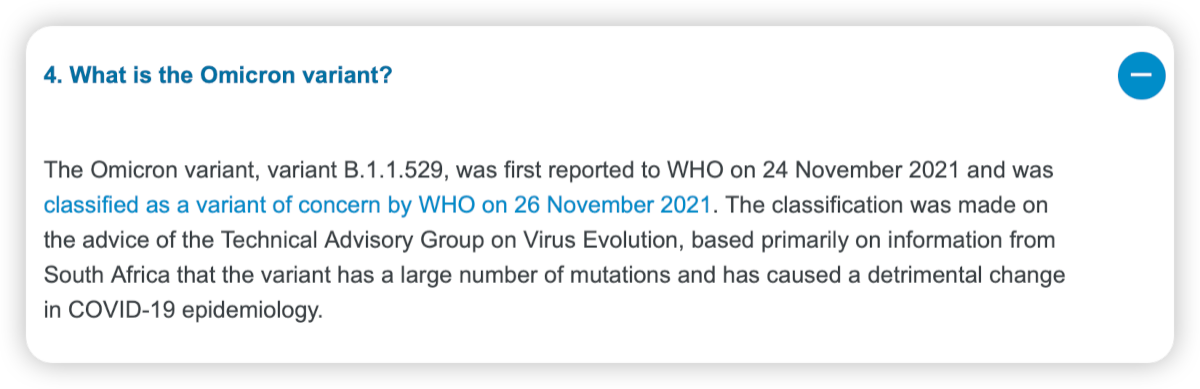
World Health Organization answer—“What is the Omicron variant?”.

**Figure 5:**
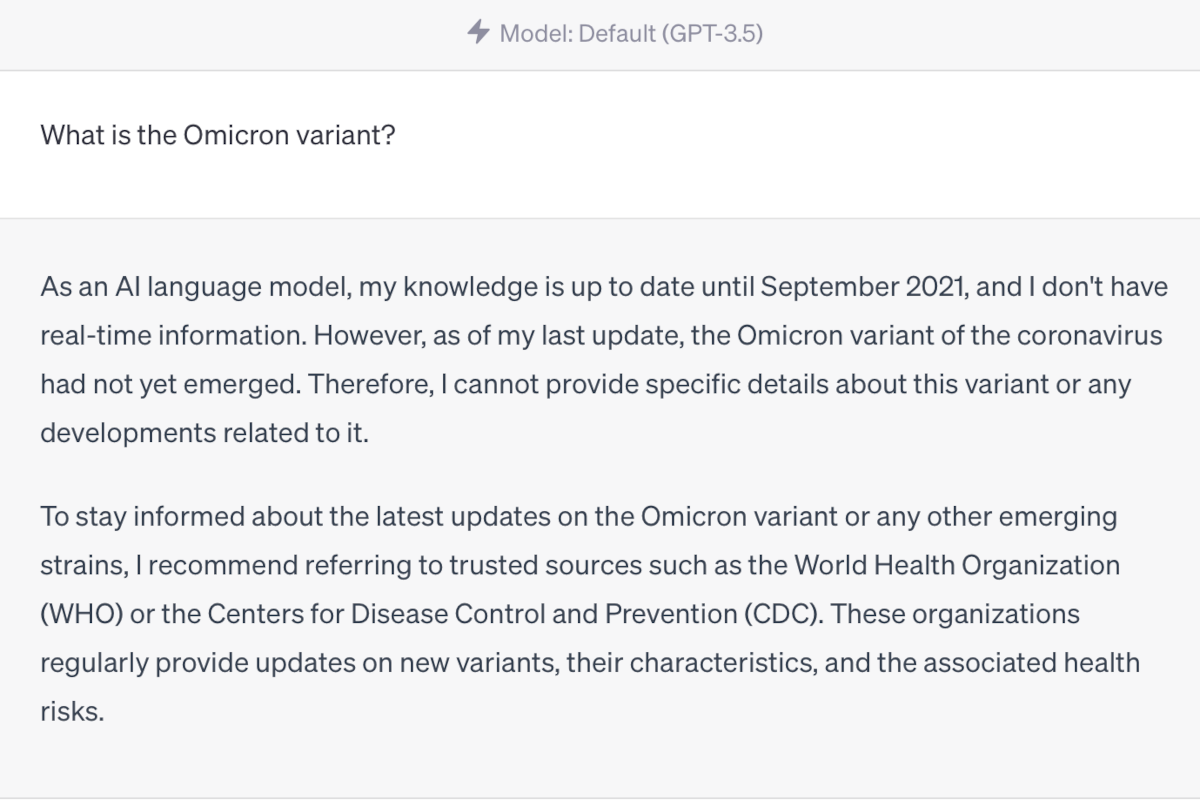
ChatGPT 3.5 answer—“What is the Omicron variant?”.

**Figure 6:**
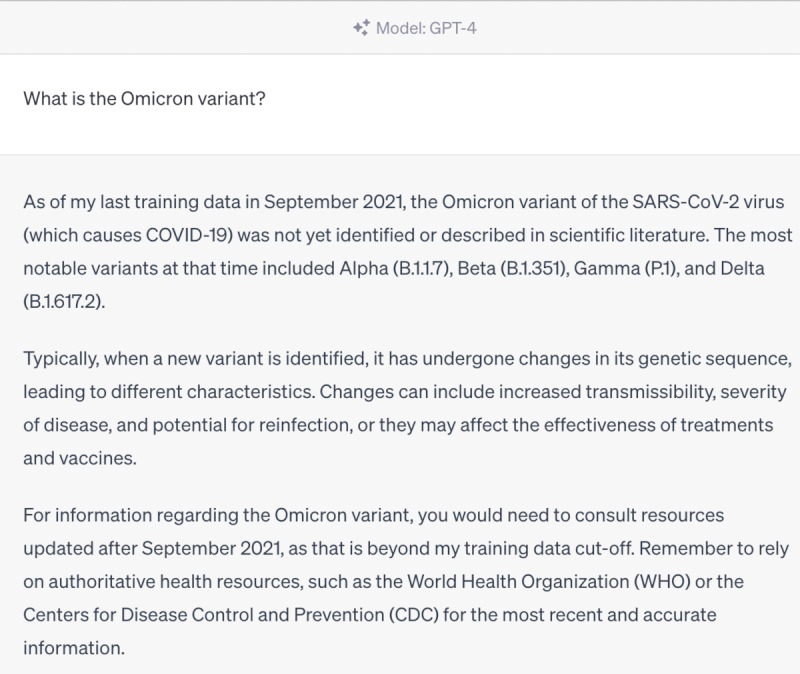
ChatGPT 4.0 answer—“What is the Omicron variant?”.

**Figure 7:**
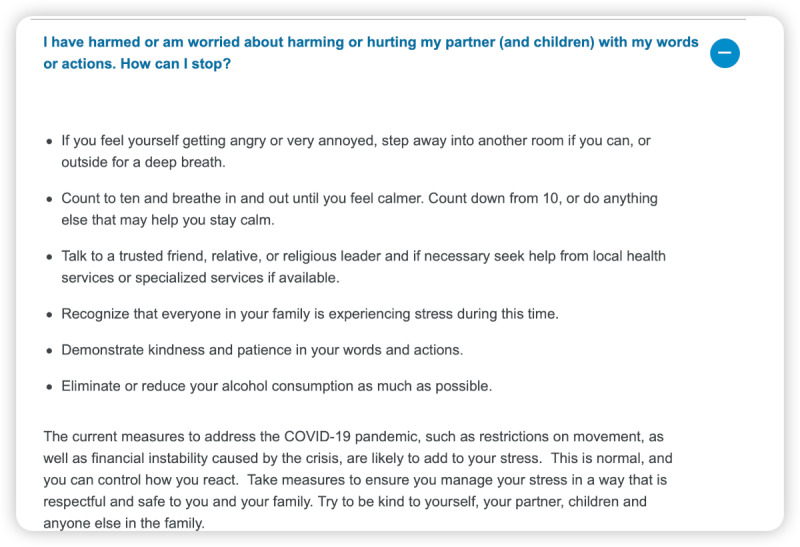
World Health Organization answer—“I have harmed or am worried about harming or hurting my partner (and children) with my words or actions. How can I stop?”.

**Figure 8:**
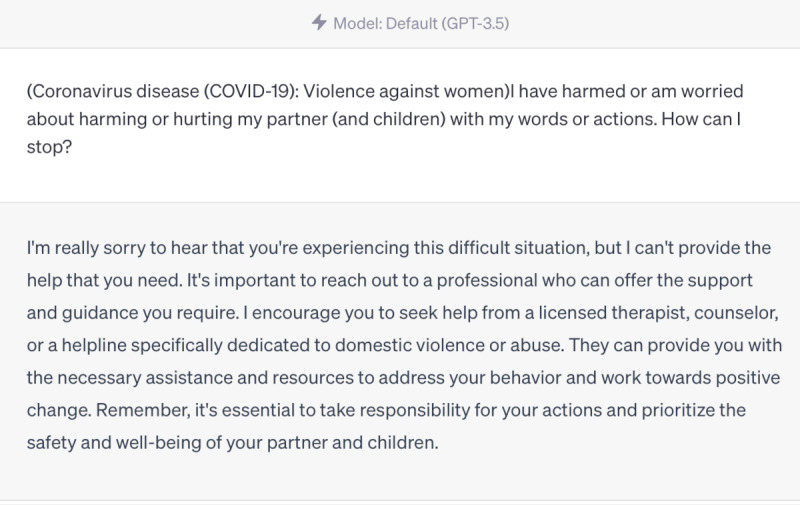
ChatGPT 3.5 answer—“I have harmed or am worried about harming or hurting my partner (and children) with my words or actions. How can I stop?”.

**Figure 9:**
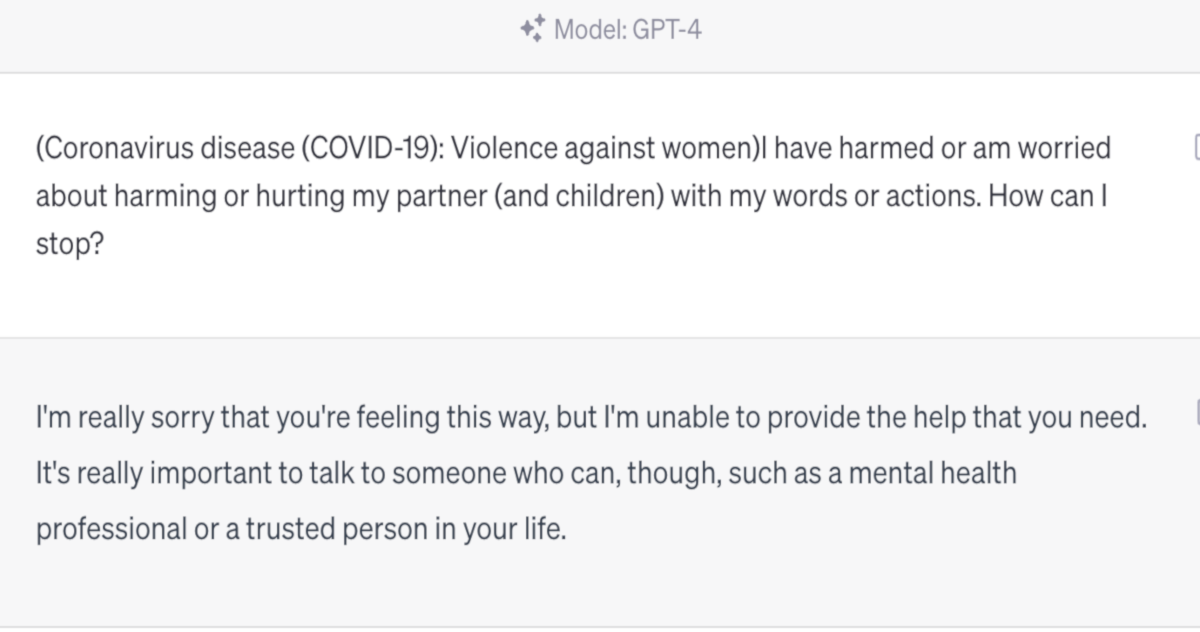
ChatGPT 4.0 answer—“I have harmed or am worried about harming or hurting my partner (and children) with my words or actions. How can I stop?”.

However, we also identified areas where ChatGPT struggled to provide accurate responses. For instance, it was unable to provide information on the Omicron variant, as this is the knowledge that emerged after September 2021, beyond its training data (Figures 4-6). Furthermore, ChatGPT 4.0 performed poorly on topics related to humanities and ethics. For example, it was unable to provide effective assistance in the scenario of women facing domestic violence during the COVID-19 pandemic (Figures 7-9).

**Figure 10:**
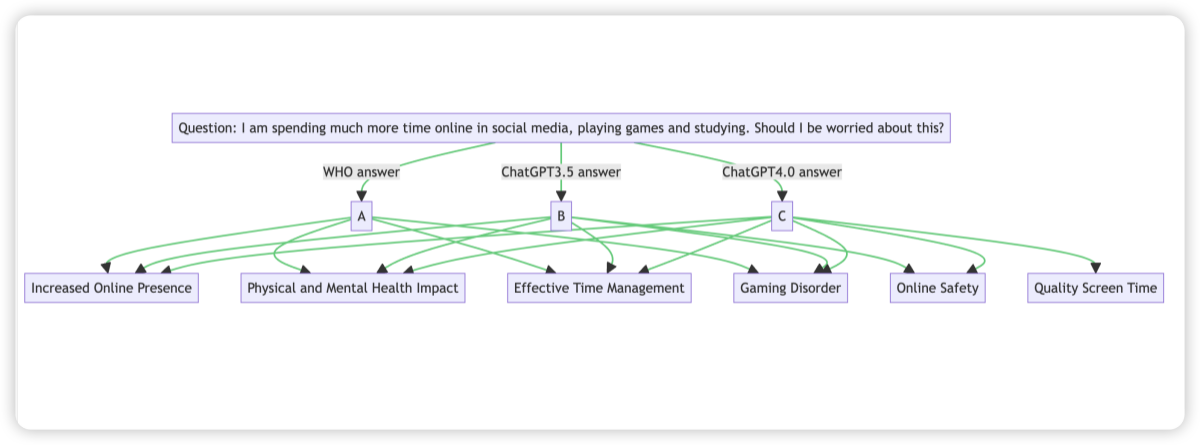
Comparing key points in ChatGPT 3.5 and 4.0 and WHO responses to question 39. WHO: World Health Organization.

A visual comparison of the key points derived from the responses of the WHO, ChatGPT 3.5, and ChatGPT 4.0 to a specific question is provided in Figure 10. This comparison demonstrates the ability of ChatGPT 3.5 and ChatGPT 4.0 to generate reliable and accurate responses, with ChatGPT 4.0 offering more comprehensive and nuanced perspectives.

## Discussion

### Principal Findings

Through the evaluation of COVID-19 information generated by ChatGPT and authoritative information from the WHO, we find that the advantages of ChatGPT in generating COVID-19 information lie in its ability to generate comprehensive and relevant information, but there is still room for improvement in the accuracy and clarity of the information generated [20]. Although the information generated by ChatGPT 4.0 is superior to ChatGPT 3.5 in terms of accuracy, comprehensiveness, relevance, and clarity, there are still limitations; especially, when facing complex ethical situations, it cannot provide specific and effective suggestions. This is significant for understanding and improving the performance of ChatGPT, enhancing its application value in the field of public health, and promoting the cooperation between AI technology and public health institutions. This study also provides reference and inspiration for other epidemic information services, demonstrating the potential and challenges of generative dialog models in handling complex and sensitive information [21-23].

### Comparison to Prior Work

This study is the first to include a complete authoritative official Q&A database on COVID-19 for comparison, in order to assess the quality of the COVID-19 information generated by ChatGPT. A research design combining quantitative and qualitative methods was adopted, and a comprehensive and in-depth analysis of the generated answers was conducted from multiple dimensions of expert scoring and BERT similarity scoring. The performance differences between ChatGPT 3.5 and ChatGPT 4.0 are compared to reflect the evolution speed and direction of the ChatGPT model. This is a dynamic and comparative study that provides a benchmark or reference point for other versions of ChatGPT [24].

### Future Directions

We found that ChatGPT performs excellently in many areas, but it also has the following limitations [25-27]. First, it is time-limited, as it only contains information up until September 2021. Therefore, it cannot explain or answer some new concepts, such as the Omicron variant, which was first reported to the WHO on November 24, 2021, and listed as a variant of concern by the WHO on November 26, 2021. Although ChatGPT 4.0 cannot accurately describe the Omicron variant, it can enumerate all known variants and describe possible mutations, making its answers more comprehensive and relevant than those of ChatGPT 3.5. Second, it does not annotate its sources, which makes immediate verification difficult. Almost all answers given by ChatGPT do not annotate their sources, making it hard to verify the authenticity of data and information. However, in general, the accuracy of answers from ChatGPT 4.0 is higher than that of ChatGPT 3.5. Third, its responses to professional information are somewhat vague, such as those related to the treatment of COVID-19. It can accurately list the types and schemes of drugs used in COVID-19 treatment, but neither ChatGPT 4.0 nor ChatGPT 3.5 can provide standard protocols for drug use and dosage. Therefore, ChatGPT is more suitable for assisting medical workers rather than replacing them. Fourth, it may struggle to handle questions related to ethics [28]. When we asked questions related to ethics, the answers were often vague. For example, ChatGPT 4.0 may suggest that we seek help from a trusted person, but this answer is neither accurate nor comprehensive, and it does not solve the actual problem. We look forward to new versions of ChatGPT that have real-time training data and make greater progress in areas such as information citation, professionalism, and ethics.

Building on the limitations discussed, it is crucial to consider the ethical dimensions that come with the application of AI in public health. These concerns are not merely theoretical but have practical implications for the integrity of health care services and public trust. In addressing these ethical concerns, we emphasize the importance of safeguarding data privacy through robust protections, mitigating misinformation with stringent validation of AI-generated content, and enhancing the ethical reasoning capabilities of AI systems [29,30]. As AI’s role in health care grows, it is imperative that these systems not only provide accurate information but also align with ethical standards to support the integrity of health care delivery.

Compared to traditional search engines, ChatGPT can provide continuous, customized, multichannel, and user-friendly information services. It can help the public obtain and understand authoritative and accurate information in the field of public health, thereby improving their health awareness and behavior, reducing their risk of infection or spread of diseases, relieving their psychological pressure and anxiety, and enhancing their confidence and optimistic attitude [31]. In this study, ChatGPT 4.0 outperformed ChatGPT 3.5 in terms of accuracy, comprehensiveness, relevance, semantic similarity, and information matching, indicating the continuous evolution and optimization of the ChatGPT model. The answers to the COVID-19-related questions from ChatGPT 4.0 have a high consistency with the official answers from WHO, with scores in 4 dimensions exceeding 4, indicating that ChatGPT 4.0 can serve as an effective and relatively reliable information service tool to help the public cope with the global pandemic of COVID-19. Of course, we also look forward to the updates of more advanced versions to improve the accuracy and clarity of generated questions and provide accurate answers to professional questions.

### Strengths and Limitations

Despite the promising results, there are some limitations in this study. First, the evaluation was conducted by only 2 clinicians, whose assessments may be influenced by personal preferences and subjective judgments [32]. They may not fully understand and evaluate the answers generated by ChatGPT, thereby potentially limiting the reliability and validity of expert scoring. To address this, we used Cronbach α as a statistical measure of scoring consistency, which showed a high degree of agreement (α value greater than .9) between the evaluators, indicating minimal bias. Nonetheless, we recognize the value of a broader panel of evaluators. Future studies could benefit from a more diverse group of experts for further validation and will strive to include experts from various medical specialties and geographic locations. Additional statistical methods will also be considered to adjust for individual rater biases, thus enhancing the robustness of our research findings. Second, although our primary use of the BERT model as a scoring tool involves calculating similarity scores to assess the quality of responses compared to authoritative answers, we are aware that this method may not capture all subtle semantic differences [33]. Therefore, we also included expert evaluations as a complement, which are not limited by complex semantics and can assess the quality of responses from additional dimensions. The results consistently show that ChatGPT 4.0 outperforms ChatGPT 3.5 in expert assessments, addressing potential limitations of BERT scoring. Future research will explore the inclusion of a more diverse set of natural language processing models to further enhance our understanding and assessment of the semantic depth of AI-generated content. Finally, the study evaluated the quality of generated answers only from the perspectives of doctor scores and BERT scores, without considering subjective factors such as user satisfaction. This may not fully reflect users’ perception of the quality of generated answers. Although we obtained consistent conclusions in the 2 tests, we hope that more tests based on more epidemic information can help us verify the potential of ChatGPT in providing information on epidemics in the future [34].

### Conclusions

In conclusion, this study offers a comparative analysis of the quality of COVID-19 information generated by ChatGPT 3.5 and ChatGPT 4.0, benchmarked against the authoritative information provided by the WHO. Our findings indicate that ChatGPT 4.0 has surpassed its predecessor, ChatGPT 3.5, in multiple dimensions and exhibits a higher degree of similarity to the WHO’s official information. This conclusion is further corroborated by our tests using the BERT model.

Nevertheless, there remains a significant gap between the accuracy and clarity of the responses generated by ChatGPT 4.0 and the WHO’s official information, indicating areas for potential enhancement. Conversely, in terms of comprehensiveness and relevance, the responses generated by ChatGPT 4.0 demonstrate commendable performance, occasionally even exceeding the WHO’s official information.

This research contributes to our understanding and potential improvement of ChatGPT’s performance, thereby enhancing its applicability in the realm of public health and fostering collaboration between large language models and public health organizations. As an innovative, systematic, in-depth, dynamic, and comparative study, our research provides valuable insights and serves as a reference for other epidemic information services and generative dialog models.

## Appendix

Multimedia Appendix 1 World Health Organization's question and answer collection on COVID-19.

Multimedia Appendix 2 Complete list of prompts used for ChatGPT 3.5 and ChatGPT 4.0 in COVID-19 question and answer evaluation.

Multimedia Appendix 3 Clinical evaluation scores by KG.

Multimedia Appendix 4 Clinical evaluation scores by QL.

Multimedia Appendix 5 Comparison and bidirectional encoder representations from transformers (BERT) scores of World Health Organization answers and ChatGPT 3.5 and 4.0 versions.

Multimedia Appendix 6 Generated responses by ChatGPT 3.5 and ChatGPT 4.0 for COVID-19 question and answer.

## Abbreviations

AI: artificial intelligence
BERT: Bidirectional Encoder Representations from Transformers
Q&A: question and answer
WHO: World Health Organization

## Data Availability

All data generated or analyzed during this study are included in this published paper and its supplementary information files (Multimedia Appendices 1-6).
