# Supplementary material for: Potential and Limitations of ChatGPT 3.5 and 4.0 as a Source of COVID-19 Information: Comprehensive Comparative Analysis of Generative and Authoritative Information
Source: J Med Internet Res. 2023 Dec 14;25:e49771. doi: 10.2196/49771 (PMC10755661; doi:10.2196/49771)
Supplement: Multimedia Appendix 1 [file jmir_v25i1e49771_app1.doc]

**Q&As on COVID-19 and related health topics from WHO**

#### [Coronavirus disease (COVID-19)](https://www.who.int/emergencies/diseases/novel-coronavirus-2019/question-and-answers-hub/q-a-detail/coronavirus-disease-covid-19)

1、 [**What is COVID-19?**](https://www.who.int/emergencies/diseases/novel-coronavirus-2019/question-and-answers-hub/q-a-detail/coronavirus-disease-covid-19)

COVID-19 is the disease caused by a coronavirus called SARS-CoV-2.  WHO first learned of this new virus on 31 December 2019, following a report of a cluster of cases of so-called viral pneumonia in Wuhan, People’s Republic of China.

2、**[What are the symptoms of COVID-19?](https://www.who.int/emergencies/diseases/novel-coronavirus-2019/question-and-answers-hub/q-a-detail/coronavirus-disease-covid-19)**

The most common symptoms of COVID-19 are

fever

chills

sore throat.

Other symptoms that are less common and may affect some patients include:

muscle aches

severe fatigue or tiredness

runny or blocked nose, or sneezing

headache

sore eyes

dizziness

new and persistent cough

tight chest or chest pain

shortness of breath

hoarse voice

heavy arms/legs

numbness/tingling

nausea, vomiting, abdominal pain/ belly ache, or diarrhoea

appetite loss

loss or change of sense of taste or smell

difficulty sleeping.

Symptoms of severe COVID‐19 disease which need immediate medical attention include:

difficulty in breathing, especially at rest, or unable to speak in sentences

confusion

drowsiness or loss of consciousness

persistent pain or pressure in the chest

skin being cold or clammy, or turning pale or a bluish colour

loss of speech or movement.

If possible, call your health care provider, hotline or health facility first, so you can be directed to the right clinic.

People who have pre-existing health problems are at higher risk when they have COVID-19; they should seek medical help early if worried about their condition. These include, but are not limited to: those taking immunosuppressive medication; those with chronic heart, lung, liver or rheumatological problems; those with HIV, diabetes, cancer or dementia.

[3、](https://www.who.int/emergencies/diseases/novel-coronavirus-2019/question-and-answers-hub/q-a-detail/coronavirus-disease-covid-19)**[What happens to people who get COVID-19?](https://www.who.int/emergencies/diseases/novel-coronavirus-2019/question-and-answers-hub/q-a-detail/coronavirus-disease-covid-19)**

As testing rates fall, it is more difficult to know how many people have COVID-19 and do not seek any treatment. At the start of the pandemic, 15% of people were thought to become seriously unwell and require hospital treatment and oxygen. More recent estimates suggest that hospitalization is required in around 3% of people with COVID-19. This is partly due to immunization, partly due to changes in the virus (especially the Omicron variants), and partly due to the availability of targeted medical treatments.

Most people make a full recovery without needing hospital treatment. For those with COVID-19 who are at high risk of severe illness (see question below), WHO has made recommendations on which drug treatments are effective in improving outcomes and preventing hospital admissions.

It is also important to be vigilant in recognizing people with severe disease and those needing hospital treatment so that they are treated early. The consequences of severe COVID-19 include death, respiratory failure, sepsis, thromboembolism (blood clots), and multiorgan failure, including injury of the heart, liver or kidneys.

In rare situations, children can develop a severe inflammatory syndrome a few weeks after infection.

4、**[Who is most at risk of severe illness from COVID-19?](https://www.who.int/emergencies/diseases/novel-coronavirus-2019/question-and-answers-hub/q-a-detail/coronavirus-disease-covid-19)**

People aged 60 years and over, and those with underlying medical problems like high blood pressure, diabetes, other chronic health problems (for example those affecting the heart, lungs, kidneys, and brain), low immune function / immunosuppression (including HIV), obesity, cancer, and unvaccinated people are most at risk of severe illness.

However, anyone at any age can get sick with COVID-19 and become seriously ill or die.

5、**[Are there long-term effects of COVID-19?](https://www.who.int/emergencies/diseases/novel-coronavirus-2019/question-and-answers-hub/q-a-detail/coronavirus-disease-covid-19)**

Some people who have had COVID-19, whether they have needed hospitalization or not, continue to experience symptoms, including fatigue, respiratory and neurological symptoms. These long-term effects are called post COVID-19 condition (also called long COVID). For further information on [long COVID](https://www.who.int/news-room/questions-and-answers/item/coronavirus-disease-(covid-19)-post-covid-19-condition), see [post COVID-19 condition](https://www.who.int/teams/health-care-readiness/post-covid-19-condition) and [questions and answers](https://www.who.int/news-room/questions-and-answers/item/coronavirus-disease-(covid-19)-post-covid-19-condition).

6、**[How can we protect others and ourselves if we don't know who is infected?](https://www.who.int/emergencies/diseases/novel-coronavirus-2019/question-and-answers-hub/q-a-detail/coronavirus-disease-covid-19)**

You can protect yourself and others from COVID-19 by following preventive measures, such as keeping a distance, wearing a mask in crowded and poorly ventilated spaces, practicing hand hygiene, respiratory etiquette (covering your mouth and nose with a bent elbow or a tissue when you cough or sneeze), getting vaccinated and staying up to date with booster doses.

 Check local advice where you live and work.

Read our [public advice page](https://www.who.int/emergencies/diseases/novel-coronavirus-2019/advice-for-public) for more information.

7、**[When should I get a test for COVID-19?](https://www.who.int/emergencies/diseases/novel-coronavirus-2019/question-and-answers-hub/q-a-detail/coronavirus-disease-covid-19)**

Anyone with symptoms such as acute onset of fever and cough should be tested, wherever possible, to ensure that they receive appropriate clinical care. People who do not have symptoms but have had close contact with someone who is, or may be, infected may also consider testing. Contact your local health guidelines and follow their guidance.

While a person is waiting for test results, they should preferably wear a mask when interacting with others in or outside of their household or sharing space with others. Where testing capacity is limited, tests should first be done for those at higher risk of infection, such as health workers, and those at higher risk of severe illness such as older people, especially those living in seniors’ residences or long-term care facilities.

Individuals with signs and symptoms suggestive of COVID-19 or those who test positive for the virus should wear a mask when interacting with others in or outside of one’s household or sharing space with others.

8、**[What test should I get to see if I have COVID-19?](https://www.who.int/emergencies/diseases/novel-coronavirus-2019/question-and-answers-hub/q-a-detail/coronavirus-disease-covid-19)**

There are two main types of tests that can confirm whether you are infected with SARS-CoV-2, the virus that causes COVID-19. Molecular tests, such as polymerase chain reaction (PCR), are the most accurate tests for diagnosing SARS-CoV-2 infection. Molecular tests detect virus in the sample by amplifying viral genetic material to detectable levels. Rapid antigen tests (sometimes known as rapid diagnostic tests or RDTs) detect viral proteins (known as antigens). RDTs are a simpler and faster option than molecular tests and are available for testing by trained operators or by the individual themselves (sometimes called self-tests). They perform best when there is more virus circulating in the community and when sampled from an individual during the time they are most infectious, generally within the first 5–7 days following symptom onset. Samples for both types of tests are collected from the nose and/or throat with a swab.

[Learn more about what kind of COVID-19 tests are available](https://www.who.int/emergencies/diseases/novel-coronavirus-2019/media-resources/science-in-5/episode-14---covid-19---tests)

[Why testing for SARS-CoV-2 is important](https://www.who.int/multi-media/details/why-testing-is-important)

[Use of Ag-RDTs](https://www.who.int/multi-media/details/use-of-antigen-detection-rapid-diagnostic-testing)

[What you need to know about self-testing](https://www.who.int/multi-media/details/what-you-need-to-know-about-covid-19-self-testing)

9、**[I want to find out if I had COVID-19 in the past, what test could I take?](https://www.who.int/emergencies/diseases/novel-coronavirus-2019/question-and-answers-hub/q-a-detail/coronavirus-disease-covid-19)**

Antibody tests can tell us whether someone has had an infection in the past, even if they have not had symptoms. Also known as serological tests, these tests detect antibodies produced in response to an infection or vaccination. In most people, antibodies start to develop after days to weeks and can indicate if a person has had past infection or has been vaccinated. Antibody tests cannot be used to diagnose SARS-CoV-2 infection in the early stages of infection or disease but can indicate whether or not someone has had the disease in the past. If you have been vaccinated, many antibody tests are not able to distinguish between whether you were previously infected or have been vaccinated (or both), so in such situations you will test positive in either case.

10、**[What is the difference between isolation and quarantine?](https://www.who.int/emergencies/diseases/novel-coronavirus-2019/question-and-answers-hub/q-a-detail/coronavirus-disease-covid-19)**

Both isolation and quarantine are methods of preventing the spread of COVID-19.

**Quarantine**is used for certain persons who are a contact of someone infected with SARS-CoV-2, the virus that causes COVID-19, whether the infected person has symptoms or not. Quarantine means that you remain separated from others because you have been exposed to the virus and you may be infected and can take place in a designated facility or at home. For COVID-19, this means staying in the facility or at home for several days.

**Isolation** is used for people with COVID-19 symptoms or who have tested positive for the virus. Being in isolation means being separated from other people, ideally in a medical facility where you can receive clinical care. If isolation in a medical facility is not possible and you are not in a high-risk group for developing severe disease, isolation can take place at home. If you have symptoms, you should remain in isolation for at least 10 days. If you are infected and do not develop symptoms, you should remain in isolation for 5 days from the time you test positive. You can be discharged from isolation early if you test negative on a rapid antigen test.

11、**[What should I do if I have been exposed to someone who has COVID-19?](https://www.who.int/emergencies/diseases/novel-coronavirus-2019/question-and-answers-hub/q-a-detail/coronavirus-disease-covid-19)**

If you have been exposed to someone with COVID-19, you may become infected, even if you feel well.

After exposure to someone who has COVID-19, do the following:

- Call your healthcare provider to get tested or test yourself.
- Stay home if you feel unwell.
- Wear a mask when interacting with others in or outside of your household or sharing space with others.
- Clean your hands regularly.
- Practice respiratory etiquette: cover your mouth and nose with your bent elbow or a tissue when you cough or sneeze.
- Avoid crowded, enclosed or poorly ventilated spaces.
- Get vaccinated and stay up to date with booster doses.

12、**[How long does it take to develop symptoms?](https://www.who.int/emergencies/diseases/novel-coronavirus-2019/question-and-answers-hub/q-a-detail/coronavirus-disease-covid-19)**

- The time from exposure to COVID-19 to the moment when symptoms begin is, on average, 5–6 days and can range from 1–14 days. This is why people who have been exposed to the virus are advised to remain at home and stay away from others in order to prevent the spread of the virus.

13、**[Is there a vaccine for COVID-19?](https://www.who.int/emergencies/diseases/novel-coronavirus-2019/question-and-answers-hub/q-a-detail/coronavirus-disease-covid-19)**

- Yes. There are several COVID-19 vaccines validated for use by WHO (given Emergency Use Listing) and from other stringent national regulatory agencies (SRAs). The first mass vaccination programme started in early December 2020 and the number of vaccination doses administered is updated on a regular basis [here](https://covid19.who.int/). For more information on vaccine for COVID-19, see the vaccine questions and answers: [Coronavirus disease (COVID-19): Vaccines (who.int)](https://www.who.int/emergencies/diseases/novel-coronavirus-2019/question-and-answers-hub/q-a-detail/coronavirus-disease-(covid-19)-vaccines)

14、**[What should I do if I have COVID-19 symptoms?](https://www.who.int/emergencies/diseases/novel-coronavirus-2019/question-and-answers-hub/q-a-detail/coronavirus-disease-covid-19)**

- If you are unwell, stay at home.
- If you have any symptoms suggestive of COVID-19, wear a mask when interacting with others in or outside of your household or sharing space with others. If you have shortness of breath or pain or pressure in the chest, seek medical attention at a health facility immediately. Call your health care provider or hotline in advance for direction to the right health facility.
- Get tested for COVID-19, regardless of your vaccination status, and especially if you are at high-risk for severe illness and could therefore be eligible for drug treatments.
- Practice protective and preventive measures. Wear a mask, avoid crowded and poorly ventilated areas, improve ventilation in indoor spaces, keep a distance, practice hand hygiene, and respiratory etiquette (covering your mouth and nose with a bent elbow or a tissue when you cough or sneeze), get vaccinated and stay up to date with booster doses.

15、**[Are there treatments for COVID-19?](https://www.who.int/emergencies/diseases/novel-coronavirus-2019/question-and-answers-hub/q-a-detail/coronavirus-disease-covid-19)**

There has been huge progress in developing treatments for patients with COVID-19. Treatments for COVID-19 should be decided on an individual basis between the person and the healthcare professional looking after them. The choice will depend on the severity of disease and the risk of disease worsening (including the person’s age and whether they have any health problems). WHO maintains a list of recommended treatments, together with the evidence for each, available at <https://app.magicapp.org/#/guideline/nBkO1E>.

These currently include:

- for non-severe COVID-19: nirmatrelvir-ritonavir; molnupiravir; remdesivir
- for severe COVID-19: corticosteroids (including dexamethasone); IL-6 receptor blockers (tocilizumab or sarilumab); baricitinib; remdesivir.

Apart from these medications, amongst the most commonly and globally used, and important treatments is oxygen for severely ill patients. WHO leads work which aims to improve global capacity and access to oxygen production, distribution and supply to patients.

For corticosteroids, further specific information can be found in the Corticosteroids, including dexamethasone [questions and answers](https://www.who.int/emergencies/diseases/novel-coronavirus-2019/question-and-answers-hub/q-a-detail/coronavirus-disease-covid-19-dexamethasone).

16、**[Are antibiotics effective in preventing or treating COVID-19?](https://www.who.int/emergencies/diseases/novel-coronavirus-2019/question-and-answers-hub/q-a-detail/coronavirus-disease-covid-19)**

Antibiotics do not work against viruses; they only work on bacterial infections. COVID-19 is caused by a virus, so antibiotics do not work. Antibiotics should not be used as a means of prevention or treatment of COVID-19.

In hospitals, physicians will sometimes use antibiotics to treat secondary bacterial infections, which can be a complication of COVID-19 in severely ill patients. They should only be used as directed by a physician to treat a bacterial infection.

#### [Coronavirus disease (COVID-19) and people living with HIV](https://www.who.int/emergencies/diseases/novel-coronavirus-2019/question-and-answers-hub/q-a-detail/coronavirus-disease-(covid-19)-covid-19-and-people-living-with-hiv)

17、**[Are people living with HIV at increased risk of being infected with SARS-CoV-2 or develop more severe COVID-19?](https://www.who.int/emergencies/diseases/novel-coronavirus-2019/question-and-answers-hub/q-a-detail/coronavirus-disease-(covid-19)-covid-19-and-people-living-with-hiv)**

People living with HIV (PLHIV) who have not achieved viral suppression through antiretroviral treatment (ART) may have a compromised immune system that leaves them vulnerable to opportunistic infections and further HIV disease progression. There is no clinical evidence that PLHIV have a higher risk of infection with SARS-CoV-2 when compared with HIV-negative people.

There are several small studies conducted early in the pandemic that describe the clinical presentation of COVID-19 in PLHIV, indicating that the clinical presentation of COVID-19 is similar in people with and without HIV, particularly if they are on ART and have achieved HIV viral suppression However, global data compiled by WHO from almost 350 000 patients in 38 countries indicate that PLHIV are at increased risk for development of severe illness and death due to COVID-19. This analysis found that the risk of developing severe fatal COVID-19 was 38% greater in this population when compared to people without HIV infection . PLHIV frequently face adverse social determinants of health and structural factors that may lead to higher SARS-CoV-2 exposure. They also have a high prevalence of some comorbidities associated with poorer COVID-19 outcomes, such as cardiovascular disease, diabetes, chronic respiratory disease and hypertension. Additionally, lower CD4 T-cell counts are associated with advanced HIV disease and several epidemiological studies are suggesting that this HIV subpopulation are at greater risk for hospitalization due to COVID-19 and mortality.

Taken all these considerations together, there is no clear evidence of a higher risk for PLHIV to be infected with SARS-COV-2 when compared with the general population. However, those with advanced HIV disease, those with low CD4 T-cell counts and high HIV viral load and those who are not taking ART have an increased risk of COVID-19 complications in general.

18、**[Should the treatment or prevention of COVID-19 be different in people living with HIV?](https://www.who.int/emergencies/diseases/novel-coronavirus-2019/question-and-answers-hub/q-a-detail/coronavirus-disease-(covid-19)-covid-19-and-people-living-with-hiv)**

Current recommendations for COVID-19 clinical management in people living with HIV generally do not differ from those of the general population, including treatment and prevention measures.

PLHIV are as eligible to receive anti-SARS-CoV-2 therapies as people without HIV infection and those individuals with advanced HIV disease should be considered as a high priority.

When starting treatment for COVID-19 in patients with HIV, clinicians should pay careful attention to potential interactions between drugs and overlapping toxicities among COVID-19 treatments, antiretrovirals (ARV), antimicrobial therapies, and other medications.

PLHIV who develop COVID-19 should continue their ARV regimen, opportunistic infection treatment and prophylaxis whenever possible. The ARV regimen should not be switched or adjusted (i.e., by adding ARV drugs to the regimen) for the purpose of preventing or treating SARS-CoV-2 infection.

Individuals who know their HIV status are advised to take the same COVID-19 precautions as the general population (e.g., wash hands often, practice cough hygiene, avoid touching your face, social distancing, seek medical care if symptomatic, self-isolation if in contact with someone with COVID-19 and other actions recommended in the government’s response). PLHIV who are taking ARV drugs should ensure that they have at least 30 days of ARVs, if not a 3- to 6-month supply.

It is also an important opportunity to ensure that all PLHIV who are not yet on antiretroviral treatment begin to do so.  People who feel they may have been at HIV risk are advised to seek testing to protect against HIV disease progression and complications from any other health issues.

19、c [**an antiretrovirals be used to treat COVID-19 or prevent SARS-CoV-2 infection?**](https://www.who.int/emergencies/diseases/novel-coronavirus-2019/question-and-answers-hub/q-a-detail/coronavirus-disease-(covid-19)-covid-19-and-people-living-with-hiv)

No ARVs are currently recommended by WHO to treat or prevent COVID-19. Initial in vitro, animal and small human observational studies conducted during previous coronavirus outbreaks (SARS and MERS) and in the early phases of the COVID-19 pandemic have suggested that some ARVs, such as lopinavir/ritonavir (LPV/r), could help against SARS-CoV-2 and should be repurposed for treatment and prevention of COVID-19. This hypothesis was subsequently assessed in further observational studies and in large randomized controlled trials, but no clinical benefit or impact on disease severity or mortality were demonstrated *(2)*.

Several observational studies also indicated that use of tenofovir disoproxil fumarate (TDF) and tenofovir alafenamide (TAF) could be associated with lower risk of acquiring SARS-CoV-2 and develop severe COVID-19. However, these studies had limitations and may have been influenced by other health issues in the participants.. Subsequent larger observational studies and clinical trials have not found a relationship between TDF or TAF and risk of SARS-CoV-2 acquisition or severe outcomes.

While the evidence of benefit of using ARVs to treat coronavirus infections was not demonstrated, serious side effects from these drugs were not rare. The routine use of LPV/r and TDF or TAF as part of treatment regimens for HIV can be associated with several side effects of moderate severity.

Therefore, at this time there is no evidence to suggest that any particular ARV agent improves or worsens COVID-19 clinical outcomes in PLHIV, or can be used for prevention of SARS-CoV-2 infection. WHO recommends maintaining the standard approaches to initiation and continuation of ART in PLHIV, with a focus on reducing HIV-associated immune compromise and achievement of virologic suppression. The same principles are applicable for use of [pre-exposure prophylaxis (PreP)](https://www.who.int/teams/global-hiv-hepatitis-and-stis-programmes/hiv/prevention/pre-exposure-prophylaxis) or post-exposure prophylaxis (PEP) regimens for HIV prevention.

20、**[Are COVID-19 vaccines safe for people living with HIV?](https://www.who.int/emergencies/diseases/novel-coronavirus-2019/question-and-answers-hub/q-a-detail/coronavirus-disease-(covid-19)-covid-19-and-people-living-with-hiv)**

Many of the initial clinical studies with COVID-19 vaccines have included a small number of people living with HIV (PLHIV) in their trials. Despite limited data, available information from those studies suggests that [current WHO recommended COVID-19 vaccines](https://www.who.int/emergencies/diseases/novel-coronavirus-2019/covid-19-vaccines/advice)are safe for PLHIV. The currently available COVID-19 vaccine products are not live vaccines but include modified viral proteins or genetic material from SARS-CoV-2 which cannot replicate or cause changes to human genes. More recent observational studies have further evaluated the safety of COVID-19 vaccines in this population and didn’t find any evidence of a higher rate of unusual side effects after vaccination when compared with general population. In addition, no pharmacological interactions have been reported between COVID-19 vaccines and antiretroviral medications (ARV) which PLHIV should continue to take after vaccination to maintain health.

Recently, a debate in the scientific community has led to broader concerns about a potential association observed more than a decade ago between adenovirus vector-based vaccines and an increased risk of acquiring HIV infection among men who received this type of vaccine. This unexpected finding was detected in two HIV vaccine trials that used adenovirus type 5 (Ad5) vector containing products *(3, 4)*. The reason for this observed increase in the HIV acquisition risk remains uncertain, although several hypothetical mechanisms have suggested a possible interference in the HIV specific vaccine response or in the CD4 T-cell susceptibility to HIV infection induced by pre-existing adenovirus- associated immunity in combination with other factors.  However, a third study also using Ad5 vector-based HIV vaccine has not reported this finding *(5)*.

Despite these potential concerns, it is important to highlight that the benefits of all authorized COVID-19 vaccines in a pandemic context largely outweigh this potential risk. The theoretical concern on increased risk of HIV acquisition is confined to a specific Ad5 vectored HIV vaccine and should not be extended to other vaccine platforms. However, as several COVID-19 vaccines are using human and animal adenovirus vector platforms, specific studies are encouraged to better assess the safety of Ad5 vectored COVID-19 vaccines in sub-populations at high risk of HIV acquisition. An extended follow-up from a Phase III study of an Ad5 vectored COVID-19 vaccine is currently evaluating HIV seroconversion rates in the participants, and interim results are expected in the end of 2022.

Global data have shown that PLHIV have a 38% greater risk of developing severe or fatal COVID-19 compared to people without HIV infection. For this reason, it is important to prioritize PLHIV to receive the COVID-19 vaccine. People with stable HIV disease have been now included in several COVID-19 vaccine trials, and specific cohort studies on COVID-19 vaccines with PLHIV has been recently rolled out. Therefore, more information on this topic specific to PLHIV is expected in the future. WHO will continue to monitor the situation as new data become available and [SAGE recommendations](https://www.who.int/groups/strategic-advisory-group-of-experts-on-immunization)will be updated accordingly.

21、**[Do COVID-19 vaccines provide protection for people living with HIV?](https://www.who.int/emergencies/diseases/novel-coronavirus-2019/question-and-answers-hub/q-a-detail/coronavirus-disease-(covid-19)-covid-19-and-people-living-with-hiv)**

Current available studies on the efficacy of COVID-19 vaccines in PLHIV with high CD4 T-cell counts and using ART, indicate that most of these individuals produce antibody levels and cellular immune response in the same range observed in people without HIV infection, suggesting that current recommended COVID-19 vaccines are safe and protective in this population. However, there is data in these studies from individuals with low CD4 T-cell counts (i.e., below 200 cells/mm3) or without HIV viral suppression that show a reduced immune response to the COVID-19 vaccine. Furthermore, the antibody responses after vaccination may dwindle faster than people with higher CD4 T-cell counts.

This reduced immune response to COVID-19 vaccines has also been reported in individuals with other causes of severe immunodeficiency (e.g., solid and hematologic malignancies, people with organ transplants and in hemodialysis). Suboptimal immune response against certain vaccine preventable diseases has also been demonstrated in people with advanced HIV disease and/or lower CD4 T-cell counts.

Higher rates of SARS-CoV-2 infection after primary vaccination (i.e., breakthrough infections) among PLHIV when compared with people without HIV have been described. A population-level analysis conducted in immunocompromised patients indicated a 33% higher incidence rate of breakthrough SARS-CoV-2 infections among PLHIV, albeit without accounting for HIV-specific factors *(6)*.

Additionally, a large HIV cohort study with more than 100 000 individuals vaccinated and with good HIV control showed a low overall (3.8%) but 28% higher risk of breakthrough infection without association with HIV viral load or CD4 T-cell counts. The breakthrough rate was also higher in people with vs without HIV (55 cases per 1000 person-years vs 43 cases per 1000 person-years) *(7)* .

These higher rates and risk of breakthrough infections observed in PLHIV when compared with the general population should not be a reason to postpone or to avoid COVID-19 vaccines in these patients, who have a higher risk of COVID-19 complications and death. It supports the recommendation for the use of additional primary doses in this population as in other immunocompromised groups.

Furthermore, national COVID-19 immunization programmes should not exclude people from key and vulnerable populations at risk of HIV, who frequently have limited access to health services. WHO will continue to monitor emerging evidence and will provide timely guidance updates.

22、**[Should people living with HIV get vaccines early in the roll out?](https://www.who.int/emergencies/diseases/novel-coronavirus-2019/question-and-answers-hub/q-a-detail/coronavirus-disease-(covid-19)-covid-19-and-people-living-with-hiv)**

WHO currently recommends that PLHIV should be included as a priority population for COVID-19 immunization. It has been supported by [growing evidence](https://www.who.int/publications-detail-redirect/WHO-2019-nCoV-vaccines-SAGE_recommendation-immunocompromised-persons)suggesting that PLHIV, particularly those with advanced HIV disease, lower CD4 T-cell count, or non-suppressed viral load appear to be at increased risk of poor outcomes and death due to COVID-19 when compared with people without HIV infection. Furthermore, many PLHIV have 1 or more chronic comorbidities that may put them at increased risk of severe COVID-19 or death, independent from their HIV disease or immune status. Therefore, PLHIV, particularly those with co-morbidities (such chronic obstructive pulmonary diseases, diabetes, heart, kidney, liver, motor neurone or Parkinson disease, multiple sclerosis, severe obesity) should be prioritized for COVID-19 vaccination.

[Many countries](https://www.who.int/publications-detail-redirect/WHO-2019-nCoV-vaccines-SAGE_recommendation-immunocompromised-persons)have included PLHIV as a priority group for COVID-19 vaccination according to their epidemiological context, prioritizing vaccination for all PLHIV or those who are immunocompromised (as indicated by having a CD4 T-cell count <200/mm3).  In mid-2021, according an informal WHO poll survey conducted with more than 100 countries from all regions, more than 40 have established a COVID-19 immunization policy that prioritizes vaccinations for PLHIV. However, the real coverage of COVID-19 vaccination among PLHIV in countries varies widely. A regional survey conducted at the end of 2021 in central and eastern Europe found that PLHIV had been prioritized for COVID-19 vaccination in 8 of the 22 countries and only 3 countries had formulated guidelines for the vaccination of PLHIV *(8)*. A recent study conducted in New York State, USA, found that COVID-19 vaccination coverage among PLHIV was lower than that in the general adult population (63.5% vs 75.0%) *(9)*.

Differences in demographic composition between PLHIV and the general population might partly explain lower coverage; however, coverage was 75% lower across all examined PLHIV subgroups. Unmeasured structural factors, including socioeconomic status, might further explain the lower COVID-19 vaccination coverage among this group. As countries are in different stages of the COVID-19 pandemic and vaccine rollout and have different demographic structure, they should also refer to the  [WHO SAGE Roadmap for Prioritizing Uses of COVID-19 Vaccines](https://www.who.int/publications-detail-redirect/WHO-2019-nCoV-Vaccines-SAGE-Roadmap)  for operational details.

23、**[Should all people living with HIV receive additional doses of COVID-19 vaccine?](https://www.who.int/emergencies/diseases/novel-coronavirus-2019/question-and-answers-hub/q-a-detail/coronavirus-disease-(covid-19)-covid-19-and-people-living-with-hiv)**

Currently approved COVID-19 vaccines are safe and effective in most PLHIV, with significant reduction in the risk of severe COVID-19 disease or death when the standard regimen (currently 1 or 2 doses of the approved COVID-19 vaccine depending on the product) is fully taken as recommended. However, there is a growing evidence of a suboptimal immune response to the standard course of COVID-19 vaccination in people with moderate to severe immunodeficiency, including those with advanced HIV disease, lower CD4 T-cell counts, or unsuppressed viral load.

Therefore, in people with moderate to severe [advanced HIV disease](https://www.who.int/teams/global-hiv-hepatitis-and-stis-programmes/hiv/treatment/advanced-hiv-disease), an additional vaccine dose should be administered as part of the extended primary series and should be given 1–3 months after the completion of the primary series to enhance the immune response and increase protection. This approach is [recommended by WHO](https://www.who.int/publications-detail-redirect/WHO-2019-nCoV-vaccines-SAGE_recommendation-immunocompromised-persons) as well as several other major COVID-19 guidelines.

A gradual reduction of vaccine effectiveness against SARS-CoV-2 infection has been observed after the completion of primary COVID-19 vaccine series, even in those individuals without clinical immunodeficiency and with good initial response to the primary vaccine course. Many countries are now recommending the administration of an additional vaccine dose as a booster in all individuals, including PLHIV who are asymptomatic or mild clinical immunodeficiency and CD4 counts above 200 T-cells/mm3 and virologically suppressed. [This booster dose](https://www.who.int/news/item/22-12-2021-interim-statement-on-booster-doses-for-covid-19-vaccination---update-22-december-2021) can be taken 4 to 6 months after the completion of the primary vaccination series . The objective of the booster doses strategy is to restore vaccine effectiveness from that deemed no longer sufficient. Some countries are implementing a second booster dose for their highest risk populations, including people with moderate to severe advanced HIV disease, 3–5 months after an initial booster dose . More data on the waning of protective immunity and vaccine effectiveness against severe disease and hospitalization after an initial booster dose is required before WHO endorsed recommendation on the additional booster dose. According to the WHO SAGE roadmap, the highest priority continues to be ensuring that all PLHIV receive their primary vaccine series against COVID-19, complemented by an additional dose in those with advanced HIV disease.

**Risk of development of immune escape variants in immunosuppressed patients**

People with moderate to severe immunodeficiency have an increased risk of developing severe COVID-19 and suffering from a long-term persistent infection with prolonged viral shedding due to suboptimal immune response.

For persons with immune dysfunction, continued use of nonpharmaceutical interventions (e.g., facial mask wearing) and alternative vaccine strategies (e.g., additional vaccine doses or immunogenicity testing) are recommended even after full vaccination.

This situation can create an environment for immune escape and selection of evolutionary variants. Several case reports indicated that multi-mutational SARS-CoV-2 variants can arise during such persistent infections in immunocompromised individuals and could result in novel SARS-CoV-2 variants of concern.

The findings that immunocompromised patients with persistent SARS-CoV-2 infection may generate new SARS-CoV-2 variants have several medical and public health implications. Heightened precautions should be taken to avert nosocomial transmission of COVID-19 among immunocompromised patients. Such patients should be prioritized for anti-COVID-19 immunization not only to protect them but also to mitigate persistent SARS-CoV-2 infections.

24、**[What is WHO’s position on clinical trials/research while the COVID-19 pandemic is ongoing?](https://www.who.int/emergencies/diseases/novel-coronavirus-2019/question-and-answers-hub/q-a-detail/coronavirus-disease-(covid-19)-covid-19-and-people-living-with-hiv)**

WHO is providing support and direction to the scientific community and welcomes the research and development of effective tests, vaccines, medicines and other interventions for COVID-19.

For public health emergencies, WHO has a systematic and transparent process for research and development, including for clinical trials of new drugs and vaccines. The [WHO R&D Blueprint for COVID-19](https://www.who.int/teams/blueprint/covid-19), initiated on 7 January 2020, will serve as the global strategy for research and development activities. Its aim is to fast-track the availability of effective tests, vaccines and medicines that can be used to save lives and avert large scale crises. As part of this, WHO is leading the global prioritization of candidate vaccines and therapeutics for development and evaluation. To support testing, WHO convened a scientific advisory group to develop guidance on trial designs for experimental vaccines and therapeutics.

WHO is actively following the ongoing clinical trials for existing antivirals and other medicines that are being conducted for COVID-19. WHO continues to emphasize that all clinical trials should and must follow stringent ethical and regulatory standards. Regulatory authorities have a role to play to ensure close oversight of all clinical trials that will be undertaken.

PLHIV should be offered the opportunity to participate in clinical trials that are evaluating agents for the prevention and treatment of SARS-CoV-2 infection.

25、**[What can WHO and the world do to support people living with HIV to live a healthy life?](https://www.who.int/emergencies/diseases/novel-coronavirus-2019/question-and-answers-hub/q-a-detail/coronavirus-disease-(covid-19)-covid-19-and-people-living-with-hiv)**

While WHO is working with countries to ensure fair and equitable access to safe and effective COVID-19 vaccines, it is important to continue actions to prevent SARS-CoV-2 transmission and to reduce COVID-19 deaths. Alongside the response to COVID-19, it is critical to maintain access to essential health services. This includes:

- supporting PLHIV to continue taking [antiretroviral therapy (ART)](https://www.who.int/teams/global-hiv-hepatitis-and-stis-programmes/hiv/treatment) and adapting services to make this easier and more efficient during the COVID-19 response;
- continuing to provide [HIV prevention](https://www.who.int/teams/global-hiv-hepatitis-and-stis-programmes/hiv/prevention) and [testing services](https://www.who.int/teams/global-hiv-hepatitis-and-stis-programmes/hiv/testing-diagnostics) with linkage to ART initiation as a priority;
- ensuring those who start ART can remain on it to reduce health risks and complications during COVID-19. This must be classified as [an essential service](https://www.who.int/emergencies/diseases/novel-coronavirus-2019/related-health-issues), together with prevention, diagnosis and [treatment of co-morbidities and co-infections](https://www.who.int/teams/global-hiv-hepatitis-and-stis-programmes/hiv/treatment/chronic-comorbidities-and-coinfections); and
- monitoring all PLHIV and SARS-CoV-2 infections, especially those with [advanced HIV disease](https://www.who.int/teams/global-hiv-hepatitis-and-stis-programmes/hiv/treatment/advanced-hiv-disease) or with co-morbidities.

Although there may be an increase in the risk of developing severe disease from COVID-19 among PLHIV, making sure that people have access to effective ART and other health care services they need will help minimize this risk.

For further information on COVID-19 vaccines and all WHO guidance related to COVID-19 see <https://www.who.int/emergencies/diseases/novel-coronavirus-2019/technical-guidance>.

For community friendly resources please see: <https://i-base.info/covid-19/>

#### [Coronavirus disease (COVID-19): Adolescents and youth](https://www.who.int/emergencies/diseases/novel-coronavirus-2019/question-and-answers-hub/q-a-detail/coronavirus-disease-covid-19-adolescents-and-youth)

26、**[Can adolescents catch COVID-19?](https://www.who.int/emergencies/diseases/novel-coronavirus-2019/question-and-answers-hub/q-a-detail/coronavirus-disease-covid-19-adolescents-and-youth)**

Yes. All age groups can catch COVID-19.

While we are still learning about how COVID-19 affects people, so far, data suggests that children under the age of 18 years have few deaths compared to other age groups and usually mild disease. However, cases of critical illness have been reported. As with adults, pre-existing medical problems like high blood pressure, heart and lung problems, asthma, diabetes, obesity, cancer and neurological and developmental conditions are risk factors for severe disease and intensive care admission in children.

**Further resources:**

- *Read our*[Q*&A on Coronavirus disease (COVID-19)*](https://www.who.int/news-room/questions-and-answers/item/q-a-coronaviruses)*on who is most at risk of severe illness from COVID-19*

27、**[Can adolescents spread COVID-19 to other people even if they have mild or no symptoms?](https://www.who.int/emergencies/diseases/novel-coronavirus-2019/question-and-answers-hub/q-a-detail/coronavirus-disease-covid-19-adolescents-and-youth)**

Yes. Infected people in all age groups – including adolescents - can transmit the virus to other people, even if they have mild symptoms or do not feel ill.

The virus is spread from person to person through liquid particles such as aerosols (smaller) and droplets (larger) from the nose or mouth which are spread when a person with COVID-19 coughs, sneezes or speaks. People can catch COVID-19 if they breathe in these droplets from an adolescent infected with the virus.  Therefore, it is important to stay at least 1 meter away from others. These droplets can land on objects and surfaces. People can then become infected by touching these objects or surfaces, and then touching their eyes, nose or mouth.

Further resources:

- *Read our* Q*&A on*[*Coronavirus disease (COVID-19): How is it transmitted?*](https://www.who.int/emergencies/diseases/novel-coronavirus-2019/question-and-answers-hub/q-a-detail/coronavirus-disease-covid-19-how-is-it-transmitted)*for more information on how COVID-19 spreads between people*

28、**[Since there are few known cases of adolescents getting seriously ill with COVID-19, should I go to a health facility if I develop symptoms of the disease?](https://www.who.int/emergencies/diseases/novel-coronavirus-2019/question-and-answers-hub/q-a-detail/coronavirus-disease-covid-19-adolescents-and-youth)**

If you have any symptoms suggestive of COVID-19, contact your health care provider or COVID-19 hotline for instructions and find out what to do. If you have minor symptoms, such as a slight cough or a mild fever, and no risk factors for severe disease there is generally no need to go to a health care facility. Your health provider will assess the situation and give instructions when and where to get a test, stay at home for 14 days away from others and monitor your health.

Seek medical care immediately if your health gets worse, or if there is no one in your family that can take care of you at home. If possible, call your health care provider, hotline or health facility first, so you can be directed to the right clinic. It is important that you follow the procedures put in place by your country. Ask a family member or another trusted adult how you can find out what these procedures are where you live.

*Find out more on our*[*main public advice page*](https://www.who.int/emergencies/diseases/novel-coronavirus-2019/advice-for-public)*.*

**Further resources:**

- *Read our* [Q*&A on Coronavirus disease (COVID-19)*](https://www.who.int/news-room/questions-and-answers/item/q-a-coronaviruses)*for more information on what to do if you have COVID-19 symptoms*
- *Read our*[*Q&A on Coronavirus disease (COVID-19): Home care for families and caregivers*](https://www.who.int/emergencies/diseases/novel-coronavirus-2019/question-and-answers-hub/q-a-detail/coronavirus-disease-covid-19-home-care-for-families-and-caregivers)*on when someone who has tested positive for COVID-19 can be cared for at home*

29、**[What should I do if someone in my family gets really ill with COVID-19?](https://www.who.int/emergencies/diseases/novel-coronavirus-2019/question-and-answers-hub/q-a-detail/coronavirus-disease-covid-19-adolescents-and-youth)**

Immediately seek medical care if a member of your family gets seriously ill, for example develops difficulty breathing or feels pain or pressure in the chest. If possible, either you or an adult should contact your health care provider or COVID-19 hotline for instructions and find out where and how you could get care. If your family member is confirmed as having COVID-19, you must be prepared that you and other known contacts will need to isolate for 14 days and monitor symptoms, even if you feel healthy.

*Find out more on our*[*main public advice page*](https://www.who.int/emergencies/diseases/novel-coronavirus-2019/advice-for-public)*.*

**Further resources:**

- *Read our*[*Q&A on Coronavirus disease (COVID-19): Home care for families and caregivers*](https://www.who.int/emergencies/diseases/novel-coronavirus-2019/question-and-answers-hub/q-a-detail/coronavirus-disease-covid-19-home-care-for-families-and-caregivers)*on when someone who has tested positive for COVID-19 can be cared for at home*
- *WHO has developed the guidance on*[*Considerations in the investigation of cases and clusters of COVID-19*](file:///C:/Users/baltagv/Downloads/WHO-2019-nCoV-cases_clusters_investigation-2020.3-eng.pdf)*that offers guidance to local, regional, or national health authorities what to do if a person is suspected or confirmed of having COVID-19*

30、**[I am on medication for a chronic health condition. Should I change anything?](https://www.who.int/emergencies/diseases/novel-coronavirus-2019/question-and-answers-hub/q-a-detail/coronavirus-disease-covid-19-adolescents-and-youth)**

For people with chronic conditions such as such as asthma, diabetes, TB and HIV the most important thing is to continue your medication as prescribed, attend recommended check-ups and seek medical help if you have new symptoms.

Check with your health authorities and health provider if your regular check-ups should be done differently during the COVID-19 pandemic. Some services, such as counselling, may be available remotely. For treatment of clinically stable adolescents with HIV and adolescents with TB and/or other chronic conditions, your health provider should consider multi-month prescriptions and dispensing which will reduce the frequency of your visits to the clinic and ensure continuity of treatment, if movements are disrupted during the pandemic.  Seek advice from your health authorities and health care provider on how to be protected from COVID-19 and continue your treatment as prescribed.

*Find out more on our*[*main public advice page*](https://www.who.int/emergencies/diseases/novel-coronavirus-2019/advice-for-public)*.*

**Further resources:**

- *Read our*[*Q&A on Coronavirus disease (COVID-19): HIV and antiretrovirals*](https://www.who.int/emergencies/diseases/novel-coronavirus-2019/question-and-answers-hub/q-a-detail/q-a-on-covid-19-hiv-and-antiretrovirals)*on whether people living with HIV are at increased risk of being infected with the virus that causes COVID-19*
- *Read our*[*Q&A on Coronavirus disease (COVID-19): Tuberculosis*](https://www.who.int/emergencies/diseases/novel-coronavirus-2019/question-and-answers-hub/q-a-detail/coronavirus-disease-covid-19-tuberculosis)*to find out if people with tuberculosis are likely to be at increased risk of COVID-19 infection and severe illness*

31、**[I am bored having to spend so much time at home. Since I am very unlikely to get severely ill even if I was to get COVID-19, why is it important that I follow the guidelines to prevent transmission such as keeping physical distance from other people?](https://www.who.int/emergencies/diseases/novel-coronavirus-2019/question-and-answers-hub/q-a-detail/coronavirus-disease-covid-19-adolescents-and-youth)**

Spending more time at home is difficult and can get boring, but it may help to do something you enjoy. This could be reading a book, playing games or listening to music. Try to stay connected with friends and family every day either by communicating with them by phone or internet if you can, or, if you live close to them and the local rules allow you to, by talking in-person while keeping your distance. You can also get involved with your community to help fight the transmission of the virus.

At the same time, it is still really important that you reduce your chances of being infected or spreading COVID-19 by washing your hands with soap and water or alcohol-based hand rub as often as possible, keeping at least 1 metre (3 feet) from other people, and avoiding crowded places. Even if adolescents with COVID-19 usually do not have symptoms and have mild disease, you might be one of the unlucky adolescents who does get severely ill from COVID-19, or you could spread it to others and be responsible for them getting really ill or even dying. You have the power to make choices that could save lives and together young people can play an important part in fighting COVID-19.

*Find out more on our*[*main public advice page*](https://www.who.int/emergencies/diseases/novel-coronavirus-2019/advice-for-public)*.*

Further resources:

- Read about [10 Ways Young People are Leading the Way Against COVID-19](https://womendeliver.org/2020/10-ways-young-people-are-leading-the-way-against-covid-19/)

32、**[Some of my friends are not sticking to the rules about physical distancing. What should I do?](https://www.who.int/emergencies/diseases/novel-coronavirus-2019/question-and-answers-hub/q-a-detail/coronavirus-disease-covid-19-adolescents-and-youth)**

Explain to your friends why it is important to protect themselves and others by washing their hands, avoiding touching their face, always coughing or sneezing into their elbow, sleeve, or a tissue, and cooperating with physical distancing measures and movement restrictions when called on to do so. Maybe you can share ideas for fun virtual activities that your friends can participate in, and you can encourage them to do them together with you or with other friends. For example, you can encourage them to join [YouthAgainstCOVID19](https://www.unfpa.org/sites/default/files/resource-pdf/YouthAgaistCovid19_Campaign.pdf) campaign that aims to help teach young people around the world about COVID-19 and what they can do to keep their friends, families and communities safe. This way you are giving them alternatives rather than just telling them to stay at home.

But, remember that you do not have control over other peoples’ actions so do not get into an argument or a fight to try to change their minds.

33、**[I know there is a risk of getting COVID-19, but can I still play sports?](https://www.who.int/emergencies/diseases/novel-coronavirus-2019/question-and-answers-hub/q-a-detail/coronavirus-disease-covid-19-adolescents-and-youth)**

Yes. You can play sports that are in line with the physical distancing measures and movement restrictions that are in place in your country. If you are able to go for a bicycle ride, or if you go to a park or public open space to walk, run or exercise always practice physical distancing and wash your hands with water and soap before you leave, when you get to where you are going, and as soon as you get home.  If water and soap are not immediately available, use alcohol-based hand rub.

Being physically active is good for your health, both physical and mental. Set up a regular routine to practice activities or sports that do not require close contact with others every day for 1 hour. You can do individual sports, like jogging, walking, dancing or yoga. There are many options to try. You can set up playground games indoors, such as jump rope and hop-scotch, play with your brothers and sisters, and practice some strength training activities, using improvised weights like bottles full of water or sand. If you have access to the internet, you can also join in online active games or fitness classes, or set up your own online physical exercises with your friends or classmates. Find an activity that is fun, can be done within the restrictions that are in place in your country, and makes you feel good.

**Do not exercise if you have a fever, cough and difficulty breathing.**Stay home and rest, seek medical attention and follow the directions of your local health authority.

*Further resources:*

- Read our [Q&A on How to be active during COVID-19](https://www.who.int/emergencies/diseases/novel-coronavirus-2019/question-and-answers-hub/q-a-detail/be-active-during-covid-19)

34、**[COVID-19 makes me really anxious. I feel worried and at times have difficulty coping with stress. What should I do?](https://www.who.int/emergencies/diseases/novel-coronavirus-2019/question-and-answers-hub/q-a-detail/coronavirus-disease-covid-19-adolescents-and-youth)**

In situations like a pandemic it is very normal to feel anxious and powerless, and that is ok. Here are a few suggestions on things that could help you proactively manage your anxiety:

TIP 1: DO THINGS THAT ARE GOOD FOR YOUR BODY AND MIND

Your body and mind are connected. Here are some things you can do to keep them healthy:

• Stay active! Being physically active is good for your body and can help your mind feel better too. If you can go outside, try a walk, run, bike ride, or any other sport. If indoors, try dancing, stretching, or any other movement you can do. Find what works for you – and do it!

• Eat well! Try to make healthy choices about what you eat if you can.

• Take notice of yourself and the world around you. This means becoming more aware of your breathing, your body and your surroundings. Try to BE PRESENT in the moment!

• Sleep! Try to get the right balance of sleep each night. This will help you grow well, stay healthy, and keep clear thoughts.

•  Find things that make you happy and do those things more! Listening to music, reading, playing games, chatting with friends, growing plants, cooking, drawing, playing sports…there are many possibilities! Perhaps even try something new! Just make sure that during these activities you respect physical distancing and other protection measures that are in place in your country.

TIP 2: STAY CONNECTED TO LOVED ONES

Keep in touch with your family and friends however you can. Use social media, email, phone calls, write a letter! Be creative. And if you can’t connect, then think about a memory of a shared time together.

TIP 3: RECOGNISE HOW YOU ARE FEELING

Understanding how you feel is important. Don’t ignore it. Sometimes writing your feelings down can help you to describe it. It might sound easy or simple, but try this “I feel ....... right now”.

TIP 4: BE KIND TO YOURSELF

It is okay to feel however it is that you’re feeling. Putting pressure on yourself to always ‘be happy’ or ‘stay positive’ or ‘stay productive’ can sometimes make you feel worse. Instead, if you notice you are experiencing difficult emotions, try telling yourself: “I feel worried and scared, but that does not mean I am not coping.” “It’s been a tough time, it’s okay to be upset.” “I am feeling [insert how you are feeling] and that is okay.” “These are difficult times, it’s normal to feel upset.”

Or think of something to tell yourself that works for you.

TIP 5: LISTEN TO YOUR BODY

Our body experiences and reacts to how we feel. Do you often get headaches? Do your shoulders, chest, or stomach sometimes feel tense? Try closing your eyes, and listening to your own breathing. Notice how you feel in each part of your body, starting from your head down to your toes. Notice if you are feeling any tightness, pain, or pressure in your body. Being aware of where you are feeling tension can help you release it.

TIP 6: TRY USING YOUR BREATH TO CALM YOURSELF

Slow breathing is one of the quickest ways of calming down the body when we experience feelings such as fear, worry or anger. Close your eyes and think of a calm place. Imagine yourself there, feeling relaxed.

• Focus on breathing slowly.

• Breathe in for a slow count of 3 and out for a slow count of 3.

• Practice this for a few minutes.

How do you feel after?

TIP 7: AVOID UNHEALTHY WAYS OF COPING WITH STRESS

When you are experiencing difficult feelings, it is important to find healthy ways to take care of yourself.

*Find out more on our*[#HealthyAtHome - Mental health page](https://www.who.int/campaigns/connecting-the-world-to-combat-coronavirus/healthyathome/healthyathome---mental-health)*.*

**Further resources:**

- Read our guide “[*Doing What Matters in Times of Stress: An Illustrated Guide*](https://www.who.int/publications/i/item/9789240003927)*”*
- Read more from [Voices of Youth](https://www.voicesofyouth.org/campaign/mental-health-wellbeing-guide-how-take-care-yourself-during-stressful-times) on how to take care of yourself and your relationships in stressful times
- Read about [Coping with stress during the 2019-nCoV outbreak](https://www.who.int/docs/default-source/coronaviruse/coping-with-stress.pdf?sfvrsn=9845bc3a_8)
- Your parents or legal guardians may access this resources on [Helping children cope with stress during the 2019-nCoV outbreak](https://www.who.int/docs/default-source/coronaviruse/helping-children-cope-with-stress-print.pdf?sfvrsn=f3a063ff_2)
- The [#CopingWithCOVID is a webinar series](https://www.un.org/youthenvoy/2020/04/copingwithcovid-a-webinar-series-on-young-people-and-mental-health/) that provides young people with a platform for genuine connection amid uncertainty, encouraging them to field their questions to the experts from UNICEF and WHO, and generate mental health awareness among young people. If you missed any of the sessions, you can watch them still!
- [Teamup at home: support for children during the coronavirus pandemic](https://www.warchildholland.org/projects/teamup/teamup-at-home/) is a resource specifically developed for children aged 6 to 11 but can be enjoyed by people of all ages – it provides a simple, safe and above all fun way to play and exercise in pairs or individually.

35、**[COVID-19 is everywhere in the news, and I am finding it difficult to know what is true and what is false. What should I do?](https://www.who.int/emergencies/diseases/novel-coronavirus-2019/question-and-answers-hub/q-a-detail/coronavirus-disease-covid-19-adolescents-and-youth)**

A near-constant stream of news, sometimes contradictory, can cause anyone to feel lost and distressed. Make sure to use reliable sources such as [UNICEF](https://www.unicef.org/coronavirus) and WHO’s sites to get information, or to check any information you might be getting through less reliable channels.

If you have a phone, you can use the [WHO Health Alert](https://www.who.int/emergencies/diseases/novel-coronavirus-2019/question-and-answers-hub/q-a-detail/coronavirus-disease-covid-19-adolescents-and-youth) on WhatsApp to get the latest information about the pandemic. This is a new service, which is free to use, designed to give prompt, reliable and official information 24 hours a day, worldwide. Start by clicking the [WHO Health Alert](https://api.whatsapp.com/send?phone=41798931892&text=hi&source=&data=&app_absent=), then simply text the word ‘Hi’ in a WhatsApp message to get started. Many countries have similar initiatives to provide context-specific information and updates. Keep in mind that overloading yourself with information about the COVID-19 pandemic can also be stressful, so seek information updates and practical guidance at specific times during the day and avoid listening to or following rumours that make you feel uncomfortable.

*Find out more on our*[Mythbusters page](https://www.who.int/emergencies/diseases/novel-coronavirus-2019/advice-for-public/myth-busters)

*Find out more on our*[#HealthyAtHome - Mental health page](https://www.who.int/campaigns/connecting-the-world-to-combat-coronavirus/healthyathome/healthyathome---mental-health)*.*

***Further resources:***

- *Read about*[*Coping with stress during the 2019-nCoV outbreak*](https://www.who.int/docs/default-source/coronaviruse/coping-with-stress.pdf?sfvrsn=9845bc3a_8)

36、**[Schools are reopening in some areas of my country. Is it safe to go back to school?](https://www.who.int/emergencies/diseases/novel-coronavirus-2019/question-and-answers-hub/q-a-detail/coronavirus-disease-covid-19-adolescents-and-youth)**

A decision to reopen schools in every country and area is made based on careful assessment of the situation, with consensus among all the key parties involved, including the health and education policy-makers, teachers and other school staff, parents and health and community workers. In addition, reopening of schools is carefully planned and prepared, with all necessary measures in place to protect the safety and health of everyone in the school community.

Therefore, if your school reopens, you should feel assured it is safe for you to go back to school – provided that you strictly follow the guidelines and rules that will be provided by your school.

Of course, if you have any concerns with going back to school, do not hesitate to speak out to your teachers and parents or guardians.

***Further resources:***

- *Read what WHO recommends for safe schools reopening in*[*Considerations for school-related public health measures in the context of COVID-19*](https://apps.who.int/iris/rest/bitstreams/1303058/retrieve)
- *Read our*[*Q&A on Schools and Covid-19*](https://www.who.int/emergencies/diseases/novel-coronavirus-2019/question-and-answers-hub/q-a-detail/q-a-schools-and-covid-19)

37、**[What should I do if I missed out on my education due to the COVID-19 pandemic?](https://www.who.int/emergencies/diseases/novel-coronavirus-2019/question-and-answers-hub/q-a-detail/coronavirus-disease-covid-19-adolescents-and-youth)**

Your school or place where you are studying are likely to make arrangements for you to catch up on what you have missed when your school was closed. Many schools have put in place accelerated learning modalities to help pupils catch up on learning loss.

If your school is still closed, and you cannot attend classes in person, follow the procedures that your school has put in place to give you access to educational materials and technologies (internet, texting radio, radio, or television). If you have access to the internet, you can also consult your teachers and other trusted adults to identify and access reliable online learning opportunities and resources, including those included in the [distance learning solutions recommended by UNESCO](https://en.unesco.org/covid19/educationresponse/solutions), the United Nations agency that helps countries improve their education systems. In addition, UNESCO is collecting [stories from students, teachers and parents](https://en.unesco.org/covid19/educationresponse/learningneverstops/) about how they are coping and continuing to learn during school closures. Access those stories, they might inspire you. You can also contact UNESCO and share your story! Find out [how to share it here](https://en.unesco.org/sites/default/files/unesco-learning-never-stops-tips-for-testimonies.pdf).

In places where internet connectivity is a problem, many governments have started to broadcast educational programmes on TV and radio channels during school closures. If you live in such a place, look out for educational programmes on your local TV and radio channels.

***Further resources:***

- *Studying at home due to coronavirus?*[*This is how*](https://www.voicesofyouth.org/campaign/studying-home-due-coronavirus-how-young-people-around-world-are-keeping-their-mood)*young people around the world are keeping their mood up*

38、**[Should I wear a mask at school or when playing sports?](https://www.who.int/emergencies/diseases/novel-coronavirus-2019/question-and-answers-hub/q-a-detail/coronavirus-disease-covid-19-adolescents-and-youth)**

You should not wear a mask when playing sports or doing physical activities, such as running, jumping or playing on the playground, so that it doesn’t compromise your breathing. However, remember to maintain at least a 1-metre distance from others, limit the number of friends playing together, and respect hand hygiene.

Regarding wearing masks in schools and other public places, WHO advises that people always consult and abide by local authorities on recommended practices in their area. In countries or areas where there is intense community transmission of the virus and in settings where physical distancing cannot be achieved, WHO and UNICEF advise decision makers to apply the following criteria for use of masks in schools (either in classes, corridors or communal areas) when developing national policies:

• Children aged 5 years and under should not be required to wear masks.

• For children between six and 11 years of age, the decision to use a mask will vary from place to place, and will depend on several factors, such as the intensity of transmission in the area where the child lives, local norms that influence social interactions, the child’s capacity to comply with the appropriate use of masks and availability of appropriate adult supervision, and other factors.

• Children and adolescents 12 years or older should follow the national mask guidelines for adults.

*Watch our ‘*[*How to wear a fabric mask*](https://youtu.be/ciUniZGD4tY)*’ video for a demonstration.*

*Watch our ‘*[*WHO’s recommended fabric mask materials and composition*](https://youtu.be/XEhPyZX7WiA)*’ video for more information.*

Further resources:

- *Read our*[*Q&A on children and masks related to COVID-19*](https://www.who.int/emergencies/diseases/novel-coronavirus-2019/question-and-answers-hub/q-a-detail/q-a-children-and-masks-related-to-covid-19)*for more information on precautions for younger age groups.*
- *Watch our*[*animation*](https://youtu.be/esM_ePHn0aw)*on medical and fabric masks, explaining who wears what, when and where.*
- *Read more in our guidance*[Considerations for school-related public health measures in the context of COVID-19](https://apps.who.int/iris/rest/bitstreams/1277622/retrieve) *what are the considerations in schools for wearing masks*

39、**[I am spending much more time online in social media, playing games and studying. Should I be worried about this?](https://www.who.int/emergencies/diseases/novel-coronavirus-2019/question-and-answers-hub/q-a-detail/coronavirus-disease-covid-19-adolescents-and-youth)**

COVID-19 has abruptly pushed many people’s daily lives online, and you may be spending even more hours online than before. While online solutions provide huge opportunities for continuing your learning, socializing and playing, you should try to limit the amount of screen time that is not related to your studies or physical activity. This is because you need to be physically active to keep healthy and a positive attitude. In addition, some people are sensitive to flickering lights and may get headaches, nausea, and dizziness, and even seizures if they spend too much time in front of a screen. Therefore, it is important to replace some of the recreational screen time with non-screen activities, like listening to music, reading, playing board games, and physical activity, like going for a walk or jog. Excessive gaming can lead to “gaming disorder” that leads to reduced sleep or day-night reversal, loss of appetite, aggression, headaches, and attention problems. If you experience these symptoms, seek help from your parents or a trusted adult.

*Further resources:*

- *Read our brief on*[*Addictive behaviours: Gaming disorder*](https://www.who.int/news-room/questions-and-answers/item/gaming-disorder)
- *Find out more about*[*COVID-19 and its implications for protecting children online*](https://www.unicef.org/media/67396/file/COVID-19%20and%20Its%20Implications%20for%20Protecting%20Children%20Online.pdf)

40、**[I heard I can be hurt by online contacts. What are my risks and how I can protect myself online.](https://www.who.int/emergencies/diseases/novel-coronavirus-2019/question-and-answers-hub/q-a-detail/coronavirus-disease-covid-19-adolescents-and-youth)**

Since you might be spending even more time online than before, it is wise to be aware of some of the risks. First, be careful what content you share online. Risky behaviour, such as sexting or sharing of sexualized content, can expose you to risks of blackmail, harassment and humiliation. Second, spending more time online may increase the chances that you could come into contact with online predators who seek to sexually exploit young people. When in front of webcams wear appropriate clothing and avoid using private instant messaging services in your communication with teachers. In addition, it’s important to note that some adolescents – for example those with disabilities and those perceived to be different or at greater risk of catching or spreading COVID-19 - may be at increased risk of cyberbullying and discrimination. Hurtful, discriminatory or inappropriate online contact is never okay. If in doubt, or if you feel uncomfortable or distressed about some interactions you have online, tell a parent or another trusted adult immediately.

***Further resources:***

*Find out more about*[*COVID-19 and its implications for protecting children online*](https://www.unicef.org/media/67396/file/COVID-19%20and%20Its%20Implications%20for%20Protecting%20Children%20Online.pdf)

41、**[Since my parents stopped going out to work, they have been arguing with each other much more, and in some instances, I have seen one parent harm or hurt the other either verbally or physically. I don’t feel safe at home. What should I do?](https://www.who.int/emergencies/diseases/novel-coronavirus-2019/question-and-answers-hub/q-a-detail/coronavirus-disease-covid-19-adolescents-and-youth)**

This is a difficult time. Many people – including perhaps your parents - are worrying about security, health, and money. When people are in cramped and confined living conditions, these tensions and stress can become even greater. It is normal to have disagreements. However, if the disagreements become verbal or physical, then it is right to take action.

If you are worried about what is happening in your home, or don’t feel safe, talk to a trusted adult about what worries you, and seek their advice. During an argument or a fight, try not to draw attention to yourself so that you don’t end up getting hurt. It might be useful to have a safety plan in case the violence escalates. This includes preparing a bag with essential items, like clothing, documents and electric charger, and having a neighbour, friend, relative, or shelter identified to go to should you need to leave the house immediately for safety reasons. Arrange with the trusted adult to help you alert the relevant authorities who can help you stay safe, including the police, emergency health services and social services.

In many of the countries that have been most affected by COVID-19, essential services are still available, including shelters or protection services. If there are no trusted adults for you to share your concerns with, your country may have helplines, including text services so that you can communicate with someone who can help you or give you advice.

***Further resources:***

- *Read more in the*[*COVID-19 and Ending Violence Against Women and Girls*](https://www.unwomen.org/-/media/headquarters/attachments/sections/library/publications/2020/issue-brief-covid-19-and-ending-violence-against-women-and-girls-en.pdf?la=en&vs=5006)*brief*
- *Find out more how COVID-19 can exacerbate risks of violence for women*
- *in this brief*[*COVID-19 and violence against women*](https://apps.who.int/iris/bitstream/handle/10665/331699/WHO-SRH-20.04-eng.pdf)
- *Read our*[*Guidelines for the health sector response to child maltreatment*](https://www.who.int/publications/i/item)

42、 [**Is it safe to have sex at this time?**](https://www.who.int/emergencies/diseases/novel-coronavirus-2019/question-and-answers-hub/q-a-detail/coronavirus-disease-covid-19-adolescents-and-youth)

There is no evidence that COVID-19 is transmitted through semen or vaginal fluids. However, having sex with someone means that you are very close to them. This puts one person at risk if the other person has COVID-19. Masturbation does not involve another person and carries no risk of COVID-19. Depending on the government guideline, there might be restrictions in place to meet people outside your household, so it is important you follow these guidelines. Your risk of COVID-19 is not increased if you already live in the same household as your sexual partner and you are both taking steps to protect yourself from the virus. Make sure to use condoms and contraception to avoid sexually transmitted infections and unintended pregnancy.

***Further resources:***

- *Read our Q&A on*[*Coronavirus disease (COVID-19): Contraception and family planning*](https://www.who.int/news-room/questions-and-answers/item/contraception-family-planning-and-covid-19)
- *Find out more about contraceptive counselling and services for young people during COVID-19 in this brief*[*Not in Pause.  Responding to the Sexual and Reproductive Health Needs of Adolescents During the COVID-19 Crisis*](https://www.unfpa.org/sites/default/files/resource-pdf/Not_on_Pause.pdf)

43、**[I don’t like the way someone touches me at home and we are both at home all the time because of the pandemic. What should I do?](https://www.who.int/emergencies/diseases/novel-coronavirus-2019/question-and-answers-hub/q-a-detail/coronavirus-disease-covid-19-adolescents-and-youth)**

It is wrong for anyone to do this. And it is not your fault in any way. If you are staying in the same house as the person and/or are dependent on him (it will usually be a man), that may make some of the things that you could do difficult, especially during stay-at-home restrictions due to COVID-19. Here are some things that you can consider doing to improve your safety while in the house.

- You could tell him politely but firmly that you do not want him to touch you and ask him to please stop.
- You could inform your parents or other caregivers or trusted adult in the house about what is happening.
- You could inform a trusted adult outside your home such as a neighbour or a teacher or family friend or relative.
- If you have access to a phone, you could call or text for help and support. This includes calling hotlines/helplines for children and women who are in need of help or feeling distressed or subjected to abuse, or calling a child protection service in your area. Be careful not to leave your phone calls or text messages where anyone else could access them.
- If you need to leave the house immediately because he is hurting or harming you, think of discretely (without him overhearing) pre-arranging with a neighbour or trusted relative or family friend to help you leave the house and stay with them until it is safe for you to return home.

 If you have been sexually abused or raped and need urgent medical help or care, go as soon as possible to the nearest hospital or clinic to ask for medical care.

**Further resources:**

- *Find out more what can you do if you feel home is not a safe place in our*[*Q&A Coronavirus disease (COVID-19): Violence against women*](https://www.who.int/emergencies/diseases/novel-coronavirus-2019/question-and-answers-hub/q-a-detail/coronavirus-disease-covid-19-violence-against-women)
- *Find out more how COVID-19 can exacerbate risks of violence for women in this brief*[*COVID-19 and violence against women*](https://apps.who.int/iris/bitstream/handle/10665/331699/WHO-SRH-20.04-eng.pdf)

44、**[When will this pandemic end so things can go back to normal?](https://www.who.int/emergencies/diseases/novel-coronavirus-2019/question-and-answers-hub/q-a-detail/coronavirus-disease-covid-19-adolescents-and-youth)**

We don’t know when the pandemic will end, but we know it depends on every person’s contribution in helping stop the spread of the virus. The sacrifices you have made by not seeing your friends and by not going to school for a while, and other activities, are your contributions to fighting the pandemic. By putting societies and economies on hold, we have reduced the

ability of the virus to spread through our communities. These defensive measures have helped to limit the damage the virus can cause, and bought us time to learn more about the virus and find solutions so that we can get back to a more familiar way of living. This is why it was possible to reopen schools and businesses in many places. It is important that you continue to practice the recommended measures and encourage your friends to do the same to prevent the situation from getting worse.

**Further resources:**

- *Find out more on our*[*main public advice page*](https://www.who.int/emergencies/diseases/novel-coronavirus-2019/advice-for-public)*.*
- Read more about [COVID-19 vaccine development](https://www.who.int/emergencies/diseases/novel-coronavirus-2019/covid-19-vaccines).

45、**[I feel like my future has been affected. I am not able to apply for the jobs I wanted, and now that I have a new job I find it hard to be noticed when remote working](https://www.who.int/emergencies/diseases/novel-coronavirus-2019/question-and-answers-hub/q-a-detail/coronavirus-disease-covid-19-adolescents-and-youth)**

The anonymity of working from home is really taking a toll on people and it is normal if you are feeling a bit forgotten and lost. It can feel difficult to get the attention you deserve and get your work noticed, especially if this is a new job and you do not yet know your manager and other colleagues well.  If you have a comfortable relationship with your supervisor it may be helpful to discuss whether adjustments to your workload, work schedule or other work-related matters can be made. This conversation could also be held with the person responsible for human resources at work.

There are many things you can do to feel more comfortable in the new job while working remotely. Ask your supervisor to access a training on remote working and personal effectiveness skills.  Using your time to access learning and training opportunities can not only help you develop skills for your current role but also prepare you for future roles you may desire. Agree with your manager to have regular check-in calls where you can keep your supervisor aware of accomplishments with an informative summary. If you have regular team meetings use this as an opportunity to flag your key successes, and let your co-workers to know you better. Invite your colleagues with whom you haven’t connected yet to virtual coffee dates. Don’t be shy to volunteer for new projects, and offer help to your colleagues when you can.

When solutions to problems are hard to find, it can help to talk through with people who you trust, who may be able to help or can be a person to discuss ideas with - ranging from friends, to our work colleagues or supervisors or HR or to external advisors such as career guidance and job-seeking support. Many public employment offices have moved their services online and can offer you great insights about the labour market, vacancies and training opportunities.

If you feel your mental health is taking a big toll and its affecting you much more than expected (such as affecting how well you work) then consider the following 1) engaging in stress management techniques – as reduced stress can help to improve our problem solving thinking (see [Doing What Matters in Times of Stress](https://www.who.int/publications/i/item/9789240003927) and [Managing work-related psychosocial risks during the COVID-19 pandemic](https://www.ilo.org/global/topics/safety-and-health-at-work/resources-library/publications/WCMS_748638/lang--en/index.htm)); 2) if you are still in work, consider reaching out to your staff health or human resources services who may be able to help you find to mental health and psychosocial support at work or through your local health services including online support.

Further resources:

- Read Tips for teens to learn about [5 ways to stay safe at work](http://www.ilo.org/ipecinfo/product/download.do?type=document&id=12354)
- Access this resource for young people who are about to enter, or have recently entered, the labour market to know more about [Rights@work for youth](https://www.ilo.org/wcmsp5/groups/public/---ed_emp/documents/instructionalmaterial/wcms_310206.pdf). It has hands-on examples on workplace situations and tips how to manage them.
- Watch these videos on [Positive wellbeing in the workplace during COVID-19 pandemic](https://www.ilo.org/jakarta/info/public/vid/WCMS_744569/lang--en/index.htm)
- Read this piece on[how human factors/ergonomic considerations can improve your teleworking experience](https://www.ilo.org/wcmsp5/groups/public/---ed_protect/---protrav/---safework/documents/publication/wcms_742061.pdf)
- Read and get inspired by the [stories of young people volunteering and leading collective action to mitigate the impacts of the pandemic.](https://www.ilo.org/wcmsp5/groups/public/---ed_emp/documents/publication/wcms_753026.pdf)

46、**[What can we do so that other diseases like COVID-19 do not affect us in future?](https://www.who.int/emergencies/diseases/novel-coronavirus-2019/question-and-answers-hub/q-a-detail/coronavirus-disease-covid-19-adolescents-and-youth)**

COVID-19 is a zoonotic disease which means that it existed naturally in animals before it spilled over to humans. Most new or emerging infectious diseases —whether in wildlife, domestic animals, plants or people — are driven by human activities that lead to environmental degradation. Human pressures, from deforestation, to intensive and polluting agricultural practices, to unsafe management and consumption of wildlife, increase the risk of new or emerging infectious diseases. This is why the number of emerging infectious disease outbreaks has increased steadily since 1980.

To prevent the disastrous effects of emerging infectious diseases, we have to recognize that human health is intimately connected to the health of animals and our shared environment, and take action. Millions of young people have already mobilized to demand action not only on climate and biodiversity - but also for the right to breathe clean air, and for their future on a liveable planet. You can join them to add your voice and talents to this global movement:

- Read and disseminate the [WHO Manifesto for a healthy recovery from COVID-19](https://www.who.int/news-room/feature-stories/detail/who-manifesto-for-a-healthy-recovery-from-covid-19) by disseminating it and taking personal steps
- Support actions in your country to implement the [6 prescriptions of the WHO Manifesto for a healthy recovery from COVID-19](https://www.who.int/multi-media/details/six-prescriptions-to-who-manifesto-for-healthy-recovery).

*Further resources:*

- *Find out more about in these Q&A about*[*Biodiversity and infectious diseases*](https://www.cbd.int/health/infectiousdiseases)
- *Read our*[*Manifesto for a healthy recovery from COVID-19*](file:///C:/Users/Kim/Downloads/19%20https:/www.who.int/news-room/feature-stories/detail/who-manifesto-for-a-healthy-recovery-from-covid-19)
- *Find out what actions can be taken for a*[*healthy, green recovery from COVID-19*](https://www.who.int/news-room/feature-stories/detail/actionables-for-a-healthy-recovery-from-covid-19)

（补，原30）47、**[I was due to get vaccinated for HPV, meningitis or tetanus, but immunization services have been disrupted. Should I be concerned?](https://www.who.int/emergencies/diseases/novel-coronavirus-2019/question-and-answers-hub/q-a-detail/coronavirus-disease-covid-19-adolescents-and-youth)**

WHO recommends that countries continue routine immunization services wherever feasible. For example, school-based vaccination initiatives should continue only if infection prevention and control measures are implemented to avoid increased risk of transmission of the COVID-19 virus among the students, school personnel and health care providers. However, vaccines provided to adolescents have sufficiently flexible schedules to make sure you can get the vaccine in time when vaccination services start again. For example, the HPV vaccine that requires two doses can be started any time between 9 and 14 years of age and the interval between the two doses can be longer. The minimum interval between doses is 6 months, but it can be 12 or 15 months and, if necessary, even longer. It is most important that you receive the second dose at some point in time to be fully protected.

Decisions to continue routine vaccination services are made by each country. Ask a family member or another trusted adult how you can get information about vaccination services where you live.  It is important you get the vaccines you are supposed to get, even if they have to be delayed due to the COVID-19 pandemic.

**Further resources:**

- Read our [Q&A on Vaccines and immunization: What is vaccination?](https://www.who.int/news-room/questions-and-answers/item/vaccines-and-immunization-what-is-vaccination) to find out more why is vaccination important and how does a vaccine work
- Read our [Frequently Asked Questions on Immunization in the context of COVID-19 pandemic](https://apps.who.int/iris/bitstream/handle/10665/331818/WHO-2019-nCoV-immunization_services-FAQ-2020.1-eng.pdf)
- WHO has developed the guidance that provides [Guiding principles for immunization activities during the COVID-19 pandemic](https://apps.who.int/iris/bitstream/handle/10665/331590/WHO-2019-nCoV-immunization_services-2020.1-eng.pdf)

#### [Coronavirus disease (COVID-19): Breastfeeding（48-56）](https://www.who.int/emergencies/diseases/novel-coronavirus-2019/question-and-answers-hub/q-a-detail/coronavirus-disease-covid-19-breastfeeding)

48、**[Can COVID-19 be passed through breastfeeding?](https://www.who.int/emergencies/diseases/novel-coronavirus-2019/question-and-answers-hub/q-a-detail/coronavirus-disease-covid-19-breastfeeding)**

Transmission of active COVID-19 (virus that can cause infection) through breast milk and breastfeeding has not been detected to date. There is no reason to avoid or stop breastfeeding.

49、**[In communities where COVID-19 is prevalent, should mothers breastfeed?](https://www.who.int/emergencies/diseases/novel-coronavirus-2019/question-and-answers-hub/q-a-detail/coronavirus-disease-covid-19-breastfeeding)**

Yes. In all socio-economic settings, breastfeeding improves survival and provides lifelong health and development advantages to newborns and infants. Breastfeeding also improves the health of mothers.

50、**[Following delivery, should a baby still be immediately placed skin-to-skin and breastfed if the mother is confirmed or suspected to have COVID-19?](https://www.who.int/emergencies/diseases/novel-coronavirus-2019/question-and-answers-hub/q-a-detail/coronavirus-disease-covid-19-breastfeeding)**

Yes. Immediate and continued skin-to-skin care, including kangaroo mother care, improves the temperature control of newborns and is associated with improved survival among newborn babies. Placing the newborn close to the mother also enables early initiation of breastfeeding which also reduces mortality.

The numerous benefits of skin-to-skin contact and breastfeeding substantially outweigh the potential risks of transmission and illness associated with COVID-19.

51、**[Can women with confirmed or suspected COVID-19 breastfeed?](https://www.who.int/emergencies/diseases/novel-coronavirus-2019/question-and-answers-hub/q-a-detail/coronavirus-disease-covid-19-breastfeeding)**

Yes. Women with confirmed or suspected COVID-19 can breastfeed if they wish to do so. They should:

- Wash hands frequently with soap and water or use alcohol-based hand rub and especially before touching the baby;
- Wear a medical mask during any contact with the baby, including while feeding;
- Sneeze or cough into a tissue. Then dispose of it immediately and wash hands again;
- Routinely clean and disinfect surfaces that mothers have touched.

It is important to replace medical masks as soon as they become damp and dispose of them immediately. Masks should not be reused or touched in the front.

52、**[If a mother confirmed or suspected to have COVID-19 does not have a medical face mask should she still breastfeed?](https://www.who.int/emergencies/diseases/novel-coronavirus-2019/question-and-answers-hub/q-a-detail/coronavirus-disease-covid-19-breastfeeding)**

Yes. Breastfeeding unquestionably reduces mortality in newborns and infants and provides numerous lifelong health and brain development advantages to the child.

Mothers with symptoms of COVID-19 are advised to wear a medical mask, but even if this is not possible, breastfeeding should be continued. Mothers should follow other infection prevention measures, such as washing hands, cleaning surfaces, sneezing or coughing into a tissue.

Non-medical masks (e.g. home-made or cloth masks) have not been evaluated. At this time, it is not possible to make a recommendation for or against their use.

53、**[I have confirmed or suspected COVID-19 and am too unwell to breastfeed my baby directly. What can I do?](https://www.who.int/emergencies/diseases/novel-coronavirus-2019/question-and-answers-hub/q-a-detail/coronavirus-disease-covid-19-breastfeeding)**

 If you are too unwell to breastfeed your baby due to COVID-19 or other complications, you should be supported to safely provide your baby with breast milk in a way possible, available, and acceptable to you. This could include:

- Expressing milk;
- Donor human milk.

If expressing breast milk or donor human milk are not feasible then consider wet nursing (another woman breastfeeds the child) or infant formula milk with measures to ensure that it is feasible, correctly prepared, safe and sustainable.

54、**[I had confirmed or suspected COVID-19 and was unable to breastfeed, when can I start to breastfeed again?](https://www.who.int/emergencies/diseases/novel-coronavirus-2019/question-and-answers-hub/q-a-detail/coronavirus-disease-covid-19-breastfeeding)**

You can start to breastfeed when you feel well enough to do so. There is no fixed time interval to wait after confirmed or suspected COVID-19. There is no evidence that breastfeeding changes the clinical course of COVID-19 in a mother. Health workers or breastfeeding counsellors should support you to relactate.

55、**[I have confirmed or suspected COVID-19, is it safer to give my baby infant formula milk?](https://www.who.int/emergencies/diseases/novel-coronavirus-2019/question-and-answers-hub/q-a-detail/coronavirus-disease-covid-19-breastfeeding)**

No. There are always risks associated with giving infant formula milk to newborns and infants in all settings. The risks associated with giving infant formula milk are increased whenever home and community conditions are compromised, such as reduced access to health services if a baby becomes unwell, reduced access to clean water and/or access to supplies of infant formula milk are difficult or not guaranteed, affordable and sustainable.

The numerous benefits of breastfeeding substantially outweigh the potential risks of transmission and illness associated with the COVID-19 virus.

56、**[Can a breastfeeding woman get vaccinated against COVID-19?](https://www.who.int/emergencies/diseases/novel-coronavirus-2019/question-and-answers-hub/q-a-detail/coronavirus-disease-covid-19-breastfeeding)**

Yes, women who are breastfeeding can take the vaccine when it becomes available to them.

None of the currently approved vaccines use the live virus, so there is no risk of passing the virus to the baby through breastmilk.

There is also some evidence that, after vaccination, antibodies are found in breastmilk, which may help protect the baby against COVID-19.

#### [Coronavirus disease (COVID-19): Casirivimab and Imdevimab -  monoclonal antibody therapy（57-63）](https://www.who.int/emergencies/diseases/novel-coronavirus-2019/question-and-answers-hub/q-a-detail/coronavirus-disease-(covid-19)-casirivimab-and-imdevimab---monoclonal-antibody-therapy)

57、**[I have COVID-19, should I be prescribed this monoclonal antibody therapy?](https://www.who.int/emergencies/diseases/novel-coronavirus-2019/question-and-answers-hub/q-a-detail/coronavirus-disease-(covid-19)-casirivimab-and-imdevimab---monoclonal-antibody-therapy)**

A combination of casirivimab and imdevimab, a monoclonal antibody cocktail developed by Regeneron, is recommended for:

Patients with confirmed non-severe COVID-19 at highest risk for hospitalization.

- - Those at highest risk are typically individuals older than 60, have a chronic disease, are immunocompromised, or are not vaccinated.
- Patients with severe or critical COVID-19* who do not have antibodies to the COVID-19 virus    (i.e., those who are seronegative).

This drug should be administered by a healthcare worker in a monitored clinical setting along with the current standard of care for COVID-19 which may include oxygen and other medications.

*A patient has severe COVID-19 when they have signs of pneumonia, severe respiratory distress, and their blood oxygen level is low. A patient has critical COVID-19 when they need life sustaining treatment, have acute respiratory distress syndrome, or have septic shock (evidence of injury to other organs ).

[More.](https://www.bmj.com/content/370/bmj.m3379)

58、**[How is this monoclonal antibody therapy administered and what is the dosage?](https://www.who.int/emergencies/diseases/novel-coronavirus-2019/question-and-answers-hub/q-a-detail/coronavirus-disease-(covid-19)-casirivimab-and-imdevimab---monoclonal-antibody-therapy)**

Casirivimab and imdevimab should be administered by a healthcare worker in a monitored clinical setting intravenously through an infusion (drip). In some instances, the medication may be given subcutaneously through an injection.

The dosage of the medication will be determined by the healthcare provider.

For patients with non-severe COVID-19 the total dose of casirivimab and imdevimab is 1200 – 2400 mg given once intravenously. Alternatively, a patient may receive a total one-time dose of 1200 mg subcutaneously.

For patients with severe or critical COVID-19 the total dose of casirivimab and imdevimab is 2400 – 8000 mg given once intravenously.

59、**[Is this monoclonal antibody therapy suitable for anyone with COVID-19?](https://www.who.int/emergencies/diseases/novel-coronavirus-2019/question-and-answers-hub/q-a-detail/coronavirus-disease-(covid-19)-casirivimab-and-imdevimab---monoclonal-antibody-therapy)**

There is limited data regarding the use of casirivimab and imdevimab in patients who are pregnant or breastfeeding. If you are pregnant or breastfeeding discuss the risks and benefits of this drug with your healthcare provider first.

60、**[How does this monoclonal antibody therapy act?](https://www.who.int/emergencies/diseases/novel-coronavirus-2019/question-and-answers-hub/q-a-detail/coronavirus-disease-(covid-19)-casirivimab-and-imdevimab---monoclonal-antibody-therapy)**

These drugs (casirivimab and imdevimab) are antibodies like the ones produced by our bodies when they are faced with the COVID-19 virus. They act by blocking the ability of the virus to infect our body’s cells.

Casirivimab and imdevimab are a combination of two recombinant human antibodies that targets a different part of the spike protein, the part of the coronavirus responsible for infecting human cells.

61、**[Are there any side effects? Interactions with other drugs?](https://www.who.int/emergencies/diseases/novel-coronavirus-2019/question-and-answers-hub/q-a-detail/coronavirus-disease-(covid-19)-casirivimab-and-imdevimab---monoclonal-antibody-therapy)**

In rare instances, patients receiving casirivimab and imdevimab can develop severe allergic reactions. If this occurs, appropriate medical therapy should be initiated immediately. This is why it is important for patients to receive this medication in the presence of a healthcare professional in a monitored clinical setting.

Infusion-related reactions are rare and may occur while receiving the infusion or up to 24 hours after completion. If during the infusion a severe or life-threatening reaction occurs, the infusion may be given more slowly or stopped. Signs of an infusion-related reaction include fever, difficulty breathing, reduced oxygenation, chills, fatigue, irregular heartbeat, chest pain or discomfort, weakness, nausea, headache, angioedema (swelling of the lips), throat irritation, hypertension (high blood pressure), hypotension (low blood pressure), throat irritation, rash, tching, muscle aches, feeling faint, passing out, dizziness, and diaphoresis (sweating).

62、**[Do patients receiving the monoclonal antibody therapy need monitoring?](https://www.who.int/emergencies/diseases/novel-coronavirus-2019/question-and-answers-hub/q-a-detail/coronavirus-disease-(covid-19)-casirivimab-and-imdevimab---monoclonal-antibody-therapy)**

It is important for patients to receive this medication in the presence of a healthcare professional in a monitored clinical setting in the event of rare severe allergic reactions.

63、**[How expensive is this drug? Is it widely available?](https://www.who.int/emergencies/diseases/novel-coronavirus-2019/question-and-answers-hub/q-a-detail/coronavirus-disease-(covid-19)-casirivimab-and-imdevimab---monoclonal-antibody-therapy)**

There is currently a shortage of casirivimab and imdevimab globally and these medications are expensive.

WHO and partners are working with the manufacturer of this therapeutic, Roche Pharmaceutical, to see how the medication can be developed and obtained at a lower price. It is important that we improve access to this life saving medication in low- and middle-income countries  .

WHO has launched a call to manufacturers to submit biosimilar versions of this drug for approval or [prequalification](https://extranet.who.int/pqweb/sites/default/files/documents/EOI-COVID-19_v4.pdf). This would allow global production to be ramped up so that more people can access the drugs.

#### [Coronavirus disease (COVID-19): Children and masks（64-73）](https://www.who.int/emergencies/diseases/novel-coronavirus-2019/question-and-answers-hub/q-a-detail/q-a-children-and-masks-related-to-covid-19)

64、**[Should my child wear a mask?](https://www.who.int/emergencies/diseases/novel-coronavirus-2019/question-and-answers-hub/q-a-detail/q-a-children-and-masks-related-to-covid-19)**

Decisions about mask use in children should be driven by what is in the best interest of the child. Mask use should be flexible, so that children can continue play, education and everyday activities. These activities are an important part of child development and health. No child should be denied access to school or activities because of lack of a mask.

Some countries and regions may have specific policies or recommendations in place. As always, follow the guidance provided by your country or local health department or ministry.

WHO and UNICEF recommend the following:

1. Children aged **5 years and under**do not need to wear a mask because in this age group, they may not be able to properly wear a mask without help or supervision.

2. **In areas where SARS-CoV-2 is spreading**, children**ages 6-11 years**are recommended to wear awell-fitted mask

- in indoor settings where ventilation is poor or unknown, even if physical distancing of at least 1 meter can be maintained; and
- in indoor settings that have adequate ventilation when physical distancing of at least 1 meter cannot be maintained.

3. Adolescents **12 years or older** should follow the same WHO recommendations for mask use as adults:

A well-fitted mask that covers the nose and mouth should be worn in settings where SARS CoV-2 is spreading, regardless of vaccination status or history of prior infection, when interacting with individuals who are not members of their household:

- in indoor settings where ventilation is known to be poor or cannot be assessed, or the ventilation system is not properly maintained, regardless of whether physical distancing of at least 1 meter can be maintained
- in indoor settings that have adequate ventilation if physical distancing of at least 1 meter cannot be maintained; or
- in outdoor settings where physical distancing of at least 1 meter cannot be maintained.

As much as possible, it is important that children of all ages keep their hands clean when putting on and taking off their mask.

A safe environment should be created for children who are not able to tolerate a mask, including requirements for caregivers, teachers or other adults interacting with the child to wear a mask and to be vaccinated against COVID-19 according to national vaccination policies.

65、**[My child is under 5 years old. Should they wear a mask?](https://www.who.int/emergencies/diseases/novel-coronavirus-2019/question-and-answers-hub/q-a-detail/q-a-children-and-masks-related-to-covid-19)**

In general, children aged **5 years and under** do not need to wear masks. However, there may be times when caregivers will choose to put a mask on a child – for example, if the child has contact with a person who is at a high risk of developing severe disease or is around someone who is ill. Children of this age should not wear masks for a long duration or without supervision.

66、**[My child has disabilities. Should they wear a mask?](https://www.who.int/emergencies/diseases/novel-coronavirus-2019/question-and-answers-hub/q-a-detail/q-a-children-and-masks-related-to-covid-19)**

Children with cognitive or respiratory impairments, developmental disorders, disabilities or other specific health conditions who experience difficulties wearing a mask should not be required to do so.

The individual decision for a child to wear a mask should be discussed in consultation with the child's medical provider where possible.

A safe environment should be created for children who are not able to tolerate a mask, including requirements for caregivers, teachers or other adults interacting with the child to wear a mask and to be vaccinated against COVID-19 according to national vaccination policies

67、**[My child has a medical condition that compromises their immune system. Should they wear a mask?](https://www.who.int/emergencies/diseases/novel-coronavirus-2019/question-and-answers-hub/q-a-detail/q-a-children-and-masks-related-to-covid-19)**

The use of a medical mask is recommended for children with a higher risk of severe complications from COVID-19. This includes children with underlying noncommunicable diseases such as diabetes, cardiac disease, chronic lung disease, chronic kidney disease, immunosuppression, HIV, obesity, mental disorders and cancer.

68、**[What type of mask should my child wear?](https://www.who.int/emergencies/diseases/novel-coronavirus-2019/question-and-answers-hub/q-a-detail/q-a-children-and-masks-related-to-covid-19)**

Children should wear a**well-fitted mask that overs the nose, mouth and chin**.

There are three types of masks that *WHO recommends for the public*:*

- reusable non-medical masks that comply with standards
- disposable medical masks
- other types of reusable non-medical masks, including homemade multi-layered (fabric) masks are acceptable when other options are not available.

*More on the types of masks, how to choose them, and how to wear them is available here:  <https://www.who.int/emergencies/diseases/novel-coronavirus-2019/advice-for-public/when-and-how-to-use-masks>

69、**[How should children wear a mask?](https://www.who.int/emergencies/diseases/novel-coronavirus-2019/question-and-answers-hub/q-a-detail/q-a-children-and-masks-related-to-covid-19)**

Adults and children should follow the same principles for safe mask wearing. Some children may not be able to properly wear a mask without help or supervision. Parents or caregivers who help children with masks should be prepared to talk about mask safety and help children understand how to wear masks properly. If you are helping a child to put on or take off a mask, be sure to follow the same steps when you put on and take off your own mask.

Children should be encouraged to clean their hands before putting on their mask and after taking it off and to wear a well-fitted mask that covers the nose, mouth and chin. It is important to not share masks with others.

More information, including videos on how to put on, take off and care for a mask, can be found here:
<https://www.who.int/emergencies/diseases/novel-coronavirus-2019/advice-for-public/when-and-how-to-use-masks>

70、**[Should my child wear a mask at home?](https://www.who.int/emergencies/diseases/novel-coronavirus-2019/question-and-answers-hub/q-a-detail/q-a-children-and-masks-related-to-covid-19)**

Your child’s health is a priority, and there may be times when wearing a mask at home is the safest thing to do.

If you have visitors, outdoor gatherings are safer than indoor gatherings. If visitors come into your home, it may be best for everyone to wear a mask if ventilation is poor or if physical distancing of at least 1 metre cannot be maintained.

Children who have symptoms of COVID-19 should wear a medical mask at home when they are in shared spaces, as long as they can tolerate it. Family members/caregivers who come within 1 metre of the sick child at home should also wear a medical mask.

For more information refer to the [Coronavirus disease (COVID-19): Home care for families and caregivers](https://www.who.int/news-room/questions-and-answers/item/coronavirus-disease-covid-19-home-care-for-families-and-caregivers)

71、**[Should teachers or other adults interacting with children wear a mask?](https://www.who.int/emergencies/diseases/novel-coronavirus-2019/question-and-answers-hub/q-a-detail/q-a-children-and-masks-related-to-covid-19)**

In areas where SARS-CoV-2 is spreading, adults under the age of 60 interacting with children should wear a **well-fitted mask that covers the nose and mouth when they are:**

- in indoor settings where ventilation is known to be poor or cannot be assessed, or the ventilation system is not properly maintained, regardless of whether or not physical distancing of at least 1 meter can be maintained; or
- in indoor settings that have adequate ventilation if physical distancing of at least 1 meter cannot be maintained.

Adults aged 60 or over, or who have any underlying health conditions, such as heart disease, diabetes or cancer, should wear a **medical mask** due to their higher risk of getting seriously ill from COVID-19.

72、**[Should children wear a mask when playing sports or doing physical activities?](https://www.who.int/emergencies/diseases/novel-coronavirus-2019/question-and-answers-hub/q-a-detail/q-a-children-and-masks-related-to-covid-19)**

Children do not need to wear a mask when playing sports or doing physical activities, such as running, jumping or playing, as it may affect their breathing. When organizing sporting activities for children, it is important to encourage all other public health measures:

- Choose outside venues over indoor ones.
- If gatherings must be held indoors, open windows to ensure good ventilation.
- Maintain at least a 1-metre distance from others and limit the number of children playing together.
- Provide access to hand hygiene facilities and encourage children to use them.

73、**[Are there alternatives to fabric masks such as face shields?](https://www.who.int/emergencies/diseases/novel-coronavirus-2019/question-and-answers-hub/q-a-detail/q-a-children-and-masks-related-to-covid-19)**

In the context of COVID-19, some children may not be able to wear a mask due to disabilities or specific situations such as speech classes where the teacher needs to see their mouths. In these cases, face shields may be considered an alternative to masks, but they do not provide the equivalent protection in keeping the virus from being transmitted to others.

If a decision is made to use a face shield, it should cover the entire face, wrap around the sides of the face and extend to below the chin. Caution should be taken while wearing one to avoid injuries that could break it and harm the eyes or face.

#### [Coronavirus disease (COVID-19): Cleaning and disinfecting surfaces in non-health care settings（74-81）](https://www.who.int/emergencies/diseases/novel-coronavirus-2019/question-and-answers-hub/q-a-detail/coronavirus-disease-covid-19-cleaning-and-disinfecting-surfaces-in-non-health-care-settings)

74、**[What areas should be prioritized for disinfection in non-health care settings?](https://www.who.int/emergencies/diseases/novel-coronavirus-2019/question-and-answers-hub/q-a-detail/coronavirus-disease-covid-19-cleaning-and-disinfecting-surfaces-in-non-health-care-settings)**

Disinfection practices are important to reduce the potential for COVID-19 virus contamination in non-healthcare settings, such as in the home, office, schools, gyms, publicly accessible buildings, faith-based community centres, markets, transportation and business settings or restaurants. **High-touch surfaces** in these non-health care settings should be identified for priority disinfection such as door and window handles, kitchen and food preparation areas, counter tops, bathroom surfaces, toilets and taps, touchscreen personal devices, personal computer keyboards, and work surfaces

75. [**Which surface disinfectants are effective against COVID-19 in non-health care setting environments?**](https://www.who.int/emergencies/diseases/novel-coronavirus-2019/question-and-answers-hub/q-a-detail/coronavirus-disease-covid-19-cleaning-and-disinfecting-surfaces-in-non-health-care-settings)

In non-health care settings, sodium hypochlorite (bleach / chlorine) may be used at a recommended concentration of 0.1% or 1,000ppm (1 part of 5% strength household bleach to 49 parts of water). Alcohol at 70-90% can also be used for surface disinfection. Surfaces must be cleaned with water and soap or a detergent first to remove dirt, followed by disinfection.  Cleaning should always start from the least soiled (cleanest) area to the most soiled (dirtiest) area in order to not spread the dirty to areas that are less soiled.

All disinfectant solutions should be stored in opaque containers, in a well-ventilated, covered area that is not exposed to direct sunlight and ideally should be freshly prepared every day.

In indoor spaces, routine application of disinfectants to surfaces via spraying is not recommended for COVID-19. If disinfectants are to be applied, these should be via a cloth or wipe which is soaked in the disinfectant.

76、**[What protection measures should people take when using disinfectants?](https://www.who.int/emergencies/diseases/novel-coronavirus-2019/question-and-answers-hub/q-a-detail/coronavirus-disease-covid-19-cleaning-and-disinfecting-surfaces-in-non-health-care-settings)**

It is important to reduce your risk when using disinfectants:

- The disinfectant and its concentration should be carefully selected to avoid damaging surfaces and to avoid or minimize toxic effects on household members (or users of public spaces).
- Avoid combining disinfectants, such as bleach and ammonia, since mixtures can cause respiratory irritation and release potentially fatal gases.
- Keep children, pets and other people away during the application of the product until it is dry and there is no odour.
- Open windows and use fans to ventilate. Step away from odours if they become too strong. Disinfectant solutions should always be prepared in well-ventilated areas.
- Wash your hands after using any disinfectant, including surface wipes.
- Keep lids tightly closed when not in use. Spills and accidents are more likely to happen when containers are open.
- Do not allow children to use disinfectant wipes. Keep cleaning fluids and disinfectants out of the reach of children and pets.
- Throw away disposable items like gloves and masks if they are used during cleaning. Do not clean and re-use.
- Do not use disinfectant wipes to clean hands or as baby wipes.
- The minimum recommended personal protective equipment when disinfecting in non-health care settings is rubber gloves, waterproof aprons and closed shoes. Eye protection and medical masks may also be needed to protect against chemicals in use or if there is a risk of splashing.

*Note: Where cleaning and disinfection are not possible on a regular basis due to resource limitations, frequent hand washing and avoiding touching the face should be the primary prevention approaches to reduce any potential transmission associated with surface contamination.*

77、**[What is the guidance for the disinfection of outdoor spaces such as open markets, roads?](https://www.who.int/emergencies/diseases/novel-coronavirus-2019/question-and-answers-hub/q-a-detail/coronavirus-disease-covid-19-cleaning-and-disinfecting-surfaces-in-non-health-care-settings)**

In outdoor spaces, large-scale spraying or fumigation in areas such as streets or open market places for the COVID-19 virus or other pathogens is not recommended. Streets and sidewalks are not considered as routes of infection for COVID-19. Spraying disinfectants, even outdoors, can be noxious for people’s health and cause eye, respiratory or skin irritation or damage.

This practice will be ineffective since the presence of dirt or rubbish for example, inactivates the disinfectant, and manual cleaning to physically remove all matter is not feasible. This is even less effective on porous surfaces such as sidewalks and unpaved walkways. Even in the absence of dirt or rubbish, it is unlikely that chemical spraying would adequately cover surfaces allowing the required contact time to inactivate pathogens.

78、**[Are public systems for disinfecting individuals such as spraying via tunnel or chambers safe to use?](https://www.who.int/emergencies/diseases/novel-coronavirus-2019/question-and-answers-hub/q-a-detail/coronavirus-disease-covid-19-cleaning-and-disinfecting-surfaces-in-non-health-care-settings)**

No. Spraying of individuals with disinfectants (such as in a tunnel, cabinet, or chamber) is not recommended under any circumstances. This practice could be physically and psychologically harmful and would not reduce an infected person’s ability to spread the virus through droplets or contact. Even if someone who is infected with COVID-19 goes through a disinfection tunnel or chamber, as soon as they start speaking, coughing or sneezing they can still spread the virus.

The toxic effect of spraying with chemicals such as chlorine on individuals can lead to eye and skin irritation, bronchospasm due to inhalation, and potentially gastrointestinal effects such as nausea and vomiting. In addition to health safety concerns, the use of chlorine in large-scale spraying practices may prevent this resource from being used for important interventions such as drinking water treatment and environmental disinfection of health care facilities.

79、**[What are the recommended practices once back home after outdoor activities?](https://www.who.int/emergencies/diseases/novel-coronavirus-2019/question-and-answers-hub/q-a-detail/coronavirus-disease-covid-19-cleaning-and-disinfecting-surfaces-in-non-health-care-settings)**

Thorough hand hygiene: washing hands with soap and water or using alcohol-based hand gel, should be performed before touching surfaces, items, pets, and people within the household environment. Please see: [https://www.who.int/media/docs/default-source/integrated-health-services-(ihs)/infection-prevention-and-control/hand-hygiene-when-and-how-leaflet.pdf](https://cdn.who.int/media/docs/default-source/integrated-health-services-(ihs)/infection-prevention-and-control/hand-hygiene-when-and-how-leaflet.pdf?sfvrsn=a92dc108_2) 

While outside, people should always follow physical distancing measures, staying at least one metre from another person; perform hand hygiene by washing hands frequently with soap and water or using alcohol-based hand rub; follow good respiratory hygiene by covering your mouth and nose with your bent elbow or tissue when coughing or sneezing; avoid touching your eyes, nose and mouth; and avoid crowded places

80、**[Are gloves recommended for the community in public spaces to protect against COVID-19, for example when going to the grocery store supermarket?](https://www.who.int/emergencies/diseases/novel-coronavirus-2019/question-and-answers-hub/q-a-detail/coronavirus-disease-covid-19-cleaning-and-disinfecting-surfaces-in-non-health-care-settings)**

No. The use of gloves by the public in public spaces is not a recommended or proven prevention measure. Wearing gloves in public spaces does not replace the need for hand hygiene, nor does it offer any additional measure of protection against the COVID-19 virus than hand hygiene. Gloves do not provide complete protection against hand contamination, as pathogens may gain access to the hands via small defects in gloves or by contamination of the hands during glove removal. People can also transfer pathogens from one surface to another by touching with gloved hands, or even transfer pathogens to the mouth, nose, or eyes if they touch their face with gloved hands.

81、**[How should I clean food items from the grocery store, for example fruit, vegetables or packaged items?](https://www.who.int/emergencies/diseases/novel-coronavirus-2019/question-and-answers-hub/q-a-detail/coronavirus-disease-covid-19-cleaning-and-disinfecting-surfaces-in-non-health-care-settings)**

There is no evidence to date of viruses that cause respiratory illnesses being transmitted via food or food packaging. Coronaviruses cannot multiply in food; they need an animal or human host to multiply.

The COVID-19 virus is generally thought to be spread from person to person through respiratory droplets. Currently, there is no evidence to support transmission of the COVID-19 virus associated with food.

Before preparing or eating food it is important to always wash your hands with soap and water for at least 40-60 seconds. Regular food safety and handling guidance should be followed.
See: <https://www.who.int/activities/promoting-safe-food-handling>.

For food service businesses, please see the below guidance on COVID-19 and Food Safety: [https://www.who.int/teams/risk-communication/food-and-agriculture-sectors](https://www.who.int/teams/epi-win/food-and-agriculture-sectors).

#### [Coronavirus disease (COVID-19): Climate change（82-87）](https://www.who.int/emergencies/diseases/novel-coronavirus-2019/question-and-answers-hub/q-a-detail/coronavirus-disease-covid-19-climate-change)

82、**[Where can I find the latest information on COVID-19?](https://www.who.int/emergencies/diseases/novel-coronavirus-2019/question-and-answers-hub/q-a-detail/coronavirus-disease-covid-19-climate-change)**

For the latest information on COVID-19, check regularly on the WHO’s coronavirus pages: <https://www.who.int/emergencies/diseases/novel-coronavirus-2019>

83、**[Do weather and climate determine where COVID-19 occurs?](https://www.who.int/emergencies/diseases/novel-coronavirus-2019/question-and-answers-hub/q-a-detail/coronavirus-disease-covid-19-climate-change)**

No. There is currently no conclusive evidence that either weather (short term variations in meteorological conditions) or climate (long-term averages) have a strong influence on transmission. The SARS-CoV-2 virus which causes COVID-19 disease has been transmitted in all regions of the world, from cold and dry, to hot and humid climates.

SARS-CoV-2 is thought to be mainly transmitted directly from person-to-person through close contact, or through respiratory droplets produced when an infected person coughs or sneezes. People may be infected by touching exposed surfaces, but this is not thought to be a major transmission route. While temperature and humidity may influence how long the virus survives outside of the human body, this effect is likely to be small compared to the degree of contact between people.

Physical distancing and washing hands are therefore essential to breaking the chain of transmission, and are the most effective way to protect yourself, in all locations and all seasons of the year.

84、**[Will climate change make COVID-19 worse?](https://www.who.int/emergencies/diseases/novel-coronavirus-2019/question-and-answers-hub/q-a-detail/coronavirus-disease-covid-19-climate-change)**

There is no evidence of a direct connection between climate change and the emergence or transmission of COVID-19 disease. As the disease is now well established in the human population, efforts should focus on reducing transmission and treating patients.

However, climate change may indirectly affect the COVID-19 response, as it undermines environmental determinants of health, and places additional stress on health systems. More generally, most emerging infectious diseases, and almost all recent pandemics, originate in wildlife, and there is evidence that increasing human pressure on the natural environment may drive disease emergence. Strengthening health systems, improved surveillance of infectious disease in wildlife, livestock and humans, and greater protection of biodiversity and the natural environment, should reduce the risks of future outbreaks of other new diseases.

85、**[Have measures to contain COVID-19 reduced air pollution and emissions of greenhouse gases?](https://www.who.int/emergencies/diseases/novel-coronavirus-2019/question-and-answers-hub/q-a-detail/coronavirus-disease-covid-19-climate-change)**

Air pollution is a serious health risk. It kills approximately 7 million people every year and is responsible for one third of all deaths from stroke, lung cancer and heart disease. Over 90% of the global population lives in places where the WHO outdoor air quality guideline levels are not met, and about two-thirds of this exposure is caused by burning of fossil fuels, which also drives climate change.

Efforts to control COVID-19 transmission have reduced economic activity and led to temporary improvements in air quality in some areas. In contrast, as carbon dioxide and other greenhouse gases that drive climate change persist for a long time in the atmosphere, temporary emissions reductions only have a limited effect on atmospheric concentrations. Carbon dioxide levels at observing stations around the world in the first months of 2020 have been higher than in 2019.

Environmental improvements resulting from the COVID-19 response may be reversed by a rapid expansion of polluting economic activities once the measures have ended, unless there is a clear focus to promote equity, environmental health, around a just transition to a green economy.

Any short-term environmental benefits as a result of COVID-19 come at an unacceptable human and economic cost, and are no substitute for planned and sustained action on air quality and climate.

86、**[How does water scarcity affect infectious diseases like COVID-19?](https://www.who.int/emergencies/diseases/novel-coronavirus-2019/question-and-answers-hub/q-a-detail/coronavirus-disease-covid-19-climate-change)**

Access to adequate and safe water and sanitation is essential for communities to practice basic hygiene and reduce transmission of COVID-19. Access to these services in health facilities is crucial to preventing infections, reducing the spread of antimicrobial resistance and providing quality care.

One in four health care facilities around the world lacks basic water services, directly impacting over two billion people. Around 80% of the world’s population is already experiencing some level of water scarcity. Climate change further threatens the availability of water for consumption, food production, personal hygiene, and medical care, including for infectious disease.

87、**[What can the global response to COVID-19 teach us about our response to climate change?](https://www.who.int/emergencies/diseases/novel-coronavirus-2019/question-and-answers-hub/q-a-detail/coronavirus-disease-covid-19-climate-change)**

The COVID-19 pandemic is a Public Health Emergency of International Concern (PHEIC), which has claimed lives, and severely disrupted communities. Climate change is a gradually increasing stress that may be the defining public health threat of the 21^st^century. Nonetheless, common lessons can be drawn:

- Ensuring universal health coverage ([UHC](https://www.who.int/news-room/fact-sheets/detail/universal-health-coverage-(uhc))), through well-resourced, equitable health systems, is essential to protect the public from both short and long-term health threats.
- Guaranteeing global health security requires an all-hazards approach to preparedness, from infectious disease outbreaks, to extreme weather events, to climate change.
- Ensuring access to the environmental determinants of health, such as clean air, water and sanitation, safe and nutritious food, is an essential protection against all health risks. WHO estimates that avoidable environmental risks cause about a quarter of the global health burden.
- Early action saves lives. Delay in responding to clear evidence of threats, whether from pandemics, or from climate change, increases human and socioeconomic costs.
- Inequality is a major barrier in ensuring health and wellbeing, especially for the most vulnerable in society. Social and economic inequality manifests in unequal health risks. When faced with public health threats of a global scale, such as COVID-19 or climate change, we are only as strong as our weakest health system.

#### [Coronavirus disease (COVID-19): Contact tracing（88-96）](https://www.who.int/emergencies/diseases/novel-coronavirus-2019/question-and-answers-hub/q-a-detail/coronavirus-disease-covid-19-contact-tracing)

88、**[What is contact tracing?](https://www.who.int/emergencies/diseases/novel-coronavirus-2019/question-and-answers-hub/q-a-detail/coronavirus-disease-covid-19-contact-tracing)**

Contact tracing is the process of identifying, assessing, and managing people who have been exposed to someone who has been infected with the COVID-19 virus. Contact tracing and quarantine of contacts identified through contact tracing interrupt transmission between people and are essential public health tools for controlling the virus. Contact tracing can also help people who are at a higher risk of developing severe disease know earlier that they have been exposed so that they can  get medical care quicker if they go on to develop symptoms.

89、**[Can contact tracing help in controlling spread of the virus?](https://www.who.int/emergencies/diseases/novel-coronavirus-2019/question-and-answers-hub/q-a-detail/coronavirus-disease-covid-19-contact-tracing)**

Yes. Contact tracing is an essential public health tool for controlling infectious disease outbreaks, such as those caused bythe COVID-19 virus. Contact tracing can break the chains of transmission through the rapid identification, isolation and clinical care of cases, and providing supported quarantine of      contacts, meaning that virus transmission can be stopped.

90、**[Does contact tracing still work against new variants of the COVID-19 virus ?](https://www.who.int/emergencies/diseases/novel-coronavirus-2019/question-and-answers-hub/q-a-detail/coronavirus-disease-covid-19-contact-tracing)**

Yes, the principles of contact tracing remain the same for newly identified variants of the COVID-19 virus. While some of the variants of the virus are more transmissible, thorough and timely contact tracing and supported quarantine of contacts is even more important to control the spread of the virus.

91、**[How do I know if I’m a contact? What should I do if I am?](https://www.who.int/emergencies/diseases/novel-coronavirus-2019/question-and-answers-hub/q-a-detail/coronavirus-disease-covid-19-contact-tracing)**

If you think you may be a contact of someone who has recently been infected, you should be contacted by health authorities to determine if you meet the contact definition. You may also be informed directly by someone you were in contact with who later tested positive. If you have downloaded a COVID-19 proximity tracing application, you may also get notified through your mobile phone.

If you are identified as a contact, you should undertake quarantine, which means that you separate yourself from others in your household, stay there and monitor your health for any signs of illness. Some countries have set up dedicated facilities to enable contacts to complete quarantine outside their household. Contact your local health authorities to find out how you can safely quarantine.

WHO recommends that you stay in quarantine for 14 days after you were last in contact with the person infected with the COVID-19 virus, but the duration of quarantine may vary by country. Check with your local or national health authority.

92、**[How is digital proximity tracing data stored and protected?](https://www.who.int/emergencies/diseases/novel-coronavirus-2019/question-and-answers-hub/q-a-detail/coronavirus-disease-covid-19-contact-tracing)**

The data storage for tracing apps can either bestored and processed on a central server managed by the national public health authority, or stored and processed on the users’ phones. There is a consensus by several data protection authorities that storing data on users’ phones enhances privacy, since users have greater control over the amount of information that they share with health authorities. The collection and use of such data by health authorities can therefore be limited to what is strictly necessary for the operation of a digital tracing system.

Whichever approach is selected, governments and third parties should ensure digital tracing systems follow WHO guidance on ethical considerations on the use of digital proximity tracing for COVID-19 contact tracing. Protecting the personal data and privacy of individuals participating in digital tracing is critical to ensure the protection of human rights and civil liberties as well as the establishment of public trust.

93、**[What is quarantine? And how is it different from isolation?](https://www.who.int/emergencies/diseases/novel-coronavirus-2019/question-and-answers-hub/q-a-detail/coronavirus-disease-covid-19-contact-tracing)**

Quarantine is the separation of contacts from others after exposure to a probable or confirmed COVID-19 case – you may or may not be infected.

Isolation is the separation of people who are known to be infected with the COVID-19 virusfrom other who are not infected.

Both quarantine and isolation help to stop the spread of the virus.

94、**[How do I manage quarantine and how do I take care of myself during quarantine?](https://www.who.int/emergencies/diseases/novel-coronavirus-2019/question-and-answers-hub/q-a-detail/coronavirus-disease-covid-19-contact-tracing)**

WHO recommends supported quarantine for 14 days from the last contact with a confirmed case to minimize risk of infecting others. You should remain separated from others in the household for the duration of quarantine. If you cannot be in a separate room, stay at least one metre away from others, the farther the better. Make sure you have enough foodand water, and a way to communicate with others. The quarantine space should be well ventilated. You should minimize your contact with others. If you do have someone visit you during quarantine, you should both wear medical masks, keep windows open if possible and clean your hands before and after being together. Ideally, you should have only one visitor provide food and supplies during the quarantine period and this should be someone at low risk of developing severe COVID-19.

Quarantinemay cause worry or anxiety, and this is normal. Thiscan also be an opportunity to take time to care for yourself. Consider reconnecting with loved ones via telephone or phone apps, watch movies or read books, exercise indoors, make crafts, or catch up on things you haven’t had time for.  To help you get groceries, medicine and other necessities, ask family and friends or use delivery services.

 To learn more about taking care of yourself during quarantine, watch this video：https://youtu.be/9OVD4vmcabI

95、**[What can I do as an individual to support contact tracing measures in my community?](https://www.who.int/emergencies/diseases/novel-coronavirus-2019/question-and-answers-hub/q-a-detail/coronavirus-disease-covid-19-contact-tracing)**

Everyone has a part to play to bring COVID-19 under control, and contact tracing begins with informed, engaged and enabled communities. Understand the local public health and social measures and collaborate with health authorities for case and contact investigation. Agree to monitoring, report signs or symptoms of COVID-19 promptly, and be prepared to quarantine or go into isolation if you become a confirmedcase of COVID-19.

In addition, you can, while respecting physical distancing and all other protective measures, provide support to relatives or friends who have to be isolated or undertake quarantine. Check in on them by phone or offer to bring supplies to their house, if needed. Transmission of the COVID-19 virus can only be stopped if we all play our role to protect our family, friends and community.

96、**[If I’m travelling outside my country, do I need to provide accurate information to contact tracing personnel at the airport or at my destination? What if I’m concerned about how my private information is going to be used?](https://www.who.int/emergencies/diseases/novel-coronavirus-2019/question-and-answers-hub/q-a-detail/coronavirus-disease-covid-19-contact-tracing)**

Yes, providing accurate information when requested is important to successful contact tracing activities. National authorities may ask incoming travelers to report their health status at the time of travel, possible exposures to confirmed or probable COVID-19 cases within the previous two weeks, and to provide their contact details so they can be located for health monitoring or international contact tracing purposes. Authorities may also require arriving passengers to download and use a national COVID-19 digital proximity tracing app on arrival.  Please consult national authorities/airline companies to verify details before you travel.

#### [Coronavirus disease (COVID-19): Contact tracing for responders（97-104）](https://www.who.int/emergencies/diseases/novel-coronavirus-2019/question-and-answers-hub/q-a-detail/coronavirus-disease-(covid-19)-contact-tracing-for-responders)

97、**[How does contact tracing work?](https://www.who.int/emergencies/diseases/novel-coronavirus-2019/question-and-answers-hub/q-a-detail/coronavirus-disease-(covid-19)-contact-tracing-for-responders)**

Contact tracing identifies, and monitors people who have been exposed to someone who has been infected with SARS-CoV-2, and involves several steps:

Defining contacts: Contact definitions may vary by country. WHO defines a contact as a person who has been exposed to someone else who has had recent probable or confirmed SARS-CoV-2 infection: 1. face-to-face contact with a probable or confirmed case within 1 meter and for at least 15 minutes; 2. direct physical contact with a probable or confirmed case; 3. direct care for a patient with probable or confirmed COVID-19 disease without the use of recommended PPE; or 4. other situations as indicated by local risk assessments. Exposure is considered any time between 2 days before to at least 13 days after the case started to show symptoms. For asymptomatic cases, a contact is anyone who has been exposed between 2 days before and 10 days after the date on which the sample that led to confirmation of infection was taken.

Identifying contacts: this is usually done through an interview with the person infected with SARS-CoV-2 to find out who they have been in contact with during the period of time described above. There are other ways to identify contacts depending on the setting, for example through membership or attendance lists of places the case visited during the period described above, or through public notices. Digital proximity tracing applications have also been developed to help identify and notify contacts through automated processes.

Informing contacts: each identified contact should be contacted to confirm whether they meet the definition of a contact. This may be done through health authorities and/or via digital tracing proximity applications. Cases may also be encouraged to inform their own contacts. Each person identified as a contact will be informed about the goal of contact tracing, the process (including how their personal data will be protected), how to undertake quarantine and for how long, and who to contact with any concerns or questions. Additional information should be provided on symptoms to monitor throughout the quarantine period, and what to do if a contact returns a positive test or becomes unwell.

Managing and monitoring contacts: contacts should be encouraged and supported during the  quarantine period. WHO recommends that the quarantine period ends 14 days after the contact was last exposed to the person who has been infected with SARS-CoV-2. However, the duration of the quarantine period may vary between countries.

Data processes and analysis: The information collected from each contact is stored in a secure database. These processes vary from country to country. See question What should be considered for data protection?

98、**[Who is defined as a contact?](https://www.who.int/emergencies/diseases/novel-coronavirus-2019/question-and-answers-hub/q-a-detail/coronavirus-disease-(covid-19)-contact-tracing-for-responders)**

A contact is currently defined as anyone who had direct contact with, or was within 1 metre for at least 15 minutes, with a person who may be infected or is infected with SARS-CoV-2 while they were infectious or potentially infectious, even if the person with the infection did not have symptoms. National and local public health authorities may have additional considerations to define contacts according to local risk assessments.

Contacts should be supported to quarantine to limit the possibility of exposing other people to infection      if they become ill.(The complete definition of a contact can be found [here](https://www.who.int/publications-detail-redirect/WHO-2019-nCoV-Surveillance_Case_Definition-2022.1)).

99、**[What is backward contact tracing?](https://www.who.int/emergencies/diseases/novel-coronavirus-2019/question-and-answers-hub/q-a-detail/coronavirus-disease-(covid-19)-contact-tracing-for-responders)**

Backward contact tracing refers to the process of trying to understand how the case became infected, also known as case or source investigation. While traditional contact tracing identifies people who were exposed to cases and may become cases themselves, backward contact tracing or source investigation looks back in time to identify settings or events where exposure to the virus may have occurred. This may help public health authorities identify more cases (such as at an event or setting where the case may have been infected) and identify settings that have led to infection. This can inform the development of targeted public health and social measures to reduce the overall number of cases.

100、**[When should contact tracing be implemented?](https://www.who.int/emergencies/diseases/novel-coronavirus-2019/question-and-answers-hub/q-a-detail/coronavirus-disease-(covid-19)-contact-tracing-for-responders)**

Comprehensive contact tracing should be implemented each time cases or clusters (groups of linked cases) are identified. During intense transmission, contact tracing capacities may be overwhelmed, so contact tracing     activities may  focus on household contacts, healthcare workerscontacts, contacts in high-risk closed settings (such as dormitories, institutions, long-term living facilities),and contacts at higher risk of developing severe COVID-19.

It is important to maintain contact tracing and quarantine of contacts even when the number of new cases may be decreasing, and/or public health and social measures may be being relaxed, in order to ensure that transmission continues to reduce.

101、**[How do you form a contact tracing workforce?](https://www.who.int/emergencies/diseases/novel-coronavirus-2019/question-and-answers-hub/q-a-detail/coronavirus-disease-(covid-19)-contact-tracing-for-responders)**

A contact tracing workforce can be drawn from many settings, including individuals connected with local government, civil society, non-governmental organizations, universities and community volunteers. Ideally, a gender-balanced contact tracing team should be recruited from the community and have general literacy, strong communication skills, local language proficiency and an understanding of the local context and culture.

They should be provided with adequate training to ensure efficiency, accuracy and good communication skills when implementing case and contact investigations, and integrated within the wider COVID-19 response team.

Several training materials have been developed by WHO and partners and may be adapted to local needs. Many are available through the Global Outbreak Alert and Response Network (GOARN) [knowledge platform](https://extranet.who.int/goarn/COVID19Hub), and [OpenWHO](https://openwho.org/). Training should include the basics of virus transmission, prevention and control measures; how to monitor signs and symptoms; and standard operating procedures for contact tracing, including interview tips and ethics of public health surveillance and quarantine. Contact tracers should also be briefed on their rights, roles and responsibilities, including for occupational safety and health.

It is important for public health authorities to train the contact tracing workforce when there is no or low transmission, and anticipate ways to be able to scale the size of the trained contact tracing workforce, if transmission increases.

102、**[What are some of the challenges to effective contact tracing for COVID-19?](https://www.who.int/emergencies/diseases/novel-coronavirus-2019/question-and-answers-hub/q-a-detail/coronavirus-disease-(covid-19)-contact-tracing-for-responders)**

Some of the challenges are the availability of a trained contact tracing workforce, availability of resources for contact tracing and community engagement.

Another main challenge is the intensity of the COVID-19 transmission. In situations of intense transmission, public health resources can quickly become overwhelmed and often cannot cope with the workload of identifying contacts and monitoring them. In such situations, WHO recommends focusing on the contacts with highest exposure and those most at risk of developing severe disease.

103、**[What are some digital tools used to support contact tracing? How can they enhance contact tracing processes?](https://www.who.int/emergencies/diseases/novel-coronavirus-2019/question-and-answers-hub/q-a-detail/coronavirus-disease-(covid-19)-contact-tracing-for-responders)**

Classic interview-based contact tracing relies heavily on the presence of a trained workforce to carry out      essential activities such as contact elicitation, notification and follow-up. However, this workforce can be quickly overwhelmed in the context of widespread SARS-CoV-2 transmission.

Electronic tools and information technology have been used to enhance the efficiency ofcontact tracing processes, and are currently being used in the COVID-19 pandemic.  So far, no single digital tool addresses all the steps required to monitor end-to-end contact tracing and quarantine of contacts. Oversight from public health workforce is still required. The technical and ethical requirements related to the use of such digital tools should be considered  when making decisions as to the use of these tools. Digital tools that support contact tracing processes may be broadly divided into three categories based on their public health function during specific steps of the contact tracing process:

- **Identifying and notifying contacts:**These solutions are intended for use by the general public, health professionals and the contact tracing team. They include **digital proximity tracing tools** that use systems based on Bluetooth or GPS location signaling, to notify users who have been in close proximity and prolonged contact to individuals who tested positive for COVID-19 and registered their status in the tool. There are also **location-based digital contract tracing tools** that use quick response (QR) codes that can be scanned by smartphones users when they visit a venue, so that if they later test positive for COVID-19, other application users who attended the same venue, at the same time may be sent an alert, if local public health authorities deem it necessary.
- **Monitoring contacts**:These tools are intended for use by those identified as contacts, health professionals and the contact tracing team. They include **symptoms checker tools** that can help contacts  to self-monitor and report the presence or absence of symptoms to health professionals who may conduct further assessments, health counselling and connect them with the public health authorities for testing and other supportive services. These tools may be particularly helpful in settings where contact tracing workforce personnel may be limited and/or there are physical or security barriers preventing in-person visits by contact tracing teams.
- **Surveillance data management and analysis:** These solutions are used by public health professionals to collect, manage, analyse and visualize data collected by contact tracing teams which link cases and contacts. They include **outbreak response tools**(such as Go.Data, Commcare, SORMAS, etc.) that can be used for case investigation, listing and monitoring of contacts, and analysis.

It is important to note that these tools cannot substitute a well-trained health and community workforce, qualified supervisors, decentralized operations and good coordination; which are all necessary criteria for successful and effective contract tracing. More information on digital tools for contact tracing can be accessed [here](https://www.who.int/publications-detail-redirect/WHO-2019-nCoV-Contact_Tracing-Tools_Annex-2020.1) and ethical considerations to guide the use of digital proximity tracking technologies is available [here](https://www.who.int/publications-detail-redirect/WHO-2019-nCoV-Ethics_Contact_tracing_apps-2020.1).

104、**[What should be considered for data protection?](https://www.who.int/emergencies/diseases/novel-coronavirus-2019/question-and-answers-hub/q-a-detail/coronavirus-disease-(covid-19)-contact-tracing-for-responders)**

The ethics of public health information, data protection, and data privacy must be considered at all levels of contact tracing activities, including training and use of tools. In particular:

- Safeguards must be in place to guarantee privacy and data protection in accordance with the legal frameworks of the countries where systems are implemented.
- Everyone involved in contact tracing must adhere to the ethical principles of handling personal information, to ensure responsible data management and respect for privacy throughout the process.
- How data will be handled, stored, and used needs to be communicated to those concerned in a clear and transparent manner. This is important for buy-in and engagement as well as to avoid misperceptions that could jeopardize the effectiveness of a contact tracing programme.
- Digital tools used for contact tracing should be assessed before use to ensure safeguarding data protection according to national regulations.

See WHO’s interim guidance on [ethical considerations to guide the use of digital proximity tracking technologies for COVID-19 contact tracing.](https://www.who.int/publications-detail-redirect/WHO-2019-nCoV-Ethics_Contact_tracing_apps-2020.1)

#### [Coronavirus disease (COVID-19): Contraception and family planning（105-114）](https://www.who.int/emergencies/diseases/novel-coronavirus-2019/question-and-answers-hub/q-a-detail/coronavirus-disease-covid-19-contraception-and-family-planning)

105、**[Is contraception/ family planning safe to use during the COVID-19 pandemic?](https://www.who.int/emergencies/diseases/novel-coronavirus-2019/question-and-answers-hub/q-a-detail/coronavirus-disease-covid-19-contraception-and-family-planning)**

Yes. All modern methods of contraception are safe to use, including during the COVID-19 pandemic.

If you have had a baby in the last six months or have a health condition, such as diabetes, high blood pressure, or breast cancer – or if you smoke – seek advice from a health care professional to ensure you are using a method of contraception which is suitable and safe for you.

106、**[I want to avoid getting pregnant during the COVID-19 pandemic. What can I do?](https://www.who.int/emergencies/diseases/novel-coronavirus-2019/question-and-answers-hub/q-a-detail/coronavirus-disease-covid-19-contraception-and-family-planning)**

If you do not want to become pregnant, you should start or continue to use your contraceptive method of choice. You may be able to access information and contraceptive services from a healthcare provider by phone or online.

If you cannot access these services you may opt for a method that is available without a prescription (such as condoms, spermicides, diaphragm, pills, or emergency contraceptive pills) from a nearby pharmacy or drug shop

107、**[I can’t access my contraceptive method of choice. What do you advise?](https://www.who.int/emergencies/diseases/novel-coronavirus-2019/question-and-answers-hub/q-a-detail/coronavirus-disease-covid-19-contraception-and-family-planning)**

If you cannot access your contraceptive method of choice – perhaps because it requires a prescription, or because it can only be given to you by a health worker – consider using condoms, fertility awareness-based methods, lactational amenorrhea (if you are exclusively breastfeeding), or other contraceptive methods that are recommended for self-care in your country.

Depending on the situation in your country, methods recommended for self-care could include the pill or mini-pill, emergency contraception pills, and DMPA-SC (Sayana Press®).

108、**[What is the best contraceptive method to use during the COVID-19 pandemic?](https://www.who.int/emergencies/diseases/novel-coronavirus-2019/question-and-answers-hub/q-a-detail/coronavirus-disease-covid-19-contraception-and-family-planning)**

All modern methods of contraception help to prevent pregnancy. Women and their partners can choose any modern contraceptive method that is acceptable to and safe for them. The best method of contraception is the one that works well for you.

There is a wide variety of modern methods, one of which may suit you best.  For more information see [here](https://www.who.int/news-room/fact-sheets/detail/family-planning-contraception).

Condoms, when they are used consistently and correctly, are the only method of contraception that help to prevent unintended pregnancy and protect against sexually transmitted infections, including HIV.  They can be used together with other methods of contraception to protect against both unintended pregnancy and sexually transmitted infections.

Emergency contraceptive pills can prevent up to 95% of pregnancies when taken within 5 days after intercourse, and they can be taken by anyone with or without a health condition.

109、**[I want to change my contraceptive method – is this possible?](https://www.who.int/emergencies/diseases/novel-coronavirus-2019/question-and-answers-hub/q-a-detail/coronavirus-disease-covid-19-contraception-and-family-planning)**

Yes. It may be difficult however, to access all the methods of contraception that are normally available in your country due to restrictions on movement, lack of supply, as well as increased demands on health providers and services.If you are experiencing side effects or desire urgent removal for other reasons, contact a provider to find out what options suit you best, and which are available and feasible.

If you have a pre-existing health condition, consult a provider to find out what options suit you best, and which are available and feasible. Seek advice and information from your health provider and consider using methods that do not have medical restrictions like minipills, condoms, fertility awareness-based methods, diaphragm, spermicides or lactational amenorrhea if you are exclusively breastfeeding.

110、**[I want to remove or replace my implant or IUD – can I do this during COVID-19 pandemic?](https://www.who.int/emergencies/diseases/novel-coronavirus-2019/question-and-answers-hub/q-a-detail/coronavirus-disease-covid-19-contraception-and-family-planning)**

Removal of long acting methods such as implants or IUDs, after the recommended period of use (and routine follow up appointments) may not be prioritized by your country’s health system during this health emergency. Seek advice from your health provider.

If, due to restrictions on movement due to the COVID-19 pandemic you cannot have your long acting method removed straight away, it is important to use another method of contraception to avoid pregnancy at this time.

There are no medical problems caused by delaying removal of long acting methods such as implants or IUDs. Do not try to remove the contraception method yourself; wait until you are able to access health care from a trained provider.

111、**[Why is providing contraception/ family planning, as well as family planning services and information, important during the COVID-19 pandemic?](https://www.who.int/emergencies/diseases/novel-coronavirus-2019/question-and-answers-hub/q-a-detail/coronavirus-disease-covid-19-contraception-and-family-planning)**

Contraception and family planning information and services are life-saving and important at all times. Sexual activity does not cease with the COVID-19 pandemic, it is therefore crucial to ensure that people are able to access rights-based services and information to initiate and / or continue use of contraception.

By preventing unintended pregnancies, contraception helps to protect girls and women from the negative health consequences of unintended pregnancies, which can save their lives. Contraception reduces the need for abortion, meaning that women and girls are less at risk of unsafe abortion, which again can be lifesaving.

Condoms, when used consistently and correctly, help to prevent both unintended pregnancies and sexually transmitted infections (including HIV).

In addition, by preventing the negative health consequences associated with unintended pregnancies, unsafe abortion and sexually transmitted infections (including HIV), contraception can help alleviate unnecessary additional pressure on already-stretched health systems which are working hard to address COVID-19.

112、**[I am a policy maker. What can I do to make sure people can access contraception and family planning information and services?](https://www.who.int/emergencies/diseases/novel-coronavirus-2019/question-and-answers-hub/q-a-detail/coronavirus-disease-covid-19-contraception-and-family-planning)**

- Plan and develop innovative strategies to ensure as many eligible people as possible can access information and contraception during this period.
- Increase use of mobile phones and digital technologies to help people make decisions about which contraceptive methods to use, and how they can be accessed.
- Enable health care workers to provide contraceptive information and services as per national guidelines to the full extent possible. This is particularly important where pregnancy poses a high risk to health.
- Expand availability of contraceptive services (including both information and methods) through places other than healthcare facilities, such as pharmacies, drug shops, online platforms and other outlets.  This can be with or without prescription depending on national guidelines and contraceptive method.
- Relax restrictions on the number of repeat issues of prescription-only hormonal contraceptives that can be issued.
- Ensure access to emergency post-coital contraception, including consideration of over the counter provision.
- Enable access to contraception for women and girls in the immediate post-partum and post abortion periods when they may access health services.

113、**[I am a programme manager. What can I do to help people access contraception and family planning information and services?](https://www.who.int/emergencies/diseases/novel-coronavirus-2019/question-and-answers-hub/q-a-detail/coronavirus-disease-covid-19-contraception-and-family-planning)**

- Increase use of telehealth for counselling and sharing of messages related to safe and effective use of contraception and for selection and initiation of contraceptives.
- Ensure adequate inventory to avoid potential stock outs at all levels of the health system.
- Prepare advisories for users on how they can access contraceptive information, services and supplies.
- Monitor contraceptive consumption in your area to identify any potential pitfall and shortage
- Increase availability and access to the contraceptives which can be used by the client without service provider support.

114、**[I am a healthcare provider. I have heard that persons with COVID-19 may have increased risk of blood clots. How should I advise individuals who are using or want to use combined hormonal contraception?](https://www.who.int/emergencies/diseases/novel-coronavirus-2019/question-and-answers-hub/q-a-detail/coronavirus-disease-covid-19-contraception-and-family-planning)**

According to [currently available evidence](https://pubmed.ncbi.nlm.nih.gov/33211113/), most women of childbearing age with COVID-19 will likely be asymptomatic or have mild COVID-19 symptoms and should continue to take combined hormonal contraception (CHC). Here are the most common possible scenarios:

- **Women who are asymptomatic or with mild symptoms of known COVID-19:**combined hormonal contraception can be continued and is considered safe in this population. If women are concerned, their provider should explain that there is no evidence of increased risk of thrombosis among combined hormonal contraception users with mild COVID-19 symptoms. If a combined hormonal contraception user wishes to discontinue this method, she can be offered a progestin-only or non-hormonal method, if available and acceptable.

- **Women with serious symptoms****[[1]](file:///C:/Users/parkkalil/AppData/Local/Microsoft/Windows/INetCache/Content.Outlook/PF7B2OAO/Updated_QAs%20contraception%20COVID19_Final__2020_SK_MA_SK_MA_final.docx#_ftn1) of COVID-19 who are not hospitalized:**Discontinuation of combined hormonal contraception should be considered, based on a patient’s clinical symptoms of COVID-19 (e.g. immobilization for more than 7 days, difficulty breathing or shortness of breath, pneumonia). Considerations around stopping combined hormonal contraception should also take into account if the user has other medical conditions that may increase the risk of thrombosis during COVID-19. If combined hormonal contraception is discontinued, progestin-only or non-hormonal methods can be offered, if available and acceptable.

- **Women hospitalized with severe COVID-19:** Combined hormonal contraception should be discontinued during hospitalisation. The risk of thrombosis in this acutely ill population may outweigh the benefits of continuing combined hormonal contraception. Progestin-only and non-hormonal contraceptive methods can be used.

- **Women resuming contraception:**It is important thatall women who discontinue contraception during COVID-19 illness are given counselling about when to resume contraception and are provided with the method of their choice. Any new or existing medical conditions need to be considered when choosing a contraceptive method. Combined hormonal contraception can be resumed 2 weeks after any prolonged period of prolonged limited mobility (immobilization). the end of immobilization. Other methods can be started without delay.

[*Coronavirus disease (COVID-19): Corticosteroids, including dexamethasone（115-124）](https://www.who.int/emergencies/diseases/novel-coronavirus-2019/question-and-answers-hub/q-a-detail/coronavirus-disease-covid-19-dexamethasone)

115、**[I have COVID-19, should I be prescribed corticosteroids like dexamethasone?](https://www.who.int/emergencies/diseases/novel-coronavirus-2019/question-and-answers-hub/q-a-detail/coronavirus-disease-covid-19-dexamethasone)**

Corticosteroids are lifesaving medicines recommended for patients with severe or critical COVID-19*. They should be given along with other current standard of care treatments for COVID-19, which currently include oxygen and other medications.

They should not be given to patients with non-severe* COVID-19. In rare circumstances, they can be harmful to these patients’ health.

*A patient has severe COVID-19 when they have signs of pneumonia, severe respiratory distress, and their blood oxygen level is low. A patient has critical COVID-19 when they need life sustaining treatment, have acute respiratory distress syndrome, or have septic shock (evidence of injury to other organs). For more information, see [Guideline Clinical management of COVID-19 patients: living guideline, 23 November 2021 (magicapp.org)](https://files.magicapp.org/guideline/0a6099d8-72d5-4d1d-9438-ecfb7417a16b/published_guideline_5852-3_0.pdf).

116、**[What are corticosteroids and how do they work against COVID-19?](https://www.who.int/emergencies/diseases/novel-coronavirus-2019/question-and-answers-hub/q-a-detail/coronavirus-disease-covid-19-dexamethasone)**

Corticosteroids, including dexamethasone, have anti-inflammatory and immunosuppressant qualities that are used to treat a wide range of conditions. Patients with severe or critical COVID-19 develop an overstimulation of the immune system, which can be very harmful to their health. Corticosteroids reduce this overstimulation, which helps balance the immune system responses so that they are effective and do not cause damage to the person themselves.

117、**[What is the benefit of corticosteroid treatment?](https://www.who.int/emergencies/diseases/novel-coronavirus-2019/question-and-answers-hub/q-a-detail/coronavirus-disease-covid-19-dexamethasone)**

Current evidence comes from the combined results of 8 clinical trials, utilizing data from 7000 patients with low oxygen levels, which show that dexamethasone (or other corticosteroids) reduces COVID-19 deaths. Data from these studies show that deaths are reduced by 20% when patients with severe COVID-19 are given corticosteroid treatment during their illness. There is also a benefit in reducing the need for ventilation (putting patients on a machine to help with breathing).

118、**[How are corticosteroids administered and what is the dosage?](https://www.who.int/emergencies/diseases/novel-coronavirus-2019/question-and-answers-hub/q-a-detail/coronavirus-disease-covid-19-dexamethasone)**

Corticosteroids are given as a pill or via injection, depending on formulation

Patients with severe or critical COVID-19 should be given low dose corticosteroids for 7-10 days. The daily dose depends on the corticosteroid being used.

119、**[Are corticosteroids suitable for anyone with COVID-19?](https://www.who.int/emergencies/diseases/novel-coronavirus-2019/question-and-answers-hub/q-a-detail/coronavirus-disease-covid-19-dexamethasone)**

WHO recommends that only patients who have severe or critical COVID-19 receive corticosteroids.

120、**[Is dexamethasone prequalified by WHO?](https://www.who.int/emergencies/diseases/novel-coronavirus-2019/question-and-answers-hub/q-a-detail/coronavirus-disease-covid-19-dexamethasone)**

Yes, WHO has prequalified dexamethasone solution for injection. It is produced by 3 manufacturers for use in treating COVID-19 illness. Prequalification means that the drug has been assessed by WHO and deemed safe and effective in treating COVID-19 illness.

121、**[Do patients receiving corticosteroids need monitoring?](https://www.who.int/emergencies/diseases/novel-coronavirus-2019/question-and-answers-hub/q-a-detail/coronavirus-disease-covid-19-dexamethasone)**

WHO recommends that all patients, even those without diabetes, should be monitored when taking corticosteroids. This is because corticosteroids can increase blood glucose (sugar) levels temporarily. High blood glucose levels caused by corticosteroids usually only require monitoring until the steroids are stopped.

Certain patients should be monitored closely when receiving corticosteroids since they are at increased risk of developing complications. These include people with diabetes, cancer, open wounds following traumatic injuries, severe burns or malnourishment. Patients taking immunosuppressants/immunomodulators, those with severe immunodeficiencies and intravenous drug users should also be monitored.

122、**[How expensive are corticosteroids? Are they widely available?](https://www.who.int/emergencies/diseases/novel-coronavirus-2019/question-and-answers-hub/q-a-detail/coronavirus-disease-covid-19-dexamethasone)**

Corticosteroids are readily available at a low cost globally.

Corticosteroids are included in the WHO Model Lists of Essential Medicines. The most commonly used corticosteroid, dexamethasone is an off-patent, common supportive treatment option and is generally affordable. WHO’s 2016 and 2019 surveys of different health facilities in low- and middle-income countries indicated that dexamethasone was available to patients at a median price of US$ 0.33 per 4mg/ml injection ampoules (range: US$ 0.13–3.5).

[123、](https://www.who.int/emergencies/diseases/novel-coronavirus-2019/question-and-answers-hub/q-a-detail/coronavirus-disease-covid-19-dexamethasone)**[What are the typical side effects of corticosteroids, including dexamethasone?](https://www.who.int/emergencies/diseases/novel-coronavirus-2019/question-and-answers-hub/q-a-detail/coronavirus-disease-covid-19-dexamethasone)**

Corticosteroids including dexamethasone are generally safe. Their benefits far outweigh the risks, particularly in patients with severe forms of pneumonia. Higher blood glucose levels (hyperglycaemia) can occur, and patients should be monitored for this, particularly if they have diabetes. Treatments are usually short, and even at high doses, corticosteroids are not associated with other serious side effects.

Prolonged use (i.e., use for more than 2 weeks) may be associated with adverse events such as glaucoma, cataract, fluid retention, hypertension, psychological effects (e.g., mood swings, memory issues, confusion or irritation), weight gain, or increased risk of infections and osteoporosis.

124、**[Can corticosteroids be used in children/the elderly/pregnant?](https://www.who.int/emergencies/diseases/novel-coronavirus-2019/question-and-answers-hub/q-a-detail/coronavirus-disease-covid-19-dexamethasone)**

Yes, they can be used to treat sick children, the elderly and during pregnancy.

#### [Coronavirus disease (COVID-19): Environmental Surveillance（125-132）](https://www.who.int/emergencies/diseases/novel-coronavirus-2019/question-and-answers-hub/q-a-detail/environmental-surveillance)

125、**[Why is environmental surveillance of wastewater important during disease outbreaks?](https://www.who.int/emergencies/diseases/novel-coronavirus-2019/question-and-answers-hub/q-a-detail/environmental-surveillance)**

Testing wastewater has a long history of use in public health. Environmental surveillance is already used to detect poliovirus in high risk settings, monitor antimicrobial resistance and complement surveillance for other public health programmes.

126、**[How is wastewater testing used for COVID-19?](https://www.who.int/emergencies/diseases/novel-coronavirus-2019/question-and-answers-hub/q-a-detail/environmental-surveillance)**

Wastewater testing can be used to monitor the presence of SARS-CoV-2 virus in untreated sewage.  Several countries are monitoring wastewater for SARS-CoV-2 virus, the virus that causes COVID-19, in a range of locations for different purposes. These include:

- early warning for COVID-19 cases in a community;
- detection of COVID-19 in locations with weak clinical surveillance;
- monitoring circulation of the virus during outbreaks; or
- to trigger case-finding in locations where there are or may be suspected cases, such as quarantine hotels, university campuses or prisons.

Some countries are also analysing historical wastewater samples for evidence of past SARS-CoV-2 circulation.

127、**[What does it mean when a wastewater sample tests positive for SARS-CoV-2?](https://www.who.int/emergencies/diseases/novel-coronavirus-2019/question-and-answers-hub/q-a-detail/environmental-surveillance)**

Molecular tests such as polymerase chain reaction (PCR) tests can detect the presence of the SARS-CoV-2 virus via fragments of genetic material (RNA) in wastewater. Finding this RNA in wastewater means that one or more people in the community likely excreted virus through urine, faeces or by coughing or sneezing. The RNA suggests that the virus was in the community at the time it was excreted. However, the wastewater test does not identify who was or is infected or indicate if the persons excreting the virus are still infectious to others.

128、**[Can I get sick with COVID-19 through contact with urine, faeces or sewage?](https://www.who.int/emergencies/diseases/novel-coronavirus-2019/question-and-answers-hub/q-a-detail/environmental-surveillance)**

While fragments of SARS-CoV-2 genetic material (RNA) have been detected in the urine or faeces of some patients, there have been no reports to date of transmission of COVID-19 through urine or faeces. It remains important to protect yourself with personal protective equipment whenever contact with bodily fluids may occur and to clean your hands frequently

Infectious SARS-CoV-2 has also not been detected in wastewater, suggesting transmission through contact or contamination with sewage is highly unlikely.

Guidance for water, sanitation, hygiene and waste management can be found here.

129、**[Can I get sick with COVID-19 from swimming in the sea, rivers, lakes or swimming pools?](https://www.who.int/emergencies/diseases/novel-coronavirus-2019/question-and-answers-hub/q-a-detail/environmental-surveillance)**

Available evidence suggests that SARS-CoV-2 does not spread from water. Natural bodies of water and swimming pools do not appear to pose a risk for COVID-19 transmission even if polluted by faeces or wastewater. Pools are usually treated regularly to prevent contamination with a range of pathogens which could be present.

However, crowded beaches or swimming pools do pose a risk of spreading COVID-19 through close contact with infected people or contaminated surfaces. To reduce the risk of this transmission, clean hands frequently, stay at least 1 metre away from others in and out of the water, and wear a mask when this distancing is not possible.

130、**[Can I get sick with COVID-19 from drinking water?](https://www.who.int/emergencies/diseases/novel-coronavirus-2019/question-and-answers-hub/q-a-detail/environmental-surveillance)**

The risk coronaviruses pose to drinking water is considered to be low and SARS-CoV-2 virus has not been detected in drinking water supplies.

Drinking water treatment methods neutralize infectious pathogens present in the water. SARS-CoV-2 is an enveloped virus, which means it has a fragile fatty envelope that is easily destroyed by the treatment process. Therefore, treated drinking water does not pose a risk for COVID-19.

131、**[Can wastewater testing be conducted in low-resource settings?](https://www.who.int/emergencies/diseases/novel-coronavirus-2019/question-and-answers-hub/q-a-detail/environmental-surveillance)**

Putting in place wastewater testing requires a good understanding of how environmental surveillance could add value in monitoring the spread of disease. The laboratory capacity needed to test wastewater samples may be limited in some settings. Clinical testing and epidemiological surveillance are higher priority strategies, as they lead directly to public health measures that can reduce transmission. Water, sanitation and hygiene (WASH) activities are also top public health priorities, such as ensuring access to hand washing facilities for all. The cost-benefit of wastewater testing relative to other public health measures should be carefully evaluated.

132、**[Does WHO recommend environmental surveillance of wastewater for COVID-19?](https://www.who.int/emergencies/diseases/novel-coronavirus-2019/question-and-answers-hub/q-a-detail/environmental-surveillance)**

At present more evidence is needed regarding use of environmental surveillance for COVID-19. Wastewater monitoring may be considered as an optional and complementary approach to COVID-19 surveillance in addition to more standard clinical surveillance.  Scientists and public health authorities continue to assess effectiveness and validate testing methods in different settings. WHO encourages efforts to continue to explore potential uses of wastewater testing and recognizes its potential as an emerging tool for improving control of COVID-19.

#### [Coronavirus disease (COVID-19): Ethics, resource allocation and priority setting（133-138）](https://www.who.int/emergencies/diseases/novel-coronavirus-2019/question-and-answers-hub/q-a-detail/coronavirus-disease-covid-19-ethics-resource-allocation-and-priority-setting)

133、**[Can I adapt previous frameworks for pandemic influenza to guide resource allocation for COVID-19?](https://www.who.int/emergencies/diseases/novel-coronavirus-2019/question-and-answers-hub/q-a-detail/coronavirus-disease-covid-19-ethics-resource-allocation-and-priority-setting)**

Many ethical frameworks have been produced for resource allocation, some of which have been included in pandemic plans. Those frameworks provide useful guidance in the current scenario. Yet, when applying them, we must take into account the type of health care resource, the context, and the stage of the pandemic. That is, while the ethical principles that apply to resource allocation might be the same in different pandemics, they can lead to different decisions, given contextual circumstances. For example, this pandemic appears to significantly impact older adults (those 60 years of age or older), and such characteristics are relevant to shaping priorities for the allocation of resources during COVID-19. As a result, it may be inappropriate to use critical care triage guidelines that have age cut-offs that deprioritize or exclude those aged over 60 years.

When applying ethical guidelines for resource allocation, we should consider the extent to which resources are overwhelmed in the current context. It would be inappropriate, for instance, to exclude population groups from being allocated a resource (for example, ventilators) at the outset of a pandemic when capacity remains. When resources are scarce, though – when there is an insufficient supply to meet everyone’s needs – resource allocation should be guided by well established, broadly applicable ethical principles, unless there are characteristics of the outbreak that justify different courses of action. Irrelevant characteristics of populations within countries, such as ethnicity, race or creed, should play no role in any resource allocation in any pandemic. This reflects our commitment to treating people with equal respect.

134、**[Are the ethical considerations the same for all medical countermeasures, including therapeutics, vaccines and personal protective equipment (PPE)?](https://www.who.int/emergencies/diseases/novel-coronavirus-2019/question-and-answers-hub/q-a-detail/coronavirus-disease-covid-19-ethics-resource-allocation-and-priority-setting)**

Generally, the considerations may be different. The allocation of different resources may find ethical justifications in different principles or values. For instance, once a novel vaccine is found to be safe and effective, to prioritize those at highest risk, as well as populations like health care workers who may be more likely to serve as vectors for transmission, is justified.

Within those subgroups, some suggest that a lottery-based (i.e., random) allocation may be justified, given that resources will be limited and we can assume nearly equal benefits will be derived from any recipient within that group. This is not the case, however, for other resources such as ventilators, where some individuals may derive significantly more benefit than others

135、**[What is the basis for deciding who should have priority access to scarce resources?](https://www.who.int/emergencies/diseases/novel-coronavirus-2019/question-and-answers-hub/q-a-detail/coronavirus-disease-covid-19-ethics-resource-allocation-and-priority-setting)**

The ethical basis for deciding which individuals or groups might be prioritized, including the principles to be applied, are presented in the below table:

### Ethical considerations when deciding who to prioritize

| **Principle** | **Description** | **Practice implication** |
| --- | --- | --- |
| Equality | Each person’s interest should count equally unless there are good reasons that justify the differential prioritization of resources.  Irrelevant characteristics of individuals, such as race, ethnicity, creed, ability or gender, should not serve arbitrarily as the basis for the differential allocation of resources.  This principle can be used to justify the allocation of resources by a lottery – that is, randomly by chance – or by a system of first come, first served. | May be most appropriate to guide the allocation of scarce resources among individuals or populations who can be expected to derive the same benefit from the resource, for example, vaccines among high-risk populations, or ventilators among those with similar clinical indicators for benefit. |
| Best outcomes (utility) | This principle can be used to justify the allocation of resources according to their capacity to do the most good or minimize the most harm, for example, using available resources to save the most lives possible. | May be most appropriate to guide the allocation of scarce resources that confer substantially different benefits to different individuals, for example, ventilators to those expected to derive the most benefit. |
| Prioritize the worst off | This principle can be used to justify the allocation of resources to those in greatest medical need or those most at risk. | May be most appropriate to guide the allocation of resources that are designed or intended to protect those at risk, for example, PPE for health care workers, vaccines for those most at risk of infection and severe illness, or those most in need, as in the case of provision of drugs in short supply to those needing them most urgently. |
| Prioritize those tasked with helping others | This principle can be used to justify the allocation of resources to those who have certain skills or talents that can save many other people, or because something is owed to them on account of their participation in helping others. | May be most appropriate to guide the allocation of resources to health care workers, first responders, etc. |

Allocation principles may be relevant or justified at different stages of resource scarcity (from less scarcity to more scarcity). For example, where little scarcity exists, the allocation of resources such as ventilators may be most justified by the principle of first come, first served (which promotes the value of equality). When those resources become increasingly scarce, their allocation may be justified according to a principle that prioritizes those most in need. With even greater scarcity, a principle that aims to maximize benefit from the resource may be most justified. At each stage, allocation should aim to promote equality – that is, first come, first served, or random allocation, when no relevant factors distinguish individuals within a particular scheme of allocation (for example, among those with similar needs, or among those who can be expected to benefit similarly from the resources, or among those that are at similar levels of risk).

In addition, multiple principles may be combined within an allocation scheme. For example, an allocation scheme for PPE might find its justification in a principle prioritizing those most at risk as well as a principle prioritizing those tasked with helping others, which would support priority allocation of PPE to health care workers.

136、**[How should decision-makers make considered ethical judgements about these matters, given the likelihood that there will not be enough of a resource? Who should be involved in decision-making on scarce resources?](https://www.who.int/emergencies/diseases/novel-coronavirus-2019/question-and-answers-hub/q-a-detail/coronavirus-disease-covid-19-ethics-resource-allocation-and-priority-setting)**

For most decisions, multiple ethical values and principles will be relevant to deliberations about how to allocate resources. This is likely to generate some disagreement, because different people may weigh the values differently. Some may prioritize equality while others might put more emphasis on best outcomes or prioritization of the worst-off. For this reason, it is imperative that the different values be weighed and applied to specific allocation issues using a fair process.

A fair process for allocating scarce resources must promote certain ethical values:

- **Transparency.** In a transparent process, the decisions and their justifications should be made public. This means that the population should be informed about the criteria guiding the decisions.
- **Inclusiveness.** Those affected by allocation decisions – including individuals, communities or countries – should be able to exert at least some influence over the decision-making process as well as the decision itself. This also means that decisions 4 should be open to challenge and potentially revisable, perhaps through an appeal process.
- **Consistency.** Decisions should be consistent so that all persons in the same categories are treated in the same way. This means that favouritism towards one’s own family, religious or political compatriots, or otherwise, is not appropriate. All forms of corruption that are at variance with this principle should be challenged and condemned.
- **Accountability.** Those making decisions about allocation must be accountable for those decisions – that is, they should justify their decisions and be held responsible for them. A fair process means that allocation decisions should not be made by individuals, by individual pharmaceutical companies, or, in the case of allocation between countries, by a single country. Resources such as vaccines and therapies should not be stockpiled outside the system of fair allocation.

The fair allocation of resources is one that is valuable in itself precisely because it is fair. However, it may also be valuable because a fair system engenders solidarity and trust, which are vital to the successful and sustained collective response necessary for dealing effectively with any outbreak.

137、**[What are the key ethical considerations that governments, vaccine manufacturers and funders should take into account to ensure a fair distribution of vaccines globally?](https://www.who.int/emergencies/diseases/novel-coronavirus-2019/question-and-answers-hub/q-a-detail/coronavirus-disease-covid-19-ethics-resource-allocation-and-priority-setting)**

Countries are equally vulnerable to COVID-19 and have a shared responsibility, grounded in solidarity, to collaborate globally to mitigate the outbreak. Each government has special obligations to its own citizens, but the fair allocation of vaccines globally requires us not to simply appeal to self-interest, claims of resource ownership and the prioritization of compatriots. Vaccines should be allocated in a way that prioritizes those who fall into the categories presented in the below table. (Where individuals or populations fall into multiple categories, they should receive even higher priority.)

### Priority populations, and rationale for prioritization

| **Priority population** | **Rationale for prioritization** |
| --- | --- |
| Those at greatest risk of becoming infected and seriously ill | Maximize benefit of vaccine |
| Those who, if vaccinated, would prevent the greatest spread of the virus | Maximize benefit of vaccine |
| Those who have volunteered to participate in research aimed at developing the vaccine | Reciprocal obligation to those who were voluntarily put at risk to aid in this effort |

Those falling into each category may change over time.

138、**[What conclusions can we draw about the fair allocation of scarce resources within countries during the COVID-19 outbreak?](https://www.who.int/emergencies/diseases/novel-coronavirus-2019/question-and-answers-hub/q-a-detail/coronavirus-disease-covid-19-ethics-resource-allocation-and-priority-setting)**

1. Health care workers (caring for patients) and first responders can justifiably be prioritized when allocating some resources because of their contribution to the health and well-being of the community. Their health helps preserve the health of others.
2. Participants of research aimed at developing vaccines, therapies or other critical resources should receive some priority in receiving those resources because they have also helped save others by their participation. This is not an absolute priority – for example, it should not take precedence over giving priority to those most at risk in the case of resources such as vaccines.
3. While the principle of first come, first served is often applied when allocating resources in health care settings, it is rarely appropriate in an emergency. In practice, it is very likely to favour certain groups, such as those closest to a distribution centre, those with access to better information, or those who are most well-off.
4. Younger populations appear to be at lower risk in the COVID-19 context. Consequently, the principle of youngest first should have low priority for vaccine, but perhaps may have more weight if they do become sick and need critical care resources.
5. The allocation of different resources may find ethical justification in different principles or values. For instance, if a novel vaccine is found to be safe and effective, a lottery-based allocation may be justified among those as highest risk, the old and those with co-morbidities, if they outnumber available vaccines.
6. Maximizing utility should be balanced with the principle of priority to the worst-off: centralizing the availability of resources in larger centres may extend their benefits to more people, but may exclude isolated populations and challenge our concern for those at highest risk.

#### [Coronavirus disease (COVID-19): Food businesses（139-152）](https://www.who.int/emergencies/diseases/novel-coronavirus-2019/question-and-answers-hub/q-a-detail/coronavirus-disease-covid-19-food-businesses)

139、**[How can food businesses remain safe from virus contamination?](https://www.who.int/emergencies/diseases/novel-coronavirus-2019/question-and-answers-hub/q-a-detail/coronavirus-disease-covid-19-food-businesses)**

The main priority is to keep the virus out of the food environment. Several key measures are required including upgrading of cleaning and sanitation measures, disinfecting surfaces and high-touch points, educating staff on the virus and how to protect themselves and others, reinforcing protocols such as physical distancing, hand washing, and improved security with people staying in their vehicles/sanitizing hands when handing out documents and other material.

140、**[Should grocery store workers wear gloves? Masks?](https://www.who.int/emergencies/diseases/novel-coronavirus-2019/question-and-answers-hub/q-a-detail/coronavirus-disease-covid-19-food-businesses)**

**Disposable Gloves**

Gloves may be used by food workers but must be changed frequently and hands must be washed between glove changes and when gloves are removed. Gloves must be changed after carrying out non-food related activities such as opening/closing doors by hand, and emptying bins. Food workers should be aware that wearing gloves can allow bacteria to build up on the surface of the hands, so hand washing is extremely important when gloves are removed to avoid subsequent contamination of food. Food workers should not touch their mouth, nose and eyes when wearing gloves.

Disposable gloves can give a false sense of safety and should not be used in the food work environment as a substitute for hand washing. The COVID-19 virus can contaminate disposable gloves in the same way it can get onto workers hands and contact surfaces. Removal of disposable gloves can lead to contamination of hands. Wearing disposable gloves can give you a false sense of security and may result in staff not washing hands as frequently as required.

Handwashing is a greater protective barrier to infection than wearing of disposable gloves. Food businesses need to ensure adequate sanitary facilities are provided and ensure food workers thoroughly and frequently wash their hands. Soap and water is adequate for hand washing. Hand sanitisers can be used as an additional measure but should not replace hand washing.

**Wearing of Face Masks**

WHO [advice on the use of masks in the context of COVID-19](https://www.who.int/publications-detail-redirect/advice-on-the-use-of-masks-in-the-community-during-home-care-and-in-healthcare-settings-in-the-context-of-the-novel-coronavirus-(2019-ncov)-outbreak?fbclid=IwAR0U3P3k2Qw8JsFR0FvNR15yh8ze9cd3tFKGd1ukfJ_P4C8jH2vBiyDSA-I) should be followed. Face Masks do not have to be routinely used by food workers to protect against transmission of COVID-19. However, for some food processing activities, such as working in abattoirs or handling cooked, ready-to-eat foods, wearing face masks is a usual practice.

WHO recommends that face masks should be used if a person is sick with symptoms of COVID-19 (especially coughing) or looking after someone with suspected or confirmed COVID-19.

However, a food worker who is sick or who has symptoms of COVID-19 should not be allowed to work in grocery stores or other food businesses and should be excluded from work until free of symptoms or deemed fit for work by a medical doctor.

141、**[What is the protocol when an employee working in a food business becomes ill with COVID-19?](https://www.who.int/emergencies/diseases/novel-coronavirus-2019/question-and-answers-hub/q-a-detail/coronavirus-disease-covid-19-food-businesses)**

Staff who are feeling unwell should not report to work and should seek medical advice. However, in the event that a food worker becomes unwell in the workplace with typical symptoms of COVID-19, they should be removed to an area away from other people. If possible, find a room or area where they can be isolated behind a closed door, such as a staff office. If it is possible to open a window, do so for ventilation. Arrangements should be made for the unwell employee to be removed quickly from the food premise.

The employee who is unwell should follow national guidelines for reporting cases/suspect cases of COVID-19. Whilst they wait for medical advice or to be sent home, they should avoid any contact with other employees. They should avoid touching people, surfaces and objects and be advised to cover their mouth and nose with a disposable tissue when they cough or sneeze and put the tissue in a bag or pocket and then dispose of the tissue in a bin. If they do not have any tissues available, they should cough and sneeze into the crook of their elbow. If they need to go to the bathroom whilst waiting for medical assistance, they should use a separate bathroom, if available.

All surfaces that the infected employee has come into contact with must be cleaned. Alcohol based sanitizers/surface disinfectants should be used for cleaning purposes. In general, alcohol-based disinfectants (ethanol, propan-2-ol, propan-1-ol) have been shown to significantly reduce infectivity of enveloped viruses like COVID-19 virus, in concentrations of 70-80%. Common disinfectants with active ingredients based on quaternary ammonium compounds (QUATS) and chlorine would also have virucidal properties. All staff should wash their hands thoroughly for 20 seconds with soap and water after any contact with someone who is unwell with symptoms consistent with coronavirus infection.

If an employee is confirmed as a case of COVID-19 it will be necessary to notify all close contacts of the infected employee so they too can take measures to minimise further risk of spread. WHO definitions of a contact of a COVID-19 case can be found  [here](https://www.who.int/publications-detail-redirect/global-surveillance-for-human-infection-with-novel-coronavirus-(2019-ncov)). Examples of contacts in the food businesses could include any employee who was in face-to-face or physical (i.e., touching) contact; any employee who was within 1 meter with the confirmed case; anyone who has cleaned up any bodily fluids without adequate PPE (e.g. gloves, overalls, protective clothing); employees in the same working team or workgroup as the confirmed case, and any employee living in the same household as a confirmed case.

[WHO recommends](https://www.who.int/publications-detail-redirect/considerations-for-quarantine-of-individuals-in-the-context-of-containment-for-coronavirus-disease-(covid-19)) that contacts be quarantined for 14 days from the last point of exposure to the confirmed case.22 At a minimum, staff who have had close contact with the infected employee should be asked to stay at home for 14 days from the last time they had contact with the confirmed case and practice physical distancing. If they become unwell at any time within their 14-day isolation period and they test positive for COVID-19, they will become a confirmed case, and should be managed as such.

Staff who have not had close contact with the original confirmed case should continue taking the usual precautions and attend work as usual. Organising employees into small teams or workgroups will help to minimise disruption to work processes in the event of an employee reporting sick with symptoms of COVID-19. Closure of the workplace is not recommended.

More detailed information can be found in [COVID-19 and food safety: guidance for food businesses](https://apps.who.int/iris/bitstream/handle/10665/331705/WHO-2019-nCoV-Food_Safety-2020.1-eng.pdf).

For more information on contacts of COVID-19 confirmed cases, see [WHO guidance on surveillance and case definitions](https://www.who.int/emergencies/diseases/novel-coronavirus-2019/technical-guidance/surveillance-and-case-definitions).

142、**[When can an employee return to work following illness? Are temperature checks appropriate?](https://www.who.int/emergencies/diseases/novel-coronavirus-2019/question-and-answers-hub/q-a-detail/coronavirus-disease-covid-19-food-businesses)**

A return to work policy for staff who have been infected and recovered from COVID-19 should be in place. WHO recommends that a confirmed patient could be released from isolation once their symptoms resolve and they have two negative PCR tests at least 24 hours apart.  If testing is not possible, [WHO recommends](https://www.who.int/publications-detail-redirect/home-care-for-patients-with-suspected-novel-coronavirus-(ncov)-infection-presenting-with-mild-symptoms-and-management-of-contacts) that a confirmed patient can be released from isolation 14 days after symptoms resolve.

Taking the temperature of food workers is not recommended. Fever is only one of the symptoms of COVID-19 and absence of fever alone is not a reliable indicator of wellness. As part of a food business ‘fitness to work’ guidelines, staff should report to management if they are sick with typical COVID-19 symptoms, particularly fever.

143、**[What specific precautions do food workers need to take?](https://www.who.int/emergencies/diseases/novel-coronavirus-2019/question-and-answers-hub/q-a-detail/coronavirus-disease-covid-19-food-businesses)**

Physical distancing, good personal hygiene with frequent hand washing, and application of general food hygiene measures are the most important precautions food workers should adopt. Physical distancing is very important to help slow the spread of coronavirus. This is achieved by minimising contact between potentially infected individuals and healthy individuals. Frequent hand washing with soap and water and use of hand sanitizers are also important in reducing the risk of transmission. All food businesses should follow the physical distancing and hand washing [guidance of WHO](https://www.who.int/news-room/questions-and-answers/item/q-a-coronaviruses).

The [Codex Alimentarius Commission](http://www.fao.org/fao-who-codexalimentarius/home/en/) has adopted several practical guidelines on how to apply and implement best practices to ensure food hygiene ([Codex General Principles of Food Hygiene, CXC 1- 1969](http://www.fao.org/fao-who-codexalimentarius/sh-proxy/en/?lnk=1&url=https%253A%252F%252Fworkspace.fao.org%252Fsites%252Fcodex%252FStandards%252FCXC%2B1-1969%252FCXP_001e.pdf)), handle meats ([Codex Code of Hygienic Practice for Meat, CXC 58 – 2005](http://www.fao.org/fao-who-codexalimentarius/sh-proxy/en/?lnk=1&url=https%253A%252F%252Fworkspace.fao.org%252Fsites%252Fcodex%252FStandards%252FCXC%2B58-2005%252FCXP_058e.pdf)), and control viruses in foods ([Guidelines for the Application of General Principles of Food Hygiene to the Control of Viruses in Food (CAC/GL 79-2012](http://www.fao.org/fao-who-codexalimentarius/sh-proxy/en/?lnk=1&url=https%253A%252F%252Fworkspace.fao.org%252Fsites%252Fcodex%252FStandards%252FCXG%2B79-2012%252FCXG_079e.pdf)). Enhanced food safety practices at this time, such as those recommended in the Codex documents mentioned, will reduce the likelihood of contamination of foods with any pathogen and help lowering the public health burden caused by already established foodborne infections, reducing the stress on an already overburdened public health system.

144、**[How should employees maintain safe distance from one another during food production and processing?](https://www.who.int/emergencies/diseases/novel-coronavirus-2019/question-and-answers-hub/q-a-detail/coronavirus-disease-covid-19-food-businesses)**

WHO guidelines are to maintain at least 1 metre (3 feet) between fellow workers. Where the food production environment makes it difficult to do so, employers need to consider what measures to put in place to protect employees. Examples of practical measures to adhere to physical distancing guidance in the food processing environment are to:

- stagger workstations on either side of processing lines so that food workers are not facing one another,
- provide personal protection equipment (PPE) such as face masks, hair nets, disposable gloves, clean overalls and slip reduction work shoes for staff. The use of PPE would be routine in high risk areas of food premises that produce ready-to-eat and cooked foods. When staff are dressed in PPE it is possible to reduce distance between workers,
- space out workstations, which may require reduction in the speed of production lines,
- limit the number of staff in a food preparation area at any one time,
- organise staff into working groups or teams to facilitate reduced interaction between groups, including during change of work shifts.

145、**[How should baked goods and fresh produce be displayed in a food market/grocery store?](https://www.who.int/emergencies/diseases/novel-coronavirus-2019/question-and-answers-hub/q-a-detail/coronavirus-disease-covid-19-food-businesses)**

It is important to maintain good hygiene practices around open food displays, with ready-to-eat food products such as salad bars, fresh produce displays and bakery products. Consumers should always be advised to wash fruits and vegetables with potable water prior to consumption. Both customers and staff should strictly observe good personal hygiene practices at all times around open food areas.

In order to hygienically manage open food displays and to avoid the transmission of COVID-19 through surface contact, food retailers should:

- Maintain frequent washing and sanitising of all food contact surfaces and utensils;
- Require food service workers to frequently wash hands, and, if using gloves, these must be changed;
- Require food service workers to frequently clean and sanitise counters, serving utensils and condiment containers;
- Make available hand sanitiser for consumers on their way in and out of the food premises;
- Should consider not openly displaying or selling unwrapped bakery products from self-service counters. Bakery products on open, self-service displays in retail stores should be placed in plastic/cellophane or paper packaging. Where loose bakery products are displayed in retail stores, these should be placed in plexiglass display cabinets and placed in bags using tongs when customers are served.
- Ready-to-eat salads available in retail for self-serve, should also be placed behind plexiglass display cabinets and serving utensils should be frequently sanitized.

146、**[How should shopping trolleys or carts be disinfected?](https://www.who.int/emergencies/diseases/novel-coronavirus-2019/question-and-answers-hub/q-a-detail/coronavirus-disease-covid-19-food-businesses)**

The handles of shopping trolleys or carts should be frequently cleaned using either alcohol-based sanitizers or chlorine-based disinfectants (sodium hypochlorite). Sanitizers, paper towels and trash bins should be placed outside the retail premises close to the trolley park for customers to use.

147、**[What is the most appropriate sanitizer to use on surfaces in a food production environment?](https://www.who.int/emergencies/diseases/novel-coronavirus-2019/question-and-answers-hub/q-a-detail/coronavirus-disease-covid-19-food-businesses)**

In general, alcohol-based disinfectants (ethanol, propan-2-ol, propan-1-ol) have been shown to significantly reduce infectivity of enveloped viruses like SARS-CoV-2, in concentrations of 70-80% with one-minute exposure time. Chlorine-based disinfectants (sodium hypochlorite) are effective for surface decontamination, as are disinfectants with active ingredients based on quaternary ammonium compounds (QUATS).

148、**[Could the virus be transmitted from humans to food animals or vice versa?](https://www.who.int/emergencies/diseases/novel-coronavirus-2019/question-and-answers-hub/q-a-detail/coronavirus-disease-covid-19-food-businesses)**

Currently, there is no evidence to suggest that food animals could be a possible route for transmission of COVID-19 to humans or that food animals can become infected by humans. Studies are underway to better understand the susceptibility of different animal species to the COVID-19 virus and to assess infection dynamics in susceptible animal species. Additional information can be found on the website of the [World Organisation for Animal Health (OIE)](https://www.oie.int/en/scientific-expertise/specific-information-and-recommendations/questions-and-answers-on-2019novel-coronavirus/).

149、**[What is known about various conditions affecting survivability of the virus?](https://www.who.int/emergencies/diseases/novel-coronavirus-2019/question-and-answers-hub/q-a-detail/coronavirus-disease-covid-19-food-businesses)**

 Very little is known about how the SARS-CoV-2 virus survives outside the human body. Probably the most up-to-date summary of scientific information we have on survival in the environment is from the European Centre for Disease control (ECDC) and the following is extracted from their recent [technical report](https://www.ecdc.europa.eu/en/publications-data/disinfection-environments-covid-19):

*Recent publications have evaluated the survival of SARS-CoV-2 on different surfaces. According to van Doremalen et al., the environmental stability of SARS-CoV-2 is up to three hours in the air post-aerosolization, up to four hours on copper, up to 24 hours on cardboard and up to two to three days on plastic and stainless steel, albeit with significantly decreased titres [3]. These findings are comparable with results obtained for environmental stability of SARS-CoV-1. These findings resulted from experiments in a controlled environment and should be interpreted with caution in the real-life environment. Moreover, different levels of environmental contamination have been detected in rooms of COVID-19 patients, ranging from 1 out of 13 to 13 out of 15 samples testing positive for SARS-CoV-2 before cleaning. No air samples were positive in these studies, but one sample from an air exhaust outlet was positive indicating that virus particles may be displaced by air and deposited on surfaces [4,5]. In a study of environmental contamination in a Chinese hospital during the COVID-19 outbreak, SARS-CoV-2 was detected in environmental samples from the COVID-19 dedicated intensive care units (ICU), the COVID-19 dedicated obstetric isolation ward and the COVID-19 dedicated isolation ward. SARS-CoV-2 was also detected on objects such as the self-service printers used by patients to self-print the results of their exams, desktop keyboards and doorknobs. Virus was detected most commonly on gloves (15.4% of samples) and rarely on eye protection (1.7%) [6]. This evidence shows the presence of SARS-CoV-2 in the environment of a COVID-19 patient, therefore reinforcing the belief that fomites play a role in transmission of SARS-CoV-2; however, the relative importance of this route of transmission compared to direct exposure to respiratory droplets is still unclear.*

150、**[What measures should be taken to ensure safe transport of food during this pandemic?](https://www.who.int/emergencies/diseases/novel-coronavirus-2019/question-and-answers-hub/q-a-detail/coronavirus-disease-covid-19-food-businesses)**

The general guidelines outlined in the [Codex Code of Hygienic Practice for the Transport of Food in Bulk and Semi-Packed Food](http://www.fao.org/fao-who-codexalimentarius/sh-proxy/en/?lnk=1&url=https%253A%252F%252Fworkspace.fao.org%252Fsites%252Fcodex%252FStandards%252FCXC%2B47-2001%252FCXP_047e.pdf).[1](applewebdata://47E89BDB-0822-436A-9A78-9ADC73B7EA72#_ftn1) should be followed, in addition to ensuring that employees delivering foods are free from COVID-19, coughing/sneezing etiquette is practiced by all involved in food transport and that frequent hand washing/sanitizing is practiced and staff practice physical distancing.

See also other [Codes of Hygienic Practice for various groups of food](http://www.fao.org/fao-who-codexalimentarius/codex-texts/codes-of-practice/en/).

151、**[What measures should be taken to ensure safe water is used in food production during this pandemic?](https://www.who.int/emergencies/diseases/novel-coronavirus-2019/question-and-answers-hub/q-a-detail/coronavirus-disease-covid-19-food-businesses)**

There is no evidence that COVID-19 is transmitted by water used in food processing. The [WHO guidelines for drinking-water quality](https://www.who.int/teams/environment-climate-change-and-health/water-sanitation-and-health/water-safety-and-quality/drinking-water-quality-guidelines) should be followed.

152、**[How should food premises be cleaned/disinfected during this pandemic?](https://www.who.int/emergencies/diseases/novel-coronavirus-2019/question-and-answers-hub/q-a-detail/coronavirus-disease-covid-19-food-businesses)**

The general guidelines outlined in the [General Principles of Food Hygiene](http://www.fao.org/fao-who-codexalimentarius/sh-proxy/en/?lnk=1&url=https%253A%252F%252Fworkspace.fao.org%252Fsites%252Fcodex%252FStandards%252FCXC%2B1-1969%252FCXP_001e.pdf) for food processing and manufacture premises should be followed. If a suspected or confirmed case of COVID-19 is identified in a food premises then there is a requirement to completely clean the area with a neutral detergent, followed by decontamination of surfaces using a disinfectant effective against viruses. All surfaces that the infected employee has come into contact with must be cleaned, including all surfaces and objects which are visibly contaminated with body fluids/respiratory secretions, and all potentially contaminated high-contact areas such as toilets, door handles, telephones. Alcohol based sanitizers/surface disinfectants should be used for cleaning purposes. In general, alcohol-based disinfectants (ethanol, propan-2-ol, propan-1-ol) have been shown to significantly reduce infectivity of enveloped viruses like SARS-CoV-2, in concentrations of 70-80% with one-minute exposure time. Chlorine-based disinfectants (sodium hypochlorite) are effective for surface decontamination, as are disinfectants with active ingredients based on quaternary ammonium compounds (QUATS).

All staff should wash their hands thoroughly for 20 seconds after any contact with someone who is unwell with symptoms consistent with coronavirus infection. Staff engaged in environmental cleaning should wear PPE when performing cleaning activities, such as overalls or uniform, single-use plastic aprons, gloves and a face mask. Protective clothing (e.g. uniforms, overalls, etc.) should be frequently washed at 60°C or above.

#### [Coronavirus disease (COVID-19): Food safety and nutrition（153-167）](https://www.who.int/emergencies/diseases/novel-coronavirus-2019/question-and-answers-hub/q-a-detail/coronavirus-disease-covid-19-food-safety-and-nutrition)

153、**[Can I get COVID-19 from eating fresh foods, like fruits and vegetables?](https://www.who.int/emergencies/diseases/novel-coronavirus-2019/question-and-answers-hub/q-a-detail/coronavirus-disease-covid-19-food-safety-and-nutrition)**

There is currently no evidence that people can catch COVID-19 from food, including fruits and vegetables. Fresh fruits and vegetables are part of a healthy diet and their consumption should be encouraged.  *Read more in the*[*COVID-19 and food safety: guidance for food businesses*](https://www.who.int/publications-detail-redirect/covid-19-and-food-safety-guidance-for-food-businesses)*.*

154、**[How should I wash fruits and vegetables in the time of COVID-19?](https://www.who.int/emergencies/diseases/novel-coronavirus-2019/question-and-answers-hub/q-a-detail/coronavirus-disease-covid-19-food-safety-and-nutrition)**

Wash fruit and vegetables the same way you would in any other circumstance. Before handling them, wash your hands with soap and water. Then wash fruits and vegetables thoroughly with clean water, especially if you eat them raw.

155、**[Can the virus that causes COVID-19 live on the surface of food packaging?](https://www.who.int/emergencies/diseases/novel-coronavirus-2019/question-and-answers-hub/q-a-detail/coronavirus-disease-covid-19-food-safety-and-nutrition)**

Coronaviruses need a live animal or human host to multiply and survive and cannot multiply on the surface of food packages. It is not necessary to disinfect food packaging materials, but hands should be properly washed after handling food packages and before eating ([*see question 10 of*Questions relating to food businesses](https://web-prod.who.int/news-room/q-a-detail/questions-relating-to-food-businesses)*).*

156、**[Can the virus that causes COVID-19 be transmitted through the consumption of cooked foods, including animal products?](https://www.who.int/emergencies/diseases/novel-coronavirus-2019/question-and-answers-hub/q-a-detail/coronavirus-disease-covid-19-food-safety-and-nutrition)**

There is currently no evidence that people can catch COVID-19 from food. The virus that causes COVID-19 can be killed at temperatures similar to that of other known viruses and bacteria found in food. Foods such as meat, poultry and eggs should always be thoroughly cooked to at least 70°C. Before cooking, raw animal products should be handled with care to avoid cross-contamination with cooked foods. [The WHO 5-Keys to Safer Food](https://www.who.int/publications-detail-redirect/9789241594639)provides further information.

157、**[Is it safe to go to grocery stores and other food markets?](https://www.who.int/emergencies/diseases/novel-coronavirus-2019/question-and-answers-hub/q-a-detail/coronavirus-disease-covid-19-food-safety-and-nutrition)**

Yes, it is generally safe to go grocery shopping and to markets by following the below prevention measures:

- Clean your hands with sanitizer before entering the store.
- Cover a cough or sneeze in your bent elbow or tissue.
- Maintain at least a 1-metre distance from others, and if you can’t maintain this distance, wear a mask (many stores now require a mask).
- Once home, wash your hands thoroughly and also after handling and storing your purchased products.

There is currently no confirmed case of COVID-19 transmitted through food or food packaging.

More information on masks is available [here](https://www.who.int/emergencies/diseases/novel-coronavirus-2019/advice-for-public/when-and-how-to-use-masks).

For more recommendations on how to minimize the risk of transmission of emerging pathogens in traditional food markets, see the [WHO recommendations to reduce risk of transmission of emerging pathogens from animals to humans in live animal markets or animal product markets](https://www.who.int/emergencies/diseases/novel-coronavirus-2019/origins-of-the-virus).

158、**[Is it safe to have groceries delivered?](https://www.who.int/emergencies/diseases/novel-coronavirus-2019/question-and-answers-hub/q-a-detail/coronavirus-disease-covid-19-food-safety-and-nutrition)**

Yes, it is safe to have groceries delivered if the provider follows good personal and food hygiene practices. After accepting food/grocery deliveries, hands should be properly washed.

159、**[What is the best household disinfectant for surfaces?](https://www.who.int/emergencies/diseases/novel-coronavirus-2019/question-and-answers-hub/q-a-detail/coronavirus-disease-covid-19-food-safety-and-nutrition)**

Regular household cleaning and disinfection products will effectively eliminate the virus from household surfaces.  For cleaning and disinfecting households with suspected or confirmed COVID19, surface virucidal disinfectants, such as 0.05% sodium hypochlorite (NaClO) and products based on ethanol (at least 70%), should be used.

160、**[What foods should be consumed to support the immune system?](https://www.who.int/emergencies/diseases/novel-coronavirus-2019/question-and-answers-hub/q-a-detail/coronavirus-disease-covid-19-food-safety-and-nutrition)**

The immune system requires the support of many nutrients. It is recommended to consume a variety of foods for a healthy and balanced diet, including whole grains, legumes, vegetables, fruits, nuts and animal source foods. There is no single food that will prevent you from catching COVID-19.  For more information on a healthy diet, see the [Healthy diet fact sheet](https://www.who.int/news-room/fact-sheets/detail/healthy-diet).

161、**[Can micronutrient (vitamin and mineral) supplements prevent COVID-19 in healthy individuals or cure it in those with COVID-19 disease?](https://www.who.int/emergencies/diseases/novel-coronavirus-2019/question-and-answers-hub/q-a-detail/coronavirus-disease-covid-19-food-safety-and-nutrition)**

No. There is currently no guidance on micronutrient supplementation for the prevention of COVID-19 in healthy individuals or for the treatment of COVID-19. Micronutrients are critical for a well-functioning immune system and play a vital role in promoting health and nutritional well-being. Wherever possible, micronutrient intakes should come from a nutritionally balanced and diverse diet, including from fruits, vegetables and animal source foods.

**[162、Are vitamin D supplements needed if individuals are not exposed to sunlight due to lockdowns?](https://www.who.int/emergencies/diseases/novel-coronavirus-2019/question-and-answers-hub/q-a-detail/coronavirus-disease-covid-19-food-safety-and-nutrition)**

Vitamin D can be made in the skin by exposure to sunlight or obtained through the diet from natural sources (e.g. fatty fishes such as salmon, tuna and mackerel, fish liver oils, beef liver, cheese and egg yolks), or from vitamin D-fortified foods or vitamin D-containing supplements.

In situations where individuals’ vitamin D status is already marginal or where foods rich in vitamin D (including vitamin D-fortified foods) are not consumed, and exposure to sunlight is limited, a vitamin D supplement in doses of the recommended nutrient intakes (200-600 IU, depending on age) or according to national guidelines may be considered.

See WHO guidance on [Vitamin and mineral requirements in human nutrition](https://apps.who.int/iris/bitstream/handle/10665/42716/9241546123.pdf?ua=1).

163、**[Are there any herbal teas or herbal supplements that may help prevent or cure COVID-19?](https://www.who.int/emergencies/diseases/novel-coronavirus-2019/question-and-answers-hub/q-a-detail/coronavirus-disease-covid-19-food-safety-and-nutrition)**

No. There is currently no evidence to support the use of herbal teas or herbal supplements to prevent or cure COVID-19.

164、**[Can probiotics help prevent COVID-19?](https://www.who.int/emergencies/diseases/novel-coronavirus-2019/question-and-answers-hub/q-a-detail/coronavirus-disease-covid-19-food-safety-and-nutrition)**

No. Probiotics are live microorganisms that are generally added to foods or used as a supplement to the diet to confer a health benefit. However, there is currently no evidence to support the use of probiotics to help prevent or cure COVID-19.

165、**[Can eating ginger help prevent COVID-19?](https://www.who.int/emergencies/diseases/novel-coronavirus-2019/question-and-answers-hub/q-a-detail/coronavirus-disease-covid-19-food-safety-and-nutrition)**

No.**T**here is no evidence that eating ginger has protected people from COVID-19. However, ginger is a food that may have some antimicrobial and anti-inflammatory properties.

166、**[Can eating garlic help prevent COVID-19?](https://www.who.int/emergencies/diseases/novel-coronavirus-2019/question-and-answers-hub/q-a-detail/coronavirus-disease-covid-19-food-safety-and-nutrition)**

No. There is no evidence that eating garlic has protected people from COVID-19. However, garlic is a food that may have some antimicrobial properties.

167、**[Can adding pepper to your soup or other meals help prevent or cure COVID-19?](https://www.who.int/emergencies/diseases/novel-coronavirus-2019/question-and-answers-hub/q-a-detail/coronavirus-disease-covid-19-food-safety-and-nutrition)**

No. There is no evidence that adding hot peppers to your food can prevent or cure COVID-19.

#### [Coronavirus disease (COVID-19): Food safety authorities（168-173）](https://www.who.int/emergencies/diseases/novel-coronavirus-2019/question-and-answers-hub/q-a-detail/coronavirus-disease-covid-19-food-safety-authorities)

168、**[How should imported foods from countries with high prevalence of COVID-19 be treated?](https://www.who.int/emergencies/diseases/novel-coronavirus-2019/question-and-answers-hub/q-a-detail/coronavirus-disease-covid-19-food-safety-authorities)**

As food has not been implicated in the transmission of COVID-19, imported food should be subjected to the same import controls as before the pandemic. See [FAO guidance on risk based imported food control](http://www.fao.org/3/a-i5381e.pdf).

169、**[Do food inspectors need to wear any protective equipment?](https://www.who.int/emergencies/diseases/novel-coronavirus-2019/question-and-answers-hub/q-a-detail/coronavirus-disease-covid-19-food-safety-authorities)**

In the course of conducting a food inspection, food inspectors routinely wear protective equipment. There is no need for additional protective equipment to be used. The primary focus of the additional hygiene and sanitation measures implemented by food businesses should be on keeping the COVID-19 out of their businesses. COVID-19 virus will only enter business premises when an infected person enters, or contaminated fomites are brought into the premises. Food safety authorities should consider reducing the frequency of food inspections during this pandemic. If food inspectors continue to carry out food inspections, they will need to demonstrate that they are free from infection, they will need to practice physical distancing while in the food premises, changing clothes/shoes between inspections, washing hands before and after entering the food premises and good coughing/sneezing etiquette.

170、**[What do food inspectors need to consider when reopening a food business when the pandemic is over?](https://www.who.int/emergencies/diseases/novel-coronavirus-2019/question-and-answers-hub/q-a-detail/coronavirus-disease-covid-19-food-safety-authorities)**

Food inspectors do not need to intervene when restaurants reopen after having closed upon the advice of national governments.

171、**[What are the lab protocols for identifying the virus in food? On surfaces?](https://www.who.int/emergencies/diseases/novel-coronavirus-2019/question-and-answers-hub/q-a-detail/coronavirus-disease-covid-19-food-safety-authorities)**

As food has not been implicated in the transmission of COVID-19, testing of food or food surfaces for this virus is not recommended. Frequent cleaning of food contact surfaces with virucidal disinfectants such as 0.05% sodium hypochlorite (NaClO) or products based on ethanol (at least 70%) should be carried out. Alcohol-based disinfectants (ethanol, propan-2-ol, propan-1-ol) have been shown to significantly reduce infectivity of enveloped viruses like SARS-CoV-2, in concentrations of 70-80% with one-minute exposure time. Chlorine-based disinfectants (sodium hypochlorite) are effective for surface decontamination, as are disinfectants with active ingredients based on quaternary ammonium compounds (QUATS).

172、**[Is quarantine necessary for live animals imported from countries experiencing a large numbers of COVID-19 cases?](https://www.who.int/emergencies/diseases/novel-coronavirus-2019/question-and-answers-hub/q-a-detail/coronavirus-disease-covid-19-food-safety-authorities)**

No, food animals have not been implicated in the transmission of COVID-19 and the same import controls should apply as before this pandemic. See [Q&A from the World Organisation for Animal Health](https://www.oie.int/en/scientific-expertise/specific-information-and-recommendations/questions-and-answers-on-2019novel-coronavirus/) for more about COVID-19 and animals.

173、**[How do we ensure the food supply chain remains intact to prevent food shortages?](https://www.who.int/emergencies/diseases/novel-coronavirus-2019/question-and-answers-hub/q-a-detail/coronavirus-disease-covid-19-food-safety-authorities)**

While many businesses have introduced working from home and teleworking, these are not options for food workers who are required to continue to work as normal. Keeping all workers in the food production and supply chains healthy and safe is critical to avoid food shortage. Maintaining the movement of food along the food chain is an essential function of all sectors of the food industry and is extremely important for ensuring consumer confidence in the food supply. In order to ensure that the food supply chain remains intact to prevent food shortages there is an urgent requirement for the industry to introduce additional measures to protect food workers from contracting COVID-19; to prevent the risk of exposure to COVID-19, and to strengthen existing food hygiene and sanitation practices.

#### [Coronavirus disease (COVID-19): Food safety for consumers（174-182）](https://www.who.int/emergencies/diseases/novel-coronavirus-2019/question-and-answers-hub/q-a-detail/coronavirus-disease-covid-19-food-safety-for-consumers)

[174、](https://www.who.int/emergencies/diseases/novel-coronavirus-2019/question-and-answers-hub/q-a-detail/coronavirus-disease-covid-19-food-safety-for-consumers)**[Can I get COVID-19 from food?](https://www.who.int/emergencies/diseases/novel-coronavirus-2019/question-and-answers-hub/q-a-detail/coronavirus-disease-covid-19-food-safety-for-consumers)**

There is currently no evidence that people can catch COVID-19 from food or food packaging. COVID-19 is a respiratory illness and the transmission route is through person-to-person contact and through direct contact with respiratory droplets generated when an infected person coughs or sneezes.

175、**[Can the virus live on the surface of foods (including fruits and vegetables, frozen foods, pre-packaged foods)?](https://www.who.int/emergencies/diseases/novel-coronavirus-2019/question-and-answers-hub/q-a-detail/coronavirus-disease-covid-19-food-safety-for-consumers)**

Coronaviruses cannot multiply in food – they need a live animal or human host to multiply and survive ( [see question 10 of Questions relating to food businesses](https://who.int/news-room/q-a-detail/coronavirus-disease-covid-19-food-businesses)).

176、**[How to wash fruits and vegetables? Just with water, or something else?](https://www.who.int/emergencies/diseases/novel-coronavirus-2019/question-and-answers-hub/q-a-detail/coronavirus-disease-covid-19-food-safety-for-consumers)**

Washing fruit and vegetables with potable water is sufficient: it is recommended to follow the [WHO Five keys to safer food](https://www.who.int/publications-detail-redirect/9789241594639).

177、**[Can the virus live on the surface of food packaging? How long? Is it necessary to disinfect?](https://www.who.int/emergencies/diseases/novel-coronavirus-2019/question-and-answers-hub/q-a-detail/coronavirus-disease-covid-19-food-safety-for-consumers)**

See [question 10 of Questions relating to food businesses](https://who.int/news-room/q-a-detail/coronavirus-disease-covid-19-food-businesses). It is not necessary to disinfect food packaging materials, but hands should be properly washed after handling food packages and before eating.

178、**[How long is it to cook food? To what temperature to kill the virus?](https://www.who.int/emergencies/diseases/novel-coronavirus-2019/question-and-answers-hub/q-a-detail/coronavirus-disease-covid-19-food-safety-for-consumers)**

This virus is not more resistant to heat than the usual viruses and bacteria found in food. As recommended for good hygiene practice, foods should be thoroughly cooked to at least 70°C. It is recommended to follow the [WHO Five keys to safer food](https://www.who.int/publications-detail-redirect/9789241594639).

179、**[What precautions should consumers take in grocery stores?](https://www.who.int/emergencies/diseases/novel-coronavirus-2019/question-and-answers-hub/q-a-detail/coronavirus-disease-covid-19-food-safety-for-consumers)**

Consumers should maintain a safe physical distance of at least one metre from all other shoppers and staff while queuing before entering the store and while shopping in the store. If a trolley or basket is used while shopping, sanitize the handle before and after use. Hands should be sanitized before entering the store. Practice good coughing/sneezing etiquette while in the store. Avoid touching mouth, nose or eyes during shopping. Minimise direct hand contact with food by using available tongs and serving utensils. Use contactless payment rather than cash/notes (where feasible).

180、**[Is food/grocery delivery safe?](https://www.who.int/emergencies/diseases/novel-coronavirus-2019/question-and-answers-hub/q-a-detail/coronavirus-disease-covid-19-food-safety-for-consumers)**

Yes, if the provider follows good personal and food hygiene practices. After accepting food/grocery deliveries, hands should be washed with soap and water.

181、**[What is the best household disinfectant for surfaces?](https://www.who.int/emergencies/diseases/novel-coronavirus-2019/question-and-answers-hub/q-a-detail/coronavirus-disease-covid-19-food-safety-for-consumers)**

Regular household cleaning and disinfection products will effectively eliminate the virus from household surfaces.  For cleaning and disinfecting households with suspected or confirmed COVID19 illnesses - surface virucidal disinfectants, such as 0.05% sodium hypochlorite (NaClO) and products based on ethanol (at least 70%), should be used.

182、**[Is it still safe to go to food markets? Animal markets? Wet markets?](https://www.who.int/emergencies/diseases/novel-coronavirus-2019/question-and-answers-hub/q-a-detail/coronavirus-disease-covid-19-food-safety-for-consumers)**

It should be safe provided it is possible to maintain a safe physical distance of at least one metre from all other shoppers and staff, it is possible to wash/sanitize hands, and that Good Manufacturing Practices and Good Hygienic Practices (GMP/GHP) standards are maintained in the market. For more recommendations on how to minimise the risk of transmission of emerging pathogens in wet markets, see the [WHO recommendations to reduce risk of transmission of emerging pathogens from animals to humans in live animal markets or animal product markets](file:////emergencies/diseases/novel-coronavirus-2019/origins-of-the-virus).

#### [Coronavirus disease (COVID-19): Health and safety in the workplace（183-200）](https://www.who.int/emergencies/diseases/novel-coronavirus-2019/question-and-answers-hub/q-a-detail/coronavirus-disease-covid-19-health-and-safety-in-the-workplace)

183、**[Can COVID-19 be transmitted at the workplace?](https://www.who.int/emergencies/diseases/novel-coronavirus-2019/question-and-answers-hub/q-a-detail/coronavirus-disease-covid-19-health-and-safety-in-the-workplace)**

COVID-19 spreads primarily through respiratory droplets or contact with contaminated surfaces. Exposure can occur at the workplace, while travelling to work, during work-related travel to an area with local community transmission, as well as on the way to and from the workplace.

184、**[What is the risk of contracting COVID-19 in the workplace?](https://www.who.int/emergencies/diseases/novel-coronavirus-2019/question-and-answers-hub/q-a-detail/coronavirus-disease-covid-19-health-and-safety-in-the-workplace)**

The risk of exposure to COVID-19 in the workplace depends on the likelihood of coming within 1 metre of others, in having frequent physical contact with people who may be infected with COVID-19, and through contact with contaminated surfaces and objects.

185、**[How can people assess the risk for exposure to COVID-19 in their workplace and plan for preventive measures?](https://www.who.int/emergencies/diseases/novel-coronavirus-2019/question-and-answers-hub/q-a-detail/coronavirus-disease-covid-19-health-and-safety-in-the-workplace)**

Managers with the support of an occupational health and safety advisor should carry out rapid risk assessments to determine the possibility of exposure risk in order to put in place preventive measures. This should be done for each specific work setting and each job.

### Low exposure risk

Jobs or work without frequent, close contact with the general public or others. Workers in this group have minimal occupational contact with the public and other co-workers. Examples of such jobs may include remote workers (i.e., working from home), office workers without frequent close contact with others and workers providing teleservices.

### Medium exposure risk

Jobs or tasks with close, frequent contact with the general public or others. This risk level may apply to workers who have frequent and close contact with the people in high-population-density work environments (e.g. food markets, bus stations, public transport, and other work activities where physical distancing of at least 1 metre may be difficult to observe), or tasks that require close and frequent contact between co-workers. This may also include frequent contact with people returning from areas with community transmission. Examples of such jobs may include frontline workers in retail, home deliveries, accommodation, construction, police and security, public transport, and water and sanitation.

### High exposure risk

Jobs or tasks with close contact with people who may be more likely to have COVID-19, as well as contact with objects and surfaces possibly contaminated with the virus. Examples include transporting people known or suspected to have COVID-19 without separation between the driver and the passenger, providing domestic services or home care for people with COVID-19, and having contact with the deceased who were known or suspected of having COVID-19 at the time of their death. Jobs that may fall under this category include domestic workers, social care workers, personal transport  and home delivery providers and home repair technicians (plumbers, electricians) who have to provide services in the homes of people with COVID-19.

186、**[Who should carry out the workplace risk assessment?](https://www.who.int/emergencies/diseases/novel-coronavirus-2019/question-and-answers-hub/q-a-detail/coronavirus-disease-covid-19-health-and-safety-in-the-workplace)**

Employers and managers, in consultation with workers, should carry out and regularly update the risk assessment for work-related exposure to COVID-19, preferably with the support of occupational health services.

187、**[What are the key considerations for the workplace risk assessment?](https://www.who.int/emergencies/diseases/novel-coronavirus-2019/question-and-answers-hub/q-a-detail/coronavirus-disease-covid-19-health-and-safety-in-the-workplace)**

For each risk assessment, consider the environment, the task, the threat, resources available, such as personal protective equipment, and the feasibility of protective measures. The risk assessment should also extend to collective accommodation provided by the employer for workers, such as dormitories.  Essential public services, such as security and police, food retail, accommodation, public transport, deliveries, water and sanitation, and other frontline workers may be at an increased risk of exposure to occupational hazards for health and safety. Workers who may be at higher risk of developing severe COVID-19 illness because of age or pre-existing medical conditions should be considered in the risk assessment for individuals.

188、**[How should employers decide when to open, close or re-open workplaces and/or suspend or downscale work activities?](https://www.who.int/emergencies/diseases/novel-coronavirus-2019/question-and-answers-hub/q-a-detail/coronavirus-disease-covid-19-health-and-safety-in-the-workplace)**

Deciding to close or re-open a workplace or suspend or downscale work activities should rely on the risk assessment, the capacity to put in place protective measures and the level of compliance, and recommendations of national authorities.

189、**[What key measures to protect against COVID-19 should be undertaken in ALL workplaces?](https://www.who.int/emergencies/diseases/novel-coronavirus-2019/question-and-answers-hub/q-a-detail/coronavirus-disease-covid-19-health-and-safety-in-the-workplace)**

Measures to prevent transmission of COVID-19 that apply to all workplaces and all people at the workplace include frequent hand-washing or disinfection with alcohol based hand sanitizer, respiratory hygiene such as covering coughs, physical distancing of at least 1 metre or more according to the national recommendations, wearing of masks where distancing is not possible, regular environmental cleaning and disinfection, and limiting unnecessary travel. Clear policies and messages, training, and education for staff and managers to increase awareness of COVID-19 are essential. The management of people with COVID-19 or their contacts is also critical e.g. requiring workers who are unwell or who develop symptoms to stay at home, self isolate and contact a medical professional or the local COVID-19 information line for advice on testing and referral.

190、**[What additional measures should be taken at workplaces and for jobs at medium risk?](https://www.who.int/emergencies/diseases/novel-coronavirus-2019/question-and-answers-hub/q-a-detail/coronavirus-disease-covid-19-health-and-safety-in-the-workplace)**

Workplaces for jobs at medium risk require daily cleaning and disinfection at least two times a day of objects and surfaces that are touched regularly, including all shared rooms, surfaces, floors, bathrooms, and changing rooms. Consider suspending any activity where physical distancing of at least 1 metre cannot be implemented in full. If this is not possible, increase ventilation, implement enhanced regular hand hygiene, and require staff to wear appropriate face masks, goggles, gloves and work clothes during cleaning procedures that generate splashes, providing training on their use. Organize changing and washing of work clothes at the workplace, so that workers to do take them home.

191、**[What additional measures should be taken at workplaces and for jobs at high risk?](https://www.who.int/emergencies/diseases/novel-coronavirus-2019/question-and-answers-hub/q-a-detail/coronavirus-disease-covid-19-health-and-safety-in-the-workplace)**

In work areas at high risk, assess the possibility of suspending the activity; enhance regular hand hygiene; provide medical masks, disposable gowns, gloves, and eye protection for workers who must work in the homes of people who are suspected or known to have COVID-19; train workers in infection prevention and control practices and use of personal protective equipment; avoid assigning tasks with high risk to workers who have pre-existing medical conditions, are pregnant, or older than 60 years of age.

192、**[What should be taken into consideration when setting a physical distance at the workplace?](https://www.who.int/emergencies/diseases/novel-coronavirus-2019/question-and-answers-hub/q-a-detail/coronavirus-disease-covid-19-health-and-safety-in-the-workplace)**

WHO recommends keeping a physical distance of at least 1 metre between each person in all settings, including in workplaces. Because transmission can occur in crowded workplaces, WHO recommends providing sufficient space, at least 10 square meters, for every worker. National recommendations for physical distancing may require greater physical distance and should be complied with.

In order to support compliance with national or local recommendations, implement physical distance guidelines in a way that is practical and feasible in the context of work tasks, and which is acceptable to both workers and employers. Stimulate workers to comply with physical distancing norms also at events outside the workplace, in the community, and in dormitories.

Risk assessment and consultation between employers and workers is very important for setting up and implementing physical distancing measures at the workplace. This may require modification of workstations, changing the use of common spaces and transport vehicles, staggered work shifts, split teams and other measures to reduce social mixing at the workplace.

If physical distancing measures at the workplace are not feasible for specific work tasks, consider whether the work can be suspended, and if this is not possible, apply additional protective measures, such as the use of screens, sneeze guards,  face masks, enhanced hand hygiene, ventilation and disinfection.

Physical distancing alone can’t prevent COVID-19 transmission, it is important that it is combined with other public health measures, such as hand and respiratory hygiene, environmental clean-up and disinfection of commonly touched surfaces and objects, ventilation, wearing face masks and a policy of staying at home if unwell.

193、**[What are the rights, duties and responsibilities of employers?](https://www.who.int/emergencies/diseases/novel-coronavirus-2019/question-and-answers-hub/q-a-detail/coronavirus-disease-covid-19-health-and-safety-in-the-workplace)**

Employers, workers, and their organizations should collaborate with health authorities to prevent and control COVID-19. Cooperation between management and workers and their representatives is essential for workplace‐related prevention measures. International labour standards on the rights and responsibilities of workers and employers in occupational safety and health should be fully respected.

Employers, in consultation with workers and their representatives, should plan and implement measures to prevent and mitigate COVID-19 at the workplace through engineering and administrative controls, and provide personal protective equipment and clothing according to the risk assessment. Such measures should not involve any expenditure on the part of the workers.

Special measures are needed to protect workers at higher risk of developing severe disease, such as those age 60 and over, or with underlying medical conditions, upon recommendation of the occupational health services. Workers in the informal economy and digital labour platforms, those in small enterprises, domestic and migrant workers should not be left behind in the protection of their health and safety at work and their livelihood*.*

There should be no social stigma or discrimination at the workplace for any reason, including access to information and protection from COVID-19, occupational health services and mental health and psychosocial support.

If COVID-19 is contracted through occupational exposure, it could be considered an occupational disease and, if so determined, should be reported and compensated according to the international labour standards and the national schemes for employment injury benefits.

194、**[What are the rights, duties and responsibilities of workers?](https://www.who.int/emergencies/diseases/novel-coronavirus-2019/question-and-answers-hub/q-a-detail/coronavirus-disease-covid-19-health-and-safety-in-the-workplace)**

Workers are responsible to follow the measures for occupational safety and health and infection prevention and control established for their workplace, and to participate in training provided by the employer. Workers should report to their supervisor any situation which may present an imminent and serious danger to their life or health. Workers have the right to remove themselves from any work situation that they have reasonable justification to believe presents an imminent and serious danger to their life or health, and should be protected from any undue consequences as a result of exercising this right.

195、**[How can workplaces plan for the prevention and mitigation of COVID-19?](https://www.who.int/emergencies/diseases/novel-coronavirus-2019/question-and-answers-hub/q-a-detail/coronavirus-disease-covid-19-health-and-safety-in-the-workplace)**

Workplaces should develop action plans to prevent and mitigate COVID-19 as part of the business continuity plan and according to the results of the risk assessments and the epidemiological situation.

The action plan and preventive measures should be regularly monitored and updated. Workers and their representatives should be consulted and should participate in the development, monitoring and updating of the workplace COVID-19. It is very important to monitor the effectiveness of preventive measures, and the compliance of workers, visitors, customers, clients and sub-contractors with the measures. The plans should be updated when someone with known or suspected COVID-19 is at the workplace.

196、**[Can the return to the workplace be immediate after public measures are lifted?](https://www.who.int/emergencies/diseases/novel-coronavirus-2019/question-and-answers-hub/q-a-detail/coronavirus-disease-covid-19-health-and-safety-in-the-workplace)**

The return to work premises should be carefully planned ahead, with preventive measures put in place according to the risk assessment of the different jobs and work tasks. All possible risks for safety and health should be assessed, such as risks resulting from reduced maintenance of machines and facilities during the closure period.  If a return to work is rushed and not done in a phased and cautious manner, it puts lives at risk, and threatens to undermine efforts to restore social and economic activity.

197、**[Does WHO recommend thermal testing of people entering a workplace?](https://www.who.int/emergencies/diseases/novel-coronavirus-2019/question-and-answers-hub/q-a-detail/coronavirus-disease-covid-19-health-and-safety-in-the-workplace)**

Temperature screening cannot detect all cases of COVID-19, since infected individuals may not have fever early in the course of infection or illness, such as during the incubation period or just before other symptoms begin, even though they may already be infectious. Some people may reduce fever with a fever-reducing medication if they are concerned about the possible consequences of not coming to work. Relying on temperature screening alone will not stop the spread of COVID-19 at work.

Thermal screening at the workplace can be considered part of a package of measures to prevent and control COVID-19 at the workplace. Workers should be encouraged to self-monitor their health, possibly with the use of questionnaires, and take their own temperature regularly at home. Workplaces should adopt “stay at home if unwell” and flexible sick leave policies to discourage workers with symptoms consistent with COVID-19 from coming to the workplaces.

198、**[Does WHO recommend workers wear masks at the workplace (office or others)? If yes, what type of masks?](https://www.who.int/emergencies/diseases/novel-coronavirus-2019/question-and-answers-hub/q-a-detail/coronavirus-disease-covid-19-health-and-safety-in-the-workplace)**

Wearing masks depends on the risk assessment. For jobs and tasks that carry a medium or high risk, for people aged 60 and older, and for those with underlying health conditions, a medical mask and other personal protective equipment should be provided. Fabric masks or face coverings are currently recommended for younger people and those with no symptoms where physical distancing is not achievable. This prevents the spread of virus from the wearer (who could have COVID-19 but no symptoms) to others. The policy on wearing a mask or face covering in low risk workplaces should be in line with national or local guidelines. Masks may carry some risks if not used properly.

199、**[Are there any directives on office ventilation and air conditioning use?](https://www.who.int/emergencies/diseases/novel-coronavirus-2019/question-and-answers-hub/q-a-detail/coronavirus-disease-covid-19-health-and-safety-in-the-workplace)**

There should be fresh, clean air in all workplaces. For jobs and work tasks at medium or high risk of exposure, WHO recommends an increased ventilation rate through natural aeration or artificial ventilation, preferably without re-circulation of the air. In case of air recirculation, filters should be cleaned regularly.

200、**[What mental health and psychosocial support should be provided to workers during COVID-19?](https://www.who.int/emergencies/diseases/novel-coronavirus-2019/question-and-answers-hub/q-a-detail/coronavirus-disease-covid-19-health-and-safety-in-the-workplace)**

COVID-19 is associated with a range of concerns, such as fear of falling ill and dying, of being socially excluded, placed in quarantine, or losing a livelihood. Symptoms of anxiety and depression are common reactions for people in the context of COVID-19. Mental health and psychosocial support should be made available to all workers. Comprehensive risk assessments can help identify and mitigate related occupational hazards for mental health

Full Guideline Document Considerations for public health and social measures in the workplace in the context of COVID-19 is accessible at: <https://www.who.int/publications/i/item/considerations-for-public-health-and-social-measures-in-the-workplace-in-the-context-of-covid-19>

#### [Coronavirus disease (COVID-19): Herd immunity, lockdowns and COVID-19（201-204）](https://www.who.int/emergencies/diseases/novel-coronavirus-2019/question-and-answers-hub/q-a-detail/herd-immunity-lockdowns-and-covid-19)

201、**[What is ‘herd immunity’?](https://www.who.int/emergencies/diseases/novel-coronavirus-2019/question-and-answers-hub/q-a-detail/herd-immunity-lockdowns-and-covid-19)**

'Herd immunity', also known as 'population immunity', is the indirect protection from an infectious disease that happens when a population is immune either through vaccination or immunity developed through previous infection. WHO supports achieving 'herd immunity' through vaccination, not by allowing a disease to spread through any segment of the population, as this would result in unnecessary cases and deaths.

Herd immunity against COVID-19 should be achieved by protecting people through vaccination, not by exposing them to the pathogen that causes the disease. *Read the Director-General’s*[*12 October media briefing speech*](https://www.who.int/director-general/speeches/detail/who-director-general-s-opening-remarks-at-the-media-briefing-on-covid-19---12-october-2020)*for more detail.*

Vaccines train our immune systems to create proteins that fight disease, known as ‘antibodies’, just as would happen when we are exposed to a disease but – crucially – vaccines work without making us sick. Vaccinated people are protected from getting the disease in question and passing on the pathogen, breaking any chains of transmission. *Visit our*[*webpage*](https://www.who.int/emergencies/diseases/novel-coronavirus-2019/covid-19-vaccines)*on COVID-19 and vaccines for more detail.*

To safely achieve herd immunity against COVID-19, a substantial proportion of a population would need to be vaccinated, lowering the overall amount of virus able to spread in the whole population. One of the aims with working towards herd immunity is to keep vulnerable groups who cannot get vaccinated (e.g. due to health conditions like allergic reactions to the vaccine) safe and protected from the disease. *Read our*[*Q&A*](https://www.who.int/news-room/questions-and-answers/item/vaccines-and-immunization-what-is-vaccination)*on vaccines and immunization for more information.*

The percentage of people who need to be immune in order to achieve herd immunity varies with each disease. For example, herd immunity against measles requires about 95% of a population to be vaccinated. The remaining 5% will be protected by the fact that measles will not spread among those who are vaccinated. For polio, the threshold is about 80%. The proportion of the population that must be vaccinated against COVID-19 to begin inducing herd immunity is not known. This is an important area of research and will likely vary according to the community, the vaccine, the populations prioritized for vaccination, and other factors.

Achieving herd immunity with safe and effective vaccines makes diseases rarer and saves lives.

*Find out more about the science behind herd immunity by watching or reading this*[*interview*](https://www.who.int/emergencies/diseases/novel-coronavirus-2019/media-resources/science-in-5/episode-1)*with WHO’s Chief Scientist, Dr Soumya Swaminathan.*

202、**[What is WHO’s position on ‘herd immunity’ as a way of fighting COVID-19?](https://www.who.int/emergencies/diseases/novel-coronavirus-2019/question-and-answers-hub/q-a-detail/herd-immunity-lockdowns-and-covid-19)**

Attempts to reach ‘herd immunity’ through exposing people to a virus are scientifically problematic and unethical. Letting COVID-19 spread through populations, of any age or health status will lead to unnecessary infections, suffering and death.

The vast majority of people in most countries remain susceptible to this virus. Seroprevalence surveys suggest that in most countries, less than 10% of the population have been infected with COVID-19.

We are still learning about immunity to COVID-19. Most people who are infected with COVID-19 develop an immune response within the first few weeks, but we don’t know how strong or lasting that immune response is, or how it differs for different people. There have also been reports of people infected with COVID-19 for a second time.

Until we better understand COVID-19 immunity, it will not be possible to know how much of a population is immune and how long that immunity last for, let alone make future predictions. These challenges should preclude any plans that try to increase immunity within a population by allowing people to get infected.

Although older people and those with underlying conditions are most at risk of severe disease and death, they are not the only ones at risk.

Finally, while most infected people get mild or moderate forms of COVID-19 and some experience no disease, many become seriously ill and must be admitted into hospital. We are only beginning to understand the long-term health impacts among people who have had COVID-19, including what is being described as ‘Long COVID.’ WHO is working with clinicians and patient groups to better understand the long term effects of COVID-19.

*Read the Director-General’s*[*opening remarks*](https://www.who.int/dg/speeches/detail/who-director-general-s-opening-remarks-at-the-media-briefing-on-covid-19---12-october-2020)*at the 12 October COVID-19 briefing for a summary of WHO’s position.*

203、**[What do we know about immunity from COVID-19?](https://www.who.int/emergencies/diseases/novel-coronavirus-2019/question-and-answers-hub/q-a-detail/herd-immunity-lockdowns-and-covid-19)**

Most people who are infected with COVID-19 develop an immune response within the first few weeks after infection.

Research is still ongoing into how strong that protection is and how long it lasts. WHO is also looking into whether the strength and length of immune response depends on the type of infection a person has: without symptoms (‘asymptomatic’), mild or severe. Even people without symptoms seem to develop an immune response.

Globally, data from seroprevalence studies suggests that less 10% of those studied have been infected, meaning that the vast majority of the world’s population remains susceptible to this virus.

For other coronaviruses – such as the common cold, SARS-CoV-1 and Middle East Respiratory Syndrome (MERS) – immunity declines over time, as is the case with other diseases. While people infected with the SARS-CoV-2 virus develop antibodies and immunity, we do not yet know how long it lasts.

*Watch this*[conversation](https://youtu.be/RCZ1gAG_EJc?t=1483)*with Dr Mike Ryan and Dr Maria Van Kerkhove for more information on immunity.*

204、**[What is WHO’s position on ‘lockdowns’ as a way of fighting COVID-19?](https://www.who.int/emergencies/diseases/novel-coronavirus-2019/question-and-answers-hub/q-a-detail/herd-immunity-lockdowns-and-covid-19)**

Large scale physical distancing measures and movement restrictions, often referred to as ‘lockdowns’, can slow COVID‑19 transmission by limiting contact between people.

However, these measures can have a profound negative impact on individuals, communities, and societies by bringing social and economic life to a near stop. Such measures disproportionately affect disadvantaged groups, including people in poverty, migrants, internally displaced people and refugees, who most often live in overcrowded and under resourced settings, and depend on daily labour for subsistence.

WHO recognizes that at certain points, some countries have had no choice but to issue stay-at-home orders and other measures, to buy time.

Governments must make the most of the extra time granted by ‘lockdown’ measures by doing all they can to build their capacities to detect, isolate, test and care for all cases; trace and quarantine all contacts; engage, empower and enable populations to drive the societal response and more.

WHO is hopeful that countries will use targeted interventions where and when needed, based on the local situation

#### [Coronavirus disease (COVID-19): Home care for families and caregivers（205-212）](https://www.who.int/emergencies/diseases/novel-coronavirus-2019/question-and-answers-hub/q-a-detail/coronavirus-disease-covid-19-home-care-for-families-and-caregivers)

205、**[Someone in my household tested positive for COVID-19. Is it safe to care for them at home?](https://www.who.int/emergencies/diseases/novel-coronavirus-2019/question-and-answers-hub/q-a-detail/coronavirus-disease-covid-19-home-care-for-families-and-caregivers)**

People at high risk for severe illness and death from COVID-19 may require care that cannot be provided at home. Contact your healthcare provider for advice. People at high risk include:

- people aged 60 and older;
- people who are pregnant and age 35 and older, and who are obese or have chronic medical conditions;
- people of any age with chronic medical conditions (such as chronic cardiac, pulmonary, renal, metabolic, neurologic, liver or hematologic diseases); and
- people with immunosuppressive conditions (such as HIV/AIDS, patients receiving chemotherapy or steroids, and people with cancer.)

If you are in any high-risk groups and you are NOT vaccinated, you are at risk of more severe disease and death.

If you have recently tested positive for COVID-19 infection, have mild symptoms AND are at high risk of severe disease, contact your health provider. There may be effective treatments available to you.

If someone in your home is not at high risk for severe disease, tests positive for COVID-19, and has no [symptoms](https://www.who.int/multi-media/details/covid-19-coronavirus-symptoms) or mild symptoms, they can usually be cared for safely at home. A quick guide to home care for people with COVID-19 infection can be found [here](https://www.who.int/multi-media/details/home-care-for-covid-19-guide-for-family-and-caregivers). As you take care of others, don’t forget to take care of yourself too. Wear a medical mask when sharing a space with someone with COVID-19. Everyone should follow prevention measures:

- Stay at least 1 metre away from the sick person;
- Where possible, open windows to bring fresh air into the sick person’s room;
- Cough or sneeze into a bent elbow;
- Clean your hands frequently; and
- Get vaccinated as soon as it is your turn.

Monitor the symptoms of the person with COVID-19 regularly, and call your healthcare provider immediately if you see any of these danger signs:

- Difficulty breathing
- Chest pain
- Confusion
- Loss of speech or mobility

206、**[Someone in my household tested positive for COVID-19. They have very mild symptoms, and our doctor says it is safe for me to care for them at home. What should I do to keep myself and others in the household healthy?](https://www.who.int/emergencies/diseases/novel-coronavirus-2019/question-and-answers-hub/q-a-detail/coronavirus-disease-covid-19-home-care-for-families-and-caregivers)**

It’s hard when someone close to you is unwell. Even though you may want to provide comfort and company to your ill relative, it is important to reduce the likelihood that you or other family members catch COVID-19. For people with mild or moderate symptoms, the best thing you can do is provide the care they need while also keeping a safe distance.

**First of all – protect the health of others in the household**

People with COVID-19 infection should (where possible) be isolated in a separate room, away from others in the home. If possible, close the door to stop air from moving from the infected person’s room into the rest of the home. If it’s safe, open windows and turn on a fan in the room if you have one. No other visitors should be allowed in the home when someone in the household has COVID-19 infection. Follow guidance from your country’s health department or ministry about regarding whether or not close contacts need to isolate at home.

If it is not possible to isolate the infected person in your home, try to move people at risk of severe disease to a household where they will not be exposed to possible infection.

If you have to share space in your home with someone with COVID-19, open windows to bring in fresh air if it’s safe to do so. COVID-19 spreads easily in places that are poorly ventilated. Learn more about [ventilation](https://www.who.int/news-room/questions-and-answers/item/coronavirus-disease-covid-19-ventilation-and-air-conditioning).

**Secondly - protect the health of the person caring for the person with COVID-19 infection**

The spread of the COVID-19 virus occurs most often when an infected person is in close or direct contact with another person. If possible, there should be only one person in the household providing care to the person who has COVID-19 infection.

If possible, choose someone to be the caregiver who is healthy and not at high risk. Caregivers should wash their hands before AND after any interaction with the person with COVID-19 infection. Both the caregiver and the person who is infected should wear a medical mask whenever they share a space with each other. If possible, open windows when the caregiver is in the room with the person with COVID-19 infection.

**Monitor how you feel.**

Caregivers and others in a home with a person with COVID-19 infection should pay close attention to how they feel (even if they are vaccinated or are being careful). On average, if takes 5-6 days from when someone is infected for symptoms to show. However, it can take up to 14 days.

Symptoms may vary. Monitor yourself and others in your home for any symptoms of COVID-19 -- including fever, cough, tiredness, loss of taste or smell, sore throat, muscle or body ache, headache, running nose, loss of appetite, nausea, diarrhea or shortness of breath. Or altered mental status.  Get tested if you have any of these symptoms.

Seek immediate medical care if you have any of these severe symptoms of COVID-19:

- Difficulty breathing
- Chest pain
- Confusion
- Loss of speech or mobility

For young children, seek immediate medical care if you notice any of these symptoms:

- High fever
- Rapid breathing
- Lethargy or not interacting when awake
- Difficulty in feeding (unable to drink or breastfeed)
- Blue lips or face

If you think you may have COVID-19 infection and are waiting for test results, avoid contact with other people if possible until you know whether or not you are infected.

**Watch for warning signs.**

Pay attention to any changes in the signs and symptoms a person with COVID-19 infection in your care. Some symptoms may be signals that more urgent medical care is needed. Depending on the age of the person in your care, their symptoms may look different. Adults may look dehydrated, have shortness of breath or chest pains. They may also complain of light-headedness. Children may suddenly appear confused or refuse to eat. Their face or lips may turn blue. Babies may be unable to breastfeed. These symptoms are warning signs that urgent care is needed.

If the person with COVID-19 infection under your care shows any of these signs, contact your healthcare provider immediately.

**Keep things clean.**

Any surfaces and household items touched by the person with COVID-19 infection should be cleaned and disinfected at least once a day. Household items include dishes, cups and flatware.

COVID-19 can spread from an infected person’s mouth or nose in small liquid particles when they cough, sneeze, speak, sing or breathe. These particles range from larger respiratory droplets to smaller aerosols.

Current evidence suggests that the virus spreads mainly between people who are in close contact with each other, typically within 1 metre. A person can be infected when aerosols or droplets containing the virus are inhaled or come directly into contact with the eyes, nose, or mouth. The virus can also spread in poorly ventilated and/or crowded indoor settings, where people tend to spend longer periods of time. This is because aerosols remain suspended in the air or travel farther than 1 metre.

People may also become infected by touching surfaces that have been contaminated with the virus and then touching their eyes, nose or mouth without cleaning their hands.

**Continue to practice prevention measures**.

- Keep a distance of at least 1 meter from others;
- Wear a well-fitted mask over your mouth and nose;
- Open windows
- Cough or sneeze into a bent elbow;
- Clean your hands frequently; and
- Get vaccinated as soon as it’s your turn.

207、**[My child has tested positive for COVID-19. What should I do?](https://www.who.int/emergencies/diseases/novel-coronavirus-2019/question-and-answers-hub/q-a-detail/coronavirus-disease-covid-19-home-care-for-families-and-caregivers)**

If your child tests positive for COVID-19, contact your healthcare provider for guidance. You should seek immediate care if a baby is unable to breastfeed, or if a child is unable to drink, has high fever, rapid breathing, suddenly appears lethargic or doesn’t interact when they are awake, or if their face or lips turn blue.

It’s natural to feel concerned or anxious about your child’s health if they are infected with COVID-19.  While some of the same [home care](https://www.who.int/multi-media/details/What-to-do-in-your-household-if-your-child-tests-positive-for-COVID-19) guidance applies to children and adults, there are special considerations when taking care of child.

**Take care of the child with COVID-19 infection**

It is natural for children to be anxious and concerned after testing positive for COVID-19. Listen to their concerns, and help them understand why it’s important to rest and keep a distance from other family members. Encourage them to rest, to stay hydrated and to eat healthy foods. If they are old enough, talk to them about COVID-19 and share accurate information. Reassure your child that their health and safety is your top priority.

Be responsive to your child’s needs. Think up ways together for children to stay connected with other family members and friends. Make the space in which the child and caregiver are isolating as child-friendly as possible. Play and learning continue to be an important part of a child’s life.

Learn more about [helping children hope with stress](https://www.who.int/multi-media/details/helping-children-cope-with-stress-during-covid-19-outbreak).

**Reduce contact with others**

The spread of the COVID-19 virus occurs most often when a person with COVID-19 infection is in close or direct contact with another person.

WHO recommends that people with COVID-19 be isolated in a separate room away from other members of the household. However, children should not be isolated on their own. If possible, there should be only one person, who is healthy and not at high risk, in the household providing care to the child with infection. The designated caregiver should take care of the child at all times and monitor their symptoms and safety.

The caregiver should wear a medical mask when caring for the infected child and should wash their hands before AND after any interaction with the infected child. If it is possible and safe, keep the room or space well ventilated and open windows frequently. The child with COVID-19 infection should wear a medical mask in shared spaces, as long as the child can tolerate it.

**Prevent transmission to others**

If it is safe and possible, open windows to get fresh air into the room where the child with COVID-19 infection is staying. Where it is not possible to separate the child and caregiver from the rest of the family, try to separate those at high risk for severe disease from the child with infection and their caregiver.

Keep things clean: Any surfaces and household items, such as dishes, cups and cutlery, touched by the child with COVID-19 infection should be cleaned and disinfected at least once a day. Separate dishes and eating utensil should be used from the infected child.

Encourage members of the household to clean their hands frequently using soap and water or an alcohol-based hand sanitiser.

**Monitor the child with COVID-19 infection and others**

The caregiver and others in a home with a child with COVID-19 infection should pay close attention to how they feel (even if they are vaccinated or are being careful). On average, it takes 5-6 days from when someone is infected for symptoms to show. However, it can take up to 14 days.

The caregiver and others in your home should pay attention for any symptoms of COVID-19: including fever, cough, tiredness, loss of taste or smell, sore throat, muscle or body ache, diarrhoea or shortness of breath. Get tested if you have any of these symptoms.

Seek immediate medical care if you have any of these severe symptoms of COVID-19:

- Difficulty breathing
- Chest pain
- Confusion
- Loss of speech or mobility

The caregiver should pay attention and monitor COVID-19 symptoms of the child with infection regularly and seek immediate medical care if notice any of these symptoms:

- High fever
- Rapid breathing
- Lethargy or not interacting when awake
- Difficulty in feeding (unable to drink or breastfeed)
- Blue lips or face

208、**[Our healthcare provider has recommended that I use a pulse oximeter as part of the care I am providing at home to a COVID-19 patient. What is a pulse oximeter and what does it do?](https://www.who.int/emergencies/diseases/novel-coronavirus-2019/question-and-answers-hub/q-a-detail/coronavirus-disease-covid-19-home-care-for-families-and-caregivers)**

A pulse oximeter is a small medical device used to measure the level of oxygen in the blood. People with COVID-19 may have low oxygen levels, which can be life-threatening.

To measure the level of oxygen in the blood, a pulse oximeter is usually placed on the finger or toe of the person who is sick. Using a pulse oximeter is painless and only takes a few minutes. If your healthcare provider recommends the use of a pulse oximeter, be sure to get instructions about how to use it and how to read and understand the results, and report them to your healthcare provider. Accurate measurements and readings from a pulse oximeter are important signs in determining if and when a COVID-19 patient at home may need urgent care at a healthcare facility. As a general rule, a decline below 90%, or a progressive downwards trend, can be an early warning of need for further medical assessment.

Most COVID-19 patients being cared for at home will not require a pulse oximeter. [Learn more about medical oxygen and pulse oximeters.](https://www.who.int/emergencies/diseases/novel-coronavirus-2019/media-resources/science-in-5/episode-33---medical-oxygen)

209、**[What should I do if the symptoms of the person with COVID-19 infection in my care seem to be getting worse?](https://www.who.int/emergencies/diseases/novel-coronavirus-2019/question-and-answers-hub/q-a-detail/coronavirus-disease-covid-19-home-care-for-families-and-caregivers)**

If symptoms worsen, contact your healthcare provider immediately.

Some symptoms may be signals that more urgent medical care is needed. Depending on the age of the person in your care, their symptoms may look different. Adults may look dehydrated, have shortness of breath or chest pains. They may also complain of light-headedness. Children may suddenly appear confused or refuse to eat. Their face or lips may turn blue. Babies may be unable to breastfeed. These symptoms are warning signs that urgent care is needed.

210、**[How long does the person with COVID-19 infection need to stay in isolation?](https://www.who.int/emergencies/diseases/novel-coronavirus-2019/question-and-answers-hub/q-a-detail/coronavirus-disease-covid-19-home-care-for-families-and-caregivers)**

People with symptoms should stay isolated for a minimum of 10 days after the first day they developed symptoms, plus another 3 days after the end of symptoms – when they are without fever and without respiratory symptoms.

People without symptoms should stay isolated for a minimum of 10 days after testing positive.

Monitor the symptoms of the person with COVID-19 infection’s symptoms regularly, and call your healthcare provider immediately if you see any of these danger signs:

- Difficulty breathing
- Chest pain
- Confusion
- Loss of speech or mobility

For young children, seek immediate medical care if you notice any of these symptoms:

- High fever
- Rapid breathing
- Lethargy or not interacting when awake
- Difficulty in feeding (unable to drink or breastfeed)
- Blue lips or face

211、**[Someone in my household has COVID-19. Do other people in the household need to isolate?](https://www.who.int/emergencies/diseases/novel-coronavirus-2019/question-and-answers-hub/q-a-detail/coronavirus-disease-covid-19-home-care-for-families-and-caregivers)**

WHO recommends that people who have been in contact with a person with COVID-19 infection should quarantine themselves if they have had face-to-face or direct physical contact with someone who has COVID-19 infection or is suspected to have COVID-19 infection. People who have not used medical masks or appropriate personal protective equipment in caring for someone with COVID-19 infection should also quarantine, if possible.

Many countries and regions have their own policies about isolation and quarantine for people who have been in contact with people who have COVID-19 infection. Follow the guidance from your health department or ministry.

212、**[If I have been vaccinated against COVID-19, can I safely care for someone with COVID-19 infection without risk?](https://www.who.int/emergencies/diseases/novel-coronavirus-2019/question-and-answers-hub/q-a-detail/coronavirus-disease-covid-19-home-care-for-families-and-caregivers)**

No. Even if you have been vaccinated, it is important to continue practicing all other prevention measures too. Vaccination does not fully protect you against infection, and you may still spread the virus to others if you are infected. WHO recommends that you should still wear a medical mask and follow prevention measures when you are caring for someone with COVID-19 infection, even if you are vaccinated.

Even though the COVID-19 vaccines are highly effective against serious disease and death, some people will still get infected or ill after they have been vaccinated. There is still a chance you could be infected with the virus and pass it on to other people around you who have not been vaccinated.

#### [Coronavirus disease (COVID-19): Home care for health workers and administrators（213-218）](https://www.who.int/emergencies/diseases/novel-coronavirus-2019/question-and-answers-hub/q-a-detail/coronavirus-disease-covid-19-home-care-for-health-workers-and-administrators)

213、**[When should a patient with COVID-19 be cared for at home?](https://www.who.int/emergencies/diseases/novel-coronavirus-2019/question-and-answers-hub/q-a-detail/coronavirus-disease-covid-19-home-care-for-health-workers-and-administrators)**

Ideally, all patients with COVID-19 are cared for in a healthcare facility. However, there may be some circumstances where patients may not require hospitalization or inpatient care is unavailable or unsafe, such as when capacity is insufficient to meet the demand for healthcare services. Patients should be assessed on a case-by-case basis by the health worker to determine where their care needs can best be met.

214、**[What factors determine whether a COVID-19 patient can be cared for at home?](https://www.who.int/emergencies/diseases/novel-coronavirus-2019/question-and-answers-hub/q-a-detail/coronavirus-disease-covid-19-home-care-for-health-workers-and-administrators)**

Patients with mild or moderate disease can be considered for home care if the home setting is suitable for the isolation and care of a COVID-19 patient, and if the patient is under the age of 60, does not smoke, is not obese, and does not have other diseases such as cardiovascular disease, diabetes mellitus, chronic lung disease, cancer, chronic kidney disease, immunosuppression. An assessment about home care for each patient should be based on the following factors:

- Clinical evaluation of the patient.
- Evaluation of the patient’s home setting according to infection prevention and control (IPC) criteria (e.g., ability to carry out hand and respiratory hygiene, environmental cleaning, adequate ventilation, limitations on movement around or from the house).
- Presence of vulnerable people at higher risk of COVID-19 in the home.
- Ability of a caregiver to provide care and closely monitor the evolution of the patient’s health, at least once per day, and to recognize signs and symptoms of any worsening of the health status.
- Availability of trained health workers to support the patient and caregiver (home-based, phone, telemedicine, trained community workers or outreach teams).

If adequate isolation from others in the home and infection prevention control measures cannot be ensured, then isolation in designated community facilities or a health facility may need to be arranged, with consent from the patient and in agreement with the caregiver and household members.

It is important to note that in areas with other endemic infections that cause fever, such as influenza, malaria, dengue, etc., febrile patients should seek medical care, be tested and treated for those endemic infections per routine protocols, irrespective of the presence of respiratory signs and symptoms.

Home care does not replace healthcare by professionals. Those patients who receive homecare should be regularly monitored by health workers.

215、**[What additional factors should be taken into consideration when assessing the home setting to determine if it is safe for patients and families to remain at home?](https://www.who.int/emergencies/diseases/novel-coronavirus-2019/question-and-answers-hub/q-a-detail/coronavirus-disease-covid-19-home-care-for-health-workers-and-administrators)**

There are a number of environmental and social factors to consider for patients to safely remain at home with their families or household members. An overall needs assessment of the patient and family that includes the availability of trained health workers for support should be conducted. A detailed description is available in the Appendix, Box 2, of the [guidance](https://www.who.int/publications-detail-redirect/home-care-for-patients-with-suspected-novel-coronavirus-(ncov)-infection-presenting-with-mild-symptoms-and-management-of-contacts).

216、**[What precautions should health workers take when providing care to patients in home settings?](https://www.who.int/emergencies/diseases/novel-coronavirus-2019/question-and-answers-hub/q-a-detail/coronavirus-disease-covid-19-home-care-for-health-workers-and-administrators)**

Health workers should take the following precautions:

- Perform a risk assessment to select the use of appropriate personal protective equipment (PPE), such as a medical mask, eye protection, gloves and gown when caring for the patient.
- Implement infection prevention and control measures, including hand hygiene.
- Ensure the room where the patient is cared for is well ventilated, opening windows if necessary.
- Provide instructions to caregivers and household members on cleaning and disinfection in the home, and on management of waste, laundry and utensils related to the patient.
- Request the patient to wear a medical mask when providing care or within a 1-metre distance.
- Request limiting the number of household members during visits and maintaining at least a 1-metre distance.
- Remove PPE and discard what is disposable, then perform hand hygiene before leaving the home.
- Make sure that the waste generated from providing care to the patient be placed in strong bags or safety boxes as appropriate, closed completely, disposed as infectious waste and removed from the home.

217、**[Could pregnant women be cared for at home if they have COVID-19?](https://www.who.int/emergencies/diseases/novel-coronavirus-2019/question-and-answers-hub/q-a-detail/coronavirus-disease-covid-19-home-care-for-health-workers-and-administrators)**

Pregnant women with mild or moderate disease can be considered for home care if the home setting is suitable for the isolation and care of a COVID-19 patient, if they do not smoke, are not obese, and do not have other diseases such as cardiovascular disease, diabetes mellitus, chronic lung disease, cancer, chronic kidney disease, immunosuppression. An assessment about home care for each patient should be based on the following factors:

- Clinical evaluation of the patient.
- Evaluation of the patient’s home setting according to IPC criteria (e.g., ability to carry out hand and respiratory hygiene, environmental cleaning, limitations on movement around or from the house).
- Presence of vulnerable people at higher risk of COVID-19 in the home.
- Ability of a caregiver to provide care and closely monitor the evolution of the patient’s health, at least once per day, and to recognize signs and symptoms of any worsening of the health status.
- Availability of trained health workers to support the patient and caregiver (home-based, phone, telemedicine, trained community workers or outreach teams).

218、**[Can children be cared for at home if they have COVID-19?](https://www.who.int/emergencies/diseases/novel-coronavirus-2019/question-and-answers-hub/q-a-detail/coronavirus-disease-covid-19-home-care-for-health-workers-and-administrators)**

Children should be kept together with their parents or caregivers wherever possible. Children with mild or moderate disease can be considered for home care if the home setting is suitable for the isolation and care of a COVID-19 patient, if they are not obese, do not smoke, and do not have other conditions such as cardiovascular disease, diabetes mellitus, chronic lung disease, cancer, chronic kidney disease, or immunosuppression.

Caregivers of children with COVID-19 should monitor for signs and symptoms of clinical deterioration requiring urgent re-evaluation. These include difficulty breathing/fast or shallow breathing (for infants: grunting, inability to breastfeed), blue lips or face, chest pain or pressure, new confusion, inability to awaken/not interacting when awake, inability to drink or keep down any liquids.

If caregivers are suspected or have confirmed COVID-19 infection, medical and non-medical factors must be taken into account due to the negative and possible long-term consequences of even a short period of family separation.

Community protection focal points and caseworkers should help families plan–in advance–agreements for the care of children in case the caregiver becomes ill.  Children living with primary caregivers who are elderly, disabled or have underlying health conditions should be prioritized.

#### [Coronavirus disease (COVID-19): How is it transmitted?（219-223）](https://www.who.int/emergencies/diseases/novel-coronavirus-2019/question-and-answers-hub/q-a-detail/coronavirus-disease-covid-19-how-is-it-transmitted)

219、**[How does COVID-19 spread between people?](https://www.who.int/emergencies/diseases/novel-coronavirus-2019/question-and-answers-hub/q-a-detail/coronavirus-disease-covid-19-how-is-it-transmitted)**

We know that the disease is caused by the SARS-CoV-2 virus, which spreads between people in several different ways.

- Current evidence suggests that the virus spreads mainly between people who are in close contact with each other, for example at a conversational distance. The virus can spread from an infected person’s mouth or nose in small liquid particles when they cough, sneeze, speak, sing or breathe. Another person can then contract the virus when infectious particles that pass through the air are inhaled at short range (this is often called short-range aerosol or short-range airborne transmission) or if infectious particles come into direct contact with the eyes, nose, or mouth (droplet transmission).
- The virus can also spread in poorly ventilated and/or crowded indoor settings, where people tend to spend longer periods of time. This is because aerosols can remain suspended in the air or travel farther than conversational distance (this is often called long-range aerosol or long-range airborne transmission).
- People may also become infected when touching their eyes, nose or mouth after touching surfaces or objects that have been contaminated by the virus.

Further research is ongoing to better understand the spread of the virus and which settings are most risky and why. Research is also under way to study virus variants that are emerging and why some are more transmissible. For updated information on SARS-CoV-2 variants, please read the [weekly epidemiologic updates](https://www.who.int/emergencies/diseases/novel-coronavirus-2019/situation-reports).

220、**[When do infected people transmit the virus?](https://www.who.int/emergencies/diseases/novel-coronavirus-2019/question-and-answers-hub/q-a-detail/coronavirus-disease-covid-19-how-is-it-transmitted)**

Whether or not they have symptoms, infected people can be contagious and the virus can spread from them to other people.

Laboratory data suggests that infected people appear to be most infectious just before they develop symptoms (namely 2 days before they develop symptoms) and early in their illness. People who develop severe disease can be infectious for longer.

While someone who never develops symptoms can pass the virus to others, it is still not clear how frequently this occurs and more research is needed in this area.

221、**[What is the difference between people who are asymptomatic or pre-symptomatic? Don’t they both mean someone without symptoms?](https://www.who.int/emergencies/diseases/novel-coronavirus-2019/question-and-answers-hub/q-a-detail/coronavirus-disease-covid-19-how-is-it-transmitted)**

Both terms refer to people who do not have symptoms. The difference is that ‘asymptomatic’ refers to people who are infected but never develop any symptoms, while ‘pre-symptomatic’ refers to infected people who have not yet developed symptoms but go on to develop symptoms later.

222、**[Are there certain settings where COVID-19 can spread more easily?](https://www.who.int/emergencies/diseases/novel-coronavirus-2019/question-and-answers-hub/q-a-detail/coronavirus-disease-covid-19-how-is-it-transmitted)**

Yes, any situation in which people are in close proximity to one another for long periods of time increases the risk of transmission. Indoor locations, especially settings where there is poor ventilation, are riskier than outdoor locations. Activities where more particles are expelled from the mouth, such as singing or breathing heavily during exercise, also increase the risk of transmission.

The “Three C’s” are a useful way to think about this. They describe settings where transmission of the COVID-19 virus spreads more easily:

- Crowded places;
- Close-contact settings, especially where people have conversations very near each other;
- Confined and enclosed spaces with poor ventilation.

The risk of COVID-19 spreading is especially high in places where these “3Cs” overlap.


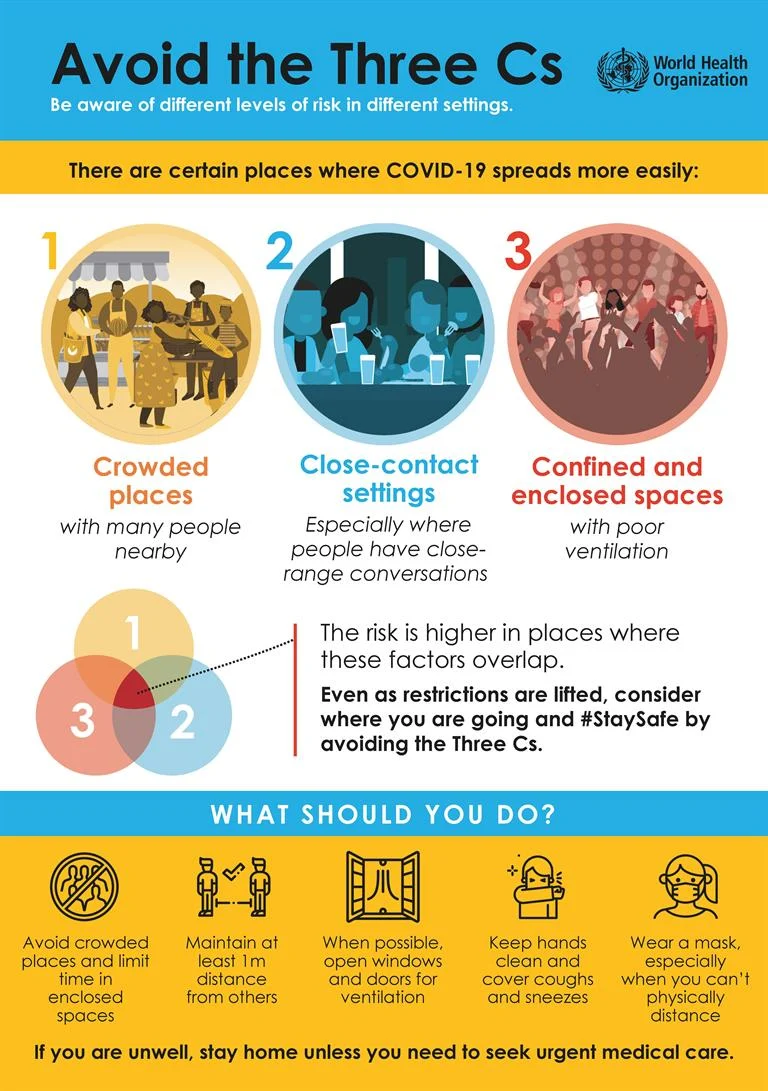


In health facilities where people are receiving treatment for COVID-19, there is an increased risk of infection during medical procedures called aerosol generating procedures. These can produce very small droplets that can stay suspended in the air for longer periods of time and spread beyond conversational distances (typically 1 meter). This is why health workers performing these procedures or in settings where these procedures are performed should take specific airborne protection measures, including using appropriate personal protective equipment such as respirators. This is also why visitors are not permitted in areas where these procedures are being performed.

223、**[How can I reduce my risk of getting COVID-19?](https://www.who.int/emergencies/diseases/novel-coronavirus-2019/question-and-answers-hub/q-a-detail/coronavirus-disease-covid-19-how-is-it-transmitted)**

There are many things you can do to keep yourself and your loved ones safe from COVID-19. Know your risks to lower risks. Follow these basic precautions:

- Follow local guidance: Check to see what national, regional and local authorities are advising so you have the most relevant information for where you are.
- Keep your distance: Stay at least 1 metre away from others, even if they don’t appear to be sick, since people can have the virus without having symptoms.
- Wear a mask:  Wear a well-fitting three-layer mask, especially when you can’t physically distance, or if you’re indoors. Clean your hands before putting on and taking off a mask.
  - Read our [Masks and COVID-19 Q&A](https://www.who.int/emergencies/diseases/novel-coronavirus-2019/question-and-answers-hub/q-a-detail/q-a-on-covid-19-and-masks) and watch our [videos](https://www.who.int/emergencies/diseases/novel-coronavirus-2019/advice-for-public/when-and-how-to-use-masks) on how to wear and make masks.
- Avoid crowded places, poorly ventilated, indoor locations and avoid prolonged contact with others. Spend more time outdoors than indoors.
- Ventilation is important: Open windows when indoors to increase the amount of outdoor air.
- Avoid touching surfaces, especially in public settings or health facilities, in case people infected with COVID-19 have touched them. Clean surfaces regularly with standard disinfectants.
- Frequently clean your hands with soap and water, or an alcohol-based hand rub. If you can, carry alcohol-based rub with you and use it often.
- Cover your coughs and sneezes with a bent elbow or tissue, throwing used tissues into a closed bin right away. Then wash your hands or use an alcohol-based hand rub.
- Get vaccinated: When it’s your turn, get vaccinated. Follow local guidance and recommendations about vaccination.

Read our [public advice page](https://www.who.int/emergencies/diseases/novel-coronavirus-2019/advice-for-public) for more information.

Read our [Q&A about how to stay safe when attending and organizing small gatherings](https://www.who.int/emergencies/diseases/novel-coronavirus-2019/question-and-answers-hub/q-a-detail/q-a-small-public-gatherings-and-covid-19)

Read our [Q&A about ventilation and air conditioning and COVID-19](https://www.who.int/emergencies/diseases/novel-coronavirus-2019/question-and-answers-hub/q-a-detail/q-a-ventilation-and-air-conditioning-and-covid-19)

#### [Coronavirus disease (COVID-19): Hydroxychloroquine（224-225）](https://www.who.int/emergencies/diseases/novel-coronavirus-2019/question-and-answers-hub/q-a-detail/coronavirus-disease-(covid-19)-hydroxychloroquine)

224、**[Does WHO recommend hydroxychloroquine to prevent COVID-19?](https://www.who.int/emergencies/diseases/novel-coronavirus-2019/question-and-answers-hub/q-a-detail/coronavirus-disease-(covid-19)-hydroxychloroquine)**

WHO does not recommend hydroxychloroquine to prevent COVID-19. This recommendation is based on findings from 6 trials, with more than 6000 participants, who did not have COVID-19 and received hydroxychloroquine. Using hydroxychloroquine for prevention had little or no effect on preventing illness, hospitalization or death from COVID-19. Taking hydroxychloroquine to prevent COVID-19 may increase the risk of diarrhoea, nausea, abdominal pain, drowsiness and headache. More information can be found [here.](https://www.who.int/publications/i/item/WHO-2019-nCoV-prophylaxes-2021-1)

Hydroxychloroquine is used to treat autoimmune diseases including arthritis. While considered generally safe, several adverse events are associated with its use, and it should only be used under medical supervision.

225、**[Does WHO recommend hydroxychloroquine as a treatment for COVID-19?](https://www.who.int/emergencies/diseases/novel-coronavirus-2019/question-and-answers-hub/q-a-detail/coronavirus-disease-(covid-19)-hydroxychloroquine)**

WHO does not recommend hydroxychloroquine as a treatment for COVID-19. This recommendation is based on findings from 30 trials with more than 10 000 COVID-19 patients. Hydroxychloroquine did not reduce mortality, the need for or duration of mechanical ventilation. Taking hydroxychloroquine to treat COVID-19 may increase the risk of heart rhythm problems, blood and lymph disorders, kidney injury, liver problems and failure. 
More information can be found [here.](https://www.who.int/publications/i/item/therapeutics-and-covid-19-living-guideline)

Hydroxychloroquine is used to treat autoimmune diseases including arthritis. While considered generally safe, several adverse events are associated with its use, and it should only be used under medical supervision.

#### [Coronavirus disease (COVID-19): Infection prevention and control for health care workers（226X-](https://www.who.int/emergencies/diseases/novel-coronavirus-2019/question-and-answers-hub/q-a-detail/coronavirus-disease-covid-19-infection-prevention-and-control-for-health-care-workers)

226X、On 15 January 2021 this content was updated and moved to a more comprehensive page of resources and information for healthcare workers  [here](https://www.who.int/teams/risk-communication/health-workers-and-administrators).

#### [Coronavirus disease (COVID-19): Malaria and COVID-19（226-236）](https://www.who.int/emergencies/diseases/novel-coronavirus-2019/question-and-answers-hub/q-a-detail/coronavirus-disease-covid-19-malaria-and-covid-19)

226、**[How many malaria-affected countries have reported cases of COVID-19?](https://www.who.int/emergencies/diseases/novel-coronavirus-2019/question-and-answers-hub/q-a-detail/coronavirus-disease-covid-19-malaria-and-covid-19)**

Malaria-endemic countries in all WHO haveregions have reported cases of COVID-19. In the WHO African Region, which carries more than 90% of the global malaria burden, there have been more than 1 million confirmed cases of COVID-19 since the beginning of the pandemic. The [latest situation reports](https://www.who.int/emergencies/diseases/novel-coronavirus-2019/situation-reports) on the COVID-19 pandemic are available on the WHO website.

227、**[Should core malaria vector control interventions be maintained in view of the rapid global spread of COVID-19?](https://www.who.int/emergencies/diseases/novel-coronavirus-2019/question-and-answers-hub/q-a-detail/coronavirus-disease-covid-19-malaria-and-covid-19)**

As of March 2020, there have been reports of the suspension of insecticide-treated net (ITN) and indoor residual spraying (IRS) campaigns in several African countries due to concerns around exposure to COVID-19. Suspending such campaigns will leave many vulnerable populations at greater risk of malaria, particularly young children and pregnant women.

WHO strongly encourages countries**not to suspend**the planningfor – or implementation of – vector control activities, including ITN and IRS campaigns, while ensuring these services are delivered using [best practices to protect health workers](https://www.who.int/emergencies/diseases/novel-coronavirus-2019/technical-guidance/health-workers) and communities from COVID-19 infection. Modifications of planned distribution strategies may be needed to minimize exposure to the coronavirus.

WHO commends the leaders of Benin, Chad, the Central African Republic, the Democratic Republic of the Congo, Mali, Niger, Sierra Leone and Uganda for committing to move forward with ITN campaigns during the pandemic. Other countries are adapting their net distribution strategies to ensure households receive the nets as quickly and safely as possible.

Together with partners, WHO has developed guidance to ensure that those suffering from malaria can safely receive the care they need in COVID-19 settings.  [*Tailoring malaria interventions in the COVID-19 response*](https://www.who.int/publications/m/item/tailoring-malaria-interventions-in-the-covid-19-response) includes guidance on the prevention of infection through vector control and chemoprevention, testing, treatment of cases, clinical services, supply chain and laboratory activities. The document is consistent with broader [WHO guidance](https://www.who.int/publications-detail-redirect/covid-19-operational-guidance-for-maintaining-essential-health-services-during-an-outbreak) on how to maintain essential health services during the pandemic

228、**[Should WHO-recommended preventive therapies be maintained in sub-Saharan Africa?](https://www.who.int/emergencies/diseases/novel-coronavirus-2019/question-and-answers-hub/q-a-detail/coronavirus-disease-covid-19-malaria-and-covid-19)**

Yes, delivery of intermittent preventive treatment in pregnancy (IPTp), seasonal malaria chemoprevention (SMC), and intermittent preventive treatment in infants (IPTi) **should be maintained** provided that [best practices](https://www.who.int/emergencies/diseases/novel-coronavirus-2019/technical-guidance/health-workers) for protecting health workers – and other front-line workers – from COVID-19 are followed. Ensuring access to these and other core malaria prevention tools saves lives and is an important strategy for reducing the strain on health systems in the context of the COVID-19 response.

[*Tailoring malaria interventions in the COVID-19 response*](https://www.who.int/publications/m/item/tailoring-malaria-interventions-in-the-covid-19-response)*,*developed by WHO and partners*,*includes guidance on how to deliver preventive therapies for pregnant women and young children in ways that protect health workers and communities against potential COVID-19 transmission.

229、**[Are there any changes to WHO guidance with respect to malaria diagnosis and treatment?](https://www.who.int/emergencies/diseases/novel-coronavirus-2019/question-and-answers-hub/q-a-detail/coronavirus-disease-covid-19-malaria-and-covid-19)**

WHO guidance remains the same. Countries **should not scale back** efforts to detect and treat malaria; doing so would seriously undermine the health and well-being of millions of people infected with a potentially life-threatening disease.

As signs and symptoms of malaria and COVID-19 can overlap (such as a fever), public health messages will need to be adapted in malaria-endemic settings so that people who have a fever are encouraged to seek immediate treatment rather than stay at home; without prompt treatment, a mild case of malaria can rapidly progress to severe illness and death.

230、**[What additional special measures may be needed in the context of COVID-19?](https://www.who.int/emergencies/diseases/novel-coronavirus-2019/question-and-answers-hub/q-a-detail/coronavirus-disease-covid-19-malaria-and-covid-19)**

In addition to routine approaches to malaria control, there may be a case for special measures in the context of the COVID-19 pandemic – such as a temporary return to presumptive malaria treatment, or the use of mass drug administration – which have proved useful in some previous emergencies.

Presumptive malaria treatment refers to treatment of a suspected malaria case without the benefit of diagnostic confirmation (e.g. through a rapid diagnostic test). This approach is typically reserved for extreme circumstances, such as disease in settings where prompt diagnosis is no longer possible.

Mass drug administration (MDA) is a WHO-recommended approach for rapidly reducing malaria mortality and morbidity during epidemics and in complex emergency settings. Through MDA, all individuals in a targeted population are given antimalarial medicines – often at repeated intervals – regardless of whether or not they show symptoms of the disease.

Such special measures should only be adopted after careful consideration of 2 key aims: lowering malaria-related mortality and keeping health workers and communities safe. WHO is exploring concrete proposals for when and how to activate such measures; guidance will be published in due course.

231、**[What are the key considerations for countries that are working to eliminate malaria or prevent re-establishment of transmission?](https://www.who.int/emergencies/diseases/novel-coronavirus-2019/question-and-answers-hub/q-a-detail/coronavirus-disease-covid-19-malaria-and-covid-19)**

All of the considerations described above apply to malaria-eliminating countries and those preventing re-establishment of the disease: efforts must be sustained to prevent, detect and treat malaria cases while preventing the spread of COVID-19 and ensuring the safety of those who deliver the services. Countries that are nearing malaria elimination must protect their important gains and avoid malaria resurgences. Countries that have eliminated malaria must remain vigilant for any imported cases of malaria that may be occurring to prevent reintroduction of the disease.

232、**[Why is WHO particularly concerned about the spread of COVID-19 in malaria-affected areas?](https://www.who.int/emergencies/diseases/novel-coronavirus-2019/question-and-answers-hub/q-a-detail/coronavirus-disease-covid-19-malaria-and-covid-19)**

Experience from previous disease outbreaks has shown the disruptive effect on health service delivery and the consequences for diseases such as malaria. The 2014-2016 Ebola outbreak in Guinea, Liberia and Sierra Leone, for example, undermined malaria control efforts and led to a massive increase in malaria-related illness and death in the 3 countries.

A [modelling analysis](https://www.who.int/publications-detail-redirect/9789240004641) from WHO and partners, published on [23 April](https://www.who.int/news/item/23-04-2020-who-urges-countries-to-move-quickly-to-save-lives-from-malaria-in-sub-saharan-africa), found that the number of malaria deaths in sub-Saharan Africa could double this year alone if there are severe disruptions in access to insecticide-treated nets and antimalarial medicines due to COVID-19. These projections reinforce the critical importance of sustaining efforts to prevent, detect and treat malaria during the pandemic.

In all regions, protective measures should be utilized to minimize the risk of COVID-19 transmission between patients, communities and health providers. WHO and partners have developed [guidance](https://www.who.int/publications/m/item/tailoring-malaria-interventions-in-the-covid-19-response)on how to safely maintain malaria prevention and treatment services in COVID-19 settings.

233、**[Have there been disruptions in the global supply of key malaria-related commodities as a result of the COVID-19 pandemic?](https://www.who.int/emergencies/diseases/novel-coronavirus-2019/question-and-answers-hub/q-a-detail/coronavirus-disease-covid-19-malaria-and-covid-19)**

Yes. Since the early days of the pandemic, there have been reports of disruptions in the supply chains of essential malaria commodities – such as long-lasting insecticidal nets, rapid diagnostic tests and antimalarial medicines – resulting from lockdowns and from a suspension of the importation and exportation of goods in response to COVID-19. WHO and partners are working together to ensure the availability of key malaria control tools, particularly in countries with a high burden of the disease, and that efforts to limit the spread of COVID-19 do not compromise access to malaria prevention, diagnosis and treatment services.

234、**[What is WHO’s position on the use of chloroquine and hydroxychloroquine in the context of the COVID-19 response?](https://www.who.int/emergencies/diseases/novel-coronavirus-2019/question-and-answers-hub/q-a-detail/coronavirus-disease-covid-19-malaria-and-covid-19)**

WHO is actively following the ongoing clinical trials that are being conducted in response to COVID-19, including the more than 80 studies looking at the use of chloroquine and its derivative, hydroxychloroquine, for treatment and/or prevention.

To date, 3 large randomized controlled trials, including the WHO Solidarity trial, have failed to show that the use of hydroxychloroquine among hospitalized patients infected with COVID-19 can prevent death or disease progression. Additionally, 3 trials of patients with mild or moderate disease failed to show a significant benefit in prevention of respiratory failure through the use of hydroxychloroquine. Thus, there is now growing evidence that hydroxychloroquine is not an effective treatment for COVID-19. This evidence will inform the next update of WHO guidance on therapeutics.

Studies on the use of chloroquine or hydroxychloroquine to prevent individuals, particularly those at high risk such as health care workers, from contracting COVID-19 are ongoing. Currently, there is insufficient evidence to assess the protective efficacy of either of these medicines for the prevention of COVID-19 infection or disease.

WHO cautions physicians against administering these unproven treatments to patients with COVID-19 outside the context of a clinical trial. Individuals are also advised against self-medicating with these drugs.

For public health emergencies, WHO has a systematic and transparent process for research and development (R&D), including for clinical trials of drugs. The[WHO R&D Blueprint](https://www.who.int/teams/blueprint/covid-19)for COVID-19, initiated on 7 January 2020, aims to fast-track the availability of effective tests, vaccines and medicines that can be used to save lives and avert large-scale crises.

235、**[What is WHO’s position on the use of Artemisia plant material for the prevention or treatment of malaria and/or COVID-19?](https://www.who.int/emergencies/diseases/novel-coronavirus-2019/question-and-answers-hub/q-a-detail/coronavirus-disease-covid-19-malaria-and-covid-19)**

The most widely used antimalarial treatments, artemisinin-based combination therapies (ACTs), are produced using the pure artemisinin compound extracted from the plant *Artemisia annua*. There have been reports that products or extracts (e.g. herbal teas or tablets) made from *Artemisia* plant material may have a preventive or curative effect on COVID-19.

However, available in vitro data suggests that purified artemisinin compounds or *A. annua* plant product or extracts do not have an appreciable effect against COVID-19 at concentrations that could be safely achieved in humans. As such, current evidence does not support the use of artemisinins or *A. annua* products or extracts as an antiviral for COVID-19.

WHO urges extreme caution over reports touting the efficacy of such products. As explained in a [WHO position statement](https://www.who.int/publications-detail-redirect/WHO-CDS-GMP-2019.14), there is no scientific evidence base to support the use of non-pharmaceutical forms of *Artemisia* for the prevention or treatment of malaria. There is also no evidence to suggest that COVID-19 can be prevented or treated with products made from *Artemisia*-based plant material.

236、**[What is WHO doing to support malaria-affected countries in the context of COVID-19?](https://www.who.int/emergencies/diseases/novel-coronavirus-2019/question-and-answers-hub/q-a-detail/coronavirus-disease-covid-19-malaria-and-covid-19)**

The WHO Global Malaria Programme is [leading a cross-partner effort](https://www.who.int/malaria/areas/epidemics_emergencies/covid-19/who-gmp-response-covid-19.pdf) to mitigate the negative impact of the coronavirus in malaria-affected countries and, where possible, contribute towards a successful COVID-19 response. The work is being carried out in close collaboration with colleagues based at WHO headquarters, regional offices and country level.

In March 2020, before the pandemic had secured a strong footing in Africa, WHO sounded an [urgent call](https://www.who.int/news/item/25-03-2020-who-urges-countries-to-ensure-the-continuity-of-malaria-services-in-the-context-of-the-covid-19-pandemic) for maintaining core malaria prevention and treatment services while protecting health workers and communities against COVID-19 transmission. The findings of a [modelling analysis](https://www.who.int/publications-detail-redirect/9789240004641) from WHO and partners, published in April, reinforced the WHO call for continuity of malaria services during then pandemic.

In collaboration with partners, WHO developed technical guidance for countries on how to safely maintain malaria prevention and treatment in COVID-19 settings.[*Tailoring malaria interventions in the COVID-19 response*](https://www.who.int/publications/m/item/tailoring-malaria-interventions-in-the-covid-19-response)is consistent with broader [WHO guidance](https://www.who.int/publications-detail-redirect/covid-19-operational-guidance-for-maintaining-essential-health-services-during-an-outbreak) on how to maintain essential health services during the pandemic.

#### [Coronavirus disease (COVID-19): Masks（237-246）](https://www.who.int/emergencies/diseases/novel-coronavirus-2019/question-and-answers-hub/q-a-detail/coronavirus-disease-covid-19-masks)

237、**[Why should people wear masks?](https://www.who.int/emergencies/diseases/novel-coronavirus-2019/question-and-answers-hub/q-a-detail/coronavirus-disease-covid-19-masks)**

Wearing a mask reduces the spread of respiratory illnesses within the community by  reducing the number of infectious particles that may be inhaled or exhaled. These particles may be spread when an infected individual talks, sings, shouts, coughs, or sneezes (even if not symptomatic).

Hence, masks may provide protection to the wearer and those around them.

Masks are one component of a package of prevention and control measures to limit the spread of COVID-19. In addition to mask wearing, these measures include avoiding crowded spaces, avoiding poorly ventilated spaces and improving ventilation in indoor spaces, keeping a distance, hand hygiene, respiratory etiquette - covering your mouth and nose with a bent elbow or a tissue when you cough or sneeze, getting vaccinated and staying up to date with booster doses.

Be supportive of others wearing a mask if they choose to.

238、**[When should masks be worn by the public?](https://www.who.int/emergencies/diseases/novel-coronavirus-2019/question-and-answers-hub/q-a-detail/coronavirus-disease-covid-19-masks)**

To help reduce the spread of COVID-19, WHO recommends wearing a mask:

- When in a crowded, enclosed or poorly ventilation area (eg. you are unable to maintain distance). If you have any doubts regarding the quality of ventilation, wear a mask.
- If you have or think you might have COVID-19, when sharing a space with others:
  - If you feel unwell and have any of the signs and symptoms suggestive of COVID-19
  - If you recently tested positive for COVID-19 with or without symptoms
  - If you recently had contact (exposure) to someone who has tested positive for or displays symptoms of COVID-19
- When sharing a space with someone who has symptoms of COVID-19 or is COVID-19 positive
- When you are in a public space, and you are at *high risk of becoming seriously ill with COVID-19 or dying*. For example, if you have underlying health conditions (such as diabetes, heart or lung disease, cancer, immunosuppression, history of stroke and/or asthma) or if you are 60 years or older.

In other situations, not included in the above, you should assess your individual risk to determine if a mask should be worn. When deciding to wear a mask or not, the following should be considered:

- If COVID-19 infections are increasing in your community or there is an increase in those being hospitalized for COVID-19
- When your community has low levels of population immunity (such as low vaccination coverage)
- The overall status of your health

239、**[What kind of mask should the public wear?](https://www.who.int/emergencies/diseases/novel-coronavirus-2019/question-and-answers-hub/q-a-detail/coronavirus-disease-covid-19-masks)**

There are three types of masks that WHO recommends for the public:

- disposable medical masks*;
- non-medical masks that comply with standards for safety and efficacy and can be washed prior to reuse;
- if the above options are not available, other types of well-fitting non-medical masks are an acceptable option (according to local policies).

*Look for disposable medical masks labelled with any of the following standard certifications: ASTM F2100, EN 14683, YY 0469, YY/T 0969, GB 19083, or ASTM F3502. For more information on acceptable mask type, see the guideline, [*infection prevention and control in the context of COVID-19: a living guideline*](https://app.magicapp.org/#/guideline/Lr2a8L)

240、**[How do I choose what fabric mask to buy, or ideal fit and fabrics if I make my own?](https://www.who.int/emergencies/diseases/novel-coronavirus-2019/question-and-answers-hub/q-a-detail/coronavirus-disease-covid-19-masks)**

Filtration, breathability and fit are all important.

If you purchase a non-medical mask, check the labels to make sure it complies with the ASTM F3502 standard or CEN/TS17553.

If you choose to make your own mask, it should be made of three layers of fabric:

- Inner layer of absorbent material, such as cotton.
- Middle layer of non-woven non-absorbent material, such as polypropylene.
- Outer layer of non-absorbent material, such as polyester or polyester blend.

*Watch*[*the video*](https://www.who.int/emergencies/diseases/novel-coronavirus-2019/advice-for-public/when-and-how-to-use-masks)*on WHO’s recommended fabric mask materials and composition for more information.*

*For either type of mask, you should choose the one that fits your face well - cover your nose, cheeks and chin without leaving gaps on the sides. Masks with vents or exhalation valves are not advised because they allow unfiltered breath to escape the mask.*

241、**[How should I put on, wear and take off a medical mask?](https://www.who.int/emergencies/diseases/novel-coronavirus-2019/question-and-answers-hub/q-a-detail/coronavirus-disease-covid-19-masks)**

How to put on and take off a medical mask:

- Before touching the mask, clean your hands with an alcohol-based hand rub or soap and water.
- Inspect the mask for tears or holes; do not use a mask that has previously been worn or is damaged.
- Verify which side is the top – this is usually where the metal strip is.
- Then, identify the inside of the mask, which is usually the white side.
- Place the mask on your face covering your nose, mouth and chin, making sure that there are no gaps between your face and the mask. Place the straps behind your head or ears. Do not cross the straps because this can cause gaps on the side of the mask.
- Pinch the metal strip so it moulds to the shape of your nose.
- Remember, do not touch the front of the mask while using it to avoid contamination; if you accidentally touch it, clean your hands.

How to take off a medical mask:

- Before touching the mask, clean your hands with an alcohol-based hand rub or soap and water.
- Remove the straps from behind the head or ears, without touching the front of the mask.
- As you remove the mask, lean forward and pull the mask away from your face.
- Medical masks are for single use only; discard the mask immediately, preferably into a closed bin.
- Clean your hands after touching the mask.
- Be aware of the condition of the mask; replace it if it gets soiled or damp.

242、**[How should I wear and clean a fabric mask?](https://www.who.int/emergencies/diseases/novel-coronavirus-2019/question-and-answers-hub/q-a-detail/coronavirus-disease-covid-19-masks)**

How to put on and wear a fabric mask:

- Before touching the mask, clean your hands with an alcohol-based hand rub or soap and water.
- Inspect the mask for tears or holes, do not use a mask that is damaged.
- Adjust the mask to cover your mouth, nose, and chin, leaving no gaps on the sides.
- Place the straps behind your head or ears. Do not cross the straps because this can cause gaps on the side of your face.
- Avoid touching the mask while wearing it. If you touch it, clean your hands.
- Change your mask if it gets dirty or wet.

How to take off and store a fabric mask:

- Clean your hands before taking off the mask.
- Take off the mask by removing it from the ear loops, without touching the front of the mask.
- If your fabric mask is not dirty or wet and you plan to reuse it, put it in a clean resealable bag. If you need to use it again, hold the mask at the elastic loops when removing it from the bag. Clean your mask once a day.
- Clean your hands after removing the mask.

How to clean a fabric mask:

- Wash fabric masks in soap or detergent and preferably hot water (at least 60 degrees Centigrade/140 degrees Fahrenheit) at least once a day.
- If it is not possible to wash the mask in hot water, then wash it in soap/detergent and room temperature water, followed by boiling the mask for 1 minute.

Watch our [‘How to wear a fabric mask’ video](https://www.youtube.com/watch?v=ciUniZGD4tY&feature=emb_logo) for a demonstration.

243、**[Should I wear a mask while exercising?](https://www.who.int/emergencies/diseases/novel-coronavirus-2019/question-and-answers-hub/q-a-detail/coronavirus-disease-covid-19-masks)**

Even when you’re in an area with active COVID-19 transmission, masks should not be worn during vigorous physical activity because of the risk of reducing your breathing capacity. No matter how intensely you exercise, keep at least 1 metre away from others, and if you’re indoors, make sure there is adequate ventilation.


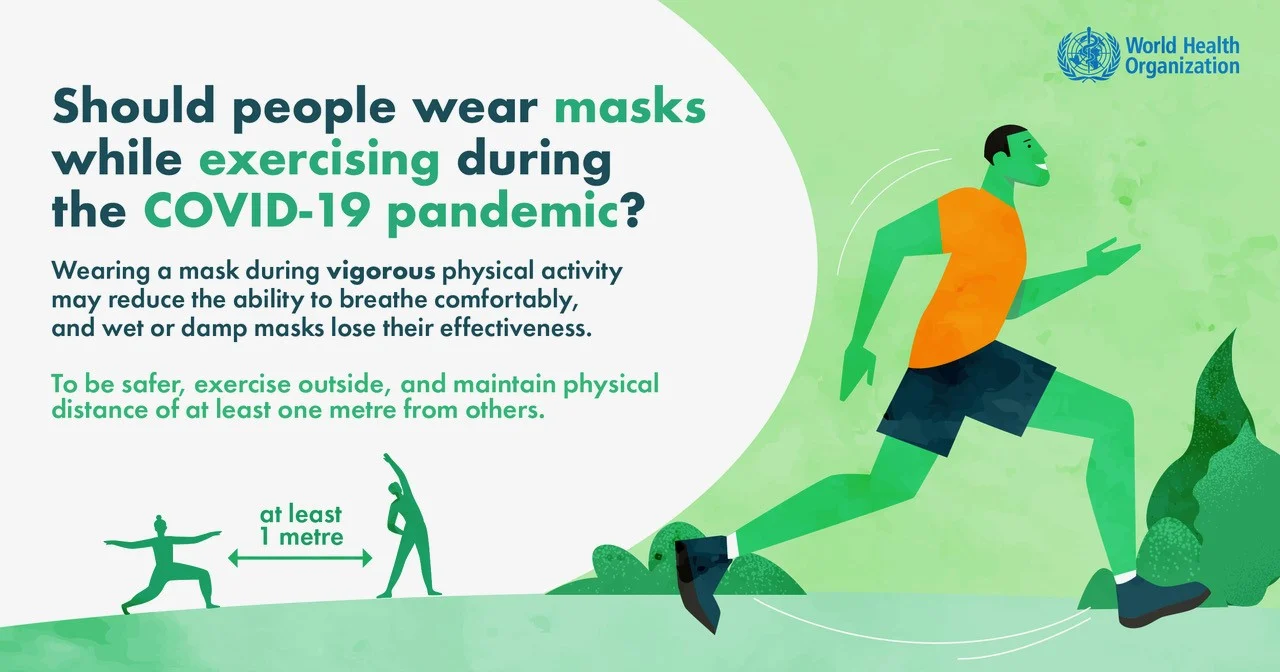


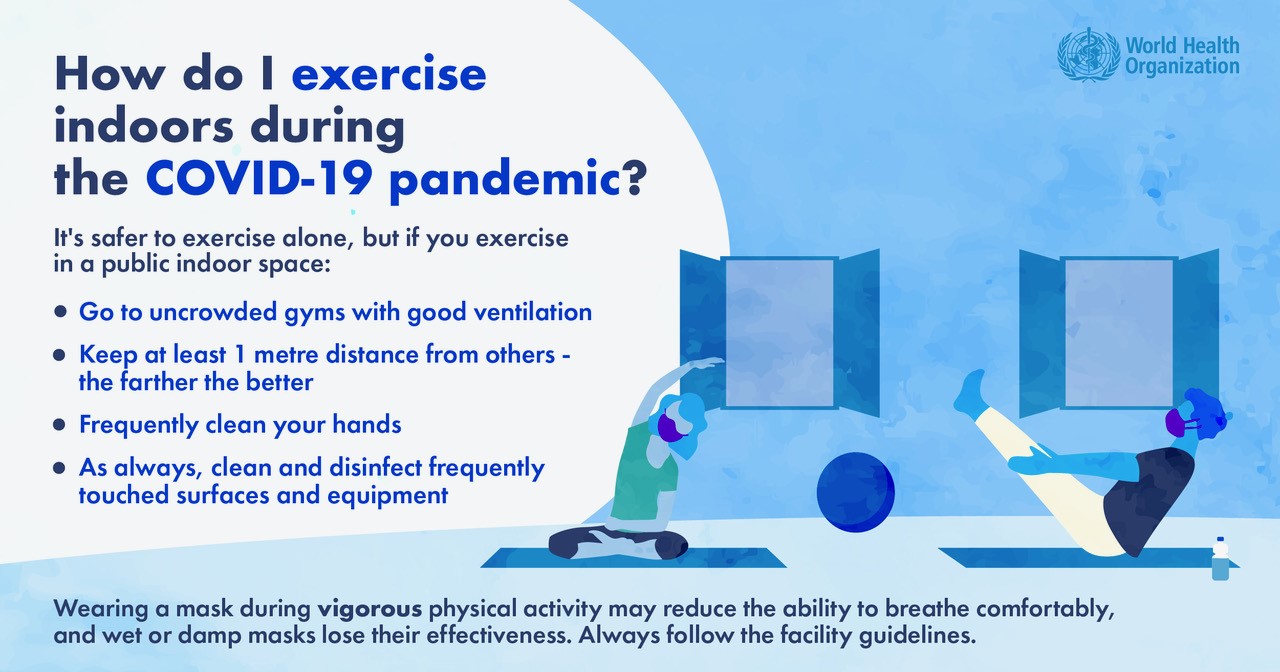


244、**[When should health workers wear a respirator in the context of COVID-19?](https://www.who.int/emergencies/diseases/novel-coronavirus-2019/question-and-answers-hub/q-a-detail/coronavirus-disease-covid-19-masks)**

A r**espirator or a medical mask**should be worn by health workers along with other personal protective equipment (PPE) - gown, gloves and eye protection - before entering a room where there is a patient with suspected or confirmed COVID-19.

- Respirators or medical masks should be worn throughout their shifts, apart from when eating, drinking or needing to change the respirator or medical mask for specific reasons.
- Health workers must remember to combine hand hygiene with any time they touch their respirator or medical mask or face, before and after putting on and removing them, as well as before they touch them to readjust them.
- Health workers should continue to physically distance and avoid unnecessary close contact with colleagues and others in the healthcare facility when not providing patient care.

**Respirators**should be worn in the following situations:

- A respirator should always be worn along with other PPE (gown, gloves, eye protection) by health workers performing aerosol-generating procedures (AGP) and by health workers on duty in settings where AGP are regularly performed on patients with suspected or confirmed COVID-19, such as intensive care units, semi-intensive care units or emergency departments.
- In healthcare facilities where ventilation is known to be poor or cannot be assessed, or the ventilation system is not properly maintained.
- Based on health workers' values and preferences and on their perception of what offers the highest protection possible to prevent SARS-CoV-2 infection.

Further resources:

- Healthcare workers can consult our [OpenWHO](https://openwho.org/) course on how to put on and remove personal protective equipment.
- Read our guidance on ‘[Infection prevention and control during health care when coronavirus disease (‎COVID-19)‎ is suspected or confirmed](https://www.who.int/publications-detail-redirect/WHO-2019-nCoV-IPC-2021.1)’.

245、**[What is the difference between medical masks and respirators, like N95s?](https://www.who.int/emergencies/diseases/novel-coronavirus-2019/question-and-answers-hub/q-a-detail/coronavirus-disease-covid-19-masks)**

Medical masks (also known as surgical masks) are:

- composed of 3 layers of synthetic nonwoven materials
- configured to have filtration layers sandwiched in the middle
- available in different thicknesses
- have various levels of fluid-resistance and filtration

Respirators (also known as filtering facepiece respirators – FFP) are available at different performance levels such as FFP2, FFP3, N95, N99, N100.

Medical masks and respirator masks are similar in their filtration value. However, respirators also have a tight fit around the wearer face as the model and size of the respirator is specific to the wearer to ensure an adequate seal. Respirator masks are designed to protect health workers who provide care to COVID-19 patients in settings and areas where aerosol generating procedures are undertaken. They are also recommended for health workers providing care to suspected or confirmed COVID-19 patients in settings where ventilation is known to be poor or cannot be assessed or the ventilation system is not properly maintained.

Health workers should be fit tested before using a respirator to ensure that they are wearing the correct size. Wearing a loose-fitting respirator will not offer the same level of protection to the wearer as it may allow small particles to get inside the mask where there are gaps, for example through the side.

246、**[In healthcare facilities, when should medical mask be worn?](https://www.who.int/emergencies/diseases/novel-coronavirus-2019/question-and-answers-hub/q-a-detail/coronavirus-disease-covid-19-masks)**

When health workers are caring for COVID-19 patients, they should wear a respirator or a medical mask.

If COVID-19 is circulating, visitors, along with health workers and caregivers, should wear awell-fitting medical maskat all times when caring for non-COVID-19 patients and in all common areas, even if physical distancing can be maintained.

Health workers, caregivers and visitors must remember to combine hand hygiene with any time they touch their medical mask or face, before and after putting on and removing them, as well as before they touch them to readjust them.

Health workers should continue to physically distance and avoid close contact with colleagues and others in the facility when not providing patient care.

Inpatients are not required to wear a medical mask in general. A mask may be required if physical distancing of at least 1 metre cannot be maintained (e.g., during examinations or bedside visits) or when the patient is outside of their care area (e.g., when being transported), provided the patient is able to tolerate the mask and there are no contraindications.

#### [Coronavirus disease (COVID-19): Mass gatherings（247-257）](https://www.who.int/emergencies/diseases/novel-coronavirus-2019/question-and-answers-hub/q-a-detail/coronavirus-disease-covid-19-mass-gatherings)

247、**[How large does a meeting or event need to be in order to be a “mass gathering”?](https://www.who.int/emergencies/diseases/novel-coronavirus-2019/question-and-answers-hub/q-a-detail/coronavirus-disease-covid-19-mass-gatherings)**

High profile international sporting events such as the Olympics or World Cups as well as international religious events such as the Hajj count as mass gatherings. However, lower profile conferences and events can also meet WHO’s definition of a mass gathering. An event counts as a “mass gatherings” if the number of people it brings together is so large that it has the potential to strain the planning and response resources of the health system in the community where it takes place. You need to consider the location and duration of the event as well as the number of participants. For example, if the event takes place over several days in a small island state where the capacity of the health system is quite limited then even an event with just a few thousand participants could place a big strain on the health system and then be considered a “mass gathering” event. Conversely, if the event is held in a big city in a country with a large, well-resourced health system and lasts just a few hours, the event may not constitute a “mass gathering” event.

248、**[Does WHO recommend that all international mass gatherings be cancelled because of COVID-19?](https://www.who.int/emergencies/diseases/novel-coronavirus-2019/question-and-answers-hub/q-a-detail/coronavirus-disease-covid-19-mass-gatherings)**

No. As each international mass gathering is different, the factors to consider when determining if the event should be cancelled may also differ. Any decision to change a planned international gathering should be based on a careful assessment of the risks and how they can be managed, and the level of event planning. The assessment should involve all stakeholders in the event, and in particular the health authorities in the country or community where the event is due to take place. These authorities and stakeholders are in the best position to assess the level of stress the event might place on the local health system and emergency services – and whether this level of stress is acceptable in the current situation.

It is WHO’s view that all countries with community transmission should seriously consider postponing or reducing mass gatherings that bring people together and have the potential to amplify disease and support the recommended best practice of physical distancing. Any decision will be supported through the use of WHO tools, in particular the Risk Assessment for Mass Gatherings during COVID-19.

If movement restrictions and further national measures have been established in the country, the WHO RA does not apply. However, when the process of re-opening/conducting mass gatherings is being considered post movement restrictions, it will be key to ensure any decisions are based on a risk assessment, such as the WHO Mass gatherings COVID-19 risk assessment.

249、**[What factors should organizers and health authorities look at when assessing whether the risks are acceptable or not?](https://www.who.int/emergencies/diseases/novel-coronavirus-2019/question-and-answers-hub/q-a-detail/coronavirus-disease-covid-19-mass-gatherings)**

For countries not currently known to be experiencing community transmission of COVID-19, the priority consideration will be whether the planned mass gathering event substantially increases the risk of the virus entering the country and becoming established, as well as the risk for participants to importing infection back to their home country and further increasing global spread. In making this assessment, the organizers and their national or local health authorities should recognize that the risk of imported cases of COVID-19 is naturally linked to international travel. They should also recognize that it is neither realistic or desirable to aim for zero risk. When organizers and health authorities are determining whether to hold a mass gathering, they should determine what is an acceptable risk and what additional measures should be implemented to mitigate the risks.

For countries where COVID-19 has already started to spread in the community, key consideration will be:

- aiming at containing or at least slowing down the spread of the virus in the local community/country.
- preventing participants from other countries being infected with COVID-19

In each case the risk should be considered in the context of the known features of COVID-19, its severity, its transmissibility and the effectiveness of measures to prevent or reduce transmission. The strain already placed on the local health system in responding to COVID-19 outbreak(s), and the additional strain the mass gathering might place on the system also need to be taken into account.

You can find more advice on what to look at in the WHO document *Key planning recommendations for Mass Gatherings in the context of the current COVID-19 outbreak* of 14 February 2020. See: [https://www.who.int/publications-detail/key-planning-recommendations-for-mass-gatherings-in-the-context-of-the-current-covid-19-outbreak](https://www.who.int/publications-detail-redirect/key-planning-recommendations-for-mass-gatherings-in-the-context-of-the-current-covid-19-outbreak)

250、**[What if my organization does not have the expertise to assess the risks COVID-19 poses for our planned mass gathering?](https://www.who.int/emergencies/diseases/novel-coronavirus-2019/question-and-answers-hub/q-a-detail/coronavirus-disease-covid-19-mass-gatherings)**

The national and local public health authorities in the country where you plan to hold the mass gathering will most likely know how to conduct a health risk assessment. If there is a WHO Country Office there they may also be able to provide some expert support. So too might the WHO Regional Office in your part of the world. You can find the names and contact details of the WHO Regional Offices at <https://www.who.int/about/who-we-are/regional-offices>

251、**[If we go ahead with an international mass gathering, what can we do to reduce the risk of participants catching COVID-19?](https://www.who.int/emergencies/diseases/novel-coronavirus-2019/question-and-answers-hub/q-a-detail/coronavirus-disease-covid-19-mass-gatherings)**

Promote hand washing, respiratory hygiene and social distancing at the event. Make sure you have emergency contact details for all participants, including where they are staying during the event. You should make it clear to them that this information will be shared with the local public health authorities to enable rapid contact tracing if a participant at the event becomes ill with COVID-19. The event organisers need to have an agreed preparedness plan in case one or more participants become ill with COVID-19 symptoms. This should include rapid isolation of the ill person and their safe transfer to a local health facility. You should consider whether the number of participants at the event could be reduced, making available participation by video or teleconference and possibly screening participants for COVID-19 symptoms (cough, fever, malaise) at points of entry to the venue. You can find advice on how individual participants can protect themselves and their loved ones from COVID-19 at: <https://www.who.int/emergencies/diseases/novel-coronavirus-2019/advice-for-public>

252、**[Where can I find more advice on assessing and managing health risks around international mass gatherings?](https://www.who.int/emergencies/diseases/novel-coronavirus-2019/question-and-answers-hub/q-a-detail/coronavirus-disease-covid-19-mass-gatherings)**

WHO has produced guidance and also a training course on how to plan for a mass gathering. The guidance and the course both look at how to conduct a risk assessment, plan for and manage health risks in partnership with the local authorities: <https://www.who.int/emergencies/diseases/novel-coronavirus-2019/technical-guidance/points-of-entry-and-mass-gatherings> You can find advice to give individual participants on how to protect themselves and their loved ones from COVID-19 at: <https://www.who.int/emergencies/diseases/novel-coronavirus-2019/advice-for-public>

253、**[What should be the criteria for excluding an athlete or other accredited participant from competing?](https://www.who.int/emergencies/diseases/novel-coronavirus-2019/question-and-answers-hub/q-a-detail/coronavirus-disease-covid-19-mass-gatherings)**

Agreed health criteria are needed for the exclusion of athletes from competing and for allowing them to return to competition wherever appropriate. These should be consistent with public health guidelines for the general population, with risk profiles based on recent travel to high-risk community transmission zones, recent contact with individuals diagnosed with or suspected of having COVID-19, current symptomatology, etc. Athletes who are feeling unwell should not participate in events.

If testing of athletes has been agreed with the local public health service there is a need to consider test availability, time taken for receipt of test results and host-country health services capacity.

Criteria for exclusion of officials/judges from competition and for allowing them to return to competition where appropriate should follow [guidance and criteria for the workplace](https://www.who.int/docs/default-source/coronaviruse/advice-for-workplace-clean-19-03-2020.pdf?sfvrsn=bd671114_6).

Requirements for the attendance of all athletes and other accredited participants should be put in place:

–   health check: travel declaration and temperature screening

–   contact tracing protocols ready (with all details of those present)

–   allowing extra spacing (physical distancing)

–   following all hygiene precautions.

Isolation procedures for athletes and other accredited participants (e.g. field-of-play staff, medical teams, officials who have close contact with the athletes) who feel unwell or become symptomatic should be clear and documented and rehearsed prior to holding the event, especially for higher risk sports, e.g. contact sports.

254、**[Should event organizers arrange screening at venues beyond national government requirements for point of entry (PoE)?](https://www.who.int/emergencies/diseases/novel-coronavirus-2019/question-and-answers-hub/q-a-detail/coronavirus-disease-covid-19-mass-gatherings)**

Temperature screening alone, at exit or entry, is not an effective way to stop international spread, since infected individuals may be in incubation period, may not express apparent symptoms early on in the course of the disease, or may dissimulate fever through the use of antipyretics; in addition, such measures require substantial investments for what may bear little benefits. It is more effective to provide prevention recommendation messages to travellers and to collect health declarations at arrival, with travellers’ contact details, to allow for a proper risk assessment and a possible contact tracing of [incoming travellers](https://apps.who.int/iris/bitstream/handle/10665/331512/WHO-2019-nCoV-POEmgmt-2020.2-eng.pdf).

Event organizers should also consider the capacity to undertake this and the management of suspected cases. However, sporting organizations may have criteria for participation that are stricter than government requirements for Ports of Entry into the country that are based on event risk assessment, including organizers' healthcare capacity.

Any additional screening questionnaires for participation in events need to be aligned with public health agencies in the jurisdiction. There is a need to consider how to manage different groups – athletes, officials and spectators, and the large numbers of individuals likely to arrive at a sporting event from many oversea countries – and to follow both host country and [WHO travel advice](https://www.who.int/emergencies/diseases/novel-coronavirus-2019/travel-advice) and to assess whether there is difficulty in obtaining accurate information about the travel history of those individuals.

255、**[Should event organizers provide COVID-19 testing?](https://www.who.int/emergencies/diseases/novel-coronavirus-2019/question-and-answers-hub/q-a-detail/coronavirus-disease-covid-19-mass-gatherings)**

No, testing should be conducted in accordance with local health providers and national guidance.  Anyone unwell or symptomatic should not be allowed to participate in the event. Establish close collaboration with local public health authorities well before the event, to facilitate testing for athletes, officials or spectators who meet pre-agreed suspect cases definitions.

Consider host-country health service capacity to manage any COVID-19 activity, and other public health issues happening at the event over and above its own national pandemic response.

256、**[Are there additional safeguards event organizers can implement or recommend to athletes/officials/visitors in the context of COVID-19?](https://www.who.int/emergencies/diseases/novel-coronavirus-2019/question-and-answers-hub/q-a-detail/coronavirus-disease-covid-19-mass-gatherings)**

Follow standard preventative advice to the general public, including physical distancing, in the athletes’ village.

Also promote:

- signage, digital messaging to all participants and their entourages about COVID-19 and how to prevent infection
- regular disinfection and cleaning of surfaces, in venues and in personal spaces
- non-sharing of equipment and cleaning of equipment after each user.

Organizers are recommended to develop a checklist (include hand gels stations, hygiene facilities, etc.). See the event mitigation recommendations checklist for event organizers above.

257、**[What are the risks arising from public transport to the venue(s)?](https://www.who.int/emergencies/diseases/novel-coronavirus-2019/question-and-answers-hub/q-a-detail/coronavirus-disease-covid-19-mass-gatherings)**

The extra risk from participants and spectators travelling on public transport in a major city may not be significant compared to the ongoing risk to the local population using the transport all the time if physical distancing is not possible on public transport.

#### [Coronavirus disease (COVID-19): Post COVID-19 condition（258-268）](https://www.who.int/emergencies/diseases/novel-coronavirus-2019/question-and-answers-hub/q-a-detail/coronavirus-disease-(covid-19)-post-covid-19-condition)

258、**[What is post COVID-19 condition?](https://www.who.int/emergencies/diseases/novel-coronavirus-2019/question-and-answers-hub/q-a-detail/coronavirus-disease-(covid-19)-post-covid-19-condition)**

Post COVID-19 condition, also known as long COVID, refers to long-term symptoms that some people experience after they have had COVID-19. People who experience post COVID-19 condition sometimes refer to themselves as “long-haulers”.

These symptoms might persist from their initial illness or develop after their recovery. They can come and go or relapse over time.

The most common symptoms associated with post COVID-19 condition include fatigue, breathlessness and cognitive dysfunction (for example, confusion, forgetfulness, or a lack of mental focus or clarity). Post COVID-19 condition can affect a person’s ability to perform daily activities such as work or household chores.

259、**[How soon after getting COVID-19 can someone be diagnosed with post COVID-19 condition?](https://www.who.int/emergencies/diseases/novel-coronavirus-2019/question-and-answers-hub/q-a-detail/coronavirus-disease-(covid-19)-post-covid-19-condition)**

Post COVID-19 condition is usually diagnosed by a healthcare provider at least 3 months after a patient falls ill with COVID-19. This 3-month period allows healthcare providers to rule out the usual recovery period from an acute illness. Sometimes this recovery period can be long, especially if someone is very sick.

Symptoms should last for at least 2 months from when someone first falls ill for it to be considered as post COVID-19 condition.

Symptoms of post COVID-19 condition can persist from the initial illness or begin after recovery. The symptoms and effects of post COVID-19 condition can only be explained when other conditions with similar symptoms as post COVID-19 condition have been ruled out through a medical diagnosis. Symptoms may also change over time.

260、**[What are common symptoms of post COVID-19 condition (long COVID)?](https://www.who.int/emergencies/diseases/novel-coronavirus-2019/question-and-answers-hub/q-a-detail/coronavirus-disease-(covid-19)-post-covid-19-condition)**

Symptoms differ between people, and between adults and children.  Overall, the most common symptoms of post COVID-19 condition include:

- fatigue
- shortness of breath or difficulty breathing
- memory, concentration or sleep problems
- persistent cough
- chest pain
- trouble speaking
- muscle aches
- loss of smell or taste
- depression or anxiety
- fever.

People with post COVID-19 condition, also known as long COVID, may have difficulty functioning in everyday life. Their condition may affect their ability to perform daily activities such as work or household chores.

261、**[What should I do if I have had COVID-19 and am experiencing symptoms and effects like those described as post COVID-19 condition?](https://www.who.int/emergencies/diseases/novel-coronavirus-2019/question-and-answers-hub/q-a-detail/coronavirus-disease-(covid-19)-post-covid-19-condition)**

If you have ongoing symptoms after COVID-19, seek help from your healthcare provider. They will help you to determine the cause and provide you with the care you need to manage your symptoms.

At present, there are no proven drug treatments for post COVID-19 condition. This is an area of active research. However, some commonly available medications can alleviate symptoms. There are data suggesting that holistic care can help patients regain their physical, cognitive and emotional function and help to improve their quality of life.

262、**[Who is most at risk of developing post COVID-19 condition?](https://www.who.int/emergencies/diseases/novel-coronavirus-2019/question-and-answers-hub/q-a-detail/coronavirus-disease-(covid-19)-post-covid-19-condition)**

Anyone can develop post COVID-19 condition. Research suggests that approximately 10–20% of COVID-19 patients go on to develop prolonged symptoms that are associated with post COVID-19 condition. Current evidence doesn’t allow us to confidently know who is more likely to be affected, although certain problems (for example breathlessness) seem to be more common amongst those with more severe initial COVID-19, and more common in women.

263、**[If I had a confirmed case of COVID-19 and I’m still experiencing symptoms, how long would it take before I could be diagnosed with post COVID-19 condition?](https://www.who.int/emergencies/diseases/novel-coronavirus-2019/question-and-answers-hub/q-a-detail/coronavirus-disease-(covid-19)-post-covid-19-condition)**

According to the WHO definition, symptoms should last two months or more before a diagnosis of post COVID-19 condition can be made, as we know that normal recovery can take this long. It is important that other causes for ongoing symptoms are considered. For more information, see the [clinical case definition of post COVID-19 condition.](https://www.who.int/publications/i/item/WHO-2019-nCoV-Post_COVID-19_condition-Clinical_case_definition-2021.1)

264、**[How long does post COVID-19 condition last?](https://www.who.int/emergencies/diseases/novel-coronavirus-2019/question-and-answers-hub/q-a-detail/coronavirus-disease-(covid-19)-post-covid-19-condition)**

We cannot predict how long post COVID-19 condition will last for any given person. Most people experience improvement in their symptoms, but we know that lingering symptoms can last from weeks to months. Currently, it remains impossible to predict how long post COVID-19 condition may last for any given person.

265、**[What can I do to protect myself against post COVID-19 condition?](https://www.who.int/emergencies/diseases/novel-coronavirus-2019/question-and-answers-hub/q-a-detail/coronavirus-disease-(covid-19)-post-covid-19-condition)**

Taking measures to avoid COVID-19 infection is the most effective way to protect yourself against post COVID-19 condition. This includes public health and social measures that reduce your chances of getting infected. To keep you and your family safe remember to:

- continue to wash your hands regularly
- keep a distance from others
- wear a mask when in a crowded, enclosed or poorly ventilated area
- get vaccinated and stay up to date with booster doses
- practice respiratory etiquette; cough or sneeze into your bent elbow

266、**[Does getting vaccinated prevent post COVID-19 condition?](https://www.who.int/emergencies/diseases/novel-coronavirus-2019/question-and-answers-hub/q-a-detail/coronavirus-disease-(covid-19)-post-covid-19-condition)**

Research is ongoing. A vaccine’s ability to prevent post COVID-19 condition depends on its ability to prevent COVID-19 in the first place. The vaccines we use today are aimed at preventing severe disease and death from COVID-19. However, some people may still get infected with COVID-19 even after they are vaccinated.

267、**[If I have post COVID-19 condition, can I give it to others?](https://www.who.int/emergencies/diseases/novel-coronavirus-2019/question-and-answers-hub/q-a-detail/coronavirus-disease-(covid-19)-post-covid-19-condition)**

No, post COVID-19 condition cannot be passed to others.

268、**[How does post COVID-19 condition affect children and adolescents?](https://www.who.int/emergencies/diseases/novel-coronavirus-2019/question-and-answers-hub/q-a-detail/coronavirus-disease-(covid-19)-post-covid-19-condition)**

WHO expert group which includes patients, healthcare workers and researchers has been considering the impact of post-COVID-19 condition on children and adolescents. Findings of this report can be found [here](https://apps.who.int/iris/bitstream/handle/10665/366126/WHO-2019-nCoV-Post-COVID-19-condition-CA-Clinical-case-definition-2023.1-eng.pdf). A free webinar is available for more information: [Post COVID-19 Condition: Children and Young Persons (who.int)](https://www.who.int/news-room/events/detail/2022/08/17/default-calendar/post-covid-19-condition--children-and-young-persons)

Young children with COVID-19 mainly present with respiratory symptoms and are more likely to seek long-term medical care for a persistent cough. Children with post COVID-19 condition are also more likely to have fatigue, altered smell and anxiety than healthy children.

#### [Coronavirus disease (COVID-19): Pregnancy, childbirth and the postnatal period（269-276）](https://www.who.int/emergencies/diseases/novel-coronavirus-2019/question-and-answers-hub/q-a-detail/coronavirus-disease-covid-19-pregnancy-and-childbirth)

269、Pregnant women do not seem to be at higher risk of getting SARS-CoV-2, the virus that causes COVID-19. However, studies have shown an increased risk of developing severe COVID-19 if they are infected, compared with non-pregnant women of a similar age. COVID-19 during pregnancy has also been associated with an increased likelihood of preterm birth.

Pregnant women who are older, overweight or have pre-existing medical conditions such as hypertension (high blood pressure) and diabetes are at particular risk of serious outcomes of COVID-19.

It is important that pregnant women – and those around them – take precautions to protect themselves against COVID-19. If they become unwell (including with fever, cough or difficulty breathing), they should seek urgent medical advice from a health worker.

视频：https://youtu.be/qA6-wL2lcUw

270: [**I’m pregnant. How can I protect myself against COVID-19?**](https://www.who.int/emergencies/diseases/novel-coronavirus-2019/question-and-answers-hub/q-a-detail/coronavirus-disease-covid-19-pregnancy-and-childbirth)

Pregnant women should take the [same precautions](https://www.who.int/emergencies/diseases/novel-coronavirus-2019/advice-for-public) to avoid COVID-19 infection as other people. Measures to protect yourself – and those around you – include:

- getting vaccinated;
- keeping space between yourself and other people, and avoiding crowded spaces;
- keeping rooms well ventilated;
- wearing a mask where it is not possible to keep sufficient physical distance from others;
- washing your hands frequently with an alcohol-based hand rub or soap and water; and
- practicing respiratory hygiene. This means covering your mouth and nose with your bent elbow or tissue when you cough or sneeze. Then dispose of the used tissue immediately.

If you have fever, cough or difficulty breathing, seek medical care early. Call before going to a health facility and follow the directions of your local health authority.

Pregnant women and women who have recently delivered should attend their routine care appointments according to local policies and following adapted measures to reduce possible transmission of the virus.

271、**[Should pregnant women be tested for COVID-19?](https://www.who.int/emergencies/diseases/novel-coronavirus-2019/question-and-answers-hub/q-a-detail/coronavirus-disease-covid-19-pregnancy-and-childbirth)**

Testing protocols and eligibility during pregnancy vary depending on where you live.

However, WHO recommendations are that pregnant women with symptoms of COVID-19 should be prioritized for testing. If they have COVID-19, they may need specialized care.

272、**[Can COVID-19 be passed from a woman to her unborn or newborn baby?](https://www.who.int/emergencies/diseases/novel-coronavirus-2019/question-and-answers-hub/q-a-detail/coronavirus-disease-covid-19-pregnancy-and-childbirth)**

Transmission of the virus while your baby is in the womb or during birth is possible, but very rare. Most babies won’t develop COVID-19 disease, and those who develop symptoms tend to recover quickly.

Babies can be infected after birth, so if you do get COVID-19, it is important for you and other caregivers to take all precautions to reduce the risks of passing the virus to the baby.

Babies can be placed skin-to-skin and breastfed if the mother is confirmed or suspected to have COVID-19. The numerous benefits of skin-to-skin contact and breastfeeding [substantially outweigh](https://www.who.int/news/item/16-03-2021-new-research-highlights-risks-of-separating-newborns-from-mothers-during-covid-19-pandemic) the potential risks of transmission and illness associated with COVID-19.

273、**[What care should be available during pregnancy, childbirth and the postnatal period?](https://www.who.int/emergencies/diseases/novel-coronavirus-2019/question-and-answers-hub/q-a-detail/coronavirus-disease-covid-19-pregnancy-and-childbirth)**

All pregnant and postpartum women and their newborns, including those with confirmed or suspected COVID-19 infections, have the right to high quality care before, during and after childbirth, including mental health care.

A safe and positive childbirth experience includes:

- being treated with respect and dignity
- having a companion of choice present during delivery
- clear communication by maternity staff
- appropriate pain relief strategies
- mobility in labour where possible, and birth position of choice.

If COVID-19 is suspected or confirmed, health workers should take all appropriate precautions to reduce risks of infection to themselves and others, including hand hygiene and appropriate use of protective clothing like gloves, gown and medical mask.

274、**[Do pregnant women with suspected or confirmed COVID-19 need to give birth by caesarean section?](https://www.who.int/emergencies/diseases/novel-coronavirus-2019/question-and-answers-hub/q-a-detail/coronavirus-disease-covid-19-pregnancy-and-childbirth)**

No. WHO advice is that caesarean sections should only be performed when medically justified.

The mode of birth should be individualized and based on a woman’s preferences alongside obstetric indications.

275、**[Can I touch and hold my newborn baby if I have COVID-19?](https://www.who.int/emergencies/diseases/novel-coronavirus-2019/question-and-answers-hub/q-a-detail/coronavirus-disease-covid-19-pregnancy-and-childbirth)**

Yes. Close contact and early, exclusive breastfeeding helps a baby to thrive. You should be supported to

- breastfeed safely, with good respiratory hygiene
- hold your newborn skin-to-skin
- share a room with your baby.

276、**[Can pregnant women get vaccinated against COVID-19?](https://www.who.int/emergencies/diseases/novel-coronavirus-2019/question-and-answers-hub/q-a-detail/coronavirus-disease-covid-19-pregnancy-and-childbirth)**

Yes, pregnant women [can be vaccinated](https://www.who.int/publications-detail-redirect/WHO-2019-nCoV-FAQ-Pregnancy-Vaccines-2022.1) against COVID-19.

COVID-19 vaccines offer strong protection against severe illness, hospitalization and death from COVID-19. Vaccination during pregnancy is important whenever there is risk of the disease, but especially for frontline health workers, people living in areas where there is high community transmission, and those with health conditions like hypertension (high blood pressure) and diabetes that add to risk of severe disease.

Some evidence also suggests that babies may receive protective benefits from the vaccine, in addition to the benefits for pregnant women.

Pregnant women in many countries around the world have now received COVID-19 vaccines, and no safety concerns have been identified related to their pregnancies or the health of their babies. None of the COVID-19 vaccines authorized to date use live viruses, which are more likely to pose risks during pregnancy.

#### [Coronavirus disease (COVID-19): Risks and safety for older people（277-286）](https://www.who.int/emergencies/diseases/novel-coronavirus-2019/question-and-answers-hub/q-a-detail/coronavirus-disease-covid-19-risks-and-safety-for-older-people)

277、**[What is COVID-19?](https://www.who.int/emergencies/diseases/novel-coronavirus-2019/question-and-answers-hub/q-a-detail/coronavirus-disease-covid-19-risks-and-safety-for-older-people)**

COVID-19 is a disease caused by a new coronavirus, which has not been previously identified in humans. In most cases, COVID-19 causes mild symptoms including dry cough, tiredness and fever, though fever may not be a symptom for some older people. Other mild symptoms include aches and pains, nasal congestion, runny nose, sore throat or diarrhoea. Some people become infected but don’t develop any symptoms and don't feel unwell. Most people recover from the disease without needing special treatment. Around 1 out of every 6 people who get COVID-19 becomes seriously ill and has difficulty breathing.

278、**[How is COVID-19 transmitted?](https://www.who.int/emergencies/diseases/novel-coronavirus-2019/question-and-answers-hub/q-a-detail/coronavirus-disease-covid-19-risks-and-safety-for-older-people)**

People can catch COVID-19 from others who have the virus. The disease can spread from person to person through small droplets from the nose or mouth, which are spread when a person with COVID-19 coughs or exhales. These droplets land on objects and surfaces around the person. Other people then catch COVID-19 by touching these objects or surfaces, and then touching their eyes, nose or mouth. People can also catch COVID-19 if they breathe in droplets from a person with COVID-19 who coughs out or exhales droplets.

279、**[Who is at risk of developing severe disease?](https://www.who.int/emergencies/diseases/novel-coronavirus-2019/question-and-answers-hub/q-a-detail/coronavirus-disease-covid-19-risks-and-safety-for-older-people)**

Older people, and people of all ages with pre-existing medical conditions (such as diabetes, high blood pressure, heart disease, lung disease, or cancer) appear to develop serious illness more often than others.

280、**[Is there a vaccine, drug or treatment for COVID-19?](https://www.who.int/emergencies/diseases/novel-coronavirus-2019/question-and-answers-hub/q-a-detail/coronavirus-disease-covid-19-risks-and-safety-for-older-people)**

While some western, traditional or home remedies may provide comfort and alleviate symptoms of mild COVID-19, there are no medicines that have been shown to prevent or cure the disease. WHO does not recommend self-medication with any medicines, including antibiotics, as a prevention or cure for COVID-19. However, there are several ongoing clinical trials of both western and traditional medicines. WHO is coordinating efforts to develop vaccines and medicines to prevent and treat COVID-19 and will continue to provide updated information as soon as research results become available.

281、**[What can I do to prevent infection?](https://www.who.int/emergencies/diseases/novel-coronavirus-2019/question-and-answers-hub/q-a-detail/coronavirus-disease-covid-19-risks-and-safety-for-older-people)**

To prevent infection, there are a five things that you can do.

### 1. ****Wash your hands frequently and thoroughly with soap and water and dry them thoroughly.****

You can also use alcohol-based hand rub if your hands are not visibly dirty and if this product is available. Cleaning your hands frequently will remove the virus if it is on your hands. You can learn how to wash your hands in [this video](https://www.youtube.com/watch?v=3PmVJQUCm4E) (or [this visual](https://www.who.int/teams/integrated-health-services/infection-prevention-control)). If an alcohol-based hand rub or soap is not available, then using chlorinated water (0.05%) for handwashing is an option, but it is not ideal because frequent use may irritate your skin.

### 2. ****Cover your mouth and nose with a flexed elbow or tissue when coughing and sneezing****.

And remember to throw away the used tissue immediately in a bin with a lid and to wash your hands. This way you protect others from any virus released through coughs and sneezes.

### 3. ****Avoid touching your eyes, nose and mouth.****

Hands touch many surfaces which can be contaminated with the virus. If you touch your eyes, nose or mouth with unclean hands, you can transfer the virus from the surface to yourself.

### 4. ****Keep physical distance from others.****

If your national or local authorities have put in place confinement measures, it is important to respect them. Taking exercise outside is good for your physical and mental health, but should only be undertaken if regulations for your area allow it. When you do go out, avoid crowded spaces and maintain at least 1 metre distance (3 feet or arms-length) from others. Avoid unnecessary visits to your house. If visits are necessary (e.g. caregiver to support with activities of daily living), ask your visitor to regularly check for symptoms to ensure that they are symptom free when visiting you. Ask them to also follow these five key actions, including washing their hands when they first enter your home.

### ****5. Clean and disinfect frequently touched surfaces every day****.

This includes tables, doorknobs, light switches, countertops, handles, desks, phones, keyboards, toilets, taps, and sinks. If surfaces are dirty, use detergent or soap and water prior to disinfection. Learn more by clicking [here](https://www.cdc.gov/coronavirus/2019-ncov/prevent-getting-sick/disinfecting-your-home.html?CDC_AA_refVal=https%3A%2F%2Fwww.cdc.gov%2Fcoronavirus%2F2019-ncov%2Fprevent-getting-sick%2Fcleaning-disinfection.html).

282、**[What can older people do to prepare for COVID-19 in their community?](https://www.who.int/emergencies/diseases/novel-coronavirus-2019/question-and-answers-hub/q-a-detail/coronavirus-disease-covid-19-risks-and-safety-for-older-people)**

There is a lot that you can do to prepare for COVID-19 in your community.

- **Inform yourself of the special measures taken in your community** **as well as the services and the sources of reliable information**that are availableduring the health emergency (e.g. home deliveries, psychosocial support, health ministry website, alternative access to your pension).

- **Create a list of the basic supplies that you will need for at least two weeks and try to get these delivered where possible**(e.g.non-perishable food items, household products, batteries for assistive devices you may use, and prescription medicines). Alternatively, ask family members, caregivers, neighbours or community leaders to help with ordering and/or delivery of groceries or prescription medicines. Make sure that your mobile phone credit is topped up and identify a safe place to charge your phone regularly so that you can keep in contact with family and friends and reach emergency services if needed.

- **Make a list of emergency numbers**(e.g. COVID-19 local helpline, nearby hospital and health emergencies numbers, hotline for victims of abuse, psychosocial support hotline)**and support contacts** (e.g. family members and friends, main caregiver, community care worker, associations of older persons). If you live alone, you may wish to share this list and ask your neighbours, family or caregiver to be in touch regularly, for example, by phone or video chat.

- **Discuss with your health-care worker**how your health needs can be addressed during COVID-19. This may involve postponing non-urgent appointments, talking to your doctor or health-care worker by phone or video chat instead of in person and/or revising your vaccination schedule.

- **If you rely on the support provided by a caregiver**,**identify with him or her another person that you trust**to support your daily living and care needs in case your caregiver is unable to continue to provide care. Together, you can note down all the personal care and assistance that you require and how it should be provided and share it with this trusted person so that they can be ready to provide care in case of need.

- **If you are the primary caregiver of another person who is care dependent** (e.g. grandchild, older spouse, child with a disability), **identify a person** that you and the person that you care for trust **to take on your caregiving responsibilities in case you fall sick**. Local authorities or volunteer organizations that provide support in these situations in your community might be able to help.

- **If multiple people live in your home, if possible prepare a separate room or space in your home** so that anyone showing symptoms compatible with COVID-19 can be isolated from others. If you do not have space for self-isolation, contact your community leaders or local health authorities to see if there is community space that could help you or other household members self-isolate.

- **Think about what matters most to you regarding care and support, including medical treatment, in case something happens to you and you are unable to make your own decisions.** If you want to develop an advanced care plan to record your treatment and care wishes, you can talk about it with your health-care worker or someone that you trust. You can write down your wishes and  share them with people you trust.

283、**[How can I keep healthy on a daily basis during the COVID-19 pandemic?](https://www.who.int/emergencies/diseases/novel-coronavirus-2019/question-and-answers-hub/q-a-detail/coronavirus-disease-covid-19-risks-and-safety-for-older-people)**

You can follow these 10 steps to keep healthy during the COVID-19 pandemic.

1. **Keep to your regular routines as much as possible** **and maintain a daily schedule** for yourself including sleeping, meals and activities.

1. **Stay socially connected**. Speak to loved ones and people you trust every day or as much as possible, using the telephone, video-calls or messaging, through writing letters, etc. Use this time to share your feelings and to do common hobbies together.

1. **Be physically active every day**. Reduce long periods of sitting and set up a daily routine that includes at least 30 minutes of exercise. Make sure to do activities that are safe and appropriate for your level of physical fitness as indicated by your health-care worker. You can use household chores as a way to keep physically active, follow an on-line class (e.g. Tai Chi, yoga) or choose your favourite music and dance to that.

1. **Drink water and eat healthy and well-balanced meals**. This will keep you hydrated, help strengthen your immune system and lower the risk of chronic illnesses and infectious diseases. [For nutrition advice click here](http://www.emro.who.int/images/stories/nutrition/documents/en_flyer_nutrition_adults_covid_19.pdf?ua=1).

1. **Avoid smoking and drinking alcohol*.***Smokers are likely to be more vulnerable to COVID-19 because smoking can affect lung capacity and because the act of smoking increases the possibility of transmission of virus from hand to mouth. Drinking alcohol not only disturbs your sleep but may also increase your risk of falls, weaken your immune system, and interact with any prescription medicines that you are taking. Limit the amount of alcohol you drink or don’t drink alcohol at all.

1. **Take breaks from news coverage** about COVID-19 as prolonged exposure can cause feelings of anxiety and despair. Seek updates at specific times of the day from a reliable source like the [WHO website (click here)](https://www.who.int/emergencies/diseases/novel-coronavirus-2019) or national or local channels in order to help you distinguish facts from rumours or scams.

1. **Engage in hobbies and activities that you enjoy or learn something new**. Cognitive exercise such as reading a book or doing crosswords/Sudoku will keep your mind active and distract you from worrying. You can also use this time to keep a well-being diary *(*[*click here for an example.*](https://pscentre.org/wp-content/uploads/2020/03/14-Day-Well-Being-Kit_ALL_English_HKRC_202003.pdf)*)*

1. **If you have ongoing health conditions*,***take your prescribed medicines and follow the advice of your health-care worker regarding any health visits or phone consultations.

1. **If you have an emergency medical condition that is not related to COVID-19**, contact health emergencies immediately and ask what you should do next. Follow the instructions of the health-care worker.

1. **If stress, worry, fear or sadness get in the way of your daily activities for several days in a row, seek psychosocial support**from available services in your community.  If you are subject to abuse or violence from others, tell someone you trust and report this to the relevant authorities. You can also seek support from dedicated helplines that may be available in your country.

284、**[What should I do if I have symptoms of COVID-19?](https://www.who.int/emergencies/diseases/novel-coronavirus-2019/question-and-answers-hub/q-a-detail/coronavirus-disease-covid-19-risks-and-safety-for-older-people)**

- **If you present symptoms related to COVID-19, seek medical advice**.Call by phone first if possible and give information about pre-existing health conditions and medicines that you are taking. Follow the instructions of the health-care worker and monitor your symptoms regularly.

- **If you have difficulty breathing, contact health emergencies immediately** as this may be due to a respiratory infection. Call by phone first if possible to learn what to do next.

- **If you live with others, make sure that you isolate yourself**as soon as you suspect infectionby using the space that you identified in advance. You and other members of the household should also wear a medical mask as much as possible if these are available. *You can learn*[*how to wear a mask here*](https://www.youtube.com/watch?v=M4olt47pr_o)*.*

- **If you live with others and home care for COVID-19 is advised by your health-care worker**, other household members should follow available guidance on home care for patients with COVID-19 presenting with mild symptoms and management of their contacts *(guidance*[*here*](https://www.who.int/publications-detail-redirect/home-care-for-patients-with-suspected-novel-coronavirus-(ncov)-infection-presenting-with-mild-symptoms-and-management-of-contacts)*).* See related [flyers for people who are ill](https://www.who.int/images/default-source/health-topics/coronavirus/risk-communications/home-care-posters/home-care-ill-people-a4-covid.png?sfvrsn=71718c4b_2)*,* [for household members](https://www.who.int/images/default-source/health-topics/coronavirus/risk-communications/home-care-posters/home-care-everyone-a4-covid.png?sfvrsn=29ba54d8_2) and [for caregivers](https://cdn.who.int/media/images/default-source/health-topics/coronavirus/risk-communications/home-care-posters/home-care-caregivers-a4-covid.png?sfvrsn=8c412ecd_4)*.*

- **If you live alone** **and home care for COVID-10 is advised by your health-care worker***,*ask your family, friends, neighbours, health-care worker or a local volunteer organisation to check in on you regularly and to provide support as needed following [existing guidance for caregivers](https://www.who.int/publications-detail-redirect/home-care-for-patients-with-suspected-novel-coronavirus-(ncov)-infection-presenting-with-mild-symptoms-and-management-of-contacts)*(guidance*[*here*](https://www.who.int/publications-detail-redirect/home-care-for-patients-with-suspected-novel-coronavirus-(ncov)-infection-presenting-with-mild-symptoms-and-management-of-contacts)*).*

285、**[I have recently lost someone I care about. What advice do you have to help me cope?](https://www.who.int/emergencies/diseases/novel-coronavirus-2019/question-and-answers-hub/q-a-detail/coronavirus-disease-covid-19-risks-and-safety-for-older-people)**

Losing someone close to you is always hard, whatever the cause. During these extraordinary circumstances, when your usual routine may be disrupted and when funerals may not be permitted, it may be even harder. Following this advice may help.

- **Do not criticise yourself for how you feel**. When you lose a family member or friend to COVID-19, you may experience a range of emotions.  You may also have difficulty sleeping or low levels of energy. All these feelings are normal and there is no right or wrong way to feel grief.
- **Allow yourself time to process your emotions in response to your loss**. You may think that the sadness and pain that you feel will never go away, but in most cases, these feelings lessen over time.
- **Talk regularly with people that you trust about your feelings.**

- **Keep to your routines as much as you feel able and try to focus on activities that bring you joy.**

- **Seek advice and comfort from people that you trust**(e.g. religious/faith leaders, mental health workers or other trusted members of your community) while maintaining physical distance (e.g. attending virtual mass)**.**

- **Think of alternative ways to say goodbye to the person who passed away**such as writing a letter or dedicating a drawing to your loved one. These are small actions that can help you cope with grief and loss, particularly in situations where funeral services are not permitted

286、**[What precautions are necessary when visiting someone in a health and/or long-term care facility?](https://www.who.int/emergencies/diseases/novel-coronavirus-2019/question-and-answers-hub/q-a-detail/coronavirus-disease-covid-19-risks-and-safety-for-older-people)**

If you have been in contact with someone suspected or confirmed with COVID-19, or are feeling unwell, do not visit any health or long-term care facility.

Follow the facility guidelines on any visit requirements, including screening and wearing a mask.

Clean your hands before entering and try to keep at least a 1 metre distance from others.

If you are 60 or over, or have a chronic condition like heart disease, take extra precautions by wearing a medical mask during your visit.

#### [Coronavirus disease (COVID-19): Schools（287-300）](https://www.who.int/emergencies/diseases/novel-coronavirus-2019/question-and-answers-hub/q-a-detail/coronavirus-disease-covid-19-schools)

287、**[Are children at lower risk of COVID-19 than adults?](https://www.who.int/emergencies/diseases/novel-coronavirus-2019/question-and-answers-hub/q-a-detail/coronavirus-disease-covid-19-schools)**

So far, data suggests that children under the age of 18 years represent about 8.5% of reported cases, with relatively few deaths compared to other age groups and usually mild disease. However, cases of critical illness have been reported. As with adults, pre-existing medical conditions have been suggested as a risk factor for severe disease and intensive care admission in children.

Further studies are underway to assess the risk of infection in children and to better understand transmission in this age group.

288、**[What is the role of children in transmission?](https://www.who.int/emergencies/diseases/novel-coronavirus-2019/question-and-answers-hub/q-a-detail/coronavirus-disease-covid-19-schools)**

The role of children in transmission is not yet fully understood. To date, few outbreaks involving children or schools have been reported. However, the small number of outbreaks reported among teaching or associated staff to date suggests that spread of COVID-19 within educational settings may be limited.

As children generally have milder illness and fewer symptoms, cases may sometimes go unnoticed. Importantly, early data from studies suggest that infection rates among teenagers may be higher than in younger children.

Considering that many countries are starting to slowly lift restrictions on activities, the longer-term effects of keeping schools open on community transmission are yet to be evaluated. Some modelling studies suggest that school re-opening might have a small effect on wider transmission in the community, but this is not well understood. Further studies are underway on the role of children in transmission in and outside of educational settings. WHO is collaborating with scientists around the world to develop protocols that countries can use to study COVID-19 transmission in educational institutions. [Click here to access this information](https://www.who.int/emergencies/diseases/novel-coronavirus-2019/technical-guidance/early-investigations).

289、**[Should children with underlying health conditions (asthma, diabetes, obesity) return to school?](https://www.who.int/emergencies/diseases/novel-coronavirus-2019/question-and-answers-hub/q-a-detail/coronavirus-disease-covid-19-schools)**

Whether a child should go to school depends on their health condition, the current transmission of COVID-19 within their community, and the protective measures the school and community have in place to reduce the risk of COVID-19 transmission. While current evidence suggests that the risk of severe disease for children is lower overall than for adults, special precautions can be taken to minimize the risk of infection among children, and the benefits of returning to school should also be considered.

Current evidence suggests that people with underlying conditions such as chronic respiratory illness including asthma (moderate-to-severe), obesity, diabetes or cancer, are at higher risk of developing severe disease and death than people without other health conditions. This also appears to be the case for children, but more information is still needed.

290、**[What is the incubation period for children?](https://www.who.int/emergencies/diseases/novel-coronavirus-2019/question-and-answers-hub/q-a-detail/coronavirus-disease-covid-19-schools)**

The incubation period for children is the same as in adults. The time between exposure to COVID-19 and when symptoms start is commonly around 5 to 6 days, and ranges from 1 to 14 days.

291、**[What should be considered when deciding whether to re-open schools or keep them open?](https://www.who.int/emergencies/diseases/novel-coronavirus-2019/question-and-answers-hub/q-a-detail/coronavirus-disease-covid-19-schools)**

Deciding to close, partially close or reopen schools should be guided by a risk-based approach, to maximize the educational, well-being and health benefit for students, teachers, staff, and the wider community, and help prevent a new outbreak of COVID-19 in the community.

Several elements should be assessed in deciding to re-open schools or keep them open:

- The epidemiology of COVID-19 at the local level: This may vary from one place to another within a country
- Benefits and risks: what are the likely benefits and risks to children and staff of open schools? Including consideration of:
  - Transmission intensity in the area where the school operates: No cases, sporadic transmission; clusters transmission or community transmission
  - Overall impact of school closures on education, general health and wellbeing; and on vulnerable and marginalized populations (e.g. girls, displaced or disabled)
  - Effectiveness of remote learning strategies
- Detection and response: Are the local health authorities able to act quickly?
- The capacity of schools/educational institutions to operate safely
- Collaboration and coordination: Is the school collaborating with local public health authorities?
- The range of other public health measures being implemented outside school

292、**[What benefits would school re-opening provide?](https://www.who.int/emergencies/diseases/novel-coronavirus-2019/question-and-answers-hub/q-a-detail/coronavirus-disease-covid-19-schools)**

School closures have clear negative impacts on child health, education and development, family income and the overall economy.

The decision to reopen schools should include consideration of the following benefits:

- Allowing students to complete their studies and continue to the next level
- Essential services, access to nutrition, child welfare, such as preventing violence against children
- Social and psychological well-being
- Access to reliable information on how to keep themselves and others safe
- Reducing the risk of non-return to school
- Benefit to society, such as allowing parents to work

293、**[What are the prevention and control measures to be prepared and put in place in schools?](https://www.who.int/emergencies/diseases/novel-coronavirus-2019/question-and-answers-hub/q-a-detail/coronavirus-disease-covid-19-schools)**

There are several actions and requirements that should be reviewed and put in place to prevent the introduction and spread of COVID-19 in schools and into the community; and to ensure the safety of children and school staff while at school. Special provisions should be considered for early childhood development, higher learning institutions, residential schools or specialized institutions.

WHO recommends the following:

**Community-level measures:**Carry out early detection, testing, contact tracing and quarantine of contacts; investigate clusters; ensure physical distancing, hand and hygiene practices and age-appropriate mask use; shield vulnerable groups. Community-led initiatives such as addressing misleading rumors also play an important role in reducing the risk of infection.

**Policy, practice and infrastructure**: Ensure the necessary resources, policies and infrastructure, are in place that protect the health and safety of all school personnel, including people at higher risk.

**Behavioral aspects**: Consider the age and capacity of students to understand and respect measures put in place. Younger children may find it more difficult to adhere to physical distancing or the appropriate use of masks.

**Safety and security**: School closure or re-opening may affect the safety and security of students and the most vulnerable children may require special attention, such as during pick-up and drop-off.

**Hygiene and daily practices at the school and classroom level**: Physical distancing of at least 1 metre between individuals including spacing of desks, frequent hand and respiratory hygiene, age-appropriate mask use, ventilation and environmental cleaning measures should be in place to limit exposure. Schools should educate staff and students on COVID-19 prevention measures, develop a schedule for daily cleaning and disinfection of the school environment, facilities and frequently touches surfaces, and ensure availability of hand hygiene facilities and national/local guidance on the use of masks.

**Screening and care of sick students, teachers and other school staff**: Schools should enforce the policy of “staying home if unwell”, waive the requirement for a doctor’s note, create a checklist for parents/students/staff to decide whether to go to school (taking into consideration the local situation), ensure students who have been in contact with a COVID-19 case stay home for 14 days, and consider options for screening on arrival.

**Protection of individuals at high-risk:**Schools should identify students and teachers at high-risk with pre-existing medical conditions to come up with strategies to keep them safe; maintain physical distancing and se of medical masks as well as frequent hand hygiene and respiratory etiquette.

**Communication with parents and students**: Schools should keep students and parents informed about the measures being implemented to ensure their collaboration and support.

**Additional school-related measures such as immunization checks and catch-up vaccination programmes**: Ensure continuity or expansion of essential services, including school feeding and mental health and psycho-social support.

**Physical distancing outside classrooms**: Maintain a distance of at least 1 metre for both students (all age groups) and staff, where feasible.

**Physical distancing inside classrooms:**

In areas with **community transmission** of COVID-19, maintain a distance of at least 1 metre between all individuals of all age groups, for any schools remaining open. This includes increasing desk spacing and staging recesses, breaks and lunchbreaks; limiting the mixing of classes and of age groups; considering smaller classes or alternating attendance schedules, and ensuring good ventilation in classrooms.

In areas with **cluster-transmission** of COVID-19, a risk-based approach should be taken when deciding whether to keep a distance of at least 1 metre between students. Staff should always keep at least 1 metre apart from each other and from students and should wear a mask in situations where 1-metre distance is not practical.

In areas with **sporadic cases/no cases** of COVID-19, children under the age of 12 should not be required to keep physical distance at all times. Where feasible, children aged 12 and over should keep at least 1 metre apart from each other.  Staff should always keep at least 1 metre from each other and from students and should wear a mask in situations where 1-metre distance is not practical. **Remote learning**: Where children cannot attend classes in person, support should be given to ensure students have continued access to educational materials and technologies (internet, texting radio, radio, or television), (e.g. delivering assignments or broadcasting lessons). Shutting down educational facilities   should only be considered when no alternatives are available.

294、**[Checklist for parents/caregivers and community members](https://www.who.int/emergencies/diseases/novel-coronavirus-2019/question-and-answers-hub/q-a-detail/coronavirus-disease-covid-19-schools)**

- Monitor your child’s health and keep them home from school if they are ill.
- Teach and model good hygiene practices for your children:
  - Wash your hands with soap and safe water frequently. If soap and water are not readily available, use an alcohol-based hand sanitizer with at least 60% alcohol. Always wash hands with soap and water, if hands are visibly dirty.
  - Ensure that safe drinking water is available and toilets or latrines are clean and available at home.
  - Ensure waste is safely collected, stored and disposed of.
  - Cough and sneeze into a tissue or your elbow and avoid touching your face, eyes, mouth and nose.
- Encourage your children to ask questions and express their feelings with you and their teachers. Remember that your child may have different reactions to stress; be patient and understanding.
- Prevent stigma by using facts and reminding students to be considerate of one another.
- Coordinate with the school to receive information and ask how you can support school safety efforts (though parent-teacher committees, etc),.

295、**[Checklist for students and children](https://www.who.int/emergencies/diseases/novel-coronavirus-2019/question-and-answers-hub/q-a-detail/coronavirus-disease-covid-19-schools)**

- In a situation like this it is normal to feel sad, worried, confused, scared or angry. Know that you are not alone and talk to someone you trust, like your parent or teacher so that you can help keep yourself and your school safe and healthy.
  - Ask questions, educate yourself and get information from reliable sources.
- Protect yourself and others:
  - Wash your hands frequently, always with soap and water for at least 20 seconds.
  - Remember to not touch your face, eyes, nose and mouth.
  - Do not share cups, eating utensils, food or drinks with others.
- Be a leader in keeping yourself, your school, family and community healthy.
  - Share what you learn about preventing disease with your family and friends, especially with younger children
  - Model good practices such as sneezing or coughing into your elbow and washing your hands, especially for younger family members.
- Don’t stigmatize your peers or tease anyone about being sick; remember that the virus doesn’t follow geographical boundaries, ethnicities, age or ability or gender.
- Tell your parents, another family member, or a caregiver if you feel sick, and ask to stay home.

296、**[What are the risks during transportation to and from schools?](https://www.who.int/emergencies/diseases/novel-coronavirus-2019/question-and-answers-hub/q-a-detail/coronavirus-disease-covid-19-schools)**

The following adaptations to transport to and from school should be implemented to limit unnecessary exposure of school or staff members.

- Promote and put in place respiratory and hand hygiene, physical distancing measures and use of masks in transportation such as school buses, in accordance with local policy.
- Provide tips for how to safely commute to and from school, including for public transportation.
- Organize only one child per seat and ensure physical distancing of at least 1 metre between passengers in school buses, if possible. This may require more school buses per school.
- If possible and safe, keep the windows of the buses, vans, and other vehicles open.

297、**[Does WHO recommend staff and children to wear masks at school? And if yes, what type of masks?](https://www.who.int/emergencies/diseases/novel-coronavirus-2019/question-and-answers-hub/q-a-detail/coronavirus-disease-covid-19-schools)**

In countries or areas where there is intense community transmission of COVID-19 and in settings where physical distancing cannot be achieved, the following criteria for use of masks in schools are recommended:

1. Children aged 5 years and under should not be required to wear masks.

2. For children between six and 11 years of age, a risk-based approach should be applied to the decision to use a mask, considering:

- intensity of transmission in the area where the child is and evidence on the risk of infection and transmission in this age group.
- beliefs, customs and behaviours.
- the child’s capacity to comply with the correct use of masks and availability of adult supervision.
- potential impact of mask wearing on learning and development.
- additional considerations such as sport activities or for children with disabilities or underlying diseases.

3. Children and adolescents 12 years or older should follow the national mask guidelines for adults.

4. Teacher and support staff may be required to wear masks when they cannot guarantee at least a 1-metre distance from others or there is widespread transmission in the area.

**Types of mask:**

Fabric masks are recommended to prevent onward transmission in the general population in public areas, particularly where distancing is not possible, and in areas of community transmission. This could include the school grounds in some situations. Masks may help to protect others, because wearers may be infected before symptoms of illness appear. The policy on wearing a mask or face covering should be in line with national or local guidelines. Where used, [masks](https://www.who.int/emergencies/diseases/novel-coronavirus-2019/question-and-answers-hub/q-a-detail/q-a-on-covid-19-and-masks) should be worn, cared for and disposed of properly.

**The use of masks by children and adolescents in schools should only be considered as one part of a strategy to limit the spread of COVID-19.**

298、**[Are there any specific recommendations on school ventilation and air conditioning use?](https://www.who.int/emergencies/diseases/novel-coronavirus-2019/question-and-answers-hub/q-a-detail/coronavirus-disease-covid-19-schools)**

Yes, ensure adequate ventilation and increase total airflow supply to occupied spaces, if possible. Clean, natural ventilation (i.e., opening windows) should be used inside buildings where possible, without re-circulating the air. If heating, ventilation and air conditioning systems are used they should be regularly inspected, maintained and cleaned. Rigorous standards for installation, maintenance and filtration are essential to make sure they are effective and safe. Consider running the systems at maximum outside airflow for two hours before and after times when the building is occupied, according to the manufacturer’s recommendations.

299、**[What should be monitored after re-opening of school?](https://www.who.int/emergencies/diseases/novel-coronavirus-2019/question-and-answers-hub/q-a-detail/coronavirus-disease-covid-19-schools)**

The following should be monitored:

- effectiveness of symptoms-reporting, monitoring, rapid testing and tracing of suspected cases
- the effects of policies and measures on educational objectives and learning outcomes
- the effects of policies and measures on health and well-being of children, siblings, staff, parents and other family members
- the trend in school dropouts after lifting the restrictions
- the number of cases in children and staff in the school, and frequency of school-based outbreaks in the local administrative area and the country.
- Assessment of impact of remote teaching on learning outcomes.

Based on what is learned from this monitoring, further modifications should be made to continue to provide children and staff with the safest environment possible.

300、补原290[**Should teachers and other staff with underlying health conditions return to school?**](https://www.who.int/emergencies/diseases/novel-coronavirus-2019/question-and-answers-hub/q-a-detail/coronavirus-disease-covid-19-schools)

Adults 60 years and older and people with underlying health conditions are at higher risk for severe disease and death. The decision to return to a teaching environment depends on the individual and should include consideration of local disease trends, as well as the measures being put in place in schools to prevent further spread.

#### [Coronavirus disease (COVID-19): Serology, antibodies and immunity（301-307）](https://www.who.int/emergencies/diseases/novel-coronavirus-2019/question-and-answers-hub/q-a-detail/coronavirus-disease-covid-19-serology)

301、**[What is serology?](https://www.who.int/emergencies/diseases/novel-coronavirus-2019/question-and-answers-hub/q-a-detail/coronavirus-disease-covid-19-serology)**

‘Serology’ is the study of antibodies in blood serum.

‘Antibodies’ are part of the body’s immune response to infection. Antibodies that work against SARS-CoV-2 – the virus that causes COVID-19 – are usually detectable in the first few weeks after infection. The presence of antibodies indicates that a person was infected with SARS-CoV-2, irrespective of whether the individual had severe or mild disease, or no symptoms.

‘Seroprevalence studies’ are conducted to measure the extent of infection, as measured by antibody levels, in a population under study. With any new virus, including SARS-CoV-2, initial seroprevalence in the population is assumed to be low or non-existent due to the fact that the virus has not circulated before.

302、**[What is the difference between molecular testing and serologic testing?](https://www.who.int/emergencies/diseases/novel-coronavirus-2019/question-and-answers-hub/q-a-detail/coronavirus-disease-covid-19-serology)**

‘Molecular testing’, including polymerase-chain reaction (PCR) testing, detects genetic material of the virus and so can detect if a person is currently infected with SARS-CoV-2.

‘Serologic testing’ detects antibodies against a virus, measuring the amount of antibodies produced following infection, thereby detecting if a person has previously been infected by SARS-CoV-2. Serologic tests should not be used to diagnose acute SARS-CoV-2 infection, as antibodies develop a few weeks after infection.

303、**[What is the purpose of serologic testing?](https://www.who.int/emergencies/diseases/novel-coronavirus-2019/question-and-answers-hub/q-a-detail/coronavirus-disease-covid-19-serology)**

When a new disease, like COVID-19 emerges, initial surveillance and testing strategies focus initially on patients with severe disease and the use of molecular testing to measure acute infections, as these are the individuals who seek and require health care. This can often miss the fraction of mild or asymptomatic infections that do not require medical attention, and as such, the full extent of infection is not known early in an outbreak.

Serologic testing helps retrospectively determine the size of an outbreak or extent of infection in a population under study. Seroprevalence studies give a more complete picture of how much of a population has been infected with SARS-CoV-2 and will capture unrecognized cases not identified through routine or active surveillance.

304、**[Does the presence of antibodies mean that a person is immune?](https://www.who.int/emergencies/diseases/novel-coronavirus-2019/question-and-answers-hub/q-a-detail/coronavirus-disease-covid-19-serology)**

There are many studies underway to better understand the antibody response following infection to SARS-CoV-2.  Several studies to date show that most people who have been infected with SARS-CoV-2 develop antibodies specific to this virus. However, the levels of these antibodies can vary between those who have severe disease (higher levels of antibodies) and those with milder disease or asymptomatic infection (lower levels of antibodies). Many studies are underway to better understand the levels of antibodies that are needed for protection, and how long these antibodies last.

305、**[Can people who have had SARS-CoV-2 infection be re-infected?](https://www.who.int/emergencies/diseases/novel-coronavirus-2019/question-and-answers-hub/q-a-detail/coronavirus-disease-covid-19-serology)**

To date, there are some reports of individuals who have been reinfected with SARS-CoV-2.  There are likely to be more examples of reinfection reported and scientists are working to understand the role of the immune response in the first and second infection. WHO is working with scientists to understand each occurrence of reinfection and the antibody response during the first and subsequent infections.

306、**[What are the results of seroprevalence studies published to date?](https://www.who.int/emergencies/diseases/novel-coronavirus-2019/question-and-answers-hub/q-a-detail/coronavirus-disease-covid-19-serology)**

There are now more than 200 peer-reviewed publications,  pre-prints,  manuscripts and government reports of SARS-CoV-2 seroprevalence studies. These studies vary in study design, populations under study, serologic tests used, timing of sample collection, and quality. Overall, the population-based seroprevalence reported across available studies remains low, at below 10%.

Some studies conducted in areas of known high virus transmission and studies of health care workers in areas of known high transmission have reported seroprevalence estimates over 20%.

Available study results indicate that, globally, most people remain susceptible to SARS-CoV-2 infection.

307、**[What is herd immunity?](https://www.who.int/emergencies/diseases/novel-coronavirus-2019/question-and-answers-hub/q-a-detail/coronavirus-disease-covid-19-serology)**

'Herd immunity', also known as 'population immunity', is the indirect protection from an infectious disease that happens when a population is immune either through vaccination or immunity developed through previous infection. WHO supports achieving 'herd immunity' through vaccination, not by allowing a disease to spread through any segment of the population, as this would result in unnecessary cases and deaths.

Herd immunity against COVID-19 should be achieved by protecting people through vaccination, not by exposing them to the pathogen that causes the disease. *Read the Director-General’s*[*12 October media briefing speech*](https://www.who.int/director-general/speeches/detail/who-director-general-s-opening-remarks-at-the-media-briefing-on-covid-19---12-october-2020)*for more detail.*

Vaccines train our immune systems to create proteins that fight disease, known as ‘antibodies’, just as would happen when we are exposed to a disease but – crucially – vaccines work without making us sick. Vaccinated people are protected from getting the disease in question and passing on the pathogen, breaking any chains of transmission. *Visit our*[*webpage*](https://www.who.int/emergencies/diseases/novel-coronavirus-2019/covid-19-vaccines)*on COVID-19 and vaccines for more detail.*

To safely achieve herd immunity against COVID-19, a substantial proportion of a population would need to be vaccinated, lowering the overall amount of virus able to spread in the whole population. One of the aims with working towards herd immunity is to keep vulnerable groups who cannot get vaccinated (e.g. due to health conditions like allergic reactions to the vaccine) safe and protected from the disease. *Read our*[*Q&A*](https://www.who.int/news-room/questions-and-answers/item/vaccines-and-immunization-what-is-vaccination)*on vaccines and immunization for more information.*

The percentage of people who need to be immune in order to achieve herd immunity varies with each disease. For example, herd immunity against measles requires about 95% of a population to be vaccinated. The remaining 5% will be protected by the fact that measles will not spread among those who are vaccinated. For polio, the threshold is about 80%. The proportion of the population that must be vaccinated against COVID-19 to begin inducing herd immunity is not known. This is an important area of research and will likely vary according to the community, the vaccine, the populations prioritized for vaccination, and other factors.

Achieving herd immunity with safe and effective vaccines makes diseases rarer and saves lives.

*Find out more about the science behind herd immunity by watching or reading this* [*interview*](https://www.who.int/emergencies/diseases/novel-coronavirus-2019/media-resources/science-in-5/episode-1) *with WHO’s Chief Scientist, Dr Soumya Swaminathan.*

#### [Coronavirus disease (COVID-19): Similarities and differences between COVID-19 and Influenza（308-312）](https://www.who.int/emergencies/diseases/novel-coronavirus-2019/question-and-answers-hub/q-a-detail/coronavirus-disease-covid-19-similarities-and-differences-with-influenza)

308、**[How are COVID-19 and influenza viruses similar?](https://www.who.int/emergencies/diseases/novel-coronavirus-2019/question-and-answers-hub/q-a-detail/coronavirus-disease-covid-19-similarities-and-differences-with-influenza)**

**1. COVID-19 and influenza are both respiratory diseases.**

Both viruses share similar symptoms, including cough, runny nose, sore throat, fever, headache and fatigue. People may have varying levels of illness with both COVID-19 and influenza. Some may have no symptoms, mild symptoms or severe disease. Both influenza and COVID-19 can be fatal.

**2. COVID-19 and influenza spread in similar ways**.

Both COVID-19 and influenza are spread by droplets and aerosols when an infected person coughs, sneezes, speaks, sings or breathes. The droplets and aerosols can land in the eyes, nose or mouth of people who are nearby -- typically within 1 metre of the infected person, but sometimes even further away. People can also get infected with both COVID-19 and influenza by touching contaminated surfaces, then touching their eyes, nose or mouth without cleaning their hands.

**3. Some of the same groups have a higher risk for severe illness from COVID-19 and influenza.**

While all age groups can be infected with both the COVID-19 virus and influenza virus, these people are at higher risk for severe disease and death from both COVID-19 and influenza:

- older adults;
- people of any age with chronic medical conditions (such as chronic cardiac, pulmonary, renal, metabolic, neurologic, liver or hematologic diseases); and
- people with immunosuppressive conditions (such as HIV/AIDS, patients receiving chemotherapy or steroids, or malignancy)

Healthcare workers are at high risk of getting infected by COVID-19 and at high risk of getting infected by influenza.

Pregnant people and those who have recently given birth are at high risk for experiencing severe influenza and severe COVID-19.

**4. The same protective measures are effective against COVID-19 and influenza.**

To protect against COVID-19 and influenza, follow these public health and social measures:

- Maintain at least a 1-metre distance from others and wear a well-fitted mask when that’s not possible;
- Avoid crowded and poorly ventilated places and settings;
- Open windows or doors to keep rooms well ventilated;
- Cough or sneeze into a bent elbow or tissue and throw the tissue into a closed bin;
- Clean your hands frequently
- Avoid touching your eyes, nose and mouth;
- Stay home if you don’t feel well; and
- Contact your medical provider if you have any of the following severe [severe](https://www.who.int/images/default-source/health-topics/coronavirus/covid19-infographic-symptoms-final.tmb-1920v.png) symptoms of COVID-19, including:
  - Shortness of breath,
  - Loss of appetite,
  - Confusion,
  - Persistent pain or pressure in the chest,
  - High temperature (above 38 °C)

Vaccination is an important part of preventing severe disease and death for both COVID-19 and influenza. Follow the advice of your local authorities on getting the influenza and COVID-19 vaccines. WHO recommends the influenza vaccine for older individuals, young children, pregnant people, people with underlying health conditions, and health workers.

COVID-19 vaccines are safe for most people 18 years and older, including those with pre-existing conditions of any kind, including auto-immune disorders. These conditions include: hypertension, diabetes, asthma, pulmonary, liver and kidney disease, as well as chronic infections that are stable and controlled.

If supplies are limited in your area, discuss your situation with your care provider if you:

- Have a compromised immune system
- Are pregnant or nursing your baby
- Have a history of severe allergies, particularly to a vaccine (or any of the ingredients in the vaccine)
- Are severely frail

People with mild symptoms of both COVID-19 and influenza can usually be cared for safely at home. For more information about caring for someone safely at home with COVID-19, watch [this episode of Science in 5](https://www.who.int/emergencies/diseases/novel-coronavirus-2019/media-resources/science-in-5/episode-36---safe-care-at-home?gclid=CjwKCAjw-sqKBhBjEiwAVaQ9a00jbT7lq1cSe5d-MCdtvr5LOtVQ2D5H3E_iCv86z99-JX9sH9QI3hoCw2wQAvD_BwE).

**5. There are safe and effective vaccines for both COVID-19 and influenza.**

COVID-19 vaccines have been proven to protect against severe illness and death caused by COVID-19 disease. Millions of people around the world have safely received COVID-19 vaccines, and the vaccines have met rigorous standards for safety, effectiveness and quality. Getting vaccinated may also protect the people around you. If you are protected from getting COVID-19, you are less likely to infect someone else. COVID-19 vaccines do not protect against influenza.

WHO recommends vaccinations each year to prevent severe influenza disease in high-risk groups: pregnant people, individuals with underlying health conditions, older adults, health workers, and young children.

309、**[How are COVID-19 and influenza viruses different?](https://www.who.int/emergencies/diseases/novel-coronavirus-2019/question-and-answers-hub/q-a-detail/coronavirus-disease-covid-19-similarities-and-differences-with-influenza)**

**1. Treatments for COVID-19 and influenza are different.**

The treatment options in use for COVID-19 at medical facilities include oxygen, corticosteroids, and IL6 receptor blockers for severely ill patients. Treatment for people with severe respiratory illness includes advanced respiratory support such as the use of ventilators. Several other treatment options for COVID-19 are currently in clinical trials.

Antiviral drugs for influenza can reduce severe complications and death, and they are especially important for high-risk groups. It’s important to remember that antibiotics are not effective against influenza or COVID-19 viruses.

People with mild symptoms of both diseases can usually be treated safely at home. Click [here](https://www.who.int/images/default-source/health-topics/coronavirus/risk-communications/home-care-posters/home-care-ill-people-a4-covid.png) for more information about caring for someone with COVID-19 safely at home.

**2. Vaccines for COVID-19 and flu are different.**

Vaccines developed for COVID-19 do not protect against influenza, and similarly, the flu vaccine does not protect against COVID-19. Follow the advice of your local authorities on getting the influenza and COVID-19 vaccines.

310、**[If I have had my flu shot, do I also need the COVID-19 vaccine?](https://www.who.int/emergencies/diseases/novel-coronavirus-2019/question-and-answers-hub/q-a-detail/coronavirus-disease-covid-19-similarities-and-differences-with-influenza)**

Yes. You need both vaccinations.

311、**[Can someone be infected with COVID-19 and influenza at the same time?](https://www.who.int/emergencies/diseases/novel-coronavirus-2019/question-and-answers-hub/q-a-detail/coronavirus-disease-covid-19-similarities-and-differences-with-influenza)**

Yes, it is possible to catch both diseases at the same time. The most effective way to prevent hospitalization and severe COVID-19 and influenza is vaccination with both vaccines.

Continue to follow prevention measures, such as maintaining at least a 1-metre distance from others, wearing a well-fitted mask when keeping your distance is not possible, avoiding crowded and poorly ventilated places and settings, opening windows and doors to keep rooms well ventilated and cleaning your hands frequently.

312、**[How can I protect myself against COVID-19 and influenza?](https://www.who.int/emergencies/diseases/novel-coronavirus-2019/question-and-answers-hub/q-a-detail/coronavirus-disease-covid-19-similarities-and-differences-with-influenza)**

The most effective way to protect yourself from both influenza and severe COVID is to get vaccinated with both influenza and COVID vaccines.

The most effective way to prevent infection with the virus that causes COVID-19 is to get vaccinated and follow prevention measures: maintaining at least a 1-metre distance from others, wearing a well-fitted mask when keeping your distance is not possible, avoiding crowded and poorly ventilated places and settings, opening windows and doors to keep rooms well ventilated and cleaning your hands frequently.

Follow the advice of your local authorities on getting the influenza and COVID-19 vaccines.

#### [Coronavirus disease (COVID-19): Small public gatherings（313-314）](https://www.who.int/emergencies/diseases/novel-coronavirus-2019/question-and-answers-hub/q-a-detail/coronavirus-disease-covid-19-small-public-gatherings)

313、**[I am planning to attend a wedding and a party. What precautions should I take to protect myself and others from getting infected with COVID-19?](https://www.who.int/emergencies/diseases/novel-coronavirus-2019/question-and-answers-hub/q-a-detail/coronavirus-disease-covid-19-small-public-gatherings)**

In the context of the COVID-19 pandemic, there is no “zero risk” when it comes to any kind of gathering – especially events that bring groups of people together. Regardless of the size of the event, you are at risk from COVID-19 whenever you get together with people. The virus that causes COVID-19 spreads easily indoors, especially in poorly ventilated settings.

If you don’t feel well or show any [symptoms of COVID-19](https://www.who.int/images/default-source/health-topics/coronavirus/covid19-infographic-symptoms-final.tmb-1920v.png), stay home.

If you choose to attend a gathering, practice prevention measures, regardless of your COVID-19 vaccination status:

- keep at least a 1 metre distance from others;
- wear a mask;
- avoid crowded or poorly ventilated areas;
- cover coughs and sneezes with bent elbow or tissues; and
- clean your hands frequently.

These measures are a good idea to protect yourself and others, even when the event or venue doesn’t require you to practice them.

314、**[I am organizing a gathering for family and friends. What steps can I take to make the event as safe as possible for my guests?](https://www.who.int/emergencies/diseases/novel-coronavirus-2019/question-and-answers-hub/q-a-detail/coronavirus-disease-covid-19-small-public-gatherings)**

If you are unable to postpone your event, consider hosting it in a well-ventilated outdoor space. The virus that causes COVID-19 spreads easily indoors, especially in poorly ventilated settings. Outdoor venues are safer than indoor spaces.

Make your event as safe as possible for guests. Keep it small and short. Encourage people to wear masks and ensure enough space for each guest to maintain at least a 1-metre distance from others. Help your guests follow COVID-19 prevention measures: provide masks, alcohol-based hand sanitizer or access to soap and water, tissues and bins with lids that close.

As always, follow guidance issued by local public health authorities before you plan a gathering.

#### [Coronavirus disease (COVID-19): Solidarity Trial and hydroxychloroquine（315-318）](https://www.who.int/emergencies/diseases/novel-coronavirus-2019/question-and-answers-hub/q-a-detail/coronavirus-disease-covid-19-hydroxychloroquine)

315、**[Why was the use of hydroxychloroquine stopped in the Solidarity Trial?](https://www.who.int/emergencies/diseases/novel-coronavirus-2019/question-and-answers-hub/q-a-detail/coronavirus-disease-covid-19-hydroxychloroquine)**

On 17 June 2020, WHO announced that the hydroxychloroquine (HCQ) arm of the Solidarity Trial to find an effective COVID-19 treatment was being stopped.

The trial's Executive Group and principal investigators made the decision based on evidence from the Solidarity trial, UK's Recovery trial and a Cochrane review of other evidence on hydroxychloroquine.

Data from Solidarity (including the French Discovery trial data) and the recently announced results from the UK's Recovery trial both showed that hydroxychloroquine does not result in the reduction of mortality of hospitalised COVID-19 patients, when compared with standard of care.

316、**[What will happen to the people who were already enrolled in the hydroxychloroquine arm of the trial?](https://www.who.int/emergencies/diseases/novel-coronavirus-2019/question-and-answers-hub/q-a-detail/coronavirus-disease-covid-19-hydroxychloroquine)**

Investigators will not randomize further patients to hydroxychloroquine in the Solidarity trial. Patients who have already started hydroxychloroquine but who have not yet finished their course in the trial may complete their course or stop at the discretion of the supervising physician.

The use of hydroxychloroquine and chloroquine are accepted as generally safe for use in patients with autoimmune diseases or malaria.

317、**[What is WHO’s view of the use of hydroxychloroquine in pre or post-exposure prophylaxis?](https://www.who.int/emergencies/diseases/novel-coronavirus-2019/question-and-answers-hub/q-a-detail/coronavirus-disease-covid-19-hydroxychloroquine)**

The decision to stop hydroxychloroquine’s use in the Solidarity trial does not apply to the use or evaluation of hydroxychloroquine in pre or post-exposure prophylaxis in patients exposed to COVID-19

318、**[What is the Solidarity Trial?](https://www.who.int/emergencies/diseases/novel-coronavirus-2019/question-and-answers-hub/q-a-detail/coronavirus-disease-covid-19-hydroxychloroquine)**

The Solidarity Trial is an international clinical trial to help find an effective treatment for COVID-19, launched by WHO and partners. It is hoped that one or more of the treatments under trial will result in improving clinical outcomes in COVID-19 patients and save lives. Other trials are on-going around the world in addition to the Solidarity Trial.

The treatment options are: Remdesivir; Lopinavir/Ritonavir; and Lopinavir/Ritonavir with Interferon beta-1a. The treatment options were originally selected based on evidence from laboratory, animal and clinical studies. Hydroxychloroquine was originally included in the trial but this arm was stopped, as of 17 June 2020, as evidence showed it did not result in the reduction of mortality of hospitalised COVID-19 patients, when compared with standard of care.

Over 100 countries have expressed an interest in participating in the trial and WHO is actively supporting more than 60 of them, including with the following:

- ethical and regulatory approvals of the WHO core protocol;
- identification of hospitals participating in the trial;
- training of hospital clinicians on the web-based randomization and data system;
- shipping the trial drugs as requested by each participating country.

As of 3 June 2020, more than 3500 patients have been recruited in 35 countries, with over 400 hospitals actively recruiting patients.

Interim trial analyses are monitored by a Global Data and Safety Monitoring Committee, which is an independent group of experts.

#### [Coronavirus disease (COVID-19): Staying active（319-323）](https://www.who.int/emergencies/diseases/novel-coronavirus-2019/question-and-answers-hub/q-a-detail/coronavirus-disease-covid-19-staying-active)

319、**[What is physical activity?](https://www.who.int/emergencies/diseases/novel-coronavirus-2019/question-and-answers-hub/q-a-detail/coronavirus-disease-covid-19-staying-active)**

Physical activity includes all forms of active recreation, sports participation, cycling and walking, as well as activities you do at work and around the home and garden. It doesn’t have to be exercise or sport – play, dance, gardening, and even house cleaning and carrying heavy shopping is all part of being physically active.

During the COVID-19 pandemic, when so many of us are very restricted in our movements, it is even more important for people of all ages and abilities to be as active as possible. Even a short break from sitting, by doing 3-5 minutes of physical movement, such as walking or stretching, will help ease muscle strain, relieve mental tension and improve blood circulation and muscle activity. Regular physical activity can also help to give the day a routine and be a way of staying in contact with family and friends.

320、**[Why do we need it?](https://www.who.int/emergencies/diseases/novel-coronavirus-2019/question-and-answers-hub/q-a-detail/coronavirus-disease-covid-19-staying-active)**

Regular physical activity benefits both the body and mind. It can reduce high blood pressure, help manage weight and reduce the risk of heart disease, stroke, type 2 diabetes, and various cancers. It also improves bone and muscle strength and increases balance, flexibility and fitness. For older people, activities that improve balance help to prevent falls and injuries. For children, regular physical activity helps support healthy growth and development and reduce the risk of disease in later life, and through regular activity, children can develop fundamental movement skills and build social relationships.

Regular physical activity also improves mental health and can reduce the risk of depression, cognitive decline and delay the onset of dementia - and improve overall feelings of wellbeing.

321、H[**ow much physical activity is recommended?**](https://www.who.int/emergencies/diseases/novel-coronavirus-2019/question-and-answers-hub/q-a-detail/coronavirus-disease-covid-19-staying-active)

WHO has detailed recommendations on the amount of physical activity people of all ages should do to benefit their health and wellbeing. (available [here](https://apps.who.int/iris/handle/10665/311664) for children under age of 5 and [here](https://apps.who.int/iris/bitstream/handle/10665/44399/9789241599979_eng.pdf;jsessionid=1CA0AB344ADA8498780C49A85807429B?sequence=1) for youth, adults and olders) Here are the minimum levels we recommend:

**Infants under the age of 1 year need to** 

- be physically active several times a day.

**Children under 5 years of age**

- should spend at least 180 minutes a day in physical activities, with 3-4 year-olds being moderately or vigorously active for an hour a day.

**Children and adolescents aged 5-17years**

- all children and adolescents should do at least 60 minutes a day of moderate to vigorous-intensity physical activity, including activities that strengthen muscle and bone, at least 3 days per week.

**Adults aged 18 years and over**

- should do a total of at least 150 minutes of moderate-intensity physical activity throughout the week, or at least 75 minutes of vigorous-intensity physical activity throughout the week, including muscle-strengthening activities 2 or more days per week.
- older adults with poor mobility should do physical activity to enhance balance and prevent falls on 3 or more days per week.

But any physical activity is better than none.  Start with small amounts and gradually increase duration, frequency and intensity over time.

Being active during the COVID-19 pandemic is challenging for us all.  Because the opportunities to be physically active seem to be more restricted, it is even more important to plan in every day the ways to be active and to reduce the time spent sitting for long periods. Put simply, it is a critical time to ensure we all move more and sit less.

322、**[So how do I stay safe while exercising in COVID-19?](https://www.who.int/emergencies/diseases/novel-coronavirus-2019/question-and-answers-hub/q-a-detail/coronavirus-disease-covid-19-staying-active)**

**Do not exercise if you have a fever, cough and difficulty breathing.** Stay home and rest, seek medical attention and call in advance. Follow the directions of your local health authority.

**If you are able to go for a walk or bicycle ride** always practice physical distancing and wash your hands with water and soap before you leave, when you get to where you are going, and as soon as you get home.  If water and soap are not immediately available, use alcohol-based hand rub.

**If you go to a park or public open space to walk, run or exercise** always practice physical distancing and wash your hands with water and soap, before you leave, when you get to where you are going, and as soon as you get home.  If water and soap are not immediately available, use alcohol-based hand rub. Follow the directions of your local health authority in regards to any restrictions on the number of people with you and/or restrictions on the use of public outdoor play or exercise equipment.

**If you are not regularly active start slowly and with low intensity activities**, like walking and low impact exercises. Start with shorter amounts, like 5-10 minutes, and gradually build up to 30 minutes or more continuously over a few weeks. It is better and safer to be active for short periods more frequently than to try and be active for long periods when you are not used to it.

**Choose the right activity so that you reduce the risk of injury** and that you enjoy the activity. Choose the right intensity according to your health status and fitness level. You should be able to breath comfortably and hold a conversation while you do light- and moderate-intensity physical activity.

323、**[How do I stay active in and around the home?](https://www.who.int/emergencies/diseases/novel-coronavirus-2019/question-and-answers-hub/q-a-detail/coronavirus-disease-covid-19-staying-active)**

**Try and reduce long periods of time spent sitting**, whether for work, studying, watching TV, reading, or using social media or playing games using screens. Reduce sitting for long periods by taking short 3-5 minute breaks every 20-30 minutes. Simply stand up and stretch or even better, take a walk around the house, up and down the stairs, or into the garden. By just moving around and stretching you can improve your health and wellbeing. For more ideas and illustrations of healthy stretches see [here.](https://www.euro.who.int/en/health-topics/health-emergencies/coronavirus-covid-19/publications-and-technical-guidance/noncommunicable-diseases/stay-physically-active-during-self-quarantine)

**Set up a regular routine to be active every day**, by planning a physical activity or exercise break either by yourself, by joining an online class, or by setting up a time to be active online with your friends or colleagues. Making a specific time to be active helps ensure you get your daily physical activity. Put the time in your diary, and it will help remind you. Stick with it, as this will help you build a regular routine, and help you adjust to new ways of working, study and family life under COVID-19 restrictions

**Be active with your family and friends,** connecting with others can help you and your family in the home and elsewhere spend time together and be active. Planning time to be active with your children with active games at home, walks in the parks, or cycling can be a way the whole family can relax, be together and be active and healthy whilst at home.

**Set yourself and your family Be Active goals,** by choosing a specific type of activity, time of day and/or number of minutes you will do every day. Get each family member to choose their own goal which sets a bit of a challenge but is realistic with help from family or friends and motivation. Record your progress on a weekly activity chart and, if you think it would help, reward yourself with something you value.

[Stay physically active during self-quarantine](https://www.euro.who.int/en/health-topics/health-emergencies/coronavirus-covid-19/publications-and-technical-guidance/noncommunicable-diseases/stay-physically-active-during-self-quarantine)

**More physical activity ideas to help you stay active**

**For Infants under 1 year of age**

- Spend regular time doing floor-based play with your baby in a prone position (‘tummy time’) and spread this throughout the day while baby is awake.

**For Children under 5 years of age**

- Active play in and around the home – invent games which involve being active and can develop skills in throwing, catching, kicking, as well as developing posture and balance.
- Active play and games where children get out of breath, such as running around, skipping and jumping.

**For Children and adolescents aged 5-17 years**

- Active games and active play with family.
- Join in online active games or activity classes, also look for online physical education classes as well as exercise routines suitable for adolescents.
- Set up playground games indoors such as Jump rope and hop-scotch – make up new games and challenges that involve being active.
- Learn a new skill – for example try an learn to juggle.
- Encourage doing some muscle strength training activities such as lifting weights or use improvised weight such as bottles full of water or sand.

**For Adults**

- Climb up the stairs as much as you can, think of it as an opportunity to be active.
- Use household chores as a way to be more physical activity.
- Join in an online exercise class or make up your own routine to music you enjoy that uses the major muscle groups and raises you heart rate.
- Do some muscle strengthening activities such as lifting weights or improvise using full bottles of water or simply use your own body weight and do sets of press ups, sit ups and squats.
- Make time for fun, such as dancing to music.

**Where to get more help and information**

For more ideas use internet search platforms and check out social media for suggestions and access to free resources suitable for children of all ages and adults of all fitness and abilities.

#### [Coronavirus disease (COVID-19): Staying at hotels and accommodation establishments(324-329)](https://www.who.int/emergencies/diseases/novel-coronavirus-2019/question-and-answers-hub/q-a-detail/coronavirus-disease-covid-19-staying-at-hotels-and-accommodation-establishments)

324、**[What precautions should everyone take in a hotel or other accommodation establishment?](https://www.who.int/emergencies/diseases/novel-coronavirus-2019/question-and-answers-hub/q-a-detail/coronavirus-disease-covid-19-staying-at-hotels-and-accommodation-establishments)**

Everyone should follow basic precautions:

- Wash all parts of your hands frequently (at least 20 seconds if using an alcohol-based hand rub, and at least 40 seconds with soap and water), including after exchanging objects such as money or credit cards. Look for hand sanitizer stations and use them before going into dining halls, restaurants or bars.
- Cover a cough or sneeze with a bent elbow or tissue, and throw away the tissue in a closed bin.
- Maintain at least a 1 metre distance from staff and other guests. This includes avoiding hugging, kissing, or shaking hands. If you can’t guarantee the distance, wear a mask. Be sure to check local and national guidelines on the use of masks.

325、**[Are pools and water areas safe to use?](https://www.who.int/emergencies/diseases/novel-coronavirus-2019/question-and-answers-hub/q-a-detail/coronavirus-disease-covid-19-staying-at-hotels-and-accommodation-establishments)**

Gym, beach, swimming pool, spa, sauna and steam bath facilities can be used safely with certain restrictions, as determined by national guidelines.  In particular, look for the following measures:

- a maximum number of people allowed to ensure adequate physical distancing
- fabric mask policies required by local or national regulations
- hand washing stations, especially in the toilet and change room areas
- single use towels only
- a bin for guests to place their towel after use for laundering
- individual use drinking water
- tissues and waste containers with lids
- high touch areas such as door handles disinfected regularly throughout the day

326、**[Are there precautions to take while eating in a restaurant?](https://www.who.int/emergencies/diseases/novel-coronavirus-2019/question-and-answers-hub/q-a-detail/coronavirus-disease-covid-19-staying-at-hotels-and-accommodation-establishments)**

There is no evidence that the virus that causes COVID-19 is transmitted by food, including fresh fruits and vegetables. The virus can be killed while cooking food at temperatures of at least 70°C.

Food buffets are not recommended because of the risk of close physical contact with others, shared serving implements and multiple people touching the surfaces on the buffet. Indoor dining spaces should have a maximum of 4 people in 10 square metres. The distance from the back of one chair to the back of another chair should be at least 1 metre apart for both indoor and outdoor dining, and guests that face each other should also be at this distance.

Guests should be reminded when entering and leaving the area to clean their hands. When the physical distance of at least 1 metre cannot be guaranteed, masks are recommended to be worn by staff and guests.

More on masks, including who should wear what kind and when, is available [here](https://www.who.int/emergencies/diseases/novel-coronavirus-2019/advice-for-public/when-and-how-to-use-masks).

327、 **[Is it safe to use fans or air conditioning in an accommodation?](https://www.who.int/emergencies/diseases/novel-coronavirus-2019/question-and-answers-hub/q-a-detail/coronavirus-disease-covid-19-staying-at-hotels-and-accommodation-establishments)**

Ventilation is an important factor in preventing the virus that causes COVID-19 from spreading. Recirculated air from split air conditioning units, fan coils or any system that runs with a recirculation mode should be avoided where possible, unless in a single occupancy room with no one else present. If recirculation is unavoidable, increase outdoor air exchange by opening windows, if possible and safe to do so, and minimize air blowing from one person directly at another.

Floor or ceiling fans can provide ventilation when the people occupying the room are from the same household, but are not recommended when travelers from different households are together.

328、**[Can you get the virus from people who were in the room previous to you?](https://www.who.int/emergencies/diseases/novel-coronavirus-2019/question-and-answers-hub/q-a-detail/coronavirus-disease-covid-19-staying-at-hotels-and-accommodation-establishments)**

Hotels and other accommodations should have procedures for cleaning, disinfecting and ventilating the room properly between every guest’s stay. These processes allow the accommodations to be used immediately afterward. If these procedures are followed, there is no need to leave the room empty between guests.

329、**[What should travellers do if they get sick while at an accommodation facility?](https://www.who.int/emergencies/diseases/novel-coronavirus-2019/question-and-answers-hub/q-a-detail/coronavirus-disease-covid-19-staying-at-hotels-and-accommodation-establishments)**

If a guest develops symptoms of COVID-19, such as fever, dry cough or tiredness, they should notify the facility manager and seek medical advice by contacting local health authorities.

The guest should isolate from others, including fellow travellers. If the guest cannot be isolated or staff need to enter the room, the ill person should put on a medical mask, and people nearby should also wear a mask.  If the medical mask cannot be tolerated by the ill person, then he or she should cough or sneeze into a bent elbow or use tissues to cover the mouth, and discard the tissue immediately into a closed waste bag.

#### [Coronavirus disease (COVID-19): Tobacco（330-335）](https://www.who.int/emergencies/diseases/novel-coronavirus-2019/question-and-answers-hub/q-a-detail/coronavirus-disease-covid-19-tobacco)

330、**[As a smoker, am I likely to get more severe symptoms if I get COVID-19?](https://www.who.int/emergencies/diseases/novel-coronavirus-2019/question-and-answers-hub/q-a-detail/coronavirus-disease-covid-19-tobacco)**

Tobacco compromises lung function, and COVID-19 primarily affects the lungs. Smoking tobacco is also a known risk factor for severe disease from many respiratory infections, including coronaviruses SARS (first identified in 2003) and MERS-CoV (first documented in June 2012). Smoking also impairs the immune system and previous studies have established that tobacco use is linked with poorer outcomes for people with TB and pneumonia.

Evidence indicates that smokers are more likely to suffer more severe outcomes of COVID-19, such as admission into intensive care units and death, than never smokers.  Furthermore, severe forms of COVID-19 or deaths due to COVID-19 are more frequent in people with comorbidities that are related to tobacco use, including COPD, lung cancer and cardiovascular diseases. There is currently only limited information on COVID-19 in relation to other tobacco products (e.g. heated tobacco products, waterpipe, cigars) and electronic nicotine delivery systems (e.g. e-cigarettes), although these products are thought to play an unfavourable role in COVID-19 severity.

331、**[As a e-cigarette user, am I more likely to have more severe symptoms if infected?](https://www.who.int/emergencies/diseases/novel-coronavirus-2019/question-and-answers-hub/q-a-detail/coronavirus-disease-covid-19-tobacco)**

There is currently limited evidence about the relationship between e-cigarette use and COVID-19. However, existing evidence indicates that electronic nicotine delivery systems (ENDS) and electronic non-nicotine delivery systems (ENNDS), more commonly referred to as e-cigarettes, are harmful and increase the risk of heart disease and lung disorders. Given that the COVID-19 virus affects the respiratory tract, e-cigarette use may increase the risk of infection or severity of outcomes due to COVID-19.

332、**[What about using smokeless tobacco, like chewing tobacco?](https://www.who.int/emergencies/diseases/novel-coronavirus-2019/question-and-answers-hub/q-a-detail/coronavirus-disease-covid-19-tobacco)**

Using smokeless tobacco often involves some hand to mouth contact. Another risk associated with using smokeless tobacco products, like chewing tobacco, is that the virus can be spread when the user spits out the excess saliva produced during the chewing process.

333、**[What does WHO recommend for tobacco users?](https://www.who.int/emergencies/diseases/novel-coronavirus-2019/question-and-answers-hub/q-a-detail/coronavirus-disease-covid-19-tobacco)**

Given the risks to health that tobacco use causes, WHO recommends quitting tobacco use. Quitting will help your lungs and heart to work better from the moment you stop. Within 20 minutes of quitting, elevated heart rate and blood pressure drop. After 12 hours, the carbon monoxide level in the bloodstream drops to normal. Within 2–12 weeks, circulation improves and lung function increases. After 1–9 months, coughing and shortness of breath decrease. Quitting will help to protect your loved ones, especially children, from exposure to second-hand smoke.

WHO recommends the use of proven interventions such as toll-free quit lines, mobile text-messaging cessation programmes, and nicotine replacement therapies (NRTs), among others, for quitting tobacco use.

334、**[What can I do to protect people from the risks associated with smoking, smokeless tobacco use and vaping?](https://www.who.int/emergencies/diseases/novel-coronavirus-2019/question-and-answers-hub/q-a-detail/coronavirus-disease-covid-19-tobacco)**

- If you smoke, use e-cigarettes or use smokeless tobacco, now is a good time to quit completely.
- Do not share devices like waterpipes and e-cigarettes.
- Spread the word about the risks of smoking, using e-cigarettes and using smokeless tobacco.
- Protect others from the harms of second-hand smoke.
- Know the importance of washing your hands, physical distancing, and not sharing any smoking or e-cigarette products.
- Do not spit in public places.

335、**[Can nicotine affect my chances in the context of COVID-19?](https://www.who.int/emergencies/diseases/novel-coronavirus-2019/question-and-answers-hub/q-a-detail/coronavirus-disease-covid-19-tobacco)**

There is currently insufficient information to confirm any link between nicotine in the prevention or treatment of COVID-19. WHO urges researchers, scientists and the media to be cautious about amplifying unproven claims that tobacco or nicotine could reduce the risk of COVID-19. WHO is constantly evaluating new research, including that which examines the link between tobacco use, nicotine use, and COVID-19.

#### [Coronavirus disease (COVID-19): Tocilizumab and sarilumab - Interleukin-6 receptor blockers（336-342）](https://www.who.int/emergencies/diseases/novel-coronavirus-2019/question-and-answers-hub/q-a-detail/coronavirus-disease-(covid-19)-tocilizumab-and-sarilumab---interleukin-6-receptor-blockers)

336、**[I have COVID-19, should I be prescribed IL-6 receptor blockers?](https://www.who.int/emergencies/diseases/novel-coronavirus-2019/question-and-answers-hub/q-a-detail/coronavirus-disease-(covid-19)-tocilizumab-and-sarilumab---interleukin-6-receptor-blockers)**

Tocilizumab and sarilumab are interleukin-6 (IL-6) receptor blockers that WHO recommends for patients diagnosed with severe or critical COVID-19.* They should be administered by a healthcare worker in a monitored clinical setting along with the current standard of care for COVID-19, which includes oxygen, corticosteroids, and other medications.

Patients with non-severe COVID-19 should not be given this treatment.

**A patient has severe COVID-19 when they have signs of pneumonia, severe respiratory distress, and their blood oxygen level is low. A patient has critical COVID-19 when the need life sustaining treatment, have acute respiratory distress syndrome, or have septic shock (evidence of injury to other organs).* [*More.*](https://www.bmj.com/content/370/bmj.m3379)

337、**[How are IL-6 receptor blockers administered?](https://www.who.int/emergencies/diseases/novel-coronavirus-2019/question-and-answers-hub/q-a-detail/coronavirus-disease-(covid-19)-tocilizumab-and-sarilumab---interleukin-6-receptor-blockers)**

For optimal effect, tocilizumab and sarilumab should be administered as soon as possible once a patient is diagnosed with severe or critical COVID-19.

Tocilizumab and sarilumab should be administered intravenously as a single dose by a healthcare worker in a monitored clinical setting over one hour. In rare circumstances a second dose may be administered intravenously at the clinical discretion of the healthcare provider 12 to 48 hours after the first dose.

Patients should have routine bloodwork prior to the administration of IL-6 receptor blockers and 3 days after completing the infusion (see the [guidelines](https://www.who.int/news/item/06-07-2021-who-recommends-life-saving-interleukin-6-receptor-blockers-for-covid-19-and-urges-producers-to-join-efforts-to-rapidly-increase-access) for more details).

338、**[Are IL-6 receptor blockers suitable for anyone with COVID-19?](https://www.who.int/emergencies/diseases/novel-coronavirus-2019/question-and-answers-hub/q-a-detail/coronavirus-disease-(covid-19)-tocilizumab-and-sarilumab---interleukin-6-receptor-blockers)**

**Patients with severe or critical COVID-19 should receive tocilizumab or sarilumab.**

Women of childbearing age should use contraception during and up to three months after receiving tocilizumab or sarilumab. This should be discussed with the patient and their healthcare provider.

339、**[How do IL-6 receptor blockers act?](https://www.who.int/emergencies/diseases/novel-coronavirus-2019/question-and-answers-hub/q-a-detail/coronavirus-disease-(covid-19)-tocilizumab-and-sarilumab---interleukin-6-receptor-blockers)**

Patients with severe or critical COVID-19 often develop overstimulation of their immune system which can be very harmful to their health. IL-6 receptor blockers act to suppress this overstimulation when given in addition to corticosteroids

340、**[Do IL-6 receptor blockers have side effects?](https://www.who.int/emergencies/diseases/novel-coronavirus-2019/question-and-answers-hub/q-a-detail/coronavirus-disease-(covid-19)-tocilizumab-and-sarilumab---interleukin-6-receptor-blockers)**

Known side effects of IL-6 receptor blockers, tocilizumab and sarilumab, include potential increase in cholesterol, decrease in neutrophils (type of white blood cells), increased risk of infection (bacterial, fungal, viral, and tuberculosis), liver abnormalities, discomfort at the infusion site, and infusion reaction. In rare cases patients may develop severe allergic reactions, significant liver dysfunction, neutropenia (low white blood count) or thrombocytopenia (low platelet count).

341、**[Do patients receiving IL-6 receptor blockers need monitoring?](https://www.who.int/emergencies/diseases/novel-coronavirus-2019/question-and-answers-hub/q-a-detail/coronavirus-disease-(covid-19)-tocilizumab-and-sarilumab---interleukin-6-receptor-blockers)**

All patients receiving tocilizumab or sarilumab should be clinically monitored by a healthcare provider before, during, and after receiving an infusion. The patient should also receive optimal standard of care for COVID-19, which includes oxygen therapy.

Certain patients who received IL-6 receptor blockers may be at a higher risk for complications and need to be monitored closely. These include patients receiving concomitant immunosuppressive therapy, those with recurring chronic infections or underlying conditions that predispose them to infection, and who are on long-term IL-6 receptor blockers for chronic medical conditions.

342、**[How expensive are IL-6 receptor blockers? Are they widely available?](https://www.who.int/emergencies/diseases/novel-coronavirus-2019/question-and-answers-hub/q-a-detail/coronavirus-disease-(covid-19)-tocilizumab-and-sarilumab---interleukin-6-receptor-blockers)**

Currently, tocilizumab and sarilumab are expensive and in short supply globally.

WHO and partners are discussing lower prices and improved access in low- and middle-income countries with the producers, Roche Pharmaceutical for tocilizumab and Sanofi S.A. for sarilumab.

WHO has launched a call to manufacturers to submit biosimilar versions of this drug for approval or [prequalification.](https://extranet.who.int/pqweb/sites/default/files/documents/EOI-COVID-19_v4.pdf) This would allow global production to be ramped up so that more people can access the drugs.

#### [Coronavirus disease (COVID-19): Travel advice for the general public（343-357）](https://www.who.int/emergencies/diseases/novel-coronavirus-2019/question-and-answers-hub/q-a-detail/coronavirus-disease-covid-19-travel-advice-for-the-general-public)

343、**[Should I travel?](https://www.who.int/emergencies/diseases/novel-coronavirus-2019/question-and-answers-hub/q-a-detail/coronavirus-disease-covid-19-travel-advice-for-the-general-public)**

If you are in a high-risk group for severe COVID-19 but have not been fully vaccinated and do not have immunity from a recent previous COVID-19 infection, consider postponing travel to areas where COVID-19 is widespread.

People in high-risk groups – including those over the age of 60, those with chronic illnesses, and those with underlying health conditions – face increased risk of severe illness and death from COVID-19. Pay close attention to the COVID-19 situation at your destination, and follow border entry requirements, which may include testing or quarantine.

In areas with high COVID-19 transmission rates, you may be at increased risk of COVID-19 infection. It’s important to know your health status and the risks of COVID-19 at your destination as you plan.

The rules for travel depend on where your trip originates and your destination. Follow travel advisories issued by both the country of origin and of destination.

344、**[What precautions should I take during travel?](https://www.who.int/emergencies/diseases/novel-coronavirus-2019/question-and-answers-hub/q-a-detail/coronavirus-disease-covid-19-travel-advice-for-the-general-public)**

Wherever you are, follow COVID-19 prevention measures, such as wearing a mask, washing hands frequently, maintaining physical distance of at least 1 metre, and avoiding crowded or poorly ventilated places and settings whenever possible.

Traveling may place you in areas that are closed, confined, or crowded (the three Cs), which puts you at a higher risk of being exposed to the virus that causes COVID-19. Whenever possible, stay in open, well-ventilated settings. While travelling, practice prevention measures even if you are fully vaccinated against COVID-19.

Be sure to follow COVID-19 travel policies and rules required by venues, transport operators and travel hubs such as airports, bus terminals, and train stations. Know before you go.

345、**[Who should not travel?](https://www.who.int/emergencies/diseases/novel-coronavirus-2019/question-and-answers-hub/q-a-detail/coronavirus-disease-covid-19-travel-advice-for-the-general-public)**

People with symptoms of COVID-19 – including fever, cough, or shortness of breath – should not travel.  If you are showing symptoms of COVID-19, stay home and isolate yourself away from other people. If you have been around someone who has been diagnosed with COVID-19, isolate yourself, follow the guidance of local authorities and watch for symptoms.

People in certain groups – including those over the age of 60, those with chronic illnesses, and those with underlying health conditions – face increased risk of severe illness and death from COVID-19. If you are in one of these groups but have not been fully vaccinated and do not have immunity from a recent previous COVID-19 infection, consider postponing travel to areas with widespread COVID-19

346、**[What is meant by “essential travel”?](https://www.who.int/emergencies/diseases/novel-coronavirus-2019/question-and-answers-hub/q-a-detail/coronavirus-disease-covid-19-travel-advice-for-the-general-public)**

As part of public health efforts to stop the spread of COVID-19, travel may be limited or restricted to travel deemed to be essential. Essential travel is defined differently by national authorities in different countries.

Essential travel may include travel for emergencies and humanitarian activities (including emergency medical flights and medical evacuation). It may include travel taken by essential personnel (including emergency responders and those who provide public health technical support, and critical personnel in transport and security sectors, such as seafarers and diplomatic officers). Essential travel may also include repatriations to home countries, as well as cargo transport needed to deliver essential supplies such as food, medicines, and fuel. WHO encourages national authorities to facilitate travel for essential reasons at all times.

347、**[If I feel well but am in the high-risk category, should I travel?](https://www.who.int/emergencies/diseases/novel-coronavirus-2019/question-and-answers-hub/q-a-detail/coronavirus-disease-covid-19-travel-advice-for-the-general-public)**

People in certain groups – including those over the age of 60, those with chronic illnesses, and those with underlying health conditions – face increased risk of severe illness and death from COVID-19. If you are in a high-risk group, you should consider the risk carefully before you make travel plans.

Be sure to follow COVID-19 prevention measures, such as wearing a mask, washing hands frequently, maintaining physical distance of at least 1 metre, and avoiding crowded places and poorly ventilated settings whenever possible. If you are in a high-risk group and must travel, wear a medical-grade face mask at all times and step up your prevention practices.

People aged 60 and over, and those with serious chronic diseases or underlying health conditions, should postpone or delay travel internationally to and from areas with community transmission unless they have been fully vaccinated at least two weeks prior to their travel, or have immunity from a recent infection within 6 months prior to travelling.  People in high-risk groups are at a greater risk of getting very sick or dying after getting COVID-19.

348、**[The travel company is asking me to wear a mask while traveling. Which kind of mask should I use?](https://www.who.int/emergencies/diseases/novel-coronavirus-2019/question-and-answers-hub/q-a-detail/coronavirus-disease-covid-19-travel-advice-for-the-general-public)**

Medical masks are recommended for:

- Anyone who is feeling unwell, including people with mild symptoms such as fever, cough, sore throat or headache.
- People aged 60 and over
- People of any age with underlying health conditions, including chronic respiratory disease, cardiovascular disease, cancer, obesity, immunocompromised patients and diabetes mellitus.

If you choose to use a fabric mask, WHO recommends the use of a well-fitting mask with three layers of fabric:

- Inner layer of absorbent material, such as cotton.
- Middle layer of non-woven non-absorbent material, such as polypropylene.
- Outer layer of non-absorbent material, such as polyester or polyester blend.

Remember that wearing a mask is only one part of important prevention measures, including washing hands frequently, maintaining physical distance of at least 1 meter, and avoiding crowded places and poorly ventilated settings whenever possible.

Learn more about how to choose and wear a mask [here](https://www.who.int/emergencies/diseases/novel-coronavirus-2019/advice-for-public/when-and-how-to-use-masks).

349、**[Should I get a vaccine or immunity certificate before travelling?](https://www.who.int/emergencies/diseases/novel-coronavirus-2019/question-and-answers-hub/q-a-detail/coronavirus-disease-covid-19-travel-advice-for-the-general-public)**

Before you travel, know the requirements and policies in place where your trip originates and at your destination. Every country has its own rules.

Some countries exempt travelers who have been fully vaccinated or have proof of recent SARS-CoV-2 infection from certain requirements, such as testing and/or quarantine.

During travel, even if you are vaccinated, WHO recommends that you continue wearing a mask, washing hands frequently, maintaining physical distance of at least 1 meter, and avoiding crowded places and poorly ventilated settings whenever possible.

350、**[Could I be screened when I exit or arrive at my destination?](https://www.who.int/emergencies/diseases/novel-coronavirus-2019/question-and-answers-hub/q-a-detail/coronavirus-disease-covid-19-travel-advice-for-the-general-public)**

Yes, you may be screened when entering or leaving a venue, destination or country. Be sure to know what is required or expected as you plan your travel.

Exit and entry screening may include measures such as interviews, checking temperatures and evaluation for symptom – including respiratory symptoms, fever, cough, or shortness of breath. You may be asked to provide information about any possible exposure you have had to people with COVID-19 symptoms or confirmed diagnoses.

351、**[Should I use a digital contact tracing app while traveling?](https://www.who.int/emergencies/diseases/novel-coronavirus-2019/question-and-answers-hub/q-a-detail/coronavirus-disease-covid-19-travel-advice-for-the-general-public)**

Some venues, destinations and countries may request or require that you download and register with a digital contact tracing app. These apps collect information to identify and inform travelers who may have been in contact with a person who has been confirmed to have COVID-19.

352、**[Should I get a COVID-19 test before departure or upon arrival?](https://www.who.int/emergencies/diseases/novel-coronavirus-2019/question-and-answers-hub/q-a-detail/coronavirus-disease-covid-19-travel-advice-for-the-general-public)**

Know before you go. Some venues, destinations and countries require COVID-19 tests prior to departure or upon arrival as part of their strategies to prevent the spread of disease. COVID-19 tests conducted immediately prior to departure or on arrival are used in an effort to determine the health status of travellers. As always, continue wearing a mask, washing hands frequently, maintaining physical distance of at least 1 metre, and avoiding crowded places and poorly ventilated settings whenever possible.

Every destination has its own policies, so it’s important to be aware of testing requirements before you hit the road.

353、**[What happens if I have symptoms while traveling or when I arrive at my destination?](https://www.who.int/emergencies/diseases/novel-coronavirus-2019/question-and-answers-hub/q-a-detail/coronavirus-disease-covid-19-travel-advice-for-the-general-public)**

If you have any symptoms of COVID-19 while you travel or when you arrive at your destination, notify health authorities immediately and follow their guidance. You may be required to quarantine or isolate yourself. Your travel companions may also be required to quarantine, since they have been in contact with you.

354、**[If I am asked to take a COVID-19 test at my destination, how much will it cost?](https://www.who.int/emergencies/diseases/novel-coronavirus-2019/question-and-answers-hub/q-a-detail/coronavirus-disease-covid-19-travel-advice-for-the-general-public)**

In most places, you should not be charged for physical exams, testing or vaccination. The [International Health Regulations (IHR)](https://www.who.int/health-topics/international-health-regulations) are an instrument of international law that is legally binding in 196 countries, including the 194 WHO Member States. The IHR includes important safeguards to protect the rights of travelers in the application of health measures.

In accordance with the IHR (2005), you should not be charged by the country for:

- examinations to determine your health status;
- required vaccinations or protective equipment on arrival (if these were not published at least 10 days earlier);
- appropriate isolation or quarantine facilities;
- required certificates at the point of entry that show or document the measures taken to allow your entry; and
- any baggage accompanying you.

355、**[Are there any special precautions I should take after I arrive at my destination?](https://www.who.int/emergencies/diseases/novel-coronavirus-2019/question-and-answers-hub/q-a-detail/coronavirus-disease-covid-19-travel-advice-for-the-general-public)**

As you plan your trip, make sure you are aware of the requirements and policies in place at your point of departure and at your destination. Follow them.

Be sure to practice COVID-19 prevention measures – wear a mask, maintain physical distance of at least one metre from others, wash your hands frequently, and avoid crowded or poorly ventilated places and settings.

Monitor yourself for [COVID-19 symptoms](https://www.who.int/news-room/questions-and-answers/item/coronavirus-disease-covid-19)  for 14 days after you travel, and notify public health agencies at your destination immediately if you develop symptoms such as fever, cough, sore throat or headache. Seek immediate medical attention if you have serious symptoms such as difficulty breathing or shortness of breath, chest pain or pressure, or loss of speech or movement.

356、**[How can ventilation reduce the risk of contracting COVID-19 in airplanes?](https://www.who.int/emergencies/diseases/novel-coronavirus-2019/question-and-answers-hub/q-a-detail/coronavirus-disease-covid-19-travel-advice-for-the-general-public)**

Some airplanes have cabin air filtration systems equipped with HEPA filters which can remove viruses and germs quickly, minimizing the duration of the exposure to any potential infectious materials produced by a cough or sneeze. The cabin air system is designed to operate most efficiently by delivering approximately 50 percent outside air and 50 percent filtered, recirculated air. The air supply is essentially sterile and particle-free.  However, adequate ventilation is just one of the preventive measures to reduce the risk of COVID-19 transmission.  Other important measures include maintaining physical distance of at least 1 meter whenever possible, frequent hand hygiene and wearing a mask. Passengers should check with the airline company and the national or local guidelines about when and where to wear a mask while flying.

357、**[What should I do if I get sick while traveling?](https://www.who.int/emergencies/diseases/novel-coronavirus-2019/question-and-answers-hub/q-a-detail/coronavirus-disease-covid-19-travel-advice-for-the-general-public)**

If you become ill during your travel, inform your travel attendant (plane, ship, train, etc.). You may be moved to a seat farther away from others.

Ask for information on how to be seen by a health care provider and seek care immediately.

Wear a mask continuously while you travel, frequently clean your hands with hand sanitizer, cover a cough or sneeze with a bent elbow or tissue, and maintain at least a 1 metre distance from others wherever possible. You should stop travelling as soon as feasible.

If you are told you must quarantine or self-isolate yourself in a specific place, you should be provided with free, appropriate facilities and care, and not be asked to stay longer than 14 days.

#### [Coronavirus disease (COVID-19): Tuberculosis（358-369）](https://www.who.int/emergencies/diseases/novel-coronavirus-2019/question-and-answers-hub/q-a-detail/coronavirus-disease-covid-19-tuberculosis)

358、**[Are people with tuberculosis likely to be at increased risk of COVID-19 infection, illness and death?](https://www.who.int/emergencies/diseases/novel-coronavirus-2019/question-and-answers-hub/q-a-detail/coronavirus-disease-covid-19-tuberculosis)**

While experience on COVID-19 infection in tuberculosis (TB) patients remains limited, it is anticipated that people ill with both TB and COVID-19 may have poorer treatment outcomes, especially if TB treatment is interrupted.

Older age, diabetes and chronic obstructive pulmonary disease (COPD) are linked with more severe COVID-19 and are also risk factors for poor outcomes in TB.

TB patients should take precautions as advised by health authorities to be protected from COVID-19 and continue their TB treatment as prescribed.

People ill with COVID-19 and TB show similar symptoms such as cough, fever and difficulty breathing. Both diseases attack primarily the lungs and although both biological agents transmit mainly via close contact, the incubation period from exposure to disease in TB is longer, often with a slow onset.

359、**[Do COVID-19 and tuberculosis spread in the same way?](https://www.who.int/emergencies/diseases/novel-coronavirus-2019/question-and-answers-hub/q-a-detail/coronavirus-disease-covid-19-tuberculosis)**

While both tuberculosis (TB) and COVID-19 spread by close contact between people the exact mode of transmission differs, explaining some differences in infection control measures to mitigate the two conditions.

TB bacilli remain suspended in the air in droplet nuclei for several hours after a TB patient coughs, sneezes, shouts, or sings, and people who inhale them can get infected. The size of these droplet nuclei is a key factor determining their infectiousness. Their concentration decreases with ventilation and exposure to direct sunlight.

COVID-19 transmission has primarily been attributed to the direct breathing of droplets expelled by someone with COVID-19 (people may be infectious before clinical features become apparent). Droplets produced by coughing, sneezing, exhaling and speaking may land on objects and surfaces, and contacts can get infected with COVID-19 by touching them and then touching their eyes, nose or mouth . Handwashing, in addition to respiratory precautions, are thus important in the control of COVID-19.

360、**[What is the potential impact of COVID-19 pandemic on essential tuberculosis services?](https://www.who.int/emergencies/diseases/novel-coronavirus-2019/question-and-answers-hub/q-a-detail/coronavirus-disease-covid-19-tuberculosis)**

Modelling work suggests that if the COVID-19 pandemic led to a global reduction of 25% in expected TB detection for 3 months – a realistic possibility given the levels of disruption in TB services being observed in multiple countries – then we could expect a 13% increase in TB deaths, bringing us back to the levels of TB mortality that we had 5 years ago. This may even be a conservative estimate as it does not factor in other possible impacts of the pandemic on TB transmission, treatment interruptions and poorer outcomes in people with TB and COVID-19 infection [(Predicted impact of the COVID-19 pandemic on global tuberculosis deaths in 2020, P. Glaziou)](https://www.medrxiv.org/content/10.1101/2020.04.28.20079582v1). Between 2020 and 2025 an additional 1.4 million TB deaths could be registered as a direct consequence of the COVID-19 pandemic ([Stop TB Partnership analysis](http://www.stoptb.org/assets/documents/news/Modeling%20Report_1%20May%202020_FINAL.pdf)).

All measures should be taken to ensure continuity of services for people who need preventive and curative treatment for tuberculosis (TB).

361、**[What should health authorities do to provide sustainability of essential tuberculosis services during the COVID-19 pandemic?](https://www.who.int/emergencies/diseases/novel-coronavirus-2019/question-and-answers-hub/q-a-detail/coronavirus-disease-covid-19-tuberculosis)**

People-centred delivery of tuberculosis (TB) prevention, diagnosis, treatment and care services should be ensured in tandem with the COVID-19 response.

**Prevention:** Measures must be implemented to limit transmission of TB and COVID-19 in congregate settings and health care facilities. Administrative, environmental and personal protection measures apply to both (e.g. basic infection prevention and control, cough etiquette, patient triage).

Provision of TB preventive treatment should be maintained as much as possible.

**Diagnosis**: Tests for TB and COVID-9 are different and both should be made available for individuals with respiratory symptoms, which may be similar for the two diseases.

**Treatment and care**: People-centred outpatient and community-based care should be strongly preferred over hospital treatment for TB patients (unless serious conditions require hospitalization) to reduce opportunities for transmission.

Anti-TB treatment, in line with the latest WHO guidelines, must be provided for all TB patients, including those in quarantine and those with confirmed COVID-19 disease. Adequate stocks of TB medicines should be provided to all patients to reduce trips to collect medicines.

Use of digital health technologies for patients and programmes should be intensified. In line with WHO recommendations, technologies like electronic medication monitors and video-supported therapy can help patients complete their TB treatment.

362、**[How can health authorities ensure supplies of tuberculosis medicines and diagnostics?](https://www.who.int/emergencies/diseases/novel-coronavirus-2019/question-and-answers-hub/q-a-detail/coronavirus-disease-covid-19-tuberculosis)**

Appropriate planning and monitoring are essential to ensure that procurement and supply of tuberculosis (TB) medicines and diagnostics are not interrupted.

WHO is monitoring medicine supply at the global level, while The Global Fund, the Stop TB Partnership Global Drug Facility (GDF), USAID, Unitaid and other donors play an essential role in supporting countries to secure adequate and sustainable supplies of TB medicines, drugs and diagnostics.

Countries are advised to place their orders for 2020 delivery as soon as possible given anticipated delays in transport and delivery mechanisms.

363、**[What services can be leveraged across both diseases?](https://www.who.int/emergencies/diseases/novel-coronavirus-2019/question-and-answers-hub/q-a-detail/coronavirus-disease-covid-19-tuberculosis)**

The response to COVID-19 can benefit from the capacity building efforts developed for tuberculosis (TB) over many years of investment by national authorities and donors. These include infection prevention and control, contact tracing, household and community-based care, and surveillance and monitoring systems.

Although modes of transmission of the two diseases are slightly different, administrative, environmental and personal protection measures apply to both (e.g. basic infection prevention and control, cough etiquette, patient triage).

TB laboratory networks have been established in countries with the support of WHO and international partners. These networks as well as specimen transportation mechanisms could also be used for COVID-19 diagnosis and surveillance.

Respiratory physicians, pulmonology staff of all grades, TB specialists and health workers at the primary health care level may be points of reference for patients with pulmonary complications of COVID-19. They should familiarize themselves with the most current WHO recommendations for the supportive treatment and containment of COVID-19.

TB programme staff with their experience and capacity, including in active case finding and contact tracing, are well placed to support the COVID-19 response.

Various digital technologies used in TB programmes can support the COVID-19 response, including adherence support, electronic medical records and eLearning.

364、**[What measures should be in place to protect staff working in tuberculosis laboratories and healthcare facilities, and community health workers, from COVID-19 infection?](https://www.who.int/emergencies/diseases/novel-coronavirus-2019/question-and-answers-hub/q-a-detail/coronavirus-disease-covid-19-tuberculosis)**

Health care facilities, including those that diagnose and care for tuberculosis (TB) and lung diseases, are bound to receive patients with COVID-19, many of whom may be undiagnosed. Additional measures may be needed to avoid that staff in these centres are exposed to COVID-19 infection.

The [Information Note](https://www.who.int/news/item/05-05-2021-updated-who-information-note-ensuring-continuity-of-tb-services-during-the-covid-19-pandemic) includes additional, temporary measures that should be considered. These include alternative arrangements to reduce visits for TB follow-up, precautions for sputum collection, transportation and testing. The note also includes a description of ethical obligations in this setting.

Existing WHO recommendations for infection prevention and control for TB and for COVID-19 should be strictly implemented, including personal protection equipment.

Lessons learnt over many years of TB infection prevention and control, contact tracing, investigation and management can benefit efforts to stop the spread of COVID-19.

365、**[How can we protect people seeking tuberculosis care during the COVID-19 pandemic?](https://www.who.int/emergencies/diseases/novel-coronavirus-2019/question-and-answers-hub/q-a-detail/coronavirus-disease-covid-19-tuberculosis)**

In a context of widespread restriction of movement of the population in response to the pandemic and isolation of COVID-19 patients, communication with the healthcare services should be maintained so that people with tuberculosis (TB), especially those most vulnerable, get essential services. This includes management of adverse drug reactions and co-morbidities, nutritional and mental health support, and restocking of the supplies of medicines.

Enough TB medicines will need to be dispensed to the patient or caregiver to last until the next visit. This will limit interruption or unnecessary visits to the clinic. Mechanisms to deliver medicines at home and even to collect specimens for follow-up testing may become expedient. Home-based TB treatment is bound to become more common. Alternative arrangements to reduce clinic visits may involve limiting appointments to specific times to avoid exposure to other clinic attendees; using digital technologies to maintain treatment support. Community health workers become more critical as treatment is more decentralized.

More TB patients will probably start their treatment at home and therefore limiting the risk of household transmission of TB during the first few weeks is important.

Vulnerable populations who have poor access to healthcare should not get further marginalized during the pandemic.

366、**[Can tuberculosis and COVID-19 be tested on the same type of specimen?](https://www.who.int/emergencies/diseases/novel-coronavirus-2019/question-and-answers-hub/q-a-detail/coronavirus-disease-covid-19-tuberculosis)**

The diagnostic methods for tuberculosis (TB) and COVID-19 are quite distinct and commonly require different specimens.

Sputum, as well as many other biological specimens, can be used to diagnose TB using culture or molecular techniques.

Tests for COVID-19 are done most commonly by nasopharyngeal or oropharyngeal swab or wash in ambulatory patients, but sputum or endotracheal aspirate or bronchoalveolar lavage may be used in patients with severe respiratory disease.  Molecular testing is the currently recommended method for the identification of infectious COVID-19 and just as for TB, serological assays are not recommended for the routine diagnosis of COVID-19.

The pipeline for COVID-19 diagnostics has flourished impressively within a few months. Amongst these is the Xpert® Xpress SARS-CoV-2 cartridge for use on GeneXpert machines, which are machines used in TB diagnosis. WHO is currently evaluating this cartridge as well as other tests. Additional resources to roll out COVID-19 testing should be mobilized rather than relying only on existing resources that are used for TB, to ensure that the diagnostic coverage for TB is maintained as necessary.

367、**[Should all people being evaluated for tuberculosis also be tested for COVID-19 and vice-versa?](https://www.who.int/emergencies/diseases/novel-coronavirus-2019/question-and-answers-hub/q-a-detail/coronavirus-disease-covid-19-tuberculosis)**

Testing of the same patient for both tuberculosis (TB) and COVID-19 would generally be indicated for three main reasons, subject to the specific setting in each country:

1. clinical features that are common to both diseases; or
2. simultaneous exposure to both diseases; or
3. presence of a risk factor

As the pandemic advances, more people of all ages, including TB patients, will be exposed to COVID-19. The [Information Note](https://www.who.int/tb/COVID_19considerations_tuberculosis_services.pdf) contains further considerations for simultaneous testing for the two diseases.

368、**[Is tuberculosis treatment different in people who have both TB and COVID-19?](https://www.who.int/emergencies/diseases/novel-coronavirus-2019/question-and-answers-hub/q-a-detail/coronavirus-disease-covid-19-tuberculosis)**

In most cases tuberculosis (TB) treatment is not different in people with or without COVID-19 infection.

Experience on joint management of both COVID-19 infection and TB remains limited. However, suspension of TB treatment in COVID-19 patients should be exceptional. TB preventive treatment, treatment for drug-susceptible or drug-resistant TB disease should continue uninterrupted to safeguard the patient’s health, reduce transmission and prevent the development of drug-resistance.

While treatment trials are ongoing, no medication is currently recommended for COVID-19 and therefore no cautions on drug-drug interactions are indicated at present. TB patients on treatment should nonetheless be asked if they are taking any medicines, including traditional cures, that may interact with their medication.

Effective treatments to prevent TB and to treat active TB have been scaled up and are in use worldwide. The risk of death in TB patients approaches 50% if left untreated and may be higher in the elderly or in the presence of comorbidity. It is critical that TB services are not disrupted during the COVID-19 response.

Gathering evidence as this pandemic unfolds will be very important, while upholding the norms of professional conduct and patient confidentiality when handling clinical details.

369、**[Does the BCG vaccine protect people from COVID-19?](https://www.who.int/emergencies/diseases/novel-coronavirus-2019/question-and-answers-hub/q-a-detail/coronavirus-disease-covid-19-tuberculosis)**

There is no evidence at this point that the Bacille Calmette-Guérin vaccine (BCG) protects people against infection with COVID-19 virus. Clinical trials addressing this question are underway, and WHO will evaluate the evidence when it is available. In the absence of evidence, WHO does not recommend BCG vaccination for the prevention of COVID-19. WHO continues to recommend reserving BCG for neonatal vaccination in settings with a high risk of tuberculosis.

- [More information](https://www.who.int/news-room/commentaries/detail/bacille-calmette-gu%C3%A9rin-(bcg)-vaccination-and-covid-19)

#### [Coronavirus disease (COVID-19): Vaccine access and allocation（370-372）](https://www.who.int/emergencies/diseases/novel-coronavirus-2019/question-and-answers-hub/q-a-detail/coronavirus-disease-(covid-19)-vaccine-access-and-allocation)

370、**[As safe and effective vaccines for COVID-19 are approved, who should be the first to receive them?](https://www.who.int/emergencies/diseases/novel-coronavirus-2019/question-and-answers-hub/q-a-detail/coronavirus-disease-(covid-19)-vaccine-access-and-allocation)**

WHO believes that everyone, everywhere who could benefit from safe and effective COVID-19 vaccines should have access as quickly as possible, starting with those at highest risk of serious disease or death.

WHO’s [Strategic Advisory Group of Experts on Immunization (SAGE)](https://www.who.int/groups/strategic-advisory-group-of-experts-on-immunization/covid-19-materials) released two key documents to help guide the allocation and prioritization of populations to receive COVID-19 vaccines:

- The [Values Framework for the allocation and prioritization of COVID-19 vaccination](https://apps.who.int/iris/bitstream/handle/10665/334299/WHO-2019-nCoV-SAGE_Framework-Allocation_and_prioritization-2020.1-eng.pdf?ua=1), which offers high-level guidance globally on the values and ethical considerations regarding allocation of COVID-19 vaccines between countries, and offers guidance nationally on the prioritization of groups for vaccination within countries while supply is limited.
- The [Roadmap for Prioritizing Population Groups for Vaccines against COVID-19](https://www.who.int/publications-detail-redirect/WHO-2019-nCoV-Vaccines-SAGE-Roadmap), which recommends public health strategies and target priority groups for different levels of vaccine availability and epidemiologic settings.
  - The SAGE Roadmap has now been [updated](https://www.who.int/news/item/16-07-2021-updated-who-sage-roadmap-for-prioritizing-uses-of-covid-19-vaccines-in-the-context-of-limited-supply) and offers recommendations on how vaccines should be prioritized in countries with limited supply for maximum public health impact, taking into account the most recent evidence on COVID-19 vaccines and on the ongoing supply constraint issues faced by the COVAX Facility.  This update reflects data that has become available from clinical vaccine studies, as well as lessons learned from the early implementation of programmes.
  - The recommendations include expanding the groups included in Stage II of the prioritization roadmap to include pregnant women and children with underlying health conditions, and updates regarding clinical trials for lactating women.

In addition, WHO led the development of a [Fair Allocation Framework](https://www.who.int/publications/m/item/fair-allocation-mechanism-for-covid-19-vaccines-through-the-covax-facility) that aims to ensure that successful COVID-19 vaccines and treatments are shared equitably across all countries. This framework is a key part of the [Access to COVID-19 Tools (ACT) Accelerator](https://www.who.int/initiatives/act-accelerator), a global collaboration to accelerate development, production, and equitable access to COVID-19 tests, treatments, and vaccines. The framework advises that as safe and effective COVID-19 vaccines are authorized for use, all countries should receive doses in proportion to their population size to immunize the highest-priority groups. In the second phase, vaccines would continue to be deployed to all countries so that additional populations can be covered according to national priorities.

371、**[What is WHO doing to ensure that COVID-19 vaccines will be allocated fairly?](https://www.who.int/emergencies/diseases/novel-coronavirus-2019/question-and-answers-hub/q-a-detail/coronavirus-disease-(covid-19)-vaccine-access-and-allocation)**

WHO is one of the leaders of a global alliance known as [COVAX](https://www.gavi.org/covax-facility), the vaccine pillar of the ACT-Accelerator collaboration, which is working to accelerate the development and manufacturing of COVID-19 vaccines and ensure that there is fair and equitable access to these vaccines for all countries.

COVAX has allocated more than 170 million vaccines across 138 countries according to a framework developed by an expert group that includes ethicists, scientists, and other public health specialists  and vetted by WHO’s Member States. It continues to work to diversify both its portfolio of vaccines and channels for access to vaccines, Under this framework, COVID-19 vaccines are being rolled out in two phases. In the first phase, they have been allocated proportionally  to the population size to all participating countries at the same time, so as to protect those people at greatest risk of infection and of severe disease. In phase II, vaccine will be allocated to target countries with higher threat and vulnerability.

WHO’s Strategic Advisory Group of Experts (SAGE) has provided recommendations about which populations should be prioritized first. These include frontline health and care workers at high risk of infection, older adults, and those people at high risk of death because of underlying conditions like heart disease and diabetes. In the second phase of the roll-out, as more doses are produced, vaccines can go to groups less at risk of being infected or of suffering badly.

COVAX aims to provide at least 2 billion vaccine doses by the end of 2021 and 1.8 billion doses to 92 lower income economies by early 2022.

 The Facility has the possibility to increase those doses by another 1 billion as right of first refusal for several candidates in clinical trials supported by CEPI. Although these doses are not enough for everyone in all countries, it may be enough to end the acute stage of the crisis and put the world on the road to ending the pandemic.

The number of countries willing to donate vaccine has been increasing. It is important that they do so using the COVAX mechanism so the vaccine are allocated to countries that need them most. At least one billion doses could be donated by high income countries in 2021.

COVAX’s need for doses is greatest right now. Countries with higher coverage rates, which are due to receive doses soon should swap their places in supply queues with COVAX so that doses can be equitably distributed as quickly as possible. COVAX continues to be in close dialogue with countries who have expressed interest in sharing doses with the Facility and expects further announcements on this to take place over the coming weeks and months.

372、**[How is WHO helping countries prepare for COVID-19 vaccines rollouts?](https://www.who.int/emergencies/diseases/novel-coronavirus-2019/question-and-answers-hub/q-a-detail/coronavirus-disease-(covid-19)-vaccine-access-and-allocation)**

Along with accelerating COVID-19 vaccine research and helping scale up manufacturing capacity, WHO is working in close partnership with countries, regional colleagues, and other partners to develop needed policies, strengthen regulatory capacity, training courses, and guide countries in all the needed preparations for a programme to deliver COVID-19 vaccines. WHO, UNICEF, and partners are supporting countries in preparing for COVID-19 vaccine introduction. The Country Readiness and Delivery workstream – which is part of the ACT Accelerator – has developed a [toolbox](https://www.who.int/initiatives/act-accelerator/covax/covid-19-vaccine-country-readiness-and-delivery/country-readiness-and-delivery-faqs) with guidance, tools, and trainings.

WHO’s Strategic Advisory Group of Experts (SAGE) has issued interim policy recommendations for the rollout of all of the COVID-19 vaccines approved for emergency use. These recommendations can guide and support country decision-making bodies, such as the National Immunization Technical Advisory Groups, on the optimal use of existing COVID-19 vaccines.

WHO has also developed detailed technical guidance and adaptable planning tools and templates to help countries plan for COVID-19 vaccines considering the many aspects of readiness that need to be put in place. These areas include planning and implementation, data and monitoring, supply and logistics, and acceptance and demand. To support initial preparations, a vaccine introduction readiness assessment tool (VIRAT) has been developed and disseminated. It provides countries with an integrated roadmap of milestones and a framework for self-monitoring progress in preparing for vaccine introduction. This will help ensure that COVID-19 vaccines reach those in need as soon as they are available.

WHO advises every country to develop a National Deployment and Vaccination Plan (NDVP) for COVID-19 vaccines.  Having one plan in each country, that comprehensively describes all elements of the country’s approach to COVID-19 vaccine rollout will be crucial for a coordinated effort. [Guidance on developing that plan](https://www.who.int/publications-detail-redirect/WHO-2019-nCoV-Vaccine_deployment-2020.1) is available from WHO, including all elements that a country needs to consider.

#### [Coronavirus disease (COVID-19): Vaccine research and development（373-376）](https://www.who.int/emergencies/diseases/novel-coronavirus-2019/question-and-answers-hub/q-a-detail/coronavirus-disease-(covid-19)-vaccine-research-and-development)

373、**[How is the research and development process being accelerated without compromising safety?](https://www.who.int/emergencies/diseases/novel-coronavirus-2019/question-and-answers-hub/q-a-detail/coronavirus-disease-(covid-19)-vaccine-research-and-development)**

WHO and its partners are committed to accelerating the development of COVID-19 vaccines while maintaining the highest standards on safety.

Vaccines go through various phases of development and testing – there are usually three phases to clinical trials, with the last one designed to assess the ability of the product to protect against disease, which is called efficacy. All phases assess safety. The last phase, phase III, are usually conducted in a large number of people, often 10’s of thousands.  After that, the vaccine needs to go through a review by the national regulatory authority, who will decide if the vaccine is safe and effective enough to be put on the market, and a policy committee, who will decide how the vaccine should be used.

In the past, vaccines have been developed through a series of consecutive steps that can take many years. Now, given the urgent need for COVID-19 vaccines, unprecedented financial investments and scientific collaborations are changing how vaccines are developed. This means that some of the steps in the research and development process have been happening in parallel, while still maintaining strict clinical and safety standards. For example, some clinical trials are evaluating multiple vaccines at the same time. It is the scale of the financial and political commitments to the development of a vaccine that has allowed this accelerated development to take place. However, this does not make the studies any less rigorous.

The more vaccines in development the more opportunities there are for success.

Any longer-term safety assessment will be conducted through continued follow up of the clinical trial participants, as well as through specific studies and general pharmacovigilance of those being vaccinated in the roll out.  This represents standard practise for all newly authorized vaccines.

More information about COVID-19 vaccine development is available [here](https://www.who.int/emergencies/diseases/novel-coronavirus-2019/covid-19-vaccines).

374、**[What are human challenge studies? What is WHO’s view on these studies?](https://www.who.int/emergencies/diseases/novel-coronavirus-2019/question-and-answers-hub/q-a-detail/coronavirus-disease-(covid-19)-vaccine-research-and-development)**

In a regular vaccine study, one group of volunteers at risk for a disease is given an experimental vaccine, and another group is not; researchers monitor both groups over time and compare outcomes to see if the vaccine is safe and effective.

In a human challenge vaccine study, healthy volunteers are given an experimental vaccine, and then deliberately exposed to the organism causing the disease to see if the vaccine works. Some scientists believe that this approach could accelerate COVID-19 vaccine development, in part because it would require far fewer volunteers than a typical study.

However, there are important ethical considerations that must be addressed – particularly for a new disease like COVID-19, which we do not yet fully understand and are still learning how to treat; it may be difficult for the medical community and potential volunteers to properly estimate the potential risks of participating in a COVID-19 human challenge study. For more information, [see this WHO publication on the ethics of COVID-19 human challenge studies](https://www.who.int/publications-detail-redirect/WHO-2019-nCoV-Ethics_criteria-2020.1).

375、**[Who should participate in clinical trials for COVID-19 vaccines?](https://www.who.int/emergencies/diseases/novel-coronavirus-2019/question-and-answers-hub/q-a-detail/coronavirus-disease-(covid-19)-vaccine-research-and-development)**

Small (phase I) safety studies of COVID-19 vaccines should enroll healthy adult volunteers. Larger (phase II and III) studies should include volunteers that reflect the populations for whom the vaccines are intended. This means enrolling people from diverse geographic areas, racial and ethnic backgrounds, genders, and ages, as well as those with underlying health conditions that put them at higher risk for COVID-19. Including these groups in clinical trials is the only way to make sure that a vaccine will be safe and effective for everyone who needs it.

376、**[How do I volunteer for a COVID-19 vaccine trial?](https://www.who.int/emergencies/diseases/novel-coronavirus-2019/question-and-answers-hub/q-a-detail/coronavirus-disease-(covid-19)-vaccine-research-and-development)**

Opportunities to volunteer for a COVID-19 vaccine trial vary from country to country. If you are interested in volunteering, check with local health officials or research institutions or email [rdblueprint@who.int](mailto:rdblueprint@who.int) for more information about vaccine trials.

#### [Coronavirus disease (COVID-19): Vaccines（377-408）](https://www.who.int/emergencies/diseases/novel-coronavirus-2019/question-and-answers-hub/q-a-detail/coronavirus-disease-(covid-19)-vaccines)

377、**[What vaccines are there against COVID-19?](https://www.who.int/emergencies/diseases/novel-coronavirus-2019/question-and-answers-hub/q-a-detail/coronavirus-disease-(covid-19)-vaccines)**

There are several COVID-19 vaccines validated for use by WHO (given Emergency Use Listing). The first mass vaccination programme started in early December 2020 and the number of vaccination doses administered is updated on a daily basis on the [COVID-19 dashboard.](https://covid19.who.int/)

The WHO Emergency Use Listing process determines whether a product can be recommended for use based on all the available data on safety and efficacy and on its suitability in low- and middle-income countries. Vaccines are assessed to ensure they meet acceptable standards of quality, safety and efficacy using clinical trial data, manufacturing and quality control processes. The assessment weighs the threat posed by the emergency as well as the benefit that would accrue from the use of the product against any potential risks.

In line with their national regulations and legislation, countries have the autonomy to issue emergency use authorizations for any health product.  Domestic emergency use authorizations are issued at the discretion of countries and not subject to WHO approval.

As of 12 January 2022, the following vaccines have obtained EUL:

- [The Pfizer/BioNTech Comirnaty vaccine](https://www.who.int/news-room/feature-stories/detail/who-can-take-the-pfizer-biontech-covid-19--vaccine-what-you-need-to-know), 31 December 2020.
- [The SII/COVISHIELD and AstraZeneca/AZD1222 vaccines](https://www.who.int/news-room/feature-stories/detail/the-oxford-astrazeneca-covid-19-vaccine-what-you-need-to-know), 16 February 2021.
- [The Janssen/Ad26.COV 2.S vaccine developed by Johnson & Johnson](https://www.who.int/news-room/feature-stories/detail/the-j-j-covid-19-vaccine-what-you-need-to-know), 12 March 2021.
- [The Moderna COVID-19 vaccine (mRNA 1273)](https://www.who.int/news-room/feature-stories/detail/the-moderna-covid-19-mrna-1273-vaccine-what-you-need-to-know), 30 April 2021.
- [The Sinopharm COVID-19 vaccine](https://www.who.int/news-room/feature-stories/detail/the-sinopharm-covid-19-vaccine-what-you-need-to-know), 7 May 2021.
- [The Sinovac-CoronaVac vaccine,](https://www.who.int/news-room/feature-stories/detail/the-sinovac-covid-19-vaccine-what-you-need-to-know) 1 June 2021.
- [The Bharat Biotech BBV152 COVAXIN vaccine](https://www.who.int/news-room/feature-stories/detail/the-bharat-biotech-bbv152-covaxin-vaccine-against-covid-19-what-you-need-to-know), 3 November 2021.
- [The Covovax (NVX-CoV2373) vaccine](https://www.who.int/news-room/feature-stories/detail/the-novavax-vaccine-against-covid-19-what-you-need-to-know), 17 December 2021.

[The](https://www.who.int/news-room/feature-stories/detail/the-novavax-vaccine-against-covid-19-what-you-need-to-know) [Nuvaxovid (NVX-CoV2373) vaccine](https://www.who.int/news-room/feature-stories/detail/the-novavax-vaccine-against-covid-19-what-you-need-to-know), 20 December 2021

Read more:

- [Other COVID-19 vaccine products and the progress of their regulatory review by WHO](https://extranet.who.int/pqweb/sites/default/files/documents/Status_COVID_VAX_16Feb2021.pdf)
- [How vaccines are developed](https://www.who.int/news-room/feature-stories/detail/how-are-vaccines-developed)
- [Manufacturing, safety and quality control of vaccines](https://www.who.int/news-room/feature-stories/detail/manufacturing-safety-and-quality-control)

378、**[What are the benefits of getting vaccinated?](https://www.who.int/emergencies/diseases/novel-coronavirus-2019/question-and-answers-hub/q-a-detail/coronavirus-disease-(covid-19)-vaccines)**

Getting vaccinated could save your life. COVID-19 vaccines provide strong protection against serious illness, hospitalization and death. There is also some evidence that being vaccinated will make it less likely that you will pass the virus on to others, which means your decision to get the vaccine also protects those around you.

Even after getting vaccinated, keep taking precautions to protect yourself, family, friends and anyone else you may come into contact with. COVID-19 vaccines are highly effective, but some people will still get ill from COVID-19 after vaccination. There is also still a chance that you could also pass the virus on to others who are not vaccinated. Stay at least 1 metre away from other people, wear a properly fitted mask over your nose and mouth when you can’t keep this distance, avoid poorly ventilated places and settings, clean your hands frequently, stay home if unwell and get tested, and stay informed about how much virus is circulating in the areas where you travel, live and work.

379、**[Who should get vaccinated against COVID-19?](https://www.who.int/emergencies/diseases/novel-coronavirus-2019/question-and-answers-hub/q-a-detail/coronavirus-disease-(covid-19)-vaccines)**

WHO recommends that while vaccine supply is limited, the people at highest risk of COVID-19 are vaccinated first. This includes people who are more likely to get severe disease if they are infected (older persons and people with existing health conditions) and people who are more likely to be exposed to the virus (such as health workers). People who are pregnant have a higher risk of serious illness and preterm birth if they are infected with COVID-19, so WHO recommends that they are also prioritized for vaccination, once the first priority groups have been vaccinated.

If you live in a country where vaccines are available to more people beyond these priority groups, get vaccinated as soon as it is your turn.

WHO-authorized COVID-19 vaccines are safe for most people of 18 years and older, including those with pre-existing conditions of any kind such as auto-immune disorders. These conditions include hypertension, diabetes, asthma, pulmonary, liver and kidney disease, as well as chronic infections that are stable and controlled.

The Pfizer vaccine can be safely administered to children from 5 years of age. Both Moderna and Pfizer vaccines are licensed for use in children from 12 years of age.

Vaccine trials for the use of other COVID-19 vaccines in children and adolescents are ongoing and WHO will update its recommendations when the evidence or epidemiological situation justifies a change in policy.

Children and adolescents above 5 years of age with comorbidities that put them at significantly higher risk of serious COVID-19 may be offered vaccination alongside other high priority groups.

However, WHO recommends that countries should vaccinate healthy children only when high vaccine coverage with two doses has been achieved in higher priority-use groups, as identified in the [WHO Prioritization Roadmap](https://www.who.int/publications-detail-redirect/WHO-2019-nCoV-Vaccines-SAGE-Roadmap).

Read more:

- [Access and allocation: how will there be fair and equitable allocation of limited supplies?](https://www.who.int/news-room/feature-stories/detail/access-and-allocation-how-will-there-be-fair-and-equitable-allocation-of-limited-supplies)

 380、**[Who should not be vaccinated against COVID-19?](https://www.who.int/emergencies/diseases/novel-coronavirus-2019/question-and-answers-hub/q-a-detail/coronavirus-disease-(covid-19)-vaccines)**

There are very few conditions that would exclude someone from being vaccinated, but you should NOT be vaccinated if:

- You have a history of severe allergic reactions/anaphylaxis to any of the ingredients of the COVID-19 vaccine, in order to avoid possible adverse effects.
- You have a fever over 38.5ºC on the day of your vaccine appointment. Postpone until you have recovered.
- You currently have confirmed or suspected COVID-19. Wait until you have completed the mandated isolation period and your acute symptoms have passed to get vaccinated.

COVID-19 vaccines are safe for people taking blood thinners, but you should let the person giving you the vaccine know about any medication you are taking BEFORE you are given the vaccine.

In addition to the general recommendations above, each vaccine may have specific considerations for specific populations and health conditions. Talk to your doctor for advice about your specific situation.

381、**[Should I be vaccinated if I have had COVID-19?](https://www.who.int/emergencies/diseases/novel-coronavirus-2019/question-and-answers-hub/q-a-detail/coronavirus-disease-(covid-19)-vaccines)**

Yes. Even if you have already had COVID-19, you should be vaccinated. The protection that someone gains from having COVID-19 will vary greatly from person to person. The immunity people get from being vaccinated after having a natural infection is consistently very strong. Getting vaccinated even if you have had COVID-19 means you are more likely to be protected for longer.

There is currently no evidence to determine the optimal time that you should wait to be vaccinated after having COVID-19. Persons with laboratory-confirmed COVID-19 infection can consider delaying vaccination for 6 months based on the fact that natural infection leads to some protection against infection. Ask your health worker for advice.

382、**[Which COVID-19 vaccine should I get?](https://www.who.int/emergencies/diseases/novel-coronavirus-2019/question-and-answers-hub/q-a-detail/coronavirus-disease-(covid-19)-vaccines)**

All vaccines with WHO Emergency Use Listing are highly effective at preventing serious illness, hospitalization and death due to COVID-19. You should accept the vaccine you are offered first and get vaccinated as soon as it is your turn to reduce your risk.

Do not delay getting vaccinated, unless advised to by your health care provider, as this could put you at risk of COVID-19. Getting vaccinated could save your life.

In April 2020, WHO published the minimum criteria for how effective COVID-19 vaccines should be to make them useful for fighting COVID-19. All current vaccines authorized by WHO meet these criteria. To fully understand how effective different vaccines are, we need more real world data. This will come as more people are vaccinated.

The best COVID-19 vaccine is the one available to you soonest.

Read more on [the different types of COVID-19 vaccines](https://www.who.int/news-room/feature-stories/detail/the-race-for-a-covid-19-vaccine-explained). You can also learn about the individual COVID-19 vaccines on our information pages for the different vaccines: [AstraZeneca](https://www.who.int/news-room/feature-stories/detail/the-oxford-astrazeneca-covid-19-vaccine-what-you-need-to-know), [Covaxin](https://www.who.int/news-room/feature-stories/detail/the-bharat-biotech-bbv152-covaxin-vaccine-against-covid-19-what-you-need-to-know), [Sinovac](https://www.who.int/news-room/feature-stories/detail/the-sinovac-covid-19-vaccine-what-you-need-to-know), [Sinopharm](https://www.who.int/news-room/feature-stories/detail/the-sinopharm-covid-19-vaccine-what-you-need-to-know), [Johnson and Johnson (J&J) Janssen](https://www.who.int/news-room/feature-stories/detail/the-j-j-covid-19-vaccine-what-you-need-to-know), [Moderna](https://www.who.int/news-room/feature-stories/detail/the-moderna-covid-19-mrna-1273-vaccine-what-you-need-to-know), [Pfizer](https://www.who.int/news-room/feature-stories/detail/who-can-take-the-pfizer-biontech-covid-19--vaccine-what-you-need-to-know).

383、**[How quickly could COVID-19 vaccines stop the pandemic?](https://www.who.int/emergencies/diseases/novel-coronavirus-2019/question-and-answers-hub/q-a-detail/coronavirus-disease-(covid-19)-vaccines)**

COVID-19 vaccines are an important tool to stop the pandemic, but they will not do so on their own. Public health and social measures such as surveillance, contact tracing, isolation and individual protective behaviours such as staying at least 1 metre away from other people, wearing a properly fitted mask over your nose and mouth, avoiding poorly ventilated places and settings, staying home if unwell, covering coughs and sneezes and cleaning your hands frequently remain essential to breaking the chain of transmission.

The impact of COVID-19 vaccines on the pandemic will depend on several factors. These include the effectiveness of the vaccines; how quickly they are approved, manufactured, and delivered; the possible development of other variants, and how many people get vaccinated.

384、**[What is the difference between the immunity you develop from getting COVID-19 and immunity from getting a COVID-19 vaccine?](https://www.who.int/emergencies/diseases/novel-coronavirus-2019/question-and-answers-hub/q-a-detail/coronavirus-disease-(covid-19)-vaccines)**

We are still learning about how long immunity to COVID-19 lasts from natural infection, and from vaccination. We are now starting to see evidence that the immunity you get after having COVID-19 can be strong. However, the type of immunity that's developed after infection varies from person to person, making it less predictable than immunity after vaccination. Scientists are working hard to understand this better.

What we do know is that COVID-19 is a life-threatening disease that can have long-term consequences. We also know that the WHO-authorised COVID-19 vaccines have been safely given to billions of people. It is much safer to get vaccinated than it is to risk getting COVID-19. Get vaccinated as soon as it’s your turn and keep doing everything you can to protect yourself and others.

As we learn more about COVID-19 and immunity, WHO continues to update our guidance and recommendations.

385、**[Do the COVID-19 vaccines protect against virus variants?](https://www.who.int/emergencies/diseases/novel-coronavirus-2019/question-and-answers-hub/q-a-detail/coronavirus-disease-(covid-19)-vaccines)**

The COVID-19 vaccines with WHO Emergency Use Listing (EUL) provide different levels of protection to infection, mild disease, severe disease, hospitalization and death. Research is ongoing by thousands of scientists around the world to better understand how new virus mutations and variants affect the effectiveness of the different COVID-19 vaccines.

In general, the COVID-19 vaccines are very effective at preventing serious illness, hospitalization and death from all current virus variants. They are less effective at protecting you against infection and mild disease than they were for earlier virus variants; but if you do get ill after being vaccinated, your symptoms are more likely to be mild.

Remember that while the COVID-19 vaccines authorised by WHO are incredibly effective at reducing your risk of developing serious illness and death, no vaccine is 100% effective. A small percentage of people will still get ill from COVID-19 even though they have been vaccinated. Currently there is limited information about the risk of vaccinated people passing the virus to others if they are infected. This makes it very important to continue to practice public health and social measures, even after you have been fully vaccinated

386、**[What types of COVID-19 vaccines are there? How do they work?](https://www.who.int/emergencies/diseases/novel-coronavirus-2019/question-and-answers-hub/q-a-detail/coronavirus-disease-(covid-19)-vaccines)**

Scientists around the world are continuing to develop many potential vaccines for COVID-19. These vaccines are all designed to teach the body’s immune system to safely recognize and block the virus that causes COVID-19.

Several different types of potential vaccines for COVID-19 have been developed, including:

- Inactivated or weakened virus vaccines, which use a form of the virus that has been inactivated or weakened so it doesn’t cause disease but still generates an immune response.
- Protein-based vaccines, which use harmless fragments of proteins or protein shells that mimic the COVID-19 virus to safely generate an immune response.
- Viral vector vaccines, which use a safe virus that cannot cause disease but serves as a platform to produce coronavirus proteins to generate an immune response.
- RNA and DNA vaccines, a cutting-edge approach that uses genetically engineered RNA or DNA to generate a protein that itself safely prompts an immune response.

Read more:

- For more information about all COVID-19 vaccines in development, see [*COVID-19 vaccine tracker and landscape*](https://www.who.int/publications/m/item/draft-landscape-of-covid-19-candidate-vaccines), which is being updated regularly.
- [The different types of COVID-19 vaccines.](https://www.who.int/news-room/feature-stories/detail/the-race-for-a-covid-19-vaccine-explained)

387、**[Can I have the second or third dose with a different vaccine than the first dose?](https://www.who.int/emergencies/diseases/novel-coronavirus-2019/question-and-answers-hub/q-a-detail/coronavirus-disease-(covid-19)-vaccines)**

It is safe and effective for you to receive a second or a third dose of a different COVID-19 vaccine. If you’re offered a different type of vaccine, you can go ahead and get vaccinated. WHO considers two doses of any WHO EUL vaccine to be a complete primary series, See the full list of COVID-19 vaccines with WHO EUL [here](https://extranet.who.int/pqweb/vaccines/vaccinescovid-19-vaccine-eul-issued).

By mixing and matching vaccines, countries are able to maximise vaccine impact in the event of constrained or limited supply.

People over 60 who have received two doses of Sinovac and Sinopharm should be given a third dose to restore waning immunity over time. WHO Strategic Advisory Group of Experts on Immunization (SAGE) has stated that Pfizer or AstraZeneca can also be used for the third dose, if the original vaccine is not available.

Read more:

- [Side effects of COVID-19 vaccines.](https://www.who.int/news-room/feature-stories/detail/side-effects-of-covid-19-vaccines)

388、**[What are the side effects of COVID-19 vaccines?](https://www.who.int/emergencies/diseases/novel-coronavirus-2019/question-and-answers-hub/q-a-detail/coronavirus-disease-(covid-19)-vaccines)**

Like with any vaccine, some people will experience mild to moderate side effects after being vaccinated against COVID-19. This is a normal sign that the body is developing protection. Side effects to COVID-19 vaccines include a fever, tiredness, headache, muscle ache, chills, diarrhoea and pain or redness at the injection site. Not everyone will experience side effects. Most side effects go away within a few days on their own. You can manage any side effects with rest, plenty of non-alcoholic liquids and taking medication to manage pain and fever, if needed.

If you are worried that the side effects that you are experiencing are unusual, if the pain in the arm where you got the injection gets worse after 24 hours or your side effects don’t go away in a few days, contact your healthcare provider for advice.

More serious or long-lasting side effects to COVID-19 vaccines are possible but extremely rare. If you experience difficulty breathing, chest pain, confusion, loss of speech or mobility after your vaccine, contact your healthcare provider immediately. Vaccines are continually monitored for as long as they are in use to detect and respond to rare adverse events.

Read more:

- The [side effects of COVID-19 vaccines.](https://www.who.int/news-room/feature-stories/detail/side-effects-of-covid-19-vaccines)

389、**[Does having side effects mean that the vaccine is working? What does having no side effects mean?](https://www.who.int/emergencies/diseases/novel-coronavirus-2019/question-and-answers-hub/q-a-detail/coronavirus-disease-(covid-19)-vaccines)**

The vaccine stimulates your immune system to protect you from the virus. This process can sometimes cause side effects like fever, chills or headache, but not everyone will experiences any side effect. The presence or magnitude of the reaction you may have vaccination does not predict or reflect your immune response to the vaccine.

You do not have to have side effects in order to be protected.

Read more:

- [Side effects of COVID-19 vaccines](https://www.who.int/news-room/feature-stories/detail/side-effects-of-covid-19-vaccines)

390、**[What should I do if I think my side effects are unusual?](https://www.who.int/emergencies/diseases/novel-coronavirus-2019/question-and-answers-hub/q-a-detail/coronavirus-disease-(covid-19)-vaccines)**

If you are worried about your side effects, contact your healthcare provider and let them know about your recent vaccination.

391、**[Is there a link between COVID-19 vaccines and allergic reactions?](https://www.who.int/emergencies/diseases/novel-coronavirus-2019/question-and-answers-hub/q-a-detail/coronavirus-disease-(covid-19)-vaccines)**

In very rare cases, some people may experience an allergic reaction after being vaccinated against COVID-19. A severe allergic reaction – such as anaphylaxis – is a very rare side effect of any vaccine.

If you have a history of allergic reactions, talk to your healthcare provider before you get vaccinated. They will be able to give you advice. In some cases, precautions will need to be taken for people who have known allergies to previous doses of the vaccine or known components of the vaccine.

Healthcare workers administering COVID-19 vaccines should be trained to recognise and treat serious allergic reactions. This is why people being vaccinated against COVID-19 will be asked to stay at the vaccination site for a period of time following their injection, to ensure that anyone experiencing an allergic reaction can receive prompt treatment.

Reports of adverse events following COVID-19 vaccination (including allergic reactions) are closely monitored by national authorities and international bodies, including WHO for the early detection of serious side effects.

392、**[What happens if a serious adverse event is reported after vaccination?](https://www.who.int/emergencies/diseases/novel-coronavirus-2019/question-and-answers-hub/q-a-detail/coronavirus-disease-(covid-19)-vaccines)**

As with any vaccine, it is essential to closely monitor the safety and effectiveness of COVID-19 vaccines that are used in immunization programmes. If a serious health problem is reported following vaccination, a thorough investigation should take place by the public health programme in the country.

It is rare to find that health problems occurring following receipt of a vaccine are actually caused by the vaccine itself. Health problems following vaccination are most often found to be coincidental and entirely unrelated to vaccination. Sometimes they are related to how the vaccine has been stored, transported, or administered. Errors related to the delivery of the vaccine can be prevented by better training health workers and strengthening supply chains.

The results of the investigation will then inform next steps. The safety of COVID-19 vaccines is the top priority of the World Health Organization.

Find out more about [how the safety of vaccines is monitored.](https://www.who.int/news-room/feature-stories/detail/safety-of-covid-19-vaccines)

393、**[Can I still get COVID-19 after I have been vaccinated?](https://www.who.int/emergencies/diseases/novel-coronavirus-2019/question-and-answers-hub/q-a-detail/coronavirus-disease-(covid-19)-vaccines)**

Yes. The maximum level of protection from COVID-19 vaccines is not reached until several weeks after full vaccination. If you have a two-dose vaccine, this means that you don’t get full immunity until 2 – 4 weeks after the second dose. You can still become infected and ill during this time.

While COVID-19 vaccines are highly effective against serious illness, hospitalisation and death, no vaccine is 100% effective. As a result, a number of vaccinated people will get infected and may fall ill with COVID-19 in spite of being fully vaccinated. This is known as a ‘breakthrough infection’ or ‘breakthrough case’. With more infectious virus variants such as Delta, we are seeing more breakthrough infections and cases.

Breakthrough infections can happen with every vaccine, and do not mean that the vaccine does not work. According to data from the US CDC, unvaccinated people are at 11 times the risk of death from COVID-19 than vaccinated people. People who get COVID-19 after being vaccinated are much more likely to only experience mild symptoms; efficacy against serious illness and death remains high. Get vaccinated, as soon as it’s your turn.

Even once you are fully vaccinated, continue to practice the same prevention measures to protect yourself. Stay at least 1 metre away from other people, wear a well fitted mask over your nose and mouth when you can’t keep this distance, avoid poorly ventilated places and settings, clean your hands frequently, stay home if unwell and get tested and stay informed about how much virus is circulating in the areas where you travel, live and work.

394、**[Does being vaccinated stop me from infecting other people with COVID-19?](https://www.who.int/emergencies/diseases/novel-coronavirus-2019/question-and-answers-hub/q-a-detail/coronavirus-disease-(covid-19)-vaccines)**

There is some evidence that being fully vaccinated can prevent infection with the COVID-19 virus. This means that being vaccinated is likely to help protect people around you by making it less likely that you will pick up the virus and pass it on.

Research is ongoing to understand the extent to which being vaccinated stops you from becoming infected and passing the virus on to others. More data is needed to know the extent of this protection. There is still a chance you could pass the virus on.

Even once you are fully vaccinated, continue to practice the same prevention measures to protect other people. Stay at least 1 metre away from other people, wear a properly fitted mask over your nose and mouth when you can’t keep this distance, avoid poorly ventilated places and settings, clean your hands frequently, stay home if unwell and get tested, and stay informed about how much virus is circulating in the areas where you travel, live and work.

395、**[Can I stop taking precautions after being vaccinated?](https://www.who.int/emergencies/diseases/novel-coronavirus-2019/question-and-answers-hub/q-a-detail/coronavirus-disease-(covid-19)-vaccines)**

No. Even after getting vaccinated, keep taking precautions to protect yourself, family and friends if there is still COVID-19 in your area.

The maximum level of protection is not reached until several weeks after full vaccination. If you have a two-dose vaccine, this means that you don’t get full immunity until 2–4 weeks after the second dose.

COVID-19 vaccines are highly effective, but a small percentage of people will still get ill from COVID-19 after vaccination (this is known as a breakthrough infection). There is also still a chance that you could pass the virus on to others who are not vaccinated. Some people have not been vaccinated against COVID-19, cannot be vaccinated, or do not develop full immunity in response to COVID-19 vaccines because of having a weakened immune system. Continue to practice all protective behaviours to protect yourself and others.

Even once you are fully vaccinated, stay at least 1 metre away from other people, wear a properly fitted mask over your nose and mouth when you can’t keep this distance, avoid poorly ventilated places and settings, clean your hands frequently, stay home if unwell and get tested, stay informed about how much virus is circulating in the areas where you travel, live and work, and get vaccinated as soon as it is your turn.

396、**[How long does protection from COVID-19 vaccines last?](https://www.who.int/emergencies/diseases/novel-coronavirus-2019/question-and-answers-hub/q-a-detail/coronavirus-disease-(covid-19)-vaccines)**

We still don’t know exactly how long protection from COVID-19 vaccines lasts, but current data indicates that most people have strong protection against serious illness and death for at least 6 months.

There is increasing evidence that the effectiveness of COVID-19 vaccine against infection and mild symptoms can wane over time. In light of this, WHO Strategic Advisory Group of Experts on Immunization (SAGE) has advised that booster doses are offered to people 4-6 months after the primary series of vaccination is completed. A booster dose should be taken when offered, to strengthen protection against serious illness and death from COVID-19.

Immunity may reduce faster in people who are older or who have underlying medical conditions, or who have a high level of exposure to the virus, so SAGE recommends that boosters are offered to higher priority-use groups before vaccines are given to those in lower-priority use groups.

People over 60 and immune-compromised who have received two doses of Sinovac and Sinopharm should also be given an additional third dose as part of the primary series to reach sufficient immunity. A booster dose is recommended for these vaccines, for more details consult the interim recommendations.

To protect yourself, get vaccinated AND continue practicing the recommended protective behaviours against COVID-19, including regular hand-washing, physical distancing, and ventilation of rooms.

Read more:

- [Vaccine efficacy, effectiveness and protection.](https://www.who.int/news-room/feature-stories/detail/vaccine-efficacy-effectiveness-and-protection)

397、**[Who needs an additional dose of COVID-19 vaccines?](https://www.who.int/emergencies/diseases/novel-coronavirus-2019/question-and-answers-hub/q-a-detail/coronavirus-disease-(covid-19)-vaccines)**

People with compromised immune systems don’t always develop maximum immunity against COVID-19 after one or two doses, so may need an additional dose to protect them. WHO recommends that people who are moderately or severely immunocompromised should be offered an additional dose of COVID-19 vaccine.

For Sinovac and Sinopharm, WHO recommends that countries should consider offering a third dose of the vaccine to the vaccination schedule for those aged 60 and over as an extension of the primary series, once a high level of coverage of first and second doses has been achieved in the priority groups.

An additional dose is different from a booster dose. It is considered part of an extended primary series for people who do not develop sufficient protection after one or two doses. It is intended to help these individuals to develop better protection against COVID-19.

398、**[How many booster doses of COVID-19 vaccine do I need?](https://www.who.int/emergencies/diseases/novel-coronavirus-2019/question-and-answers-hub/q-a-detail/coronavirus-disease-(covid-19)-vaccines)**

A booster dose is an extra dose of vaccine administered to a vaccinated person that has completed a primary vaccination series, (currently one, two or three doses of COVID-19 vaccine depending on the vaccine and the population group) when, with time, the immunity and clinical protection has fallen below a rate deemed sufficient in that population.

The protection you get from COVID-19 vaccines can wane over time, evidence shows that the effectiveness wanes around 4-6 months after the primary series of vaccination has been completed. If you are offered a booster you should take it to strengthen your protection against serious disease.

WHO currently recommends that individuals over the age of 18 have 1 booster dose 4-6 months after completing the primary series. WHO does not currently recommend that children and young adults under the age of 18 receive a booster dose.

SAGE recommends that high-priority use groups are offered boosters, prior to continuing the roll-out of primary vaccination among lower-priority use groups.

SAGE is reviewing the available data on the need for additional booster doses to maintain protection against COVID-19 and will update the recommendations accordingly.

399、**[Can the COVID-19 vaccine cause a positive test result for the disease, such as for a PCR or antigen test?](https://www.who.int/emergencies/diseases/novel-coronavirus-2019/question-and-answers-hub/q-a-detail/coronavirus-disease-(covid-19)-vaccines)**

No, the COVID-19 vaccine will not cause a positive test result for a COVID-19 PCR or antigen laboratory test. This is because the tests check for active disease and not whether an individual is immune. However, because the COVID-19 vaccine prompts an immune response, it may be possible to test positive in an antibody (serology) test that measures COVID-19 immunity in an individual.

400、**[Once I have been vaccinated, can I safely travel internationally?](https://www.who.int/emergencies/diseases/novel-coronavirus-2019/question-and-answers-hub/q-a-detail/coronavirus-disease-(covid-19)-vaccines)**

You should follow national advice and the advice of the countries you are travelling to and from.  Some countries are allowing fully vaccinated people to avoid quarantine and testing on arrival. This is because these individuals are at a lower risk of COVID-19 and are less likely to get infected and pass the virus to others. But even once you are fully vaccinated, continue to practice the same prevention measures – no vaccine is 100% effective, and doing it all helps protect yourself and others. Stay at least 1 metre away from other people, wear a properly fitted mask over your nose and mouth when you can’t keep this distance, avoid poorly ventilated places and settings, clean your hands frequently, stay home if unwell and get tested, and stay informed about how much virus is circulating in the areas where you travel, live and work.

WHO does not support using proof of vaccination as a requirement for international travel. This would not be fair, given that there are not enough vaccines for everyone, and that some countries have more access to vaccines than others.

401、**[Is it safe for me to take antibiotics after the vaccine?](https://www.who.int/emergencies/diseases/novel-coronavirus-2019/question-and-answers-hub/q-a-detail/coronavirus-disease-(covid-19)-vaccines)**

There is no known influence or interaction between antibiotics and COVID-19 vaccines. If you are prescribed antibiotics by a health professional before or after your vaccination, you should go ahead and take the full course. However, if you have a temperature over 38.5 ºC at the time of your vaccination appointment, you should reschedule for when you feel better.

402、**[Is it safe to drink alcohol after getting a COVID-19 vaccine?](https://www.who.int/emergencies/diseases/novel-coronavirus-2019/question-and-answers-hub/q-a-detail/coronavirus-disease-(covid-19)-vaccines)**

There is no evidence that the safety or effectiveness of COVID-19 vaccines is affected by anything you eat or drink before or after getting vaccinated, including alcohol. However, drinking alcohol can add to the normal mild to moderate side effects that you might experience after vaccination, such as a headache and tiredness. Because of this, it is advisable to avoid drinking until any side effects following vaccination have passed. Learn more about the[side effects of COVID-19 vaccines.](https://www.who.int/news-room/feature-stories/detail/side-effects-of-covid-19-vaccines)

While COVID-19 vaccines are highly effective at preventing serious illness and death, there is still a chance you could be infected after being vaccinated. Continue to protect yourself and others by continuing to practice physical distancing, wear a well-fitted mask over your nose and mouth, clean your hands frequently, stay home if you feel unwell, cover coughs and sneezes and keep indoor spaces well ventilated. Drinking alcohol may make you less vigilant in practicing these behaviours and so can put you and others at risk.

Please keep in mind that drinking alcohol can increase your risk of other health problems.

403、**[Can children and adolescents get vaccinated against COVID-19?](https://www.who.int/emergencies/diseases/novel-coronavirus-2019/question-and-answers-hub/q-a-detail/coronavirus-disease-(covid-19)-vaccines)**

The Pfizer vaccine can be safely administered to children from 5 years of age. Both Moderna and Pfizer vaccines are licensed for use in children from 12 years of age.

WHO recommends that children aged 5 and above with comorbidities that put them at significant risk of severe COVID-19 should be offered vaccination, at a reduced dosage, alongside other high-risk groups.

Countries may now consider vaccinating healthy children from the age of 5 years of age and adolescents as part of their national vaccination strategies. However, WHO strongly recommends that countries should vaccinate children only when high vaccine coverage with primary vaccination series has been achieved in higher priority-use groups, as identified in the [WHO Prioritization Roadmap.](https://www.who.int/publications-detail-redirect/WHO-2019-nCoV-Vaccines-SAGE-Roadmap)

Vaccine trials for children and adolescents and other COVID-19 vaccines are ongoing and WHO will update its recommendations when the evidence or epidemiological situation justifies a change in policy.

COVID-19 has also been a more serious and dangerous disease among older people. While the supply of vaccines is constrained, the ongoing priority is to vaccinate those most at risk of serious illness who still have not been vaccinated in many parts of the world: older people, those with chronic health conditions and health workers.

Most children are at low risk of serious disease and vaccinating them would be primarily about reducing transmission. There is emerging evidence that vaccines may be less effective at reducing transmission of Omicron. This means that the most impactful thing that can be done to protect children while vaccines are still being prioritised for those most at risk is to continue to practice the protective behaviours: keep a safe distance from others and avoid crowds, wear a well-fitting mask covering your mouth and nose, keep indoor spaces well ventilated, clean hands regularly and cover coughs and sneezes. Caregivers should follow national guidelines around children staying home from school if unwell, getting tested for COVID-19 when showing symptoms

404、**[Can I get vaccinated against COVID-19 if I am pregnant?](https://www.who.int/emergencies/diseases/novel-coronavirus-2019/question-and-answers-hub/q-a-detail/coronavirus-disease-(covid-19)-vaccines)**

Yes, you can get vaccinated if you are pregnant. During pregnancy, you are at higher risk of serious illness caused by COVID-19. You are also at higher risk of delivering your baby prematurely if you contract COVID-19. While there is less data available on vaccination of pregnant people, evidence on the safety of COVID-19 vaccines during pregnancy has been growing, and no safety concerns have been identified. Especially in countries with high transmission, or if you have an occupation where you are at more risk of being exposed to COVID-19, the benefits of getting the vaccine outweigh potential risks. There is no risk of getting COVID-19 from the vaccine. Talk to your healthcare provider to make an informed decision about vaccination.

405、**[Should I get vaccinated if I want to have a baby in the future?](https://www.who.int/emergencies/diseases/novel-coronavirus-2019/question-and-answers-hub/q-a-detail/coronavirus-disease-(covid-19)-vaccines)**

Yes, you should get vaccinated if you are planning to get pregnant. There is no current evidence that suggests the COVID-19 vaccines interfere with fertility. There is currently no biological evidence that antibodies from COVID-19 vaccination or vaccine ingredients could cause any problems with reproductive organs. Getting vaccinated is the best thing you can do to protect yourself and the future health of your baby.

406、**[Should I get vaccinated against COVID-19 if I am breastfeeding?](https://www.who.int/emergencies/diseases/novel-coronavirus-2019/question-and-answers-hub/q-a-detail/coronavirus-disease-(covid-19)-vaccines)**

If you are breastfeeding, you should get vaccinated against COVID-19 as soon as it is your turn. None of the current COVID-19 vaccines have live virus in them. This means there is no risk of you transmitting COVID-19 to your baby through your breastmilk from the vaccine.  In fact, the antibodies you get after vaccination may go through your breast milk and help to protect your baby.

407、**[Is it safe to get vaccinated if I am menstruating?](https://www.who.int/emergencies/diseases/novel-coronavirus-2019/question-and-answers-hub/q-a-detail/coronavirus-disease-(covid-19)-vaccines)**

You should get vaccinated if you are menstruating. If you have your period on the day of your vaccination appointment, you can go ahead and get vaccinated. Menstruation isn’t a medical reason to not get the COVID-19 vaccine. If you have concerns or questions about your periods, do not hesitate to speak with your health care provider.

408、**[Could getting vaccinated against COVID-19 disrupt my menstrual cycle?](https://www.who.int/emergencies/diseases/novel-coronavirus-2019/question-and-answers-hub/q-a-detail/coronavirus-disease-(covid-19)-vaccines)**

There have been some reports of people experiencing disruption to their menstrual cycle after being vaccinated against COVID-19. There is not yet enough data available to know whether there is a connection between the vaccines and this disruption. Several large studies researching the impact of vaccines on menstrual cycles are ongoing. WHO will continue to monitor any new evidence regarding menstrual cycles. Information regarding these studies can be found [here](https://covid19.nih.gov/news-and-stories/covid-19-vaccines-and-menstrual-cycle) and [here](https://www.nichd.nih.gov/newsroom/news/083021-COVID-19-vaccination-menstruation).

If you have concerns or questions about your periods, do not hesitate to speak with a health care provider.

#### [Coronavirus disease (COVID-19): Vaccines safety(409-424)](https://www.who.int/emergencies/diseases/novel-coronavirus-2019/question-and-answers-hub/q-a-detail/coronavirus-disease-(covid-19)-vaccines-safety)

409、**[How do we know that COVID-19 vaccines are safe?](https://www.who.int/emergencies/diseases/novel-coronavirus-2019/question-and-answers-hub/q-a-detail/coronavirus-disease-(covid-19)-vaccines-safety)**

There are strict precautions in place to help ensure the safety of all COVID-19 vaccines. Before receiving validation from WHO and national regulatory agencies for emergency use, COVID-19 vaccines must undergo rigorous testing in clinical trials to prove that they meet internationally agreed benchmarks for safety and efficacy.

Unprecedented scientific collaborations have allowed COVID-19 vaccine research, development, and authorizations to be completed in record time – to meet the urgent need for these vaccines while maintaining high safety standards. As with all vaccines, WHO and regulatory authorities will continuously monitor the use of COVID-19 vaccines to identify and respond to any safety issues that might arise. Through that process, we ascertain that they remain safe for use around the world.

Read more:

- [Safety of COVID-19 vaccines.](https://www.who.int/news-room/feature-stories/detail/safety-of-covid-19-vaccines)

410、H[**ow does WHO ensure that COVID-19 vaccines are safe and effective?**](https://www.who.int/emergencies/diseases/novel-coronavirus-2019/question-and-answers-hub/q-a-detail/coronavirus-disease-(covid-19)-vaccines-safety)

The vaccines must be proven safe and effective in large Phase III clinical trials. Some COVID-19 vaccine candidates have already completed their Phase III trials and are being used globally, and many other vaccine candidates are being developed and are still in the trial phase.

Independent reviews of the efficacy and safety evidence are required by WHO for each vaccine candidate, including regulatory review and approval in the country where the vaccine is manufactured, before WHO considers it for emergency use listing.

An external panel of experts convened by WHO (the [Strategic Advisory Group of Experts on Immunization (SAGE)](https://www.who.int/emergencies/diseases/novel-coronavirus-2019/question-and-answers-hub/q-a-detail/392406B5-1A54-4E17-B66B-5F44CA2DA775), analyses the results from clinical trials, along with evidence on the disease, age groups affected, risk factors for disease, programmatic use and other information. SAGE then recommends whether and how the vaccines should be used. In addition to reviewing data for regulatory purposes, the evidence must also be reviewed for the purpose of policy recommendations on how the vaccines should be used.

The WHO’s Global Advisory Committee on Vaccine Safety (GACVS) monitors how approved vaccines behave in the real world and to identify any signals of adverse events following immunization. GACVS is an independent group of experts providing authoritative, scientific advice to WHO on vaccine safety issues of global or regional concern.

Read more:

- [Safety of COVID-19 vaccines.](https://www.who.int/news-room/feature-stories/detail/safety-of-covid-19-vaccines)

411、**[What systems are in place to monitor vaccine safety?](https://www.who.int/emergencies/diseases/novel-coronavirus-2019/question-and-answers-hub/q-a-detail/coronavirus-disease-(covid-19)-vaccines-safety)**

Vaccine safety monitoring is critical at national, regional, and global levels. After any vaccine is introduced, WHO works with vaccine manufacturers, health officials, national advisory committees and other partners to monitor for any safety concerns on an ongoing basis. Specific safety concerns that arise are then evaluated by WHO and an independent group of experts (the Global Advisory Committee on Vaccine Safety, or GACVS) in conjunction with the relevant national authorities.

As is standard practice in all national immunization programmes, WHO is supporting the implementation of safety monitoring systems for COVID-19 vaccines in every country.

Read more:

- [How the safety of vaccines is monitored.](https://www.who.int/news-room/feature-stories/detail/safety-of-covid-19-vaccines)

412、**[How will WHO inform the public about suspected or confirmed serious adverse events related to COVID-19 vaccines?](https://www.who.int/emergencies/diseases/novel-coronavirus-2019/question-and-answers-hub/q-a-detail/coronavirus-disease-(covid-19)-vaccines-safety)**

Suspected safety events officially reported to WHO go through a series of rapid verification steps involving the Global Advisory Committee on Vaccine Safety (GACVS). WHO then shares the results of these evaluations on its website and publishes the results of any assessments.

WHO also coordinates with local, regional, and national health officials to investigate vaccine safety concerns and advise on next steps.

Additional vaccine safety information is also made available through the [Vaccine Safety Net](https://www.vaccinesafetynet.org/), a global network of websites providing reliable information on vaccine safety that have been verified by WHO.

413、**[Under what circumstances should a COVID-19 vaccine be recalled?](https://www.who.int/emergencies/diseases/novel-coronavirus-2019/question-and-answers-hub/q-a-detail/coronavirus-disease-(covid-19)-vaccines-safety)**

In rare situations where a serious adverse reaction is suspected to be related to the vaccine itself, the vaccine may be temporarily suspended from use while the situation is assessed. Investigations will take place to determine what exactly caused the event, and corrective measures will be put in place. WHO works closely with vaccine manufacturers, health officials, researchers, and other partners to monitor any safety concerns and potential side effects.

Vaccine recalls or withdrawals due to safety issues are rare. Recalls are generally associated with problems identified during the monitoring of batches of vaccines through quality control systems, stability studies and reports from the field, including cold chain issues where some vaccines have not been stored at the right conditions and so are no longer safe or effective. In this case, people who have received a vaccine from that batch may need to be vaccinated again to ensure they are protected. This is why vaccines are so closely monitored – to ensure that any issues with their production, storage or use can be rapidly identified and resolved.

414、**[What are the side effects of COVID-19 vaccines?](https://www.who.int/emergencies/diseases/novel-coronavirus-2019/question-and-answers-hub/q-a-detail/coronavirus-disease-(covid-19)-vaccines-safety)**

Like with any vaccine, some people will experience mild to moderate side effects after being vaccinated against COVID-19. This is a normal sign that the body is developing protection. Side effects to COVID-19 vaccines include a fever, tiredness, headache, muscle ache, chills, diarrhoea and pain or redness at the injection site. Not everyone will experience side effects. Most side effects go away within a few days on their own. You can manage any side effects with rest, plenty of non-alcoholic liquids and taking medication to manage pain and fever, if needed.

If you are worried that the side effects that you are experiencing are unusual, if the pain in the arm where you got the injection gets worse after 24 hours or your side effects don’t go away in a few days, contact your healthcare provider for advice.

More serious or long-lasting side effects to COVID-19 vaccines are possible but extremely rare. If you experience difficulty breathing, chest pain, confusion, loss of speech or mobility after your vaccine, contact your healthcare provider immediately. Vaccines are continually monitored for as long as they are in use to detect and respond to rare adverse events.

Read more:

- The [side effects of COVID-19 vaccines.](https://www.who.int/news-room/feature-stories/detail/side-effects-of-covid-19-vaccines)

415、**[Does having side effects mean that the vaccine is working? What does having no side effects mean?](https://www.who.int/emergencies/diseases/novel-coronavirus-2019/question-and-answers-hub/q-a-detail/coronavirus-disease-(covid-19)-vaccines-safety)**

The vaccine stimulates your immune system to protect you from the virus. This process can sometimes cause side effects like fever, chills or headache, but not everyone will experiences any side effect. The presence or magnitude of the reaction you may have vaccination does not predict or reflect your immune response to the vaccine.

You do not have to have side effects in order to be protected.

Read more:

- [Side effects of COVID-19 vaccines.](https://www.who.int/news-room/feature-stories/detail/side-effects-of-covid-19-vaccines)

416、**[What should I do if I think my side effects are unusual?](https://www.who.int/emergencies/diseases/novel-coronavirus-2019/question-and-answers-hub/q-a-detail/coronavirus-disease-(covid-19)-vaccines-safety)**

If you are worried about your side effects, contact your healthcare provider and let them know about your recent vaccination.

417、**[Is there a link between COVID-19 vaccines and allergic reactions?](https://www.who.int/emergencies/diseases/novel-coronavirus-2019/question-and-answers-hub/q-a-detail/coronavirus-disease-(covid-19)-vaccines-safety)**

In very rare cases, some people may experience an allergic reaction after being vaccinated against COVID-19. A severe allergic reaction – such as anaphylaxis – is a very rare side effect of any vaccine.

If you have a history of allergic reactions, talk to your healthcare provider before you get vaccinated. They will be able to give you advice. In some cases, precautions will need to be taken for people who have known allergies to previous doses of the vaccine or known components of the vaccine.

Healthcare workers administering COVID-19 vaccines should be trained to recognise and treat serious allergic reactions. This is why people being vaccinated against COVID-19 will be asked to stay at the vaccination site for a period of time following their injection, to ensure that anyone experiencing an allergic reaction can receive prompt treatment.

Reports of adverse events following COVID-19 vaccination (including allergic reactions) are closely monitored by national authorities and international bodies, including WHO for the early detection of serious side effects.

418、**[What happens if a serious adverse event is reported after vaccination?](https://www.who.int/emergencies/diseases/novel-coronavirus-2019/question-and-answers-hub/q-a-detail/coronavirus-disease-(covid-19)-vaccines-safety)**

As with any vaccine, it is essential to closely monitor the safety and effectiveness of COVID-19 vaccines that are used in immunization programmes. If a serious health problem is reported following vaccination, a thorough investigation should take place by the public health programme in the country.

It is rare to find that health problems occurring following receipt of a vaccine are actually caused by the vaccine itself. Health problems following vaccination are most often found to be coincidental and entirely unrelated to vaccination. Sometimes they are related to how the vaccine has been stored, transported, or administered. Errors related to the delivery of the vaccine can be prevented by better training health workers and strengthening supply chains.

The results of the investigation will then inform next steps. The safety of COVID-19 vaccines is the top priority of the World Health Organization.

Find out more about [how the safety of vaccines is monitored.](https://www.who.int/news-room/feature-stories/detail/safety-of-covid-19-vaccines)

419、**[Is it safe to mix-and-match different COVID-19 vaccines?](https://www.who.int/emergencies/diseases/novel-coronavirus-2019/question-and-answers-hub/q-a-detail/coronavirus-disease-(covid-19)-vaccines-safety)**

It is safe for you to receive two different COVID-19 vaccines for your first and second dose. This is sometimes called mixing and matching vaccines, or a heterologous vaccine schedule. WHO considers two doses of any WHO EUL COVID-19 vaccines to be a complete primary series. See the full list of COVID-19 vaccines with WHO EUL [here](https://extranet.who.int/pqweb/vaccines/vaccinescovid-19-vaccine-eul-issued).

By mixing and matching vaccines, countries are able to maximise vaccine impact in the event of constrained or limited supply.

People over 60 who have received two doses of Sinovac and Sinopharm can also be given a third dose to help boost their immunity, once a high level of coverage of the priority groups has been achieved. SAGE has stated that Pfizer or AstraZeneca can be used for the third dose if the original vaccine is not available.

Further trials are underway to understand more about mixed doses, which will inform any future changes to WHO’s recommendations.

420、**[Should women who are on their periods take the COVID-19 vaccine?](https://www.who.int/emergencies/diseases/novel-coronavirus-2019/question-and-answers-hub/q-a-detail/coronavirus-disease-(covid-19)-vaccines-safety)**

Women can receive a vaccine at any point in their menstrual cycle.

421、**[Are mRNA vaccines safe? If they’re based on new technology, how can we be sure?](https://www.who.int/emergencies/diseases/novel-coronavirus-2019/question-and-answers-hub/q-a-detail/coronavirus-disease-(covid-19)-vaccines-safety)**

The COVID-19 mRNA vaccine technology has been rigorously assessed for safety, and clinical trials have shown that mRNA vaccines produce an immune response that has high efficacy against disease. mRNA vaccine technology has been studied for several decades, including in the contexts of Zika, rabies, and influenza vaccines. mRNA vaccines are not live virus vaccines and do not interfere with human DNA.

Find out more about the [different types of COVID-19 vaccines](https://www.who.int/news-room/feature-stories/detail/the-race-for-a-covid-19-vaccine-explained) and the [safety of COVID-19 vaccines.](https://www.who.int/news-room/feature-stories/detail/safety-of-covid-19-vaccines)

422、**[Is there a risk of heart inflammation (myocarditis or pericarditis) from mRNA COVID-19 vaccines?](https://www.who.int/emergencies/diseases/novel-coronavirus-2019/question-and-answers-hub/q-a-detail/coronavirus-disease-(covid-19)-vaccines-safety)**

Globally, mRNA vaccines such as Pfizer and Moderna have been used to protect millions of people against COVID-19. A significant amount of data is available from both clinical trials and country surveillance programmes on their efficacy and safety. Some [mild side effects](https://www.who.int/emergencies/diseases/novel-coronavirus-2019/covid-19-vaccines/advice) are expected after vaccination; these are a normal sign that the body is developing protection.

There have been reports of very rare cases of myocarditis (inflammation of the heart muscle) and pericarditis (inflammation of the membrane surrounding the heart) following the second dose of mRNA COVID-19 vaccines. Myocarditis and pericarditis can be caused by many factors, including infections, viruses, medicines and environmental factors. The currently available data suggests that there is also a potential relationship between these symptoms and mRNA vaccines. Research is underway to understand more.

Cases have been mostly reported in younger men aged 12 – 29 (40.6 cases of myocarditis per million second doses) compared to females of the same age group (4.2 cases per million second doses)[1]. 

The symptoms of myocarditis and pericarditis are generally mild. Fast treatment with medication and rest can help to avoid long term heart damage and death. If you experience new and persisting chest pain, shortness of breath or have a racing or pounding heartbeat within a few days of vaccination, contact your doctor immediately. The benefits of these vaccines greatly outweigh the risk of myocarditis and pericarditis by preventing deaths and hospitalisations due to COVID-19.

For more information, please read the [updated guidance](https://www.who.int/news/item/09-07-2021-gacvs-guidance-myocarditis-pericarditis-covid-19-mrna-vaccines) from the COVID-19 subcommittee of the WHO Global Advisory Committee on Vaccine Safety (GACVS).

[Click here to see an infographic on myocarditis and pericarditis.](https://www.who.int/multi-media/details/heart-inflammation-and-mrna-covid-19-vaccines)

423、**[Do the COVID-19 Astra Zeneca and Johnson & Johnson/Janssen vaccines cause blood clots?](https://www.who.int/emergencies/diseases/novel-coronavirus-2019/question-and-answers-hub/q-a-detail/coronavirus-disease-(covid-19)-vaccines-safety)**

Globally, COVID-19 vaccines such as [AstraZeneca](https://www.who.int/news-room/feature-stories/detail/the-oxford-astrazeneca-covid-19-vaccine-what-you-need-to-know) and [Johnson &Johnson/Janssen](https://www.who.int/news-room/feature-stories/detail/the-j-j-covid-19-vaccine-what-you-need-to-know) have been used to protect millions of people. Data is available from both clinical trials and preliminary data from country surveillance programmes on their efficacy and safety. Some [mild to moderate side effects](https://www.who.int/news-room/feature-stories/detail/side-effects-of-covid-19-vaccines) such as fever, muscle and head aches, soreness around the injection site and tiredness are expected to affect some people after vaccination. These are a normal indications that the body is developing protection.

There have been reports of very rare but serious cases of blood clots accompanied by low platelet counts (known as thrombosis with thrombocytopenia syndrome (TTS)) occurring 3 to 30 days after vaccination with COVID-19 non-replicant adenovirus vector-based vaccines (such as the AstraZeneca and Janssen vaccines). Read more about the [different types of COVID-19 vaccines](https://www.who.int/news-room/feature-stories/detail/the-race-for-a-covid-19-vaccine-explained).

With the AstraZeneca vaccine, as of 15 July 2021, the data shows that these symptoms occur in about four to six people out of every million vaccinated (this figure varies based on age, sex and geographical location)[2}. Younger adults appear to be at higher risk than older adults. More research is underway to understand more about how people may be differently affected.

With the Janssen vaccine, as of the 7 May 2021, the US Food and Drug Administration and the Centers for Disease Control and Prevention had reviewed 28 reports of TTS out of a total of more than eight million people vaccinated[3]. It is possible that that there a causal link between the vaccine and these symptoms, but more data is needed.

TTS is very rare; however, blood clotting is a common health problem caused by many factors. Not all clots that occur after vaccination with Astra Zeneca or the Janssen vaccine will be due to TTS. The risk of blood clots is far higher from COVID-19 itself than from either vaccine.

If you experience a new, severe, persistent headache, blurred vision, chest pain, severe abdominal pain, leg swelling or unusual skin bruising and shortness of breath between three to 30 days following vaccination, contact your doctor immediately. WHO has published [interim guidance for clinical case management of TTS following vaccination](https://apps.who.int/iris/bitstream/handle/10665/342999/WHO-2019-nCoV-TTS-2021.1-eng.pdf).

The benefits of the COVID-19 Astra Zeneca and Janssen vaccines are far greater than the very small risk of TTS. As well as protecting you from severe disease and death due to COVID-19, being vaccinated can protect you against complications from ‘long COVID’, provide some protection for your close contacts and community by reducing transmission, and can reduce the risk of severe disease from some variant strains. WHO recommends that these vaccines continue to be used to protect priority groups.

*For more information, read the Statement of the COVID-19 subcommittee of the WHO Global Advisory Committee on Vaccine Safety (GACVS) on* [*safety signals related to the Johnson & Johnson/Janssen COVID-19 vaccine*](https://www.who.int/news/item/19-05-2021-statement-gacvs-safety-johnson-johnson-janssen-covid-19-vaccine) *and the GACVS review of* [*latest evidence of rare adverse blood coagulation events with AstraZeneca COVID-19 Vaccine*](https://www.who.int/news/item/16-04-2021-global-advisory-committee-on-vaccine-safety-(gacvs)-review-of-latest-evidence-of-rare-adverse-blood-coagulation-events-with-astrazeneca-covid-19-vaccine-(vaxzevria-and-covishield))*.*

424、**[Do the AstraZeneca and Johnson & Johnson/Janssen COVID-19 vaccines cause Guillain-Barré syndrome?](https://www.who.int/emergencies/diseases/novel-coronavirus-2019/question-and-answers-hub/q-a-detail/coronavirus-disease-(covid-19)-vaccines-safety)**

Globally, vaccines such as [AstraZeneca](https://www.who.int/news-room/feature-stories/detail/the-oxford-astrazeneca-covid-19-vaccine-what-you-need-to-know) and [Johnson &Johnson/Janssen](https://www.who.int/news-room/feature-stories/detail/the-j-j-covid-19-vaccine-what-you-need-to-know)have been used to protect millions of people against COVID-19. Data is available from both clinical trials and preliminary data from country surveillance programmes on their efficacy and safety. Some [mild to moderate side effects](https://www.who.int/news-room/feature-stories/detail/side-effects-of-covid-19-vaccines) such as fever, muscle and head aches, soreness around the injection site and tiredness are expected to affect some people after vaccination. These are a normal indications that the body is developing protection.

There have been very rare reports of cases of Guillain-Barré syndrome occurring within 42 days of vaccination with the AstraZeneca and Janssen vaccines (adenovirus vector vaccines). [Guillain-Barré syndrome](https://www.who.int/news-room/fact-sheets/detail/guillain-barr%C3%A9-syndrome) is a rare condition in which a person’s immune system attacks the nerves, causing muscle weakness, tingling and/or loss of sensation in the arms and/or legs. Most people affected recover fully. Although these cases happened after vaccination, it is not yet possible to tell whether they were related to or caused by the vaccines, or if they were coincidental. The benefits of vaccination against COVID-19 greatly outweigh any possible risk.

As of the 27 June, the European Medicines Agency had received reports of 227 cases of Guillain-Barré syndrome in the European Union following vaccination with AstraZeneca. Around 51.4 million doses of Vaxzevria (the brand of AstraZeneca produced in Europe) had been administered within the European Union by 20 June 2021. For the Janssen vaccine, the US Advisory Committee on Immunization Practices reported that 100 cases of Guillain-Barré syndrome had been reported through the Vaccine Adverse Events Reporting System in the United States of America (USA) as of 30 June, with approximately 12.2 million doses of the Janssen vaccine administered. Outside of the European Union and the USA, cases of Guillain-Barré syndrome have also been reported; however further investigation is needed to determine whether the rate has increased from usual levels.

Guillain-Barré syndrome has many causes, such as bacterial or viral infections, surgery or vaccine administration. It can also be caused by COVID-19. In 2011, it was estimated that the incidence of Guillain-Barré syndrome in Europe and Northern America was 0.8 to 1.9 cases out of every 100 000 people [[4]](https://www.who.int/emergencies/diseases/novel-coronavirus-2019/question-and-answers-hub/q-a-detail/coronavirus-disease-(covid-19)-vaccines-safety#_ftn1). It can affect people of all ages but is more common in adults and in males. Most people recover fully even from the most severe cases of Guillain-Barré syndrome; however, it is potentially life threatening and some people affected may need intensive care.

If you experience any of the following symptoms within 42 days of vaccination, contact your healthcare provider immediately:

- Difficulty walking
- Difficulty with facial movements, including breathing, speaking, chewing or swallowing
- Double vision or being unable to move your eyes
- Difficulty with bladder or bowel control
- Weakness or tingling sensations in the legs, arms and face

The benefits of the COVID-19 Janssen and AstraZeneca vaccines are far greater than the very small risk of Guillain-Barré syndrome. As well as protecting you from severe disease and death due to COVID-19, being vaccinated can protect you against complications from ‘long COVID’, provide some protection for your close contacts and community by reducing transmission, and can reduce the risk of severe disease from variant strains. COVID-19 disease can also cause Guillain-Barré syndrome. WHO recommends that these vaccines continue to be used to protect priority groups.

*For more information, read the* [Statement of the COVID-19 subcommittee of the WHO Global Advisory Committee on Vaccine Safety (GACVS) COVID-19 subcommittee on reports of Guillain-Barré](https://www.who.int/news/item/26-07-2021-statement-of-the-who-gacvs-covid-19-subcommittee-on-gbs)*.*

#### [Coronavirus disease (COVID-19): Variants of SARS-COV-2（425-435）](https://www.who.int/emergencies/diseases/novel-coronavirus-2019/question-and-answers-hub/q-a-detail/coronavirus-disease-(covid-19)-variants-of-sars-cov-2)

425、**[What are variants of SARS-COV-2, the virus that causes COVID-19?](https://www.who.int/emergencies/diseases/novel-coronavirus-2019/question-and-answers-hub/q-a-detail/coronavirus-disease-(covid-19)-variants-of-sars-cov-2)**

It is normal for viruses to change and evolve as they spread between people over time. When these changes become significantly different from the original virus, they are known as “variants.” To identify variants, scientists map the genetic material of viruses (known as sequencing) and then look for differences between them to see if they have changed. 

Since the SARS-CoV-2 virus, the virus that causes COVID-19, has been spreading globally, variants have emerged and been identified in many countries around the world.

426、**[What is the difference between a variant of interest and a variant of concern?](https://www.who.int/emergencies/diseases/novel-coronavirus-2019/question-and-answers-hub/q-a-detail/coronavirus-disease-(covid-19)-variants-of-sars-cov-2)**

A variant is considered a variant of interest if it has mutations that are suspected or known to cause significant changes, and is circulating widely (e.g., known to cause many clusters of infected people, or found in many countries). There are many variants of interest that WHO is continuing to monitor in case they become variants of concern. 

A variant of interest becomes a variant of concern if it is known to spread more easily, cause more severe disease, escape the body’s immune response, change clinical presentation, or decrease effectiveness of known tools – such as public health measures, diagnostics, treatments and vaccines.

427、**[How do variants of SARS-CoV-2, the virus that causes COVID-19, get their names?](https://www.who.int/emergencies/diseases/novel-coronavirus-2019/question-and-answers-hub/q-a-detail/coronavirus-disease-(covid-19)-variants-of-sars-cov-2)**

For the purpose of discussing variants in the media and the public, [WHO began using the Greek alphabet in May of 2021](https://www.who.int/news/item/31-05-2021-who-announces-simple-easy-to-say-labels-for-sars-cov-2-variants-of-interest-and-concern) to make it easier for people to keep track of variants without linking their names to the places where they were first identified, as variants can emerge anywhere at any time.

In accordance with WHO best practices for naming new diseases, some letters may not be used if they cause confusion in major languages or stigmatize certain groups. These WHO labels do not replace the existing scientific names of the variants, which convey important scientific information to researchers and scientists.

428、**[What is the Omicron variant?](https://www.who.int/emergencies/diseases/novel-coronavirus-2019/question-and-answers-hub/q-a-detail/coronavirus-disease-(covid-19)-variants-of-sars-cov-2)**

The Omicron variant, variant B.1.1.529, was first reported to WHO on 24 November 2021 and was [classified as a variant of concern by WHO on 26 November 2021](https://www.who.int/news/item/26-11-2021-classification-of-omicron-(b.1.1.529)-sars-cov-2-variant-of-concern). The classification was made on the advice of the Technical Advisory Group on Virus Evolution, based primarily on information from South Africa that the variant has a large number of mutations and has caused a detrimental change in COVID-19 epidemiology.

429、**[What makes the Omicron variant different from other variants?](https://www.who.int/emergencies/diseases/novel-coronavirus-2019/question-and-answers-hub/q-a-detail/coronavirus-disease-(covid-19)-variants-of-sars-cov-2)**

All variants are different. The Omicron variant has a large number of mutations which may mean the virus acts differently from other variants that are circulating.

As of 1 December 2021, there is limited information about Omicron.  Studies are ongoing to determine if there is a change in how easily the virus spreads or the severity of disease it causes, and if there are any impacts on protective measures.

It will likely take time before there will be clear evidence to determine if there is any change in the transmission of Omicron compared to other variants, how the variant responds to existing therapeutics, or whether infection or re-infection with Omicron causes more or less severe disease.

WHO is working with technical partners to understand the potential impact of Omicron on vaccine effectiveness. Currently, the Delta variant is dominant worldwide and COVID-19 vaccines are highly effective at protecting you from serious illness and death, including from infection with Delta. Researchers will assess the performance of current vaccines against Omicron and will communicate these findings as soon as they become available.

See the [announcement](https://www.who.int/news/item/26-11-2021-classification-of-omicron-(b.1.1.529)-sars-cov-2-variant-of-concern) and [short video](https://www.youtube.com/watch?v=Pl8RWEFDUT4) on the classification of the Omicron variant.

430、**[What is being done to understand more about the Omicron variant?](https://www.who.int/emergencies/diseases/novel-coronavirus-2019/question-and-answers-hub/q-a-detail/coronavirus-disease-(covid-19)-variants-of-sars-cov-2)**

WHO continues to coordinate with a large number of researchers around the world to understand more about all variants of SARS-CoV-2, the virus that causes COVID-19, including Omicron. Many studies are needed, including assessments of:

- Transmissibility, or ease of spread from person to person, of Omicron as compared to other variants
- Severity of infection and re-infection with Omicron
- Performance of current COVID-19 vaccines against Omicron
- Performance of diagnostic tests, including antigen tests, to detect infection with Omicron
- Effectiveness of current treatments for management of patients with COVID-19 disease

WHO’s Technical Advisory Group on Virus Evolution will continue to monitor and evaluate the data as it becomes available and assess if mutations in the Omicron variant alter the behaviour of the virus.

431、 **[What is the Delta variant?](https://www.who.int/emergencies/diseases/novel-coronavirus-2019/question-and-answers-hub/q-a-detail/coronavirus-disease-(covid-19)-variants-of-sars-cov-2)**

The Delta variant is a variant of concern classified by WHO on May 11, 2021 and is currently the dominant variant that is circulating globally.  Delta spreads more easily than earlier strains of the virus and is responsible for more cases and deaths worldwide.  All approved COVID-19 vaccines currently in use are safe and effective in preventing severe disease and death against the Delta variant.

432、**[What can I do to protect myself from variants?](https://www.who.int/emergencies/diseases/novel-coronavirus-2019/question-and-answers-hub/q-a-detail/coronavirus-disease-(covid-19)-variants-of-sars-cov-2)**

To protect yourself and others from COVID-19 variants:

- Keep a distance of at least 1 metre from others
- Wear a well-fitted mask over your mouth and nose
- Open windows
- Cough or sneeze into a bent elbow or tissue
- Clean your hands frequently
- Get vaccinated, as soon as it is your turn

433、**[How can we stop new variants from emerging?](https://www.who.int/emergencies/diseases/novel-coronavirus-2019/question-and-answers-hub/q-a-detail/coronavirus-disease-(covid-19)-variants-of-sars-cov-2)**

As with all viruses, SARS-COV-2, the virus that causes COVID-19, will continue to evolve as long as it continues to spread. The more that the virus spreads, the more pressure there is for the virus to change. So, the best way to prevent more variants from emerging is to stop the spread of the virus.

To protect yourself and others from COVID-19:

- Keep a distance of at least 1 metre from others
- Wear a well-fitted mask over your mouth and nose
- Open windows
- Cough or sneeze into a bent elbow or tissue
- Clean your hands frequently
- Get vaccinated as soon as it is your turn

434、**[re the variants more likely to cause infection, disease and death in people who are already vaccinated?](https://www.who.int/emergencies/diseases/novel-coronavirus-2019/question-and-answers-hub/q-a-detail/coronavirus-disease-(covid-19)-variants-of-sars-cov-2)**

We are still learning about the ways that variants impact vaccination.

The data we currently have available show us that COVID-19 vaccines are still very effective at preventing serious illness and death against all of the current variants of concern. It is important to note that the vaccines provide different levels of protection from infection, mild disease, severe disease, hospitalization and death.

No vaccine is 100% effective. Even though COVID-19 vaccines are highly effective at protecting you against serious illness and death, some people will still get ill from COVID-19 after vaccination. You could also pass the virus on to others who are not vaccinated. This makes it very important to continue to practice protective measures, even after you have been fully vaccinated.

It is more important than ever to get vaccinated as soon as it is your turn and continue to practice protective measures after vaccination.

435、**[What is the difference between a ‘mutation’ and a ‘variant’?](https://www.who.int/emergencies/diseases/novel-coronavirus-2019/question-and-answers-hub/q-a-detail/coronavirus-disease-(covid-19)-variants-of-sars-cov-2)**

Viruses are constantly evolving and changing. Every time a virus replicates (makes copies of itself), there is the potential for there to be changes in its structure. Each of these changes is a “mutation.” A virus with one or more mutations is called a “variant” of the original virus.

Some mutations can lead to changes in important characteristics of the virus, including characteristics that affect its ability to spread and/or its ability to cause more severe illness and death.

#### [Coronavirus disease (COVID-19): Ventilation and air conditioning（436-447）](https://www.who.int/emergencies/diseases/novel-coronavirus-2019/question-and-answers-hub/q-a-detail/coronavirus-disease-covid-19-ventilation-and-air-conditioning)

436、**[What is ventilation and how can it prevent COVID-19 from spreading?](https://www.who.int/emergencies/diseases/novel-coronavirus-2019/question-and-answers-hub/q-a-detail/coronavirus-disease-covid-19-ventilation-and-air-conditioning)**

Ventilation is the process of bringing fresh, outdoor air inside and letting indoor air outside in order to maintain or improve air quality.

The risk of getting COVID-19 infection is increased in crowded and poorly ventilated settings. This is because the virus passes between people through infected respiratory particles in the form of droplets and aerosols. In poorly ventilated spaces infected aerosols can remain suspended in the air or travel farther  than conversational distance. Improving indoor ventilation reduces the risk of the virus spreading indoors. For practical advice, please see our infographic.

<https://cdn.who.int/media/images/default-source/health-topics/coronavirus/ventilation_infographic_03_12.jpg?sfvrsn=78a27e26_5>

[***Download infographic***](https://cdn.who.int/media/images/default-source/health-topics/coronavirus/ventilation_infographic_03_128de6f570-5579-4aea-876c-000d80deb3f1.jpg?sfvrsn=b800173e_5)

Ventilation is not a standalone measure and it should be implemented as part of a comprehensive package of measures, such as physical distancing, avoiding crowds, wearing a mask, frequent hand cleaning, staying home if unwell, coughing or sneezing into a bent elbow, and vaccination. Each of these is important to protect you against COVID-19 infection. *Find out more about how to stay safe on our*  [public advice page](https://www.who.int/emergencies/diseases/novel-coronavirus-2019/advice-for-public).

437、**[How do I improve ventilation in my home or workplace?](https://www.who.int/emergencies/diseases/novel-coronavirus-2019/question-and-answers-hub/q-a-detail/coronavirus-disease-covid-19-ventilation-and-air-conditioning)**

When you are inside, open windows or doors whenever possible. For better ventilation, open windows/doors on opposite sides of a room to create a cross breeze. If creating a cross breeze is not possible, you can place a fan in front of an open window to increase air flow and push indoor air outside.

If the temperature outside is extremely hot or cold, you can open windows for a few minutes every hour to bring in fresh air.

438、**[Will an air conditioner provide good ventilation?](https://www.who.int/emergencies/diseases/novel-coronavirus-2019/question-and-answers-hub/q-a-detail/coronavirus-disease-covid-19-ventilation-and-air-conditioning)**

The majority of wall or window unit air conditioning systems do not provide ventilation. They are designed to reduce the temperature and humidity of the air, and they do this by recirculating indoor air. Whenever using a wall or window unit air conditioning system, open windows for a few minutes every hour to bring in fresh air from the outside.

Heating, ventilation and air-conditioning (HVAC) systems pull outside air inside. Make sure the settings on your HVAC system maximize the amount of fresh, outdoor air that is pulled into the system. HVAC systems should always be regularly inspected, maintained and cleaned according to the manufacturer’s recommendations.

If you are riding in a vehicle that has air conditioning, make sure to use the setting that bring in fresh air.

439、**[How can I improve heating and air conditioning systems at home?](https://www.who.int/emergencies/diseases/novel-coronavirus-2019/question-and-answers-hub/q-a-detail/coronavirus-disease-covid-19-ventilation-and-air-conditioning)**

Non-ducted heating and air conditioning units that recirculate air, such as fan-coil or split units, should be assessed, maintained and cleaned according to manufacturer recommendations. Assess the unit’s filter and consider replacing the existing filter with a MERV14/ISO ePM1 70-80% air filter or the highest compatible filter with the filter rack, in collaboration with an HVAC professional. The units and filters should be periodically cleaned and maintained according to manufacturer recommendations.

More information is available  [here.](https://www.who.int/publications-detail-redirect/9789240021280)

440、**[Can fans be used safely inside?](https://www.who.int/emergencies/diseases/novel-coronavirus-2019/question-and-answers-hub/q-a-detail/coronavirus-disease-covid-19-ventilation-and-air-conditioning)**

Using a fan in an enclosed space can increase the spread of the virus that causes COVID-19. This is why it is important to open windows and doors whenever using a fan to replace indoor air without outdoor air.

If using a pedestal fan, minimize as much as possible how much air blows from one person (or group of people) to another person (or group of people). You can place a fan in front of an open window to increase air flow and push indoor air outside.

The use of ceiling fans can improve the circulation of air from outside and avoid pockets of stagnant air forming indoors. However, it is important to bring in air from the outside by opening windows when using a ceiling fan

441、**[How can I improve ventilation at home if someone is sick with the virus that causes COVID-19?](https://www.who.int/emergencies/diseases/novel-coronavirus-2019/question-and-answers-hub/q-a-detail/coronavirus-disease-covid-19-ventilation-and-air-conditioning)**

If someone is sick and being cared for at home, the following can be done to improve ventilation and reduce the risk of infection to other members of the household:

- Whenever possible, the ill person should stay in a separate room. If this is not possible, then keep at least a 1-metre distance from them. The sick person and anyone else in the same room should wear a medical mask.
- Provide good ventilation in the room of the ill person and shared spaces, and open windows whenever possible.
- Create a cross breeze by opening windows or doors on opposite sides of the ill person’s room.
- If possible, try to prevent the air from moving from the sick person’s room to the rest of the house.
- If available, the ill person should stay in a separate room that has a private toilet with an air extractor or exhaust fan, which should run at a high speed.
- As a supplement, you can use a stand-alone air cleaner with a MERV14/ISO ePM1 70-80% air filter. MERV14/F8 filter. These devices may improve air quality but are not a replacement for ventilation.

Also see  [WHO’s Q&A on homecare .](https://www.who.int/news-room/questions-and-answers/item/coronavirus-disease-covid-19-home-care-for-families-and-caregivers)

442、**[Is it safe for me to have visitors in my home if I have good ventilation?](https://www.who.int/emergencies/diseases/novel-coronavirus-2019/question-and-answers-hub/q-a-detail/coronavirus-disease-covid-19-ventilation-and-air-conditioning)**

In the context of the COVID-19 pandemic, there is no “zero risk” when it comes to gathering with others, but there are many ways in which you can reduce the risk of infection and spread of the COVID-19 virus.

In general, you are at risk of COVID-19 infection whenever you get together with people and risk reduction measures are not in place.  If you meet with others who live outside of your household, WHO recommends to meet outdoors whenever possible. Outdoor venues are safer than indoor venues because there is more air flow and fresh air.

If you have visitors in your home, always maintain a physical distance of at least 1 metre, wear a mask, and open windows to improve air flow. To enhance ventilation, create a cross breeze or place a fan in front of an open window facing the outside. For more details, see “How do I improve ventilation in my home or workplace?” above.

443、**[Do portable air filters provide ventilation?](https://www.who.int/emergencies/diseases/novel-coronavirus-2019/question-and-answers-hub/q-a-detail/coronavirus-disease-covid-19-ventilation-and-air-conditioning)**

Air filters do not provide ventilation and do not replace other ventilation methods. However, they can help to reduce the concentration of the COVID-19 virus in the air, thus reducing the possibility of transmission. A MERV14/ISO ePM1 70-80% air filter can improve air quality when used in indoor settings.

444、**[Does mosquito screening impact ventilation?](https://www.who.int/emergencies/diseases/novel-coronavirus-2019/question-and-answers-hub/q-a-detail/coronavirus-disease-covid-19-ventilation-and-air-conditioning)**

Simple mosquito screening on doors and windows is important for protecting yourself against the diseases spread by mosquitoes and other insects.  The use of such screens may reduce the natural ventilation rate and it is recommended where screens are used that more screened windows are opened to create cross ventilation.

445、**[How can I improve ventilation in a car or vehicle?](https://www.who.int/emergencies/diseases/novel-coronavirus-2019/question-and-answers-hub/q-a-detail/coronavirus-disease-covid-19-ventilation-and-air-conditioning)**

When riding in a car or vehicle open windows whenever possible, the more windows the better. If you are using air conditioning in the vehicle, use the setting that brings in fresh air.

446、**[How can ventilation reduce the risk of contracting COVID-19 in airplanes?](https://www.who.int/emergencies/diseases/novel-coronavirus-2019/question-and-answers-hub/q-a-detail/coronavirus-disease-covid-19-ventilation-and-air-conditioning)**

Most airplanes have cabin air filtration systems equipped with HEPA filters which can remove viruses and germs quickly, reducing risk exposure to any potential infectious virus or bacteria expelled by a cough or sneeze. Cabin air systems are designed to operate most efficiently by delivering approximately 50 percent outside air and 50 percent filtered, recirculated air. The air supply is essentially sterile and particle-free.

However, adequate ventilation is just one of the preventive measures to reduce the risk of COVID-19 transmission.  Other important measures include maintaining physical distance of at least 1 metre, wearing a mask, cleaning hands frequently, and sneezing/coughing into a bent elbow or tissue, and getting vaccinated when you can.

447、**[What is WHO doing to address ventilation in the context of COVID-19?](https://www.who.int/emergencies/diseases/novel-coronavirus-2019/question-and-answers-hub/q-a-detail/coronavirus-disease-covid-19-ventilation-and-air-conditioning)**

Since January 2020, WHO has provided recommendation on ventilation in COVID-19 guidance. Early in the pandemic, WHO established the WHO Environment and Engineering Control Expert Advisory Panel (ECAP) for COVID-19 to review available scientific evidence and practices and update recommendations on environment and engineering controls. This multidisciplinary network brings together technical experts from various fields, such as infection control specialists, engineers, architects, aerobiologists and environmental experts.

In June 2020, WHO contributed to guidance on ventilation and air-conditioning systems in the context of COVID-19, available  [here.](http://www.ghhin.org/heat-and-covid-19/ac-and-ventilation) WHO works closely with the World Meteorological Organization Joint Office for Climate and Health and the United States National Oceanic and Atmospheric Administration (NOAA) through the Global Health Information Network to develop and update this guidance. Throughout 2020 and 2021, WHO experts worked to enhance ventilation guidance in a number of technical guidance products for different settings including health facilities, homes, quarantine facilities, schools and businesses. In addition, in March 2021, WHO published a  [roadmap](https://www.who.int/publications-detail-redirect/9789240021280) to improve ventilation in various settings.

#### [Coronavirus disease (COVID-19): Ventilation and air conditioning in public spaces and buildings（448X](https://www.who.int/emergencies/diseases/novel-coronavirus-2019/question-and-answers-hub/q-a-detail/coronavirus-disease-covid-19-ventilation-and-air-conditioning-in-public-spaces-and-buildings)

448、For advice on ventilation, please see the [*ventilation roadmap.*](https://www.who.int/publications-detail-redirect/9789240021280) This roadmap replaces the July 2020 WHO Q&A on the same topic.

#### [Coronavirus disease (COVID-19): Violence against women（448-460）](https://www.who.int/emergencies/diseases/novel-coronavirus-2019/question-and-answers-hub/q-a-detail/coronavirus-disease-covid-19-violence-against-women)

448、**[Home is not a safe place for me. What can I do?](https://www.who.int/emergencies/diseases/novel-coronavirus-2019/question-and-answers-hub/q-a-detail/coronavirus-disease-covid-19-violence-against-women)**

If you are experiencing violence, it may be helpful to reach out to family, friends and neighbors, to seek support from a hotline or, if safe, from online service for survivors of violence. Find out if local services (e.g. shelters, counselling) are open and reach out to them if available.

Make a safety plan in case the violence against you or your children escalates. This includes:

- Identifying a neighbour, friend, relative, colleague, or shelter to go to in case you need to leave the house immediately for safety.
- Have a plan for how you will exit the house safely and how you will reach there (e.g. transport).
- Keep a few essential items (e.g. identification documents, phone, money, medicines, and clothes) available, and a list of telephone numbers in case of an emergency.
- If possible, develop a code with a trusted neighbour so they can come to your aid in case of an emergency.

449、**[I need medical attention because of violence. Who can help me and where should I go?](https://www.who.int/emergencies/diseases/novel-coronavirus-2019/question-and-answers-hub/q-a-detail/coronavirus-disease-covid-19-violence-against-women)**

If you need urgent medical attention, call for an ambulance or contact your country’s emergency health services. If you need any other support, contact a specialized service if available or a health provider.

450、**[I am safe, but I am suffering from mental / sexual / social / long-term physical health problems because of violence. Is there anyone who can help me during COVID-19?](https://www.who.int/emergencies/diseases/novel-coronavirus-2019/question-and-answers-hub/q-a-detail/coronavirus-disease-covid-19-violence-against-women)**

As much as possible, reduce sources of stress:

- Maintain contact with and seek support from family and friends via phone, email, text, etc.
- Try to maintain daily routines and make time for physical activity and sleep.
- Use relaxation exercises (e.g. slow breathing, meditation, progressive muscle relaxation, grounding exercises – [see WHO Clinical handbook p.70](https://apps.who.int/iris/bitstream/handle/10665/136101/WHO_RHR_14.26_eng.pdf;jsessionid=D1A793C54703C44D28E2E95590DBBF36?sequence=1)) to relieve stressful thoughts and feelings.
- Engage in activities that in the past have helped with managing adversity.
- Seek information about COVID- 19 from reliable sources and reduce the time spent consuming news (for example, 1-2 times per day, rather than every hour).

Seek care from a trained health provider for symptoms and conditions including injuries that need medical attention. Due to restrictions on movement, and pressure on health systems, it may be difficult to access healthcare in person at this time. In this case, find out what is available in your locality and seek alternatives, including information and support offered by phone or online.

If you are currently receiving healthcare and support for violence-related issues this could continue during COVID-19 – this could even be by phone or online if feasible and appropriate for your health needs.

451、**[I am worried about someone I know. How can I help?](https://www.who.int/emergencies/diseases/novel-coronavirus-2019/question-and-answers-hub/q-a-detail/coronavirus-disease-covid-19-violence-against-women)**

If you know of, or are concerned about, someone in an abusive relationship there are some things you can do:

- Keep regularly in touch with the person to check that they are safe, ensuring that it is safe for them to be in touch with you. Assume that a perpetrator of violence can see, hear and or monitor communications, so find out how best to communicate with the person that you are concerned about.  Send them an SMS or message via email or social media, in a way that is safe for them. Be discrete in connecting with them when the abuser is present in the home so that they are not placed at risk of additional harm. Check each time, as this may change.
- Find out what services for survivors of violence against women (e.g. shelters, hotlines, counselling services, women’s organizations) are functioning during the COVID-19 pandemic and make this information available through your networks and social media. Only provide this information directly if you can do so discretely without the abuser finding out.
- If someone you know needs urgent help for whatever reason, be prepared to call emergency health services, the police, health centre, or hotline.

452、**[I want to report an incidence of violence during COVID-19. What should I do?](https://www.who.int/emergencies/diseases/novel-coronavirus-2019/question-and-answers-hub/q-a-detail/coronavirus-disease-covid-19-violence-against-women)**

If you want to report an incidence of violence, you should follow your country’s guidance on how to report violence and contact the service where you are most likely to get a survivor-centred response.

Some countries are making exceptions to movement restrictions for reporting of violence specifically. Remember that reporting may put you at risk, so ensure you have a safety plan in place.

You may find making a report mentally or emotionally difficult. Seek the support of friends, relations, neighbours or colleagues, or local support services for survivors of violence.

453、**[I have harmed or am worried about harming or hurting my partner (and children) with my words or actions. How can I stop?](https://www.who.int/emergencies/diseases/novel-coronavirus-2019/question-and-answers-hub/q-a-detail/coronavirus-disease-covid-19-violence-against-women)**

- If you feel yourself getting angry or very annoyed, step away into another room if you can, or outside for a deep breath.
- Count to ten and breathe in and out until you feel calmer. Count down from 10, or do anything else that may help you stay calm.
- Talk to a trusted friend, relative, or religious leader and if necessary seek help from local health services or specialized services if available.
- Recognize that everyone in your family is experiencing stress during this time.
- Demonstrate kindness and patience in your words and actions.
- Eliminate or reduce your alcohol consumption as much as possible.

The current measures to address the COVID-19 pandemic, such as restrictions on movement, as well as financial instability caused by the crisis, are likely to add to your stress.  This is normal, and you can control how you react.  Take measures to ensure you manage your stress in a way that is respectful and safe to you and your family. Try to be kind to yourself, your partner, children and anyone else in the family.

454、**[I am a health worker. How can I help women survivors of violence during COVID-19?](https://www.who.int/emergencies/diseases/novel-coronavirus-2019/question-and-answers-hub/q-a-detail/coronavirus-disease-covid-19-violence-against-women)**

As a health worker, there are things you can do to help mitigate the impacts of violence on women and children at any time, including during the COVID-19 pandemic.

Whilst your time and resources may be stretched during this health emergency, you have a duty of care to women who may seek help from you – often to address physical and mental hurt caused by violence.

You may need to adjust how you deliver services, if face-to-face care is not possible. Depending on your resources consider mobile phone, WhatsApp or other communication channels to deliver support, always ensuring you can do this in a way that is safe for the survivor. Communicate openly with women about any changes in service delivery.

If a woman with suspected or confirmed COVID-19 seeks your care because of violence, your response should be the same as for any other survivor. You should however ensure that you are protected from infection, by maintaining distance where possible, wearing protective clothing, practicing respiratory hygiene, and washing hands and surfaces with soap and water regularly and after touching, to avoid infection.

In any circumstances, including during the COVID-19 pandemic, health workers should provide first-line support, using the LIVES approach to help women survivors of violence:

**L**isten closely, with empathy and no judgement

**I**nquire about women’s needs and concerns

**V**alidate women’s experiences. Show you believe and understand.

**E**nhance their safety.

**S**upport women to connect with additional services.

455、**[I run a health facility. What can I do to support women survivors of violence during COVID-19?](https://www.who.int/emergencies/diseases/novel-coronavirus-2019/question-and-answers-hub/q-a-detail/coronavirus-disease-covid-19-violence-against-women)**

- Identify information on local services for survivors, including on hotlines, shelters, rape crisis centers, and counselling.
- Find out opening hours, contact details and whether these services can be offered remotely, and establish referral linkages.
- Make these available to health care providers and easily accessible to patients/clients coming to your facility.
- Support the health care providers in your facility to continue to support women survivors of violence during this time.
- Be prepared to shift rapidly to providing care through alternative ways, by creating effective response plans; for example, by ensuring frontline healthcare workers have phones and can charge them.
- Ensure there is a protocol in place to ensure continued safe document storage in case of sudden lockdown.
- Inform survivors of ways they can protect themselves, and provide small credit card-size cards listing relevant phone numbers for support

456、**[I am a policy maker. What can I do to prevent and address violence against women during COVID-19?](https://www.who.int/emergencies/diseases/novel-coronavirus-2019/question-and-answers-hub/q-a-detail/coronavirus-disease-covid-19-violence-against-women)**

- When making preparedness and response plans for the COVID-19 pandemic, include essential services for violence against women.
- Allocate adequate resources for services for survivors and identify ways to make them accessible – particularly in the context of measures to restrict people’s movement.
- Explore the use of telemedicine, digital health, or mhealth to enhance access to support and services for survivors, while ensuring this is safe for them.
- Explore alternative shelters for women who may require this.
- Ensure that lockdown measures do not penalize women for seeking support when experiencing violence, such as by going to the police or hospital to report violence.

457、**[Has violence against women increased since the beginning of the COVID-19 pandemic?](https://www.who.int/emergencies/diseases/novel-coronavirus-2019/question-and-answers-hub/q-a-detail/coronavirus-disease-covid-19-violence-against-women)**

Violence against women is highly prevalent, and intimate partner violence is the most common form of violence. During health emergencies, such as the COVID-19 pandemic, violence against women tends to increase.

Whilst data are scarce, reports from across the world, including China, the United Kingdom, the United States of America, and other countries suggest a significant increase in domestic violence cases related to the COVID-19 pandemic. Reports from other countries suggest a reduction in survivors seeking services due to a combination of lockdown measures and not wanting to attend health services for fear of infection.

458、**[How does COVID-19 increase risks of violence for women?](https://www.who.int/emergencies/diseases/novel-coronavirus-2019/question-and-answers-hub/q-a-detail/coronavirus-disease-covid-19-violence-against-women)**

Stress, the disruption of social and protective networks, loss of income and decreased access to services all can exacerbate the risk of violence for women.

In many countries, where people are encouraged or required to stay at home, the risk of intimate partner violence is likely to increase. Please see the following resource for more detail: <https://www.who.int/reproductivehealth/publications/emergencies/COVID-19-VAW-full-text.pdf>

In addition, access to sexual and reproductive health services will likely become more limited. Other services, such as hotlines, crisis centers, shelters, legal aid, and protection services, may also be reduced, making it difficult for women to access the few sources of help that would usually be available.

459、**[Who is most vulnerable?](https://www.who.int/emergencies/diseases/novel-coronavirus-2019/question-and-answers-hub/q-a-detail/coronavirus-disease-covid-19-violence-against-women)**

Women who are displaced, who are migrants or refugees, and those living in conflict-affected areas, older women and women with disabilities are particularly at risk of violence and are likely to be disproportionately affected by violence during COVID-19.

460、**[Why should I care about violence against women during COVID-19?](https://www.who.int/emergencies/diseases/novel-coronavirus-2019/question-and-answers-hub/q-a-detail/coronavirus-disease-covid-19-violence-against-women)**

Violence against women is a grave violation of women’s human rights, no matter when, where, or how it takes place. There are many forms of violence against women, which have many potential negative health consequences for women and their children.

Violence against women can result in injuries and serious physical, mental, sexual and reproductive health problems, including sexually transmitted infections, HIV, and unplanned pregnancies. In extreme cases, violence against women can result in death.

The effects of violence are very often long-lived. Violence – in all its forms – can have an impact on a woman’s well-being throughout the rest of her life. This is unacceptable, and the risks of violence that women and their children face during the current COVID-19 crisis cannot be ignored.

When we are able to prevent violence, or to support women survivors of violence, we help to safeguard women’s human rights, and promote physical and mental health and well-being for women throughout their lives. This also helps to alleviate pressure on already stretched essential public services, including the health system.

#### [Coronavirus disease (COVID-19): Virus Evolution（461-464）](https://www.who.int/emergencies/diseases/novel-coronavirus-2019/question-and-answers-hub/q-a-detail/sars-cov-2-evolution)

461、**[What does it mean to say a virus mutates or changes?](https://www.who.int/emergencies/diseases/novel-coronavirus-2019/question-and-answers-hub/q-a-detail/sars-cov-2-evolution)**

When a virus replicates or makes copies of itself, it sometimes changes a little bit. These changes are called “mutations.” A virus with one or several new mutations is  referred to as a “variant” of the original virus.

The more viruses circulate, the more they may change. These changes can occasionally result in a virus variant that is better adapted to its environment compared to the original virus. This process of changing and selection of successful variants is called “virus evolution.”

Some mutations can lead to changes in a virus’s characteristics, such as altered transmission (for example, it may spread more easily) or severity (for example, it may cause more severe disease).

Some viruses change quickly and others more slowly. SARS-CoV-2, the virus which causes COVID-19, tends to change more slowly than others such as HIV or influenza viruses. This could in part be explained by the virus’s internal “proofreading mechanism” which can correct “mistakes” when it makes copies of itself. Scientists continue to study this mechanism to better understand how it works.

462、**[Should I be concerned about SARS-CoV-2 changing?](https://www.who.int/emergencies/diseases/novel-coronavirus-2019/question-and-answers-hub/q-a-detail/sars-cov-2-evolution)**

It is normal for viruses to change, but it is still something scientists follow closely because there can be important implications. All viruses, including SARS-CoV-2, the virus that causes COVID-19, change over time. So far hundreds of variations of this virus have been identified worldwide. WHO and partners have been following them closely since January 2020.

Most changes have little to no impact on the virus’ properties. However, depending on where the changes are located in the virus’s genetic material, they may affect the virus’s properties, such as transmission (for example, it may spread more easily) or severity (for example, it may cause more severe disease).

WHO and its international network of experts, are monitoring changes to the virus so that if significant  mutations are identified, WHO can report any modifications to interventions needed by countries and individuals to prevent the spread of that variant.  The current strategies and measures recommended by WHO continue to work against virus variants identified since the start of the pandemic.

[See WHO’s Disease Outbreak News for reports on variants.](https://www.who.int/emergencies/disease-outbreak-news)

The best way to limit and suppress the transmission of COVID-19 is for people to continue taking the necessary precautions to keep themselves and others safe.

463、**[What is WHO doing to monitor and understand the changes in SARS-CoV-2?](https://www.who.int/emergencies/diseases/novel-coronavirus-2019/question-and-answers-hub/q-a-detail/sars-cov-2-evolution)**

Since the start of the outbreak, WHO has been working with a global network of expert laboratories around the world to support testing and better understanding of SARS-CoV-2, the virus that causes COVID-19.

Research groups have sequenced SARS-CoV-2 and shared these on public databases, including [GISAID](https://www.gisaid.org/). This global collaboration allows scientists to better track the virus and how it is changing.

WHO’s global SARS-CoV-2 laboratory network includes a dedicated SARS-CoV-2 Virus Evolution Working Group, which aims to detect new mutations quickly and assess their possible impact.

WHO recommends that all countries increase the sequencing of SARS-CoV-2 viruses where possible and share sequence data internationally to help one another monitor and respond to the evolving pandemic.

464、**[How does SARS-CoV-2 change when it infects animals, and what are the implications?](https://www.who.int/emergencies/diseases/novel-coronavirus-2019/question-and-answers-hub/q-a-detail/sars-cov-2-evolution)**

SARS-CoV-2 spreads primarily through human-to-human transmission, but there is evidence of transmission between humans and animals. Several animals like mink, dogs, domestic cats, lions, tigers and raccoon dogs have tested positive for SARS-CoV-2 after contact with infected humans.

There have been reports of large animal outbreaks in mink farms in several countries. SARS-CoV-2 can change while infecting minks. It has been observed that these mink variants are able to transmit back into humans through close contact with the mink. Preliminary results suggest that the mink variants infecting humans appear to have the same properties as other variants of the SARS-CoV-2 virus.

Further research is needed to better understand whether these mink variants will cause sustained transmission among humans and could have a negative impact on countermeasures, such as vaccines.

[See WHO’s Disease Outbreak News on Denmark ( 3 December 2020)](https://www.who.int/emergencies/disease-outbreak-news/item/2020-DON301)

WHO works closely with other organizations, such as the Food and Agriculture Organization of the United Nations, and the World Organisation for Animal Health, to evaluate situations of SARS-CoV-2 in animals and transmission occurring between animals and humans.

#### [Coronavirus disease (COVID-19): Working in hotels and other accommodation establishments（465-468）](https://www.who.int/emergencies/diseases/novel-coronavirus-2019/question-and-answers-hub/q-a-detail/coronavirus-disease-covid-19-working-in-hotels)

465、**[What can staff do to stay safe while working at a hotel or other accommodation establishment?](https://www.who.int/emergencies/diseases/novel-coronavirus-2019/question-and-answers-hub/q-a-detail/coronavirus-disease-covid-19-working-in-hotels)**

Everyone should follow basic precautions:

- Wash all parts of your hands frequently (at least 20 seconds if using an alcohol-based hand rub, and at least 40 seconds with soap and water), including after exchanging objects such as money or credit cards with guests.
- Cover a cough or sneeze with a bent elbow or tissue and throw away the tissue in a closed bin.
- Maintain at least a 1 metre distance from other staff and guests. This includes avoid hugging, kissing, or shaking hands. If you can’t guarantee the distance, wear a fabric mask. Be sure to check local and national guidelines on the use of masks.

Staying behind plexiglass boards at reception and concierge desks can prevent droplet transmission.

Teleworking may be a possibility for some jobs, which can help reduce physical contact with others.

466、**[Should staff wear a mask while working?](https://www.who.int/emergencies/diseases/novel-coronavirus-2019/question-and-answers-hub/q-a-detail/coronavirus-disease-covid-19-working-in-hotels)**

In areas where the virus that causes COVID-19 is circulating, staff aged 60 or over, or who have any underlying health conditions such as heart disease, diabetes or lung cancer, should wear a medical mask because of their higher risk of getting seriously ill from COVID-19.

Staff under the age of 60 and who are in general good health can wear fabric masks when they cannot guarantee at least a 1 metre distance from others. This is particularly important for staff who are in close contact or potential close contact with others. Everyone should follow local policies and regulations.

467、**[What should staff do if a guest becomes sick at an accommodation establishment?](https://www.who.int/emergencies/diseases/novel-coronavirus-2019/question-and-answers-hub/q-a-detail/coronavirus-disease-covid-19-working-in-hotels)**

If a guest at the accommodation develops symptoms of COVID-19, such as fever, dry cough or tiredness, staff at the accommodation establishment should contact the local health authority and follow their instructions. The sick person should be isolated in a room, alone, or at least 1 metre away from others, according to local health authorities’ instructions. No visitors should be permitted to enter the room occupied by the affected guest. Staff should also move people traveling with the sick person to a different room, if possible.

It is usually the relevant public health authority, not the management of the hotel and accommodation establishment, which has the authority to demand sick guests to temporarily remain in their room or to prevent them from receiving visits from other guests. National law will guide the rights of the guests to refuse or not the recommended measures.

Staff entering the room of an ill person should maintain at least a 1 metre distance from the ill person, and request that the ill person put on a medical mask.

If staff need to assist an ill guest within a 1 metre distance, they should clean their hands before putting on appropriate personal protective equipment (medical mask and eye protection, gloves and isolation gown), and clean their hands after providing assistance. Training should be provided on how to avoid contaminating themselves.

468、**[What should staff do if they have symptoms?](https://www.who.int/emergencies/diseases/novel-coronavirus-2019/question-and-answers-hub/q-a-detail/coronavirus-disease-covid-19-working-in-hotels)**

If staff develop COVID-19 symptoms while at work, such as fever, dry cough or tiredness, they should immediately stop working, put on a medical mask and isolate in a suitable room while medical services are notified. Disposable tissues and an appropriate waste bin with a lid should be available in the designated isolation area while waiting for medical assessment or transfer to an assessment facility.

If staff develop symptoms while at home, they should stay at home and seek medical attention, following instructions from a health worker that will include a period of time for self-isolation away from others, including family members, according to local guidance.  The staff member should inform the management accordingly.

#### [Global excess deaths associated with the COVID-19 pandemic（469-487）](https://www.who.int/emergencies/diseases/novel-coronavirus-2019/question-and-answers-hub/q-a-detail/global-excess-deaths-associated-with-the-COVID-19-pandemic)

469、**[What is excess mortality?](https://www.who.int/emergencies/diseases/novel-coronavirus-2019/question-and-answers-hub/q-a-detail/global-excess-deaths-associated-with-the-COVID-19-pandemic)**

Excess mortality associated with the COVID-19 pandemic is used to quantify the direct and indirect impacts of the pandemic. Excess mortality is defined as the difference between the total number of deaths estimated for a specific place and given time period and the number that would have been expected in the absence of a crisis (e.g., COVID-19 pandemic). This difference is assumed to include deaths attributable directly to COVID-19 as well as deaths indirectly associated with COVID-19 through impacts on health systems and society, minus any deaths that would have occurred under normal circumstances but were averted due to pandemic-related changes in social conditions and personal behaviours.

Estimates of the excess mortality associated with the COVID-19 pandemic includes deaths from all causes. In certain locations, these estimates may include excess deaths associated with other crises such as extreme weather, disasters, or conflicts.

470、**[Why is excess mortality the preferred measure?](https://www.who.int/emergencies/diseases/novel-coronavirus-2019/question-and-answers-hub/q-a-detail/global-excess-deaths-associated-with-the-COVID-19-pandemic)**

While aggregate COVID-19 case and death numbers are being reported to WHO, they do not always provide a complete picture of the health burden attributable to COVID-19. In general, reported death numbers under-estimate the number of lives lost due to the pandemic, there are several reasons for this. They miss those who died without testing, they are contingent on the country correctly defining COVID as the cause-of-death and they miss the increases in other deaths that are related to the pandemic leading to overwhelmed health systems or patients avoiding care. A few countries have experienced lower than expected total deaths during the pandemic due to reduced contact and reduced mobility, which have led to reduced infectious disease related mortality as well as reduced transport and injury related fatalities. Reported COVID-19 death numbers do not account for this.

In light of the challenges posed by using reported data on COVID-19 cases and deaths, excess mortality is considered a more objective and comparable measure that accounts for both the direct and indirect impacts of the pandemic.

471、**[Why do the estimates show negative excess deaths for some countries?](https://www.who.int/emergencies/diseases/novel-coronavirus-2019/question-and-answers-hub/q-a-detail/global-excess-deaths-associated-with-the-COVID-19-pandemic)**

Negative excess deaths could be observed if deaths that would have happened in the absence of the pandemic were averted due to measures taken to deal with the pandemic. Some public health measures (e.g., lockdown, social distancing, mask wearing, working from home) have led to decrease in the number of deaths from causes other than COVID-19. For instance, a decreased number of deaths due to road traffic injuries and seasonal flu has been observed due to restricted movement of people.

472、**[Why are the WHO estimates different from estimates produced by others?](https://www.who.int/emergencies/diseases/novel-coronavirus-2019/question-and-answers-hub/q-a-detail/global-excess-deaths-associated-with-the-COVID-19-pandemic)**

It is important to note that other groups and countries have produced excess death estimates. The difference in the estimates produced by WHO and those produced by other groups are due to key differences in the input data used and assumptions made including how the expected deaths are calculated, the statistical model used, and the variables used to predict deaths in locations where limited or no data is available. There might also be variations of the period/month/week assessed. To minimize these differences, uncertainty intervals are provided.

473、**[What are the challenges in calculating excess mortality including COVID-19?](https://www.who.int/emergencies/diseases/novel-coronavirus-2019/question-and-answers-hub/q-a-detail/global-excess-deaths-associated-with-the-COVID-19-pandemic)**

Mortality data to calculate actual deaths in real-time are available in only a subset of countries where reporting systems are functioning effectively, and historical datasets to calculate expected deaths are also often incomplete. Many countries do not have the mortality surveillance capacity to generate and collect data in a timely manner and these data gaps mean that excess mortality cannot be derived for all countries using standard methods.

The work of the TAG has been essential to establishing the methodology to model excess deaths where data has been unavailable and/or incomplete. This methodology is still under development and will likely be revised based on Member State feedback during the country consultation process.

474、**[Where can I review the methodology used to calculate the estimates for each country?](https://www.who.int/emergencies/diseases/novel-coronavirus-2019/question-and-answers-hub/q-a-detail/global-excess-deaths-associated-with-the-COVID-19-pandemic)**

The methodology paper can be found [here](https://www.who.int/publications/m/item/methods-for-estimating-the-excess-mortality-associatedwith-the-covid-19-pandemic). In light of the evolving situation surrounding the COVID-19 pandemic, the estimates will be updated periodically as more data becomes available.

475、**[Who are the members of the Technical Advisory Group (TAG) on COVID-19 Mortality Assessment?](https://www.who.int/emergencies/diseases/novel-coronavirus-2019/question-and-answers-hub/q-a-detail/global-excess-deaths-associated-with-the-COVID-19-pandemic)**

The TAG is co-chaired by Professor Debbie Bradshaw (Chief Specialist Scientist, South African Medical Research Council), Dr Kevin McCormack (Head of Division for Sustainable Development Goals Indicators & Reports and Geographies, Irish Central Statistics Office) and Dr Oleg Chestnov (Fellow, Federal Research Institute for Health Organization and Informatics of the Ministry of Health of the Russian Federation). Member profiles and a list of observers can be found [here](https://www.who.int/data/technical-advisory-group/covid-19--mortality-assessment/membership).

476、**[Is a ‘one size fits all’ model used to produce excess mortality estimates?](https://www.who.int/emergencies/diseases/novel-coronavirus-2019/question-and-answers-hub/q-a-detail/global-excess-deaths-associated-with-the-COVID-19-pandemic)**

WHO produces estimates using statistical modelling for all global health estimates, often with the advice of technical experts, to ensure robust statistical standards are followed and facilitate global comparability. This is the standard approach followed also by other UN agencies for global estimates. The model adopted for estimating excess deaths is not a ‘one-size-fits-all’ approach. WHO in collaboration with the TAG scrutinizes the relationships between excess mortality and certain contextually relevant variables in locations with good quality data to estimate excess mortality in countries with limited data.

This approach takes the specificity of countries (e.g., income level, reported COVID-19 deaths rate, test positivity rate, containment index) into consideration while ensuring global comparability. Countries may have their own approach to estimating excess deaths that may produce results that are different from those produced by WHO.

477、**[How are countries grouped in Tier 1 or 2 for the estimation approach?](https://www.who.int/emergencies/diseases/novel-coronavirus-2019/question-and-answers-hub/q-a-detail/global-excess-deaths-associated-with-the-COVID-19-pandemic)**

Tier classification is a simple grouping of countries based on mortality data availability. Countries are classified as Tier 1 countries if complete and nationally representative monthly all-cause mortality data for the specified period have been made available to WHO. Countries categorized as Tier 2 include countries for which WHO does not have access to the complete data and thus requires the use of alternative data sources or the application of scaling factors to generate the national aggregate. For these countries, WHO also utilizes subnational data where available.

Tier classification is not a classification of health systems but one specific for and only applied to this current exercise. It is not a reflection of the public health advancement of a country nor of the legislation in place for mortality surveillance. It concretely takes into consideration clear inclusion and exclusion criteria based on data that have been made available to WHO.

478、**[How are countries grouped to determine the age-sex pattern of excess deaths?](https://www.who.int/emergencies/diseases/novel-coronavirus-2019/question-and-answers-hub/q-a-detail/global-excess-deaths-associated-with-the-COVID-19-pandemic)**

In order to determine age-sex patterns of excess deaths, countries are categorized into eight groups using a *K*-means clustering approach. While a natural grouping would be geographically representative, using some regional identification (e.g., WHO regions), from a practical perspective it is not always possible owing to insufficient availability of empirical data in some countries or regions. Even within close geographic proximity, the impact of the pandemic, and its magnitude by sex and age can vary significantly.

*K*-means is a method commonly used to characterize data and partition a data set into groups. Countries are grouped into clusters based on mean ages at death and of population, as well as the overall excess mortality rates and porportion of total deaths. The number of clusters is chosen to maximise the variation between clusters and to minimise the variation within clusters by age and sex. More details on the *K*-means clustering are provided in the [methodology](https://www.who.int/publications/m/item/methods-for-estimating-the-excess-mortality-associatedwith-the-covid-19-pandemic).

479、**[What are the limitations of these estimates and methods?](https://www.who.int/emergencies/diseases/novel-coronavirus-2019/question-and-answers-hub/q-a-detail/global-excess-deaths-associated-with-the-COVID-19-pandemic)**

Every model is an approximation of reality. Models are subject to trade-offs, not least, the balance between comprehensiveness and comprehensibility. WHO’s estimates of the excess mortality associated with the COVID-19 pandemic are also approximations in a rapidly evolving pandemic with regard to transmissibility and severity. It is worth noting that WHO relies on models to compile a wide range of global health statistics. This is necessary as not all countries have high quality information systems, or those that do cannot always share their data in a timely manner. Therefore, WHO works with countries to strengthen their data and health information systems, while at the same time makes the best use of the data currently available to generate high quality evidence.

Specifically with regard to the excess mortality estimates, the model uses relationships that have been quantified using data from the countries with high quality systems for data reporting. We extrapolate these relationships to settings that are in many ways systematically different across multiple dimensions such as health systems capacity, underlying disease burden and age-structure. Countries have differed on when they experienced waves and how they responded, and quantifying this temporal relationships between COVID-19 surges or the effects of the emergence of variants is not trivial. In some settings the country responses were reactive but in many others they were proactive. As such, despite the effort to calibrate the model such that it is accurate in different settings, one of the more important limitations to note is that the input data have limited representation and this model generalizes the covariate relationships quantified to settings that may be systematically different from those observed. The final model is intentionally parsimonious. This reduction in complexity means it is not possible for such a reduced set of variables to explain all of the variation that is observed across countries. While the variables chosen are contextually relevant and are found to explain a significant component of the variation in excess, the underlying population characteristics that drove these differences are too complex to fully capture perfectly in a single global model and, hence, the trade-offs referred to above have had to be made. The estimates, therefore, are the best possible at this point in time for the first 24 months of the pandemic and have some degree of uncertainty around them.

480、**[What process did the WHO follow to calculate the estimates?](https://www.who.int/emergencies/diseases/novel-coronavirus-2019/question-and-answers-hub/q-a-detail/global-excess-deaths-associated-with-the-COVID-19-pandemic)**

In February 2021, in collaboration with the United Nations Department of Economic and Social Affairs (UN DESA), WHO convened a Technical Advisory Group (TAG) on COVID-19 Mortality Assessment to advise on the development of analytical methods for estimating excess mortality in all countries. The COVID TAG is comprised of leading demographers, epidemiologists, data and social scientists and statisticians from a range of backgrounds and geographies.

Under Working Group I of the TAG (Working Group I: Global excess mortality estimates including COVID-19), members considered several statistical models and after assessing performance, interpretability as well as extensibility, the TAG proposed a Poisson regression model (parameterized to account for over-dispersion) to predict the total number of deaths from all causes for the years 2020 and 2021, conditional on the monthly expected deaths over the period and a predicted relative rate parameter which is modelled using country-specific variables.

The model has been used by WHO to generate estimates for countries and regions for which adequate input data were available for reliable inference and to predict estimates for countries with no data available. In addition to determining levels of excess mortality associated with the COVID-19 pandemic for the years 2020 and 2021, the expertise of the TAG is also being leveraged to develop methods for disaggregating the estimated number of excess deaths by age and sex.

WHO’s estimates are produced following the Guidelines for Accurate and Transparent Health Estimates Reporting ([GATHER](http://gather-statement.org/)).

481、**[What is a country consultation process and what are countries be consulted on?](https://www.who.int/emergencies/diseases/novel-coronavirus-2019/question-and-answers-hub/q-a-detail/global-excess-deaths-associated-with-the-COVID-19-pandemic)**

It is a regular process that WHO consults with its Member States when producing new estimates. In August 2021, a Circular Letter (C.L. 29.2021) was shared through WHO’s official list of addresses with all Member States requesting the designation of a national focal point to interact with the technical team in this process.

Designated focal points for each Member State were requested to review estimates of excess mortality associated with the COVID-19 pandemic, the data sources and methods used and, to share primary data sources that may not have been previously available to WHO. Any additional feedback is also taken into consideration during the country consultation.

In October 2021, the draft estimates of excess mortality associated with the COVID-19 pandemic prepared for each country and the methodology applied to produce these estimates, were made available for download and accessible through WHO’s Country Portal.  The WHO estimates of excess mortality associated with the COVID-19 pandemic provide a comprehensive and comparable set of country estimates disaggregated by sex and age from January 2020 to December 2021.

Designated national focal points reviewed and uploaded supporting data and provided feedback on estimates via the WHO Country Portal. National focal points interacted with the WHO Global Health Estimates team. Regional webinars and a Mission Briefing (for Permanent Missions in Geneva) were organized to present the estimates and methodology and provided opportunity to respond to any questions.

482、**[Where can I find the estimates for my country?](https://www.who.int/emergencies/diseases/novel-coronavirus-2019/question-and-answers-hub/q-a-detail/global-excess-deaths-associated-with-the-COVID-19-pandemic)**

The global, regional, and country estimates for 2020 and 2021 were published in May 2022 and are available for download [here](https://www.who.int/data/sets/global-excess-deaths-associated-with-covid-19-modelled-estimates). The methodology to produce these estimates is available [here](https://www.who.int/publications/m/item/methods-for-estimating-the-excess-mortality-associatedwith-the-covid-19-pandemic).

483、**[What are the next steps?](https://www.who.int/emergencies/diseases/novel-coronavirus-2019/question-and-answers-hub/q-a-detail/global-excess-deaths-associated-with-the-COVID-19-pandemic)**

The estimates for 2020 and 2021 were published in May 2022. These estimates, including the full time series, will be revised following additional country consultations scheduled for later in 2022.

484、**[What is WHO's guidance on certifying COVID-19 deaths?](https://www.who.int/emergencies/diseases/novel-coronavirus-2019/question-and-answers-hub/q-a-detail/global-excess-deaths-associated-with-the-COVID-19-pandemic)**

A death due to COVID-19 is [defined](https://www.who.int/classifications/icd/Guidelines_Cause_of_Death_COVID-19.pdf) for surveillance purposes as a death resulting from a clinically compatible illness, in a probable or confirmed COVID-19 case, unless there is a clear alternative cause of death that cannot be related to COVID disease (e.g. trauma). There should be no period of complete recovery from COVID-19 between illness and death. A death due to COVID-19 may not be attributed to another disease (e.g. cancer) and should be counted independently of preexisting conditions that are suspected of triggering a severe course of COVID-19.

485、**[How is WHO supporting countries to strengthen national health information systems?](https://www.who.int/emergencies/diseases/novel-coronavirus-2019/question-and-answers-hub/q-a-detail/global-excess-deaths-associated-with-the-COVID-19-pandemic)**

At the heart of WHO’s Transformation Agenda is a commitment to support countries to strengthen their data and health information systems and make progress towards achieving the Triple Billion targets and health-related SDGs.

The SCORE for Health Data Technical Package (Survey, Count, Optimize, Review, Enable) identifies data gaps and provides countries with tools to close them. Based on findings from the first assessment of country health data and information systems, WHO is using an integrated approach to improve public health and disease surveillance, track Civil Registration and Vital Statistics (CRVS) data, and optimize routine health information systems, including regular and reliable data from health facilities.

The following tools are available to countries:

- The World Health Survey Plus (WHS+) is a multi-topic, multi-mode, multi-platform survey tool to gather health data quickly in standardized and cost effective ways to assess and improve health.
- The CRVS strategic implementation plan is focused on supporting countries so that over the next five years there will be substantial progress with accurate and timely tracking of births, deaths, and causes of death.
- WHO’s Routine Health Information Systems (RHIS) strategy aims to strengthen RHIS in countries through strengthened partnerships, improved data collection, and improved integration and interoperability of RHIS, along with building capacity and ensuring sustainability.
- The WHO Toolkit for Routine Health Information Systems Data strengthens facility data analysis through standardized indicators, visualizations, and guidance, while promoting integrated routing data platforms.
- The WHO Harmonized Health Facility Assessment (HHFA) is a comprehensive facility survey providing data on the availability of health services and the resources and systems needed to improve quality.

These data solutions enable public health decision makers to improve essential health services and better respond to emergencies.

For any questions, please contact: [healthstat@who.int](mailto:ddi@who.int)

486、**[Why is WHO producing these estimates now, in the midst of the pandemic, and what is its utility?](https://www.who.int/emergencies/diseases/novel-coronavirus-2019/question-and-answers-hub/q-a-detail/global-excess-deaths-associated-with-the-COVID-19-pandemic)**

WHO’s core normative function is to compile and disseminate statistics on mortality – numbers and causes. The world expects WHO to provide objective evidence on the impact of COVID-19.

Estimating excess mortality associated with the COVID-19 pandemic supplements the traditional direct measure ‘mortality directly attributable to COVID-19’ with a broader measure showing the direct plus the indirect effects of the pandemic.

It is important to do this now, rather than wait, in order to underpin the need for an equitable response during the pandemic by showing the real underlying impact of COVID-19 in different countries – especially lower and middle income countries that may not have developed health information systems to guide a targeted response.

Deaths directly attributable to COVID-19 provide only a narrow perspective of the wide ranging harms being caused by the pandemic. The collateral damage from COVID-19 is much wider. It is important to quantify this now as it can inform choices that governments must make regarding prioritization between routine and emergency health systems.

The utility of the excess mortality estimates goes beyond estimation of the impact of the COVID-19 pandemic. It underlines the significance of investing in health and better targeting of interventions and resources to those most in need to prevent future deaths. These estimates will serve to underscore to governments around the world the need to sharpen their data tools and specifically improve Civil Registration and Vital Statistics (CRVS), mortality surveillance, and data and health information systems.

Estimates of excess mortality associated with the COVID-19 pandemic are also critical inputs for other important work currently underway. For example, pandemic preparedness and estimating the global population. The UN is mandated to estimate the global population and prepare population projections. COVID-19 is a significant disruption to normal population growth trends and must be factored into any future estimates. Global excess mortality associated with the COVID-19 pandemic provides a key input to this work, showing the effects of the pandemic on the global population now, but also the longer tail effects on future population projections. The importance of this should not be underestimated as population is one of the most important denominators for economic and social statistics.

487、**[How is this work related to other global health and population estimates?](https://www.who.int/emergencies/diseases/novel-coronavirus-2019/question-and-answers-hub/q-a-detail/global-excess-deaths-associated-with-the-COVID-19-pandemic)**

The COVID-19 pandemic will have long lasting impacts on the structure of populations brought by changes in deaths and births. Substantial increases in mortality in many countries have affected premature mortality and life expectancy. WHO’s excess mortality estimates are critical inputs to WHO global health estimates and other UN partners’ work, including UN World Population Prospects.

The excess mortality estimates associated with the COVID-19 pandemic provide a valuable set of comparable country estimates to better understand the impact of the pandemic. It is thus of vital importance that these estimates are made available in a timely manner and updated periodically in order to identify inequalities and gaps in health information systems and civil registration and vital statistics (CRVS), which in turn will help in determining likely future vulnerabilities and target interventions.

For any questions, please contact: [healthstat@who.int](mailto:ddi@who.int)

#### [In vitro diagnostics: Frequently asked questions（488-498）](https://www.who.int/emergencies/diseases/novel-coronavirus-2019/question-and-answers-hub/q-a-detail/pqt-ivd-questions-and-answers)

488、**[How can I submit for EUL assessment?](https://www.who.int/emergencies/diseases/novel-coronavirus-2019/question-and-answers-hub/q-a-detail/pqt-ivd-questions-and-answers)**

The manufacturer should first contact [diagnostics@who.int](mailto:diagnostics@who.int).The Prequalification (PQ)-IVD team organizes a teleconference as the first step of the EUL process. Thereafter, we invite manufacturers to submit an application letter if the manufacturer has conducted all the required minimum studies in our instructions document. We prefer if manufacturers submit all information at one time rather than partial data at multiple timepoints as it assists us to undertake a more efficient data review. However, when this is not possible, manufacturers are encouraged to contact our Team to seek guidance. Once we receive the application letter, we will prepare a letter of agreement, and ask the manufacturer to submit their product dossier. The QMS documentation and product performance data are reviewed in parallel. Firstly, the provided information is screened for completeness and (if complete) then undergoes a technical assessment. We inform the manufacturer of the outcome of each step and ask for more information where required.

489、**[What are the EUL timelines?](https://www.who.int/emergencies/diseases/novel-coronavirus-2019/question-and-answers-hub/q-a-detail/pqt-ivd-questions-and-answers)**

The time to accepting the application letter is less than one week. Responses to screening and dossier review takes six to eight weeks depending on the completeness of the information submitted. If you have a question regarding your application that is currently under review, email [diagnostics@who.int](mailto:diagnostics@who.int) and we will give an update as soon as we can. For queries about active applications and products published on our website as emergency use listed, refer to the status updates and public reports on our website or email [diagnostics@who](mailto:diagnostics@who.int).int and we will try to give you as much information we can, understanding that we cannot share confidential information.

490、**[What IVD products are eligible for the abridged assessment pathway?](https://www.who.int/emergencies/diseases/novel-coronavirus-2019/question-and-answers-hub/q-a-detail/pqt-ivd-questions-and-answers)**

IVDs to detect SARS-CoV-2 nucleic acid that have US FDA Emergency Use Authorization are eligible for an abridged assessment pathway. For these applications, we accept the submission prepared for the FDA. Note however that any additional requirements outlined in the WHO instructions document must supplement the submission and the entire dossier will be assessed. We do not abridge any other national regulatory authority approvals for NAT assays (such as CE-marking, Chinese NMPA, ANVISA, Korea MFDS), nor any other types of IVDs.

491、**[We have conducted performance studies for our kit as per national regulatory authority requirements. Do we have to repeat these studies for the EUL if they do not completely align with WHO EUL instructions?](https://www.who.int/emergencies/diseases/novel-coronavirus-2019/question-and-answers-hub/q-a-detail/pqt-ivd-questions-and-answers)**

We understand that time and specimens are critical. We will work with the data that you have available at the time of submission and discuss your application in detail during the presubmission call. Submit any data that has been generated that can help us evaluate your assay more comprehensively. We publish minimum requirements in our instructions document that we require all manufacturers to meet. As the pandemic evolves and more evidence becomes, we are updating our minimum requirements. The most recent version is posted on our website.

492、**[What is the relationship between the performance studies such as the study organized by FIND and the EUL?](https://www.who.int/emergencies/diseases/novel-coronavirus-2019/question-and-answers-hub/q-a-detail/pqt-ivd-questions-and-answers)**

Currently, several performance evaluations of SARS-CoV-2 IVDs are being implemented by regulatory authorities, reference laboratories and other stakeholders in various regions. Manufacturer are strongly encouraged to participate in initiatives which generate evidence that can be used to support the EUL submission, However, participation in external evaluations does not replace the EUL submission nor is participation in such studies mandatory for submission to the WHO EUL.

493、**[Does the EUL expression of interest include multiplex NAT assays which detect other respiratory pathogens in addition to SARS-CoV-2?](https://www.who.int/emergencies/diseases/novel-coronavirus-2019/question-and-answers-hub/q-a-detail/pqt-ivd-questions-and-answers)**

PQT-IVD are not accepting applications for multiplex NAT assays that detect other respiratory pathogens in addition to SARS-CoV-2 to the EUL assessment procedure.

494、**[I am developing a SARS-CoV-2 assay and preparing to submit to the EUL. What instructions should I follow?](https://www.who.int/emergencies/diseases/novel-coronavirus-2019/question-and-answers-hub/q-a-detail/pqt-ivd-questions-and-answers)**

We have published different instructions depending on the assay type on our website to assist in the preparation of the EUL submission. As the pandemic evolves and more scientific evidence becomes PQT_IVD_Q&A_200806version1 Page 3 of 3 available, these instructions may change. It is recommended to verify that you are using the most recent version of instructions which are posted on our [website](https://www.who.int/teams/regulation-prequalification/eul).

495、**[I am submitting a RT-PCR kit to detect SARS-CoV-2 nucleic acid, do I have to include the extraction step in my validation studies?](https://www.who.int/emergencies/diseases/novel-coronavirus-2019/question-and-answers-hub/q-a-detail/pqt-ivd-questions-and-answers)**

Yes, the extraction step must be considered for all analytical and clinical performance studies. It is essential that the limit of detection is determined in each specimen type claimed using all extraction kits recommended in the instructions for use.

496、**[Do I need to provide detailed information regarding specimen collection and storage?](https://www.who.int/emergencies/diseases/novel-coronavirus-2019/question-and-answers-hub/q-a-detail/pqt-ivd-questions-and-answers)**

More and more evidence is emerging as to how critical appropriate specimen collection and storage is in the testing process, and the potential impacts it can have on the IVD kit sensitivity. PQT-IVD consider it essential that the manufacturer fully understands the impact of the viral transport media (VTM) or other means recommended in the instructions for use as users will follow this information. If commercial VTM, in-house developed VTM or other storage solutions are recommended, it is essential that the manufacturer has evidence of its performance in the claimed specimen type

497、**[I cannot get access to the full list of organisms that are required for NAT assay cross- reactivity studies. Can WHO source these specimens or conduct testing on our behalf?](https://www.who.int/emergencies/diseases/novel-coronavirus-2019/question-and-answers-hub/q-a-detail/pqt-ivd-questions-and-answers)**

PQT-IVD team are not undertaking any laboratory evaluation on manufacturers behalf. However, we understand that some organisms are proving difficult to source in different jurisdictions. As long as manufacturers can demonstrate that they have made adequate attempts to source these specimens, we can accept the testing to be submitted at an agreed later timepoint when the manufacturer can access these specimens. We will review the overall dataset and accept that there might be some small gaps in the dossier where other sections have been addressed completely.

498、**[In our clinical evidence studies, we have not used a comparator assay that is recommended in your instructions document. Do we have to repeat the clinical studies?](https://www.who.int/emergencies/diseases/novel-coronavirus-2019/question-and-answers-hub/q-a-detail/pqt-ivd-questions-and-answers)**

No, we will not necessarily request you to repeat your clinical evidence studies without reviewing the data you already have available. Please provide a justification for how you have chosen your comparator assay. We would expect that a minimum, that you have knowledge of the comparator assay primers and probes and that they are not the same sequences as used in your assay. As part of the dossier submission. PQT-IVD will consider this information as part of the total dossier.

#### [Pandemic prevention, preparedness and response accord（499-513）](https://www.who.int/emergencies/diseases/novel-coronavirus-2019/question-and-answers-hub/q-a-detail/pandemic-prevention--preparedness-and-response-accord)

499、**[What is meant by a  ‘convention, agreement or other international instrument’?](https://www.who.int/emergencies/diseases/novel-coronavirus-2019/question-and-answers-hub/q-a-detail/pandemic-prevention--preparedness-and-response-accord)**

Conventions, framework agreements and treaties are all examples of international instruments, which are legal agreements made between countries that are binding.

There are international instruments addressing a broad range of topics, including tobacco control, nuclear, chemical and biological weapons, climate change and many other threats to our shared security and well-being.

A key international instrument on international health, rooted in the WHO Constitution, is the International Health Regulations (2005), which was established “to prevent, protect against, control and provide a public health response to the international spread of disease in ways that are commensurate with and restricted to public health risks, and which avoid unnecessary interference with international traffic and trade”.

500、**[Why did WHO’s Member States decide to create an accord for pandemic preparedness and response?](https://www.who.int/emergencies/diseases/novel-coronavirus-2019/question-and-answers-hub/q-a-detail/pandemic-prevention--preparedness-and-response-accord)**

In light of the impact of the COVID-19 pandemic, WHO’s 194 Member States established a process to draft and negotiate a new convention, agreement, or other international instrument (referred to in the rest of this FAQ, generally, as an “accord”) on pandemic preparedness and response. This was driven by the need to ensure communities, governments, and all sectors of society – within countries and globally – are better prepared and protected, in order to prevent and respond to future pandemics. The great loss of human life, disruption to households and societies at large, and impact on development are among the factors cited by governments to support the need for lasting action to prevent a repeat of such crises.

At the heart of the proposed accord is the need to ensure equity in both access to the tools needed to prevent pandemics (including technologies like vaccines, personal protective equipment, information and expertise) and access to health care for all people.

501、**[Why are such international agreements and instruments needed in global public health?](https://www.who.int/emergencies/diseases/novel-coronavirus-2019/question-and-answers-hub/q-a-detail/pandemic-prevention--preparedness-and-response-accord)**

WHO Member States have developed multiple global legally-binding agreements, conventions, accords, and other types of international instruments to protect and promote people’s health, including the WHO Constitution itself, the WHO Framework Convention on Tobacco Control, and the International Health Regulations.

Such instruments were created by Member States to secure and foster further collaboration in multiple areas that impact on the health and well-being of people in communities, countries and globally.

These international instruments represent a commitment by countries of the world to address the health needs of their citizens to advance their health status and strengthen the socio-economic status of their communities at large.

502、**[What is the process underway to draft and negotiate the accord?](https://www.who.int/emergencies/diseases/novel-coronavirus-2019/question-and-answers-hub/q-a-detail/pandemic-prevention--preparedness-and-response-accord)**

The process to consider such an international accord for pandemic prevention, preparedness and response is being led fully by WHO’s Member States.

[In December 2021](https://www.who.int/news/item/01-12-2021-world-health-assembly-agrees-to-launch-process-to-develop-historic-global-accord-on-pandemic-prevention-preparedness-and-response), WHO’s Member States decided at a special session of the World Health Assembly – WHO’s highest decision-making body, comprised of all of its 194 sovereign member countries – to establish an intergovernmental negotiating body (INB), representing all regions of the world, to draft and negotiate a WHO convention, agreement, or other international instrument on pandemic prevention, preparedness and response, with a view to adoption under Article 19 of the [WHO Constitution](https://apps.who.int/gb/bd/PDF/bd47/EN/constitution-en.pdf), or other provisions of the Constitution as may be deemed appropriate by the INB.

Article 19 gives the 194 Member States forming the Health Assembly the authority to adopt conventions or agreements on any matter within WHO’s competence. The sole instrument established under Article 19 to date is the [WHO Framework Convention on Tobacco Control](https://apps.who.int/iris/handle/10665/42811), which has made a significant and rapid contribution to protecting people from tobacco since its entry into force in 2005.

The World Health Assembly decision establishing the Intergovernmental Negotiating Body (INB) and its work on this new international accord stressed that WHO’s Member States, who will be working in their sovereign capacity, should guide their efforts by the principle of solidarity with all people and countries, and that the accord should frame practical actions to deal with both causes and consequences of pandemics and other health emergencies.

In support of the INB process, inputs were solicited from the main constituencies as follows:

- written and oral inputs from Member States and relevant stakeholders on the successive iterations of the work, including the Substantive Elements, the Working Draft, the Conceptual Zero Draft and the Zero Draft;
- regional consultations during the WHO Regional Committees of 2022;
- informal, focused consultations on selected key issues, including with experts, in the second half of 2022; and
- public hearings (two rounds, in April and September 2022), for interested parties and stakeholders to express their views.

### From the Conceptual Zero Draft to the Zero Draft

The Conceptual Zero Draft (CZD), presented at the [third meeting of the INB](https://apps.who.int/gb/inb/e/e_inb-3.html), in December 2022, was not intended as an official/formal draft of the accord and was not presented for “negotiation”, “agreement” or otherwise to be “approved” by WHO’s Member States. It was intended as a “bridge”, for reflexion on, by Member States, between the Working Draft presented at the Second meeting of the INB and the Zero Draft, to be considered at the fourth meeting of the INB, beginning on 27 February 2023.

After reviewing the CZD at the third meeting of the INB in December 2022, Member States agreed that the INB Bureau should move ahead with developing a Zero Draft (ZD) of a potential pandemic accord based on inputs received.

The Zero Draft is available on the [INB website](https://inb.who.int/home/inb-process) in all six official UN languages.

[Zero draft of the pandemic accord](https://apps.who.int/gb/inb/pdf_files/inb4/A_INB4_3-en.pdf)

The INB has been requested to deliver a progress report to the 76th World Health Assembly in 2023 - which is expected to be of a procedural nature and to be addressed at the fifth meeting of the INB.

The INB is expected to submit a draft pandemic accord for consideration by the Seventy-seventh World Health Assembly in 2024.

503、**[What is the role of the WHO Secretariat role in the accord process?](https://www.who.int/emergencies/diseases/novel-coronavirus-2019/question-and-answers-hub/q-a-detail/pandemic-prevention--preparedness-and-response-accord)**

In December 2021, WHO’s Member States requested the WHO Director-General to convene the meetings of the Intergovernmental Negotiating Body and support its work, including by facilitating the participation of other United Nations system bodies, non-state actors, and other relevant stakeholders in the process to the extent decided by the 194 Member States forming part of the negotiations.

The WHO Secretariat’s job is to support countries – its Member States – as they negotiate and agree on the new international accord. The WHO Secretariat does not determine the contents of any possible international accord.

504、**[Who else is involved in the process for the accord?](https://www.who.int/emergencies/diseases/novel-coronavirus-2019/question-and-answers-hub/q-a-detail/pandemic-prevention--preparedness-and-response-accord)**

Besides WHO Member States, the process for developing a possible new accord is providing extensive opportunities for engagement with relevant stakeholders, including other United Nations system bodies, and a wide range of other non-State actors in official relations with the WHO, to ensure robust and inclusive participation in the proceedings of the Intergovernmental Negotiating Body. Furthermore, WHO is seeking complementary inputs through [public hearings](https://inb.who.int/home/public-hearings) with stakeholders including: international organizations; civil society; the private sector; philanthropic organizations; scientific, medical, public policy and academic institutions and other entities with relevant knowledge, experience and/or expertise.

505、**[What is the timeline for developing the pandemic accord?](https://www.who.int/emergencies/diseases/novel-coronavirus-2019/question-and-answers-hub/q-a-detail/pandemic-prevention--preparedness-and-response-accord)**

Member States [meet in person on a regular basis](https://apps.who.int/gb/inb/index.html) as part of the Intergovernmental Negotiating Body (INB).

Member States convening as part of the INB conducted a first phase of their work between February and June 2022 with the aim of agreeing on the way they would work, and defining how best to engage various stakeholders in an inclusive manner. The INB shared the report of this first phase of its work through a global public live [webcast.](https://apps.who.int/gb/inb/e/e_inb-1-second-resumed-session.html)

Member States started the second phase of their work as part of the INB in July 2022. The second meeting of the INB, on 18-22 July 2022, was [webcast.](https://apps.who.int/gb/inb/e/e_inb-2.html)

A Conceptual Zero Draft (CZD) of the accord was discussed at INB3 in December 2022 and it was agreed that the INB Bureau should move forward with producing a Zero Draft of the accord.

That Zero Draft (ZD) was shared with Member States on 1 February 2023 and, if agreed to by Member States at the fourth meeting of the INB, beginning on 27 February 2023, it will become the basis for negotiations by Member States on a pandemic accord.

The Zero Draft will be considered by WHO Member States and relevant stakeholders at INB4 on 27 February – 3 March 2023 and INB5 on 3-6 April 2023.

The INB will submit its outcomes for consideration by the Seventy-seventh World Health Assembly in 2024, with a progress report to the Seventy-sixth World Health Assembly in 2023.

506、**[What could the accord on pandemic prevention, preparedness and response do?](https://www.who.int/emergencies/diseases/novel-coronavirus-2019/question-and-answers-hub/q-a-detail/pandemic-prevention--preparedness-and-response-accord)**

As the process of the Intergovernmental Negotiating Body is solely the decision of WHO’s 194 sovereign Member States, WHO’s Member States will ultimately determine the form and content of the new accord, including its objectives, principle(s), and scope.

The new accord could represent a global commitment to work together, as an international community, to help prevent disease outbreaks from impacting individuals, communities, countries and the world in the same way as the COVID-19 pandemic did.

Importantly, any new accord would be expected to establish principles, priorities and targets for pandemic preparedness and response, with the aim to:

- build resilience to pandemics;
- support prevention, detection, and responses to outbreaks with pandemic potential;
- ensure equitable access to pandemic countermeasures; and
- support global coordination through a stronger and more accountable WHO.

The new accord could complement other initiatives, actions and measures aimed at making the world safer from pandemics, including the International Health Regulations and global institutions and systems working to equitably share health technologies, information and expertise.

The proposed accord could take the lessons learned from the COVID-19 pandemic and use them to build back better. Equity is one of the key principles being discussed as part of the work on the new accord.

A new accord could promote political commitment at the highest level, through ensuring an all-of-government and whole-of-society approach within countries, and sustained and sufficient political and financial investment within and among countries.

507、**[What gaps could such an accord address?](https://www.who.int/emergencies/diseases/novel-coronavirus-2019/question-and-answers-hub/q-a-detail/pandemic-prevention--preparedness-and-response-accord)**

Among other things, the new accord could, if WHO’s Member States so decide, address gaps that have been highlighted by the COVID-19 pandemic, including the following key areas for action:

- global preparedness and response arrangements – including at the human-animal interface – to help anticipate and prevent future pandemics and address them more effectively when they do arise;
- sustained, predictable funding for health emergency preparedness and response, including from domestic budgets to support preparedness measures and help ensure that the world is prepared and can respond to the emergence of dangerous pathogens; and
- governance and oversight mechanisms to increase trust, ensure accountability and foster transparency.

508、**[How will the new accord relate to the International Health Regulations (IHR)?](https://www.who.int/emergencies/diseases/novel-coronavirus-2019/question-and-answers-hub/q-a-detail/pandemic-prevention--preparedness-and-response-accord)**

The [International Health Regulations](https://www.who.int/health-topics/international-health-regulations#tab=tab_1) (2005) (the “IHR”) is a key international instrument on international health, rooted in the WHO Constitution. The IHR was established to prevent, protect against, control and provide a public health response to the international spread of disease in ways that are commensurate with and restricted to public health risks, and which avoid unnecessary interference with international traffic and trade.

The work on the new accord on pandemic prevention, preparedness and response would aim to be coherent with, and complement, the IHR. The Health Assembly decision SSA2(5) establishing the INB noted the “need for coherence and complementarity between the process of developing the new instrument and the ongoing work […] regard to implementation and strengthening of the IHR (2005)”.

A process agreed on by the World Health Assembly at its meeting in May 2022 is underway to consider potential “targeted” amendments to the IHR. This work is being conducted through a dedicated Member-State led working group process (the Working Group on Amendments to the IHR (2005)) which held its [first meeting](https://apps.who.int/gb/wgihr/e/e_wgihr-1.html) on 14-15 November 2022.

The World Health Assembly [decision](https://apps.who.int/gb/ebwha/pdf_files/WHA75/A75(9)-en.pdf) establishing the above-mentioned Working Group requested the Working Group “to coordinate with the process of the [INB], by means that include regular coordination between the two respective Bureaux and alignment of meeting schedules and workplans, as both the International Health Regulations (2005) and the new instrument are expected to play central roles in pandemic prevention, preparedness and response in the future.”

509、[**The proposed accord may focus on “pandemics”; is that term internationally defined, and how do “pandemics” fit with the International Health Regulations (IHR)?**](https://www.who.int/emergencies/diseases/novel-coronavirus-2019/question-and-answers-hub/q-a-detail/pandemic-prevention--preparedness-and-response-accord)

It could be anticipated that a new accord could define the term “pandemic” as part of its terms, to ensure clarity with respect to its scope of application, if Member States so decide. It is also possible that the new accord could include a provision on its relationship with other international instruments, including clarifying that the new accord could be complementary to the IHR. Article 57 of the [IHR](https://apps.who.int/iris/handle/10665/246107) also states that its Parties may conclude special treaties or arrangements in order to facilitate the implementation of the IHR.

510、[**How can the general public keep informed of what happens at the Intergovernmental Negotiating Body meetings, and of governments’ decisions and positions?**](https://www.who.int/emergencies/diseases/novel-coronavirus-2019/question-and-answers-hub/q-a-detail/pandemic-prevention--preparedness-and-response-accord)

The Intergovernmental Negotiating Body is, per the World Health Assembly decision, operating based on the principles of inclusiveness, transparency, efficiency, Member State leadership and consensus. Public updates are provided at all relevant steps of the process. The WHO website is the main source of information.

A report will be submitted to the World Health Assembly in 2023. The final decision on whatever pandemic accord is agreed will happen in open plenary in the World Health Assembly in 2024.

[More information on the INB](https://inb.who.int/)

511、[**How much authority could an accord have over signatory countries? Will it be legally binding? Will it take sovereignty away from signatory countries?**](https://www.who.int/emergencies/diseases/novel-coronavirus-2019/question-and-answers-hub/q-a-detail/pandemic-prevention--preparedness-and-response-accord)

The Zero Draft presented by the INB Bureau based on progress achieved and input received at the third meeting of the Intergovernmental Negotiating Body (INB) refers to a number of potential guiding principles and rights for the new accord, including the importance of national sovereign rights and full respect for the dignity, human rights and fundamental freedoms of persons.

As with all international instruments, any new accord, if and when agreed by Member States, would be determined by governments themselves, who would take any action while considering their own national laws and regulations.

Member States will decide the terms of the accord, including whether any of its provisions will be legally binding on Member States as a matter of international law.

It is expected that such an accord would aim to help prevent future disease outbreaks from impinging on people’s freedom to travel, work, seek education and, above all, lead a healthy life free of avoidable disease, as called for by another global accord, the WHO Constitution.

512、[**Would the accord be open to all countries to join or participate in?**](https://www.who.int/emergencies/diseases/novel-coronavirus-2019/question-and-answers-hub/q-a-detail/pandemic-prevention--preparedness-and-response-accord)

This would be a decision of WHO’s Member States, working through the intergovernmental negotiating body. It would be expected that a new accord would be open to the participation of all countries, who would be able to participate if they so wished. In line with the example provided by the WHO Framework Convention on Tobacco Control, the new accord could, possibly, be open to regional economic integration organizations.

513、[**What could happen if countries that join or participate in any new accord do not meet their obligations?**](https://www.who.int/emergencies/diseases/novel-coronavirus-2019/question-and-answers-hub/q-a-detail/pandemic-prevention--preparedness-and-response-accord)

It would be up to Member States to decide if and what compliance mechanisms would be included in the new accord on pandemic preparedness and response. It is a general principle of international law that once an international law instrument is in force, it would be binding on the parties to it, and would have to be performed by those parties in “good faith.”
